# Thymic exosomes promote the final maturation of thymocytes

**DOI:** 10.1038/srep36479

**Published:** 2016-11-08

**Authors:** Vanja Lundberg, Martin Berglund, Gabriel Skogberg, Susanne Lindgren, Christina Lundqvist, Judith Gudmundsdottir, Karolina Thörn, Esbjörn Telemo, Olov Ekwall

**Affiliations:** 1 Dept of Rheumatology and Inflammation Research, Institute of Medicine, Sahlgrenska Academy, University of Gothenburg, Sweden; 2 Dept of Pediatrics, Institute of Clinical Sciences, Sahlgrenska Academy, University of Gothenburg, Sweden

## Abstract

Extensive knowledge has been gained the last years concerning mechanisms underlying the selection of single positive thymocytes in the thymic medulla. Less is known regarding other important processes in the thymic medulla such as the regulation of late stage thymocyte maturation. We have previously reported that exosomes are abundant in the thymus with a phenotype that indicates an epithelial cell origin and immunoregulatory properties. In this study we use an *in vitro* system to investigate the effects of thymic exosomes on the maturation of single positive thymocytes as well as effects on nTreg formation. We show that thymic exosomes promote the maturation of single positive CD4^+^CD25^−^ cells into mature thymocytes with S1P_1_^+^Qa2^+^ and CCR7^+^Qa2^+^ phenotypes. Furthermore, we show that thymic exosomes reduce the formation of CD4^+^CD25^+^FoxP3^+^ thymocytes and that these exosome effects are independent of dendritic cell co-stimulation but require intact exosomal RNA content and surface proteins. An efficient direct uptake of exosomes by both thymocytes and thymic DC’s is also demonstrated. In conclusion, this study demonstrates that exosomes may represent a new route of communication within the thymus.

The thymus is a primary lymphoid organ responsible for the generation of a self-tolerant and diverse population of T cells from bone marrow precursors. After entering the thymus the hematopoietic progenitors undergo several differentiation steps in the thymic cortex in which CD4^−^CD8^−^ double negative thymocytes differentiate into CD4^+^CD8^+^ double positive cells, which are subject to positive selection resulting in single positive (SP) CD4^+^CD8^−^ or CD4^-^CD8^+^ cells entering the medulla. In the medulla, negative selection eliminates most self-reactive SP thymocytes, but some are rescued to form the nTreg population. The selected SP thymocytes undergo further maturational steps before exiting to the periphery^1^. Thymic stromal cells are indispensable for thymocyte differentiation and selection^2^. A key stromal cell population is the medullary thymic epithelial cells (mTECs), which express many otherwise tissue-restricted antigens (TRAs) that are crucial for the negative selection process^3^,^4^, and for the formation of the nTreg population. The expression of TRAs is in part under the control of the autoimmune regulator (Aire), but also controlled by the transcription factor Fez2f 5,6. Aire has also been implicated to be important for antigen transfer from mTECs to dendritic cells (DCs) as well as for regulation of the expression of mTEC specific miRNAs important for TRA expression and TEC maturation^7^,^8^. Antigen transfer from TECs to DCs and thymocytes as well as intercellular sharing of miRNA within the thymic microenvironment might be prerequisites for optimal thymic function. We and others have suggested that exosomes could shuttle antigens as well as miRNA within the thymus^9^,^10^.

Exosomes are membrane-enclosed nano-sized vesicles of endocytic origin. Cells secrete exosomes into the extracellular space by the fusion of multivesicular bodies (MVBs) with the cell plasma membrane^11^. The biological significance of exosomes is still debated although their potential role in cell communication has been recognized for the presentation of antigenic peptides^12^ and shuttling of mRNAs and miRNAs between cells^13^. Furthermore, intestinal epithelial derived exosomes have been shown to mediate MHC class II-dependent immune tolerance to dietary antigens^14^. The presence of exosomes is established both in the murine and human thymus, but their function is less well studied^15^,^16^.

While the mechanisms underlying the medullar selection process are relatively well studied, the knowledge of the regulation of final thymocyte maturation and thymic egress is still scarce. Following positive selection thymocytes up-regulate CCR7 and relocate to the thymic medulla in response to the increased concentration of CCL19 and CCL21, mainly produced by mTECs^4^,^17^,^18^,^19^. Furthermore, the thymocytes change their gene expression profile and up-regulate genes involved in late stage maturation, thymic egress and extrathymic functions. One such gene is the Kruppel-like factor 2 (KLF2), which drives the gene expression of both S1P_1_ and CD62L in SP thymocytes^20^. Whereas CD62L is important for the homing of mature T-lymphocytes to secondary lymphoid organs^21^, S1P expressed by neural crest-derived pericytes on the vessel wall bind S1P_1_ on mature thymocytes and thereby promote their egress at the corticomedullary junction^22^,^23^,^24^,^25^. Qa2 is a non-classical MHC class I molecule used as a marker for thymocyte maturation and expression of Qa2 is up-regulated in the final SP4 stage of thymocyte development just before their exit to the periphery^26^,^27^.

In this report we investigate the effects of thymic exosomes on the late stage maturation of CD4^+^ single positive thymocytes using an *in vitro* system. We demonstrate that thymic exosomes stimulate maturation of CD4^+^CD25^−^ SP thymocytes into an S1P_1_^+^Qa2^+^ and S1P_1_^+^CCR7^+^ phenotype and reduce the formation of CD25^+^FoxP3^+^ thymocytes.

## Results

### Characterization of thymic exosomes

Zetaview analysis revealed a heterogeneous thymic exosome population with a typical size range of 50–200 nm. Flow cytometry confirmed surface expression of the known exosome markers CD9, TSG101, MFGE8, MHCII and Lamp-1. Furthermore, expression of TGFβ was detected, which may indicate that thymic exosomes could be involved in generation of thymic regulatory T cells (nTregs). To study the origin of the thymic exosomes, we analyzed surface markers for thymic epithelial cells (EpCAM), thymocytes (CD3) and DCs (CD11c). Interestingly, there was a positive shift for EpCAM, whereas CD3 and CD11c were barely detectable, which indicates that the majority of thymic exosomes origin from TECs (Fig. S1).

### The protein content of thymic exosomes suggests a large contribution of exosomes with an epithelial cell origin to the thymic exosome pool

**Figure 1:**
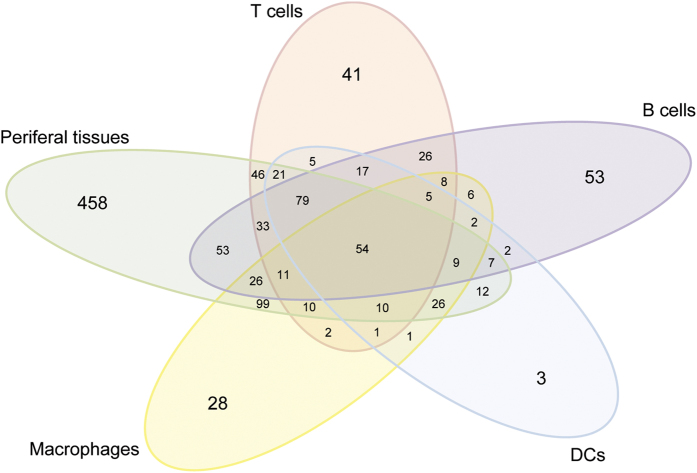
Proteomic analysis of thymic exosomes. Following isolation and proteomic analysis of thymic exosomes, the resulting 1531 proteins were grouped into 5 different cell categories based on the BioGPS database. Data was visualized using a Venn diagram as; peripheral tissues (green), T cells (red), B cells (purple), macrophages (yellow) or DCs (blue). A total of 377 proteins could not be allocated to any of these cell categories.

The analysis of the protein content of thymic exosomes from 8 C57BL/6 mice using mass spectrometry identified 1556 proteins (Table S2). Based on expression data from BioGPS database (http://ds.biogps.org/), the proteins were divided into 62 different categories that were further grouped into five different main categories representing possible cellular sources of exosomes in the thymus; peripheral tissues, B cells, T cells, macrophages and DCs. 458 proteins (29.9%) were specifically expressed in different peripheral tissues, 53 (3.5%) in B cells, 41 (2.7%) in T cells, 28 (1.8%) in macrophages and 3 (0.2%) in DCs. 54 proteins (3.5%) were expressed in all five categories, whereas 377 proteins (24.6%) were expressed in none of the categories (Fig. 1). Since the expression of peripheral tissue antigens is a hallmark for thymic epithelial cells, especially mTECs, the presence of proteins normally expressed in peripheral tissues can be regarded as an indicator of an epithelial cell origin of a fraction of the thymic exosomes.

Typical exosomal markers such as Lamp1, Annexin I, II and V and several Ras-related proteins (Rab) were present^28^. The identification of cytokeratins with a known TEC expression, e.g. K5, K8 and K14 further supports a TEC origin of a proportion of the thymic exosomes. Furthermore, thymic exosomes contain TRAs with a previously described mTEC expression, such as Fatty acid binding protein (Fabp), Alpha actin (Actc1), Fibrinogen (Fbg), Alpha-enolase (Eno1) and Antitrypsin (Serpina1b). In addition, we could detect proteins involved in thymocyte maturation and egress, including Sphingosine-1-phosphatase lyase (SGPL1), Dedicator of cytokinesis protein 2 (DOCK2), Rho GDP-dissociation inhibitor 1 (GDIR1) and p21 protein-activated kinase 2 (PAK2)^29^,^30^,^31^,^32^ (Table S2).

Taken together, these data suggests a mixed cellular origin of the thymic exosomes and that a significant fraction of the exosomes has an epithelial origin.

### miRNA analysis of thymic exosomes

Using microarray screening we identified 1175 different miRNA in thymic exosomes, compared to 1086 miRNA in the spleen exosome preparation (Fig. S1, Tables S3 and S4). Of the miRNAs found in exosomes from thymus and spleen 104 were unique for thymic exosomes and 15 were exclusively found in splenic exosomes. When comparing the expression ratios between thymic and splenic exosomes we Identified 450 miRNA that were expressed more than twofold, and 34 miRNA expressed more than 10-fold in thymic exosomes. In spleen exosomes 147 miRNA were expressed more than twofold and 14 miRNA expressed more than 10-fold as compared with thymic exosomes (Fig. S1).

### Efficient uptake of exosomes by SP CD4^+^ thymocytes and DCs

**Figure 2:**
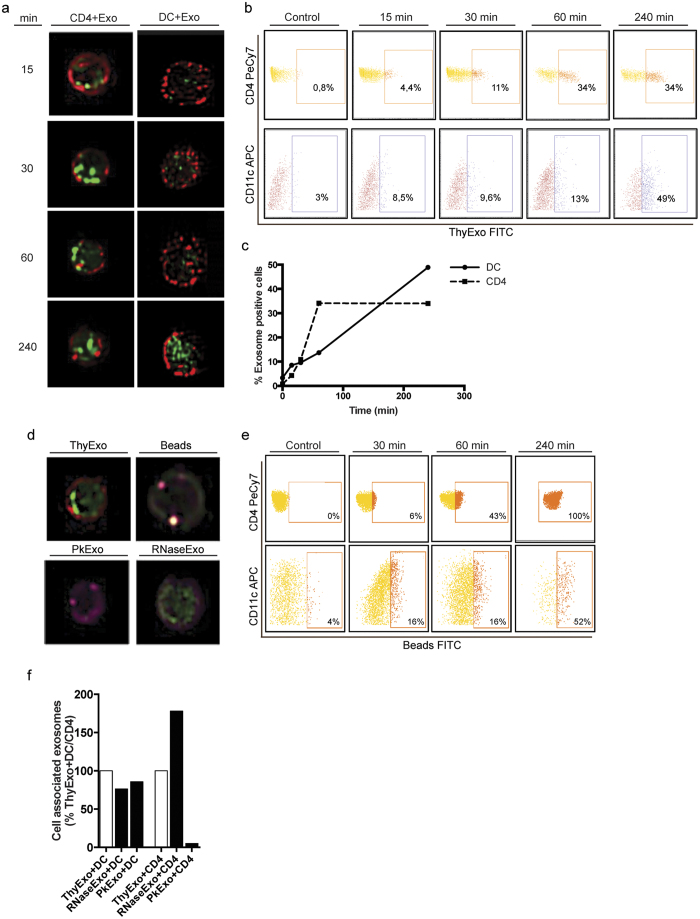
Exosomes are taken up by thymic DCs and CD4^+^ thymocytes. SP CD4^+^ thymocytes and thymic DCs were isolated and stained with CD4 PE-Cy7 and CD11c APC, respectively. After addition of FITC-stained thymic exosomes, cells were harvested after 15, 30, 60 and 240 min, and analyzed on an ImageStream X Mark II, imaging flow cytometry. (**a**) Representative images of SP CD4^+^ thymocytes (red, left) and thymic CD11c^+^ DCs (red, right) at indicated time points (min) after addition of FITC-stained thymic exosomes (green). (**b**) Representative cytometry plots of SP CD4^+^ thymocytes (top) or CD11c^+^ thymic DCs (bottom) at indicated time points after addition of thymic exosomes. (**c**) Kinetics of exosome uptake by SP CD4^+^ thymocytes (dashed line) and thymic DCs (solid line). (**d**) Representative images of SP CD4^+^ thymocytes (red) 240 min after addition of untreated FITC-stained thymic exosomes (green, top left), FITC stained 100 nm latex beads (green, top right), FITC stained thymic exosomes treated with Proteinase K (green, bottom left) or FITC stained thymic exosomes treated with RNase (green, bottom right). (**e**) Representative cytometry plots of SP CD4^+^ thymocytes (top) or CD11c^+^ thymic DCs (bottom) at indicated time points after addition of FITC stained 100 nm latex beads. (**f**) Amounts of cell associated thymic exosomes, normalized to untreated CD11c^+^ DCs or SP CD4^+^ thymocytes, 240 min after addition to RNase or Proteinase K treated SP CD4^+^ thymocytes and CD11c^+^ DCs. FITC-dye centrifuged in the same way as FITC stained exosomes was added to SP CD4^+^ thymocytes or tymic DCs, without generating any detectable staining of the cells.

Multiple potential target cells for thymic exosomes exist in the thymus, for example DCs, thymocytes, TECs and macrophages. Since thymic DCs and CD4^+^ thymocytes are the cell types we used in our *in vitro* studies of exosome effects we next focused on studying exosome uptake by these cells. For this we performed ImageStream analyses, which enable simultaneous imaging and quantification of exosome uptake. Internalization of FITC labeled thymic exosomes by DCs and CD4^+^ thymocytes was seen already after 15 minutes. After 240 minutes 34% of the CD4^+^ thymocytes and 49% of the DCs showed co-localization with exosomes (Fig. 2a,b). The uptake of exosomes by DCs increased during the whole observation period, wheras the thymocyte-associated exosomes increased during the initial 60 minutes and plateaued thereafter (Fig. 2c).

As a reference particle the uptake of 100 nm FITC stained latex beads was studied. Thymocytes stained positive for the 100 nm latex beads after 240 minutes incubation, however, in contrast to the exosomes the beads were mainly localized at the cell surface and not internalized into the intracellular space (Fig. 2d,e).

It has previously been shown that Proteinase-K pre-treatment of tumor derived exosomes reduce their uptake by cells^33^,^34^, and we next aimed to study whether this was true also for the uptake of thymic exosomes into thymic DCs and CD4^+^ thymocytes. Internalization of thymic exosomes by thymic CD4^+^ thymocytes was completely inhibited by pre-treatment of thymic exosomes with Proteinase-K, whereas no reduction was seen for thymic DCs. Furthermore, incubation with saponin and RNase did not reduce the association of thymic exosomes with thymic DCs and CD4^+^ thymocytes (Fig. 2d,f).

### Thymic exosomes induce the maturation of thymocytes *in vitro*

**Figure 3:**
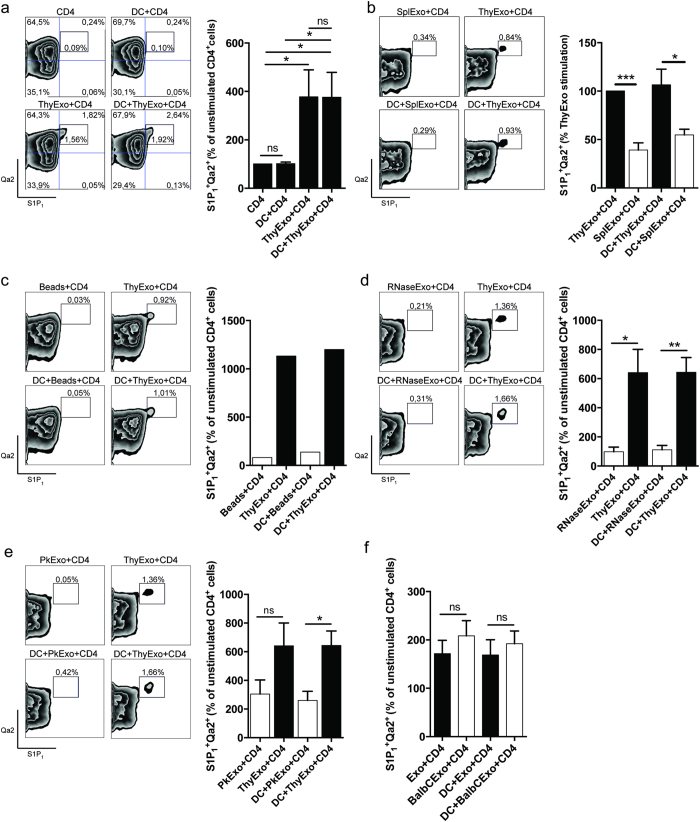
Thymic exosomes induce maturation of thymocytes. After three days in culture with thymic CD11c^+^ DCs and/or thymic exosomes (**a**), spleen exosomes (**b**), 100 nm latex beads (**c**), RNase treated thymic exosomes (**d**) or Proteinase K treated thymic exosomes (**e**); thymocytes were stained for surface expression of CD4^+^S1P_1_^+^Qa2^+^. After three days in culture with C57BL/6 or Balb/c thymic CD11c^+^ DCs, or C57BL/6 or Balb/c thymic exosomes, the C57BL/6 thymocytes were stained for surface expression of CD4^+^S1P_1_^+^Qa2^+^ (**f**). Left figure shows representative flow cytometry plots with indicated stimulations and surface markers. Right figure shows normalized flow cytometry data from at least three individual experiments. Data from exosome experiments are representative from at least three to five individual experiments.

**Figure 4:**
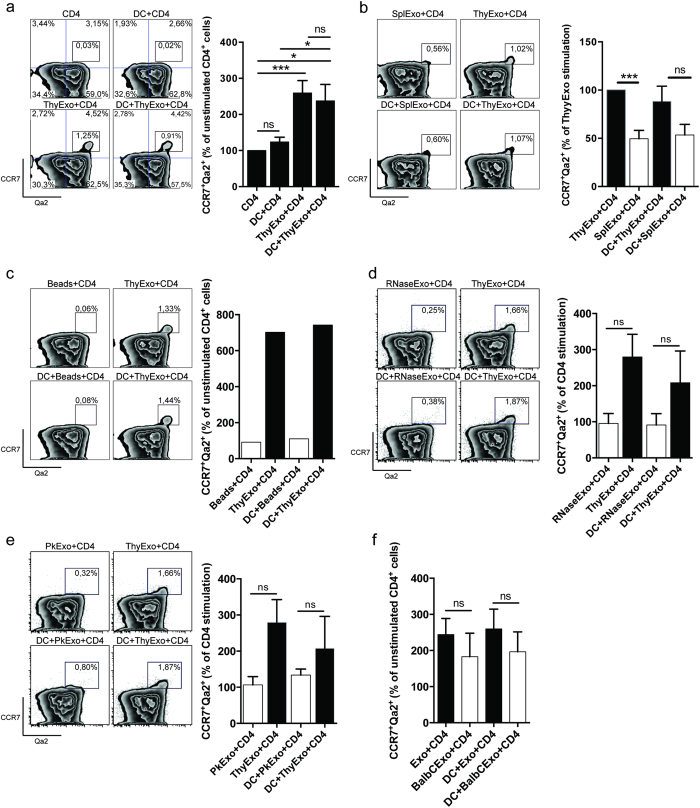
Thymic exosomes induce expression of the thymocyte maturation markers CCR7 and Qa2. After three days in culture with thymic CD11c^+^ DCs and/or thymic exosomes (**a**), spleen exosomes (**b**), 100 nm latex beads (**c**), RNase treated thymic exosomes (**d**) or Proteinase K treated thymic exosomes (**e**); thymocytes were stained for surface expression of CD4^+^CCR7^+^Qa2^+^. After three days in culture with C57BL/6 or Balb/c thymic CD11c^+^ DCs, or C57BL/6 or Balb/c thymic exosomes, the C57BL/6 thymocytes were stained for surface expression of CD4^+^CCR7^+^Qa2^+^ (**f**). Left figure shows representative flow cytometry plots with indicated stimulations and surface markers. Right figure shows normalized flow cytometry data from at least three individual experiments. Data from the exosome experiments are representative from at least three to five individual experiments.

In order to assess the influence of exosomes and DCs on thymocyte maturation, SP CD4^+^CD25^−^ thymocytes were isolated and cultured together with thymic exosomes and/or thymic DC for three days. The expression of S1P_1_, Qa2 and CCR7 was used to evaluate thymocyte maturation. The effective exosome concentration used in this study was based on dose response experiments (Fig. S2) and earlier studies^15^. Thymic exosomes, independently of DCs, significantly increased the number of CD4^+^S1P_1_^+^Qa2^+^ thymocytes, which suggests that thymic exosomes are important for the maturation of SP CD4^+^ thymocytes (Fig. 3a). Furthermore, splenic exosomes could not induce S1P_1_^+^Qa2^+^ expression as potently as thymic exosomes, indicating a superior efficacy of thymic exosomes in the thymocyte maturation process (Fig. 3b). The exosome effects on maturation observed for the CD4^+^S1P_1_^+^Qa2^+^ population was confirmed when we used the CD4^+^CCR7^+^Qa2^+^ phenotype as an endpoint. Thymic exosomes increased the population of CD4^+^CCR7^+^Qa2^+^ thymocytes, independently of the presence of DCs (Fig. 4a). This effect was less potent when stimulating with an equivalent dose of splenic exosomes (Fig. 4b). To test if any type of nano-sized particle could induce CD4^+^CD25^−^ thymocyte maturation, 100 nm latex beads were added to the cultures. However, latex beads had no effect on thymocyte maturation (Figs 3c and.4c).

### RNase and Proteinase K pre-treatment of thymic exosomes reduce their maturation inducing capacity

In order to investigate possible mechanisms underlying the observed maturation inducing capacity of thymic exosomes we tested if the effects depend on surface protein expression and/or transfer of RNA. CD4^+^CD25^−^ thymocytes were isolated and co-cultured together with or without thymic CD11c^+^ DCs, and thymic exosomes pretreated with either Proteinase K or RNase^13^,^35^,^36^. The expression of S1P_1_, Qa2 and CCR7 was used to evaluate the thymocyte maturation.

RNase pretreatment of thymic exosomes efficiently reduced the exosomal RNA content and blocked their maturation inducing effect on CD4^+^CD25^−^ thymocytes, as judged by reduced induction of CD4^+^S1P_1_^+^Qa2^+^ (Fig. 3d and S3) as well as CD4^+^CCR7^+^Qa2^+^ cells (Fig. 4d and S3). Similarly, Proteinase K pretreatment of thymic exosomes efficiently prevented their uptake by target cells, reduced surface protein expression of MFG-E8 and CD9 and blocked the induction of CD4^+^S1P_1_^+^Qa2^+^ (Fig. 3e and S4) and CD4^+^CCR7^+^Qa2^+^ (Fig. 4e and S4) expression on the thymocytes. Interestingly, Proteinase K treatment of exosomes did not lower the MHC class II expression on exosomes. The relative resistance of MHCII to proteinase K treatment was confirmed in additional experiments on B cells (Fig. S4). Zetaview analysis confirmed that the size and numbers of exosomes was not affected by treatment with Proteinase K or Saponin and RNase (Fig. S4).

Collectively, these data suggests that the maturation inducing effects of thymic exosomes are dependent both on exosomal surface proteins and the intraexosomal RNA.

### The effects of thymic exosomes on thymocyte maturation is not MHC restricted

In cross-strain experiments we incubated C57BL/6 CD4^+^CD25^−^ thymocytes and DCs with thymic exosomes from either C57BL/6 or Balb/c mice to study if the exosome effects were depending on MHC compatibility. Regardless of whether exosomes were of C57BL/6 (H2^b^) or Balb/c (H2^d^) origin, the effects on thymocyte maturation, as judged by their expression of CD4^+^S1P_1_^+^Qa2^+^ (Fig. 3f) and CD4^+^CCR7^+^Qa2^+^ (Fig. 4f) did not differ, which shows that the observed effects are not MHC dependent. This result was confirmed by the observation that pre-incubation of the exosomes with blocking antibodies against MHC class II did not alter the exosome effects (data not shown), and supported by the abrogated maturation effect as a result of Proteinase K treatment which left MHCII molecules intact on the exosomes (Figs 3e and 4e).

### Exosomes impair CD4^+^CD25^+^FoxP3^+^ development

**Figure 5:**
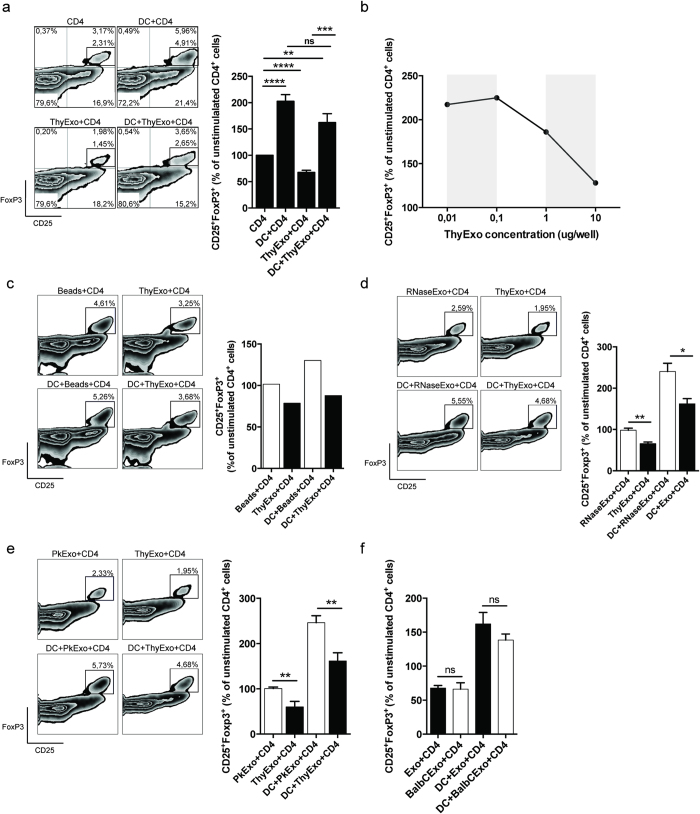
Thymic exosomes suppress formation of CD4^+^CD25^+^FoxP3^+^ thymocytes. After three days in culture with thymic CD11c^+^ DCs and/or thymic exosomes, the thymocytes were stained for surface expression of CD4^**+**^CD25^+^FoxP3^+^. (**a**) Representative flow cytometry plot with indicated stimulations (left) and normalized flow cytometry data from 6 individual experiments with the indicated stimulations (right). Data is displayed as means ± SEM. (**b**) Representative diagram of CD4^+^CD25^−^ thymocytes cultured for three days with CD11c^+^ DCs and indicated concentrations of thymic exosomes followed by staining for surface expression of CD4^+^CD25^+^FoxP3^+^. Data is representative for two individual experiments. (**c**) After three days in culture with thymic CD11c^+^ DCs and/or 100 nm latex beads thymocytes were stained for surface expression of CD4^**+**^CD25^+^FoxP3^+^. After three days in culture with thymic CD11c^+^ DCs and/or RNase (**d**) or Proteinase K (**e**) treated thymic exosomes, the thymocytes were stained for surface expression of CD4^**+**^CD25^+^FoxP3^+^. Data is representative of three individual experiments. After three days in culture with C57BL/6 or Balb/c thymic CD11c^+^ DCs, or C57BL/6 or Balb/c thymic exosomes, the C57BL/6 thymocytes were stained for surface expression of CD4^+^CD25^+^FoxP3^+^ (**f**). Data is pooled from two independent experiments and displayed as means ± SEM.

To investigate the relative effect of thymic exosomes and DCs on Treg (CD4^+^CD25^+^FoxP3^+^) development, CD4^+^CD25^−^ thymocytes were isolated and cultured together with thymic DCs and/or thymic exosomes for three days. Thymic DCs alone, without the presence of thymic exosomes, stimulated the formation of CD4^+^CD25^+^FoxP3^+^ cells. Interestingly, addition of thymic exosomes resulted in reduced proportions of CD4^+^ CD25^+^ FoxP3^+^ thymocytes, both in the presence and absence of DCs (Fig. 5a). This was confirmed in dose-response experiments where increased concentrations of thymic exosomes resulted in reduced proportions of CD4^+^FoxP3^+^ thymocytes (Fig. 5b). No reduction of CD4^+^CD25^+^FoxP3^+^ thymocytes was detected when thymocytes were cultured with 100 nm latex beads, which indicates that the mere presence of nano sized patricles was not responsible for this effect (Fig. 5c).

Treatment of exosomes with RNase (Fig. 5d) or Proteinase K (Fig. 5e) reduced their inhibitory effect on CD4^+^CD25^+^FoxP3^+^ cells, both in presence and absence of thymic DCs, indicating that the observed exosome effects are RNA and surface protein dependent. Furthermore, the inhibitory effect of thymic exosomes on CD4^+^CD25^+^FoxP3^+^ thymocytes was not dependent on MHCII compatibility (Fig. 5f).

Taken together, these data suggests that induction of thymic CD4^+^FoxP3^+^ cells is dependent on thymic DCs whereas thymic exosomes seems to hamper the formation of CD4^+^CD25^+^FoxP3^+^ cells via an exosomal surface protein- and RNA-dependent mechanism.

## Discussion

We have previously reported that human thymic exosomes display a protein content including TRAs, miRNA and surface markers which together indicate a thymic epithelial cell origin^16^. In the current study, we identify exosomes as key regulators of late stage thymocyte maturation *in vitro*. The basic characterization of the murine thymic exosomes regarding size and surface markers confirmed their identity as exosomes and showed that they share the previously described phenotype of human thymic exosomes^16^. Furthermore, proteomic analysis of thymic exosomes revealed that 458 (29.9%) of the identified proteins are normally expressed in peripheral tissues, whereas proteins restricted to thymocytes, DCs, B cells and macrophages were only present in minute numbers. In addition, we could also identify many TEC associated proteins and TRAs. Taken togehther, these data supports that a large proportion of thymic exosomes originates from TECs.

The mechanisms behind late stage thymocyte maturation are complex. Here we identify thymic exosomes as potent stimulators of the final maturation of thymocytes and that they suppress, at least in quantitative terms, the Treg development. When we studied the impact of thymic exosomes and DCs on SP thymocyte maturation *in vitro* we combined the markers CCR7, S1P_1_ and Qa2, and used the two different combinations; CD4^+^CCR7^+^Qa2^+^ and CD4^+^S1P_1_^+^Qa2^+^, to define finally mature SP thymocytes. We observed a significant increase in the proportion of mature thymocytes in the presence of exosomes, using any of the two surface marker combinations (CD4^+^S1P_1_^+^Qa2^+^ or CD4^+^CCR7^+^Qa2^+^). As shown in the present study thymic exosomes carry proteins involved in maturation and egress of developing thymocytes, including SGPL1, PAK2, DOCK2 and GDIR1^22^,^23^,^29^,^30^,^32^.

Since exosomes are nanosized particles we used 100 nm latex beads as a reference particle for the presence of nanosized particles in the cultures, however the similarly sized latex beads had no effect on thymocyte maturation.

We observe that the exosomal effects were independent of the presence or absence of DCs in the cultures, which indicates a direct interaction between exosomes and thymocytes rather than an indirect effect via the DCs. Interactions between exosomes and target cells may depend on internalization, interaction with membrane bound structures or fusing with the target cell membrane^11^. The efficient internalization of thymic exosomes by both DCs and thymocytes seen in the ImageStream analyses indicate that internalization of exosomes by the target cells is a main route of interaction (Fig. 2).

The exosome effect on thymocyte maturation was shown to be dependent on an intact surface proteome since treatment of the exosomes with Proteinase K effectively inhibited the induction of thymocyte maturation. Furthermore, also RNAse treatment of the exosomes inhibited maturation, which indicates that the RNA content of the exosomes mediates the effects on the thymocytes. In conclusion, both exosomal surface proteins and RNA content are important for their maturation inducing effect.

Spleen exosomes could only induce 40–50% of CD4^+^S1P_1_^+^Qa2^+^ and CD4^+^CCR7^+^Qa2^+^ thymocytes as compared to exosomes of thymic origin (Figs 3b and 4b). The miRNA profile of thymic exosomes reveals that a significant number of miRNAs are exclusively found in thymic exosomes and not in exosomes isolated from the spleen. This may partly explain why the splenic derived exosomes were inferior in causing maturation of the thymocytes and the differentially expressed miRNAs are potential mediators of the effects of thymic exosomes.

We were not able to confirm the reported TGFβ-dependent stimulatory effects on nTreg by thymic exosome like particles^15^. On the contrary, in our system a significant reduction of the formation of cells with a CD4^+^CD25^+^FoxP3^+^ phenotype was seen when adding thymic exosomes to cultures of CD4^+^CD25^−^ thymocytes. This effect is dose-dependent, and is not a result of the mere presence of nanosized particles since adding 100 nm latex beads in the culture did not affect the thymocytes. The exosome effect on the Treg development is seen both in the presence and absence of DCs. Similar to the exosome effects on thymocyte maturation, the DC independency suggests that direct effects of exosomes underlie the inhibition of the DC independent CD4^+^CD25^+^FoxP3^+^ cell formation. This notion is supported by the efficient uptake of exosomes by CD4^+^ thymocytes demonstrated in the present study. However, hampering of the DC induced CD4^+^CD25^+^FoxP3^+^ cell formation could still be due to effects of exosomes on the DCs themself.

Treatment of thymic exosomes with RNase or Proteinase K inhibited the effect of thymic exosomes on CD4^+^CD25^+^FoxP3^+^ formation, suggesting that both exosomal RNA and surface proteins are involved in the reduction of CD4^+^CD25^+^FoxP3^+^ cell formation *in vitro*.

A hallmark of thymic exosomes is a strong surface expression of MHCII. However, the the C57BL/6-H2b - Balb/c-H2d cross-strain experiments suggested that both the stimulatory effect of exosomes on thymocyte maturation and the inhibitory effect on Treg formation are independent of MHC compatibility between the exosome producing cells and the target cells. These results argue against involvement of the TCR- MHC interaction in this process. Indeed, it has been shown that exosomes from DCs have immunoregulatory effects on T cells independent of MHC compatibility^37^. Our findings are also congruent with observations by Li *et al*. that TECs are potent inducers of final maturation of SP thymocytes in an MHC independent manner^38^. However this does not exclude the possibility that exosomes are important in the presentation of specific MHC-peptide complexes, either directly or via thymic DCs, to individual thymocytes in the thymocyte selection process, and the presence of a strong MHC class II expression on exosomes favors the hypothesis that thymic exosomes may be involved in the transfer of MHC-peptide complexes or in direct presentation of antigens to developing thymocytes.

In conclusion, our data show that thymic exosomes facilitate maturation of thymocytes but suppress both DC dependent and DC independent Treg formation. Both these effects rely on the presence of exosomal RNA and exosomal surface proteins, but do not require MHC compatibility.

## Methods

### Mice

C57BL/6 and Balb/c mice (The Jackson Laboratory, Bar Harbor, ME) were kept under pathogen free conditions, sacrificed at three weeks of age and thymus tissue was retrieved. The work was surveyed and approved by the Gothenburg University ethics committee (ethics approval no: 2012–158). The methods were carried out in accordance with the approved guidelines.

### Isolation and characterization of thymic exosomes

Thymic tissues were collected in cold PBS and pressed against a 40 μm filter. The resulting cell suspension was centrifuged for 5 min at 400 × g to remove cells and the supernatant was used for isolation of exosomes as previously described^16^. Protein concentration of the isolated exosomes was determined by the Bradford protein assay (Bio-Rad, Hercules, CA) according to the manufacturer’s instruction. A typical exosomal yield from eight thymuses was 30 μg.

The size of the exosomes was estimated by the Brownian motion of the particles in a ZetaView instrument (Fig. S1). Flow cytometry analyses of exosomes were performed after they had been adsorbed on 4 μm latex beads (Invitrogen) as previously described (24). Monoclonal antibodies used for chacterisation of exosomes are displayed in supplementary table 1 (Table S1).

Isolated exosomes equivalent to 50 μg of protein was used for tandem mass spectrometry as previously described^16^.

### Proteomic analysis of thymic exosomes

Total protein from thymic exosomes were isolated and identified by tandem masspectometry, as previously described^16^. Entrez IDs for each of the 1556 proteins were obtained using DAVID (https://david.ncifcrf.gob/). Since several of the proteins had multiple measurements from different probe sets, probe sets with a low maximum value was filtered out, whereas probe sets with the highest maximum value was used for analysis. The remaining 1531 proteins were grouped into 62 different categories based on the BioGPS database (http://ds.biogps.org/). Since values of different proteins generally come from different probe sets, expression data were normalized by protein. Filtering and construction of the heat map was done using the statistical software R (R Foundation for Statistical Computing, Vienna, Austria. URL: https://www.R-projects.org/). Based on the BioGPS database the 62 categories were further grouped into 5 different cellular sources; peripheral tissues, T cells, B cells, macrophages and DCs. A protein was defined as expressed in a category if its expression value was above 3 times the median expression of that protein. The Venn diagram was created using R package gplots (https://www.CRAN.R-project.org/package=gplots).

### miRNA analysis by 3D-gene microarray

Thymus and spleen exosomes from 8 female C57BL/6 mice were isolated as previously described and divided in 2 + 2 samples. Samples were normalized to 113.6 ng/μl and 2.2 μl of the normalized sample (total amount 250 ng) was mixed with miRNA spike (Cat No. TRT-XR304, Toray, Tokyo, Japan) and further labelled, hybridized and washed according to the instruction manual from Toray (H-M-R miRNA protocol 4-Plex V3) using Toray miRNA Labelling kit (Cat No TRT-XE211) and Mouse miRNA Oligo chip 4plex, based on miRBase 21 (Cat No TRT-XR530, Toray). The intensity of each miRNA was analyzed with the 3D-Gene Scanner 3000 (Toray) with auto gain, auto focus and auto analysis settings, all according to manufacturer’s instructions. Quality control was performed on all four samples based on the QC report from the instrument.

### Isolation of single positive SP CD4^+^ thymocytes

Freshly isolated thymic tissues were cleaned from fat and connective tissue and placed into cold PBS. The thymic tissues were passed through a 40 μm filter, and the cell suspensions were collected and incubated for 15 minutes with rotation in FACS buffer+DNAse (1%). Cells were then again passed through a 40 μm filter and incubated with FACS buffer+DNAse for 15 minutes. Untouched single positive (SP) CD4^+^CD25^−^ cells were isolated by negative selection using Dynabeads FlowComp Mouse CD4^+^CD25^−^ Treg Cells Kit (Life Technologies, Carlsbad, CA), according to the manufacturers instructions. The purity of SP CD4^+^CD25^−^ cells was 90%, as determined by flow cytometry.

### Isolation of CDllc^+^ DCs

Thymic tissues were cleaned from fat and connective tissue and cut into small pieces. Pre-warmed enzymes, 1ml/thymus (Liberase 0.5 U/ml, DNAse 0.2 mg/ml), were added to the tissue and incubated for 20 minutes, in 37 °C, under rotation followed by gentle flushing of the pieces. The supernatant was removed and placed in cold FACS buffer (2% exosome-free FBS, 2 mM EDTA) on ice. This was repeated three times, to assure complete tissue dissociation. The supernatants were pooled, washed and Fc-blocked for 15 min. CDllc^+^ cells were enriched to a purity of 70% using CDllc microbeads (Miltenyi Biotec, Bergisch Gladbach, Germany). Futhermore, CDllc^+^ cells were characterized as 45% CDllc+CD8α^+^ Sirpα^low^ and 33%CDllc+CD8α^low^ Sirpα^+^, and 20% of the CDllc^+^ cells were identified as plasmacytoid DCs cells (B220^+^ BDA1^+^) (data not shown).

### ImageStream uptake analysis

Exosomes were purified as described above and stained with FITC 0.05 ug/ml (Sigma-Aldrich, cat no. F3651). As a reference particle, 100 nm latex beads were stained with FITC. Unbound FITC was removed by washing step and ultracentrifugation at 100 000 × g for 70 min. CD4^+^CD25^−^ thymocytes and CD11c^+^ DCs were enriched as described. Purified cells were stained with antibodies for flow cytometry: CD4 PeCy7 (BD Bioscience cat no. 552775) and CDllc biotin (eBioscience cat no 13-0114-82) followed by APC-Streptavidin (BD Pharming cat no 554067). Cells were cultured in 37 °C and FITC stained exosomes or 100 nm latex beads were added to cultures at 15, 30, 60 and 240 minutes. Ultracentrifuged FITC dye without exosomes was used as background control. CDllc^+^ DC or SP CD4^+^ thymocytes incubated with exosomes, beads or FITC dye without exosomes were fixated with 1% paraformaldehyd and analyzed on an ImageStream X Mark II imaging flow cytometer (Amnis, Seattle, WA).

### Cell culture

100,000 sorted SP CD4^+^CD25^−^ thymocytes were co-cultured in 96-well round bottom plates together with 5000 thymic DCs and 10 μg of thymic exosomes (final concentration 20 × 10^9^ exosomes/ml) in 200 μl medium for 3 days. In addition, CD4^+^CD25^−^ thymocytes were cultured alone or with either exosomes or DCs. The exosome concentration used was based on dose-response experiments and earlier studies^15^. To study if the exosome effects were MHC restricted, cells and exosomes from different mouse strains (C57BL/6 and Balb/c) were used in cross-strain experiments. 100 nm latex beads (Sigma-Aldrich, St- Louis, MO) were added to a final concentration of 75 × 10^9^ beads/ml to separate cultures as described for the exosomes and used as a reference for nano particle presence. The cell medium consisted of RPMI 1640 (Invitrogen, Paisley, Scotland), with 5% exosome-depleted FBS (Sigma-Aldrich, St- Louis, MO), 2 mM L-glutamine (Life Technologies), 100U penicillin/ 100 μg streptomycin (Sigma-Aldrich) and 50 μM 2-mercapthoethanol (Gibco (Life Technologies). To support thymocyte survival, IL-7 (Peprotech, Rocky Hill, NJ), 0.2 ng/ml and IL-2 (RnDSystems), 20 ng/ml, were added to the cultures.

### Flow cytometry of cells

Cell surface stainings were performed using the monoclonal antibodies displayed in supplementary Table 1 (Table S1). Flow cytometry analysis was performed on a FacsCantoII (BD Bioscience) and analyzed using FlowJo software (Tree Star, Ashland, OR).

### Protein cleavage and RNAse treatment of exosomes

Exosomes were isolated as previously described and incubated with Proteinase K 100 μg/ml in 37 °C for 30 min and then washed by ultracentrifugation at 100 000 × g for 70 minutes. Proteinase K treated exosomes were stained for thymic exosome surface proteins and analyzed by the flow cytometer to evaluate the degradation of surface proteins. The size of the exosomes was estimated by the Brownian motion of the particles in a ZetaView instrument (Particlemetrix, Germany).

Exosomes were incubated with RNase in 37 °C for 30 minutes to degrade exosomal RNA, 0.2% Saponin was added to the solution to make the membrane permeable. Exosomes were washed by ultracentrifugation, 100 000 × g for 70 minutes. RNase and Saponin concentrations were titrated for the most effective deletion of RNA with the lowest possible concentration of Saponin (data not shown). RNA quality of the treated and untreated thymic exosomes was analyzed with a Fragment Analyzer at TATAA Biocenter Gothenburg (Fig. S3).

### Statistical analysis

Values are presented as mean ± standard error of the mean (SEM). Data were statistically evaluated using Student’s T-test, in the Prism 6 software (GraphPad, La Jolla, CA). Values of *P* ≤ 0.05 were considered as statistically significant.

## Additional Information

**How to cite this article**: Lundberg, V. *et al*. Thymic exosomes promote the final maturation of thymocytes. *Sci. Rep.* **6**, 36479; doi: 10.1038/srep36479 (2016).

**Publisher’s note:** Springer Nature remains neutral with regard to jurisdictional claims in published maps and institutional affiliations.

## Supplementary Material

Supplementary Information

## References

[b1] Klein L., Kyewski B., Allen P. M. & Hogquist K. A. Positive and negative selection of the T cell repertoire: what thymocytes see (and don’t see). Nature reviews. Immunology, doi: 10.1038/nri3667 (2014). PMC4757912 24830344

[b2] Anderson G. & Jenkinson E. J. Lymphostromal interactions in thymic development and function. Nature reviews. Immunology 1, 31–40, doi: 10.1038/35095500 (2001). 11905812

[b3] Derbinski J. . Promiscuous gene expression in thymic epithelial cells is regulated at multiple levels. J Exp Med. 202, 33–45, doi: 10.1018/jem.20050471 (2005). 15983066 PMC2212909

[b4] Sansom S. N. . Population and single-cell genomics reveal the Aire dependency, relief from Polycomb silencing, and distribution of self-antigen expression in thymic epithelia. Genome research, doi: 10.1101/gr.171645.113 (2014). PMC4248310 25224068

[b5] Anderson M. S. . Projection of an immunological self shadow within the thymus by the aire protein. Science 298, 1395–1401, doi: 10.1126/science.1075958 (2002). 12376594

[b6] Takaba H. . Fezf2 Orchestrates a Thymic Program of Self-Antigen Expression for Immune Tolerance. Cell 163, 975–987, doi: 10.1016/j.cell.2015.10.013 (2015). 26544942

[b7] Hubert F. X. . Aire regulates the transfer of antigen from mTECs to dendritic cells for induction of thymic tolerance. Blood 118, 2462–2472, doi: 10.1182/blood-2010-06-286393 (2011). 21505196

[b8] Ucar O. & Rattay K. Promiscuous Gene Expression in the Thymus: A Matter of Epigenetics, miRNA, and More? Frontiers in immunology 6, 93, doi: 10.3389/fimmu.2015.00093 (2015). 25784915 PMC4347492

[b9] Koble C. & Kyewski B. The thymic medulla: a unique microenvironment for intercellular self-antigen transfer. The Journal of experimental medicine 206, 1505–1513, doi: 10.1084/jem.20082449 (2009). 19564355 PMC2715082

[b10] Skogberg G., Telemo E. & Ekwall O. Exosomes in the Thymus: Antigen Transfer and Vesicles. Frontiers in immunology 6, 366, doi: 10.3389/fimmu.2015.00366 (2015). 26257734 PMC4507453

[b11] Robbins P. D. & Morelli A. E. Regulation of immune responses by extracellular vesicles. Nature reviews. Immunology 14, 195–208, doi: 10.1038/nri3622 (2014). PMC4350779 24566916

[b12] Raposo G. . B lymphocytes secrete antigen-presenting vesicles. The Journal of experimental medicine 183, 1161–1172 (1996). 8642258 10.1084/jem.183.3.1161 PMC2192324

[b13] Valadi H. . Exosome-mediated transfer of mRNAs and microRNAs is a novel mechanism of genetic exchange between cells. Nature cell biology 9, 654–659, doi: 10.1038/ncb1596 (2007). 17486113

[b14] Ostman S., Taube M. & Telemo E. Tolerosome-induced oral tolerance is MHC dependent. Immunology 116, 464–476, doi: 10.1111/j.1365-2567.2005.02245.x (2005). 16313360 PMC1802439

[b15] Wang G. J. . Thymus exosomes-like particles induce regulatory T cells. Journal of immunology 181, 5242–5248 (2008). 10.4049/jimmunol.181.8.5242 PMC4319673 18832678

[b16] Skogberg G. . Characterization of human thymic exosomes. PloS one 8, e67554, doi: 10.1371/journal.pone.0067554 (2013). 23844026 PMC3699640

[b17] Ueno T. . CCR7 signals are essential for cortex-medulla migration of developing thymocytes. The Journal of experimental medicine 200, 493–505, doi: 10.1084/jem.20040643 (2004). 15302902 PMC2211934

[b18] Kwan J. & Killeen N. CCR7 directs the migration of thymocytes into the thymic medulla. Journal of immunology 172, 3999–4007 (2004). 10.4049/jimmunol.172.7.3999 15034011

[b19] Davalos-Misslitz A. C., Worbs T., Willenzon S., Bernhardt G. & Forster R. Impaired responsiveness to T-cell receptor stimulation and defective negative selection of thymocytes in CCR7-deficient mice. Blood 110, 4351–4359, doi: 10.1182/blood-2007-01-070284 (2007). 17785582

[b20] Carlson C. M. . Kruppel-like factor 2 regulates thymocyte and T-cell migration. Nature 442, 299–302, doi: 10.1038/nature04882 (2006). 16855590

[b21] Camerini D., James S. P., Stamenkovic I. & Seed B. Leu-8/TQ1 is the human equivalent of the Mel-14 lymph node homing receptor. Nature 342, 78–82, doi: 10.1038/342078a0 (1989). 2509939

[b22] Zachariah M. A. & Cyster J. G. Neural crest-derived pericytes promote egress of mature thymocytes at the corticomedullary junction. Science 328, 1129–1135, doi: 10.1126/science.1188222 (2010). 20413455 PMC3107339

[b23] Pappu R. . Promotion of lymphocyte egress into blood and lymph by distinct sources of sphingosine-1-phosphate. Science 316, 295–298, doi: 10.1126/science.1139221 (2007). 17363629

[b24] Schwab S. R. & Cyster J. G. Finding a way out: lymphocyte egress from lymphoid organs. Nature immunology 8, 1295–1301, doi: 10.1038/ni1545 (2007). 18026082

[b25] Matloubian M. . Lymphocyte egress from thymus and peripheral lymphoid organs is dependent on S1P receptor 1. Nature 427, 355–360, doi: 10.1038/nature02284 (2004). 14737169

[b26] Teng F. . The molecular signature underlying the thymic migration and maturation of TCRalphabeta+ CD4+ CD8 thymocytes. PloS one 6, e25567, doi: 10.1371/journal.pone.0025567 (2011). 22022412 PMC3192722

[b27] McCaughtry T. M., Wilken M. S. & Hogquist K. A. Thymic emigration revisited. The Journal of experimental medicine 204, 2513–2520, doi: 10.1084/jem.20070601 (2007). 17908937 PMC2118501

[b28] Mathivanan S., Ji H. & Simpson R. J. Exosomes: extracellular organelles important in intercellular communication. Journal of proteomics 73, 1907–1920, doi: 10.1016/j.jprot.2010.06.006 (2010). 20601276

[b29] Maeda Y. . S1P lyase in thymic perivascular spaces promotes egress of mature thymocytes via up-regulation of S1P receptor 1. International immunology 26, 245–255, doi: 10.1093/intimm/dxt069 (2014). 24343820

[b30] Phee H. . Pak2 is required for actin cytoskeleton remodeling, TCR signaling, and normal thymocyte development and maturation. eLife 3, e02270, doi: 10.7554/eLife.02270 (2014). 24843022 PMC4017645

[b31] Ishizaki H. . Defective chemokine-directed lymphocyte migration and development in the absence of Rho guanosine diphosphate-dissociation inhibitors alpha and beta. Journal of immunology 177, 8512–8521 (2006). 10.4049/jimmunol.177.12.8512 17142749

[b32] Nombela-Arrieta C. . A central role for DOCK2 during interstitial lymphocyte motility and sphingosine-1-phosphate-mediated egress. The Journal of experimental medicine 204, 497–510, doi: 10.1084/jem.20061780 (2007). 17325199 PMC2137902

[b33] Escrevente C., Keller S., Altevogt P. & Costa J. Interaction and uptake of exosomes by ovarian cancer cells. BMC cancer 11, 108, doi: 10.1186/1471-2407-11-108 (2011). 21439085 PMC3072949

[b34] Smyth T. J., Redzic J. S., Graner M. W. & Anchordoquy T. J. Examination of the specificity of tumor cell derived exosomes with tumor cells *in vitro*. Biochimica et biophysica acta 1838, 2954–2965, doi: 10.1016/j.bbamem.2014.07.026 (2014). 25102470 PMC5657189

[b35] Nolte-‘t Hoen E. N., Buschow S. I., Anderton S. M., Stoorvogel W. & Wauben M. H. Activated T cells recruit exosomes secreted by dendritic cells via LFA-1. Blood 113, 1977–1981, doi: 10.1182/blood-2008-08-174094 (2009). 19064723

[b36] Segura E. . ICAM-1 on exosomes from mature dendritic cells is critical for efficient naive T-cell priming. Blood 106, 216–223, doi: 10.1182/blood-2005-01-0220 (2005). 15790784

[b37] Yu L. . Exosomes with membrane-associated TGF-beta1 from gene-modified dendritic cells inhibit murine EAE independently of MHC restriction. European journal of immunology 43, 2461–2472, doi: 10.1002/eji.201243295 (2013). 23716181

[b38] Li J. . Developmental pathway of CD4+CD8- medullary thymocytes during mouse ontogeny and its defect in Aire-/- mice. Proceedings of the National Academy of Sciences of the United States of America 104, 18175–18180, doi: 10.1073/pnas.0708884104 (2007). 17984055 PMC2084316

