# Supplementary material for: Thymic exosomes promote the final maturation of thymocytes
Source: Sci Rep. 2016 Nov 8;6:36479. doi: 10.1038/srep36479 (PMC5099897; doi:10.1038/srep36479)
Supplement: Supplementary Information [file srep36479-s1.pdf]

Thymic exosomes promote the final maturation of thymocytes

Vanja Lundberg[1,2]*, Martin Berglund[1]*, Gabriel Skogberg[1,†], Susanne Lindgren[1,2], Christina Lundqvist[1], Judith Gudmundsdottir [1,2], Karolina Thörn[1], Esbjörn Telemo[1] and Olov Ekwall[1,2].

[1]Dept of Rheumatology and Inflammation Research at the Institute of Medicine and [2]Dept of Pediatrics at the Institute of Clinical Sciences at the Sahlgrenska Academy, University of Gothenburg, Sweden.

Supplementary data

Supplementary figure S1

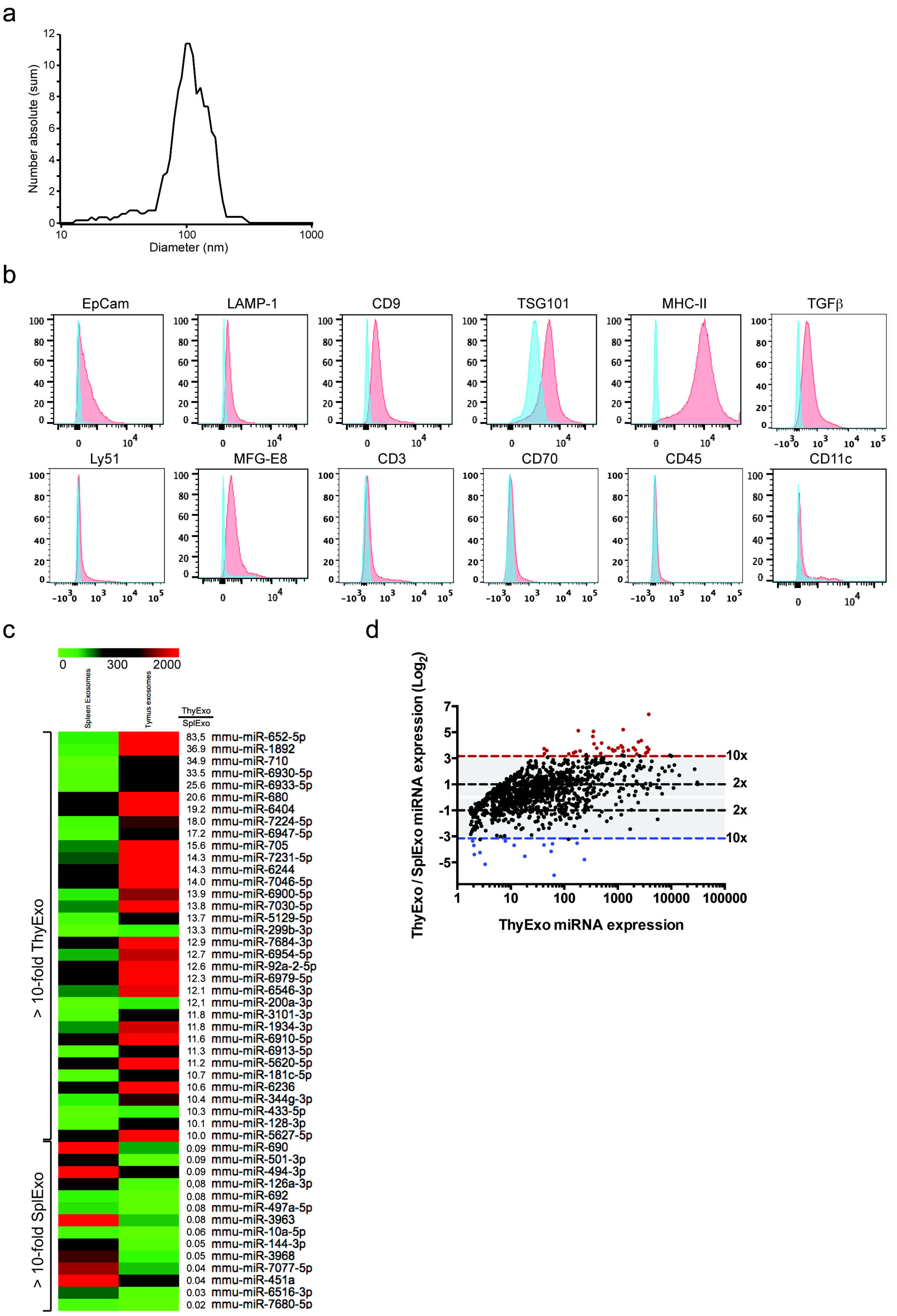

Figure S1. Characteristics of thymic exosomes.

(a) Representative Zetaview analysis of thymic exosomes shows vesicles with a median size of 102 nm in diameter. (b) Flow cytometry of thymic exosomes attached to 4μm latex beads. Thymic exosomes showed positive staining for EpCam, CD9, TSG101, MHC-II, MFG-E8, TGFβ and LAMP-1, and negative/weak staining for Ly51, CD70, CD45, CD3 and CD11c. Red profiles indicate latex beads with thymic exosomes and blue profiles latex beads without exosomes. Data are representative of 3 independent experiments. (c) Thymus and spleen exosomes from 8 female C57BL/6 mice were isolated and analyzed with 3D gene microarray. Heat map based on the mean expression level of miRNAs expressed more than 10 fold in thymus exosomes (top, n=34) and in spleen exosomes (bottom, n=14). miRNAs are ordered according to the thymus vs spleen expression ratio from top to bottom. (d) miRNA differentially expressed between thymus and spleen exosomes. 450 miRNAs were expressed > 2 fold, and 34 miRNAs were expressed more than 10 fold (red) in thymus exosomes, as compared to spleen exosomes.

Supplementary figure S2

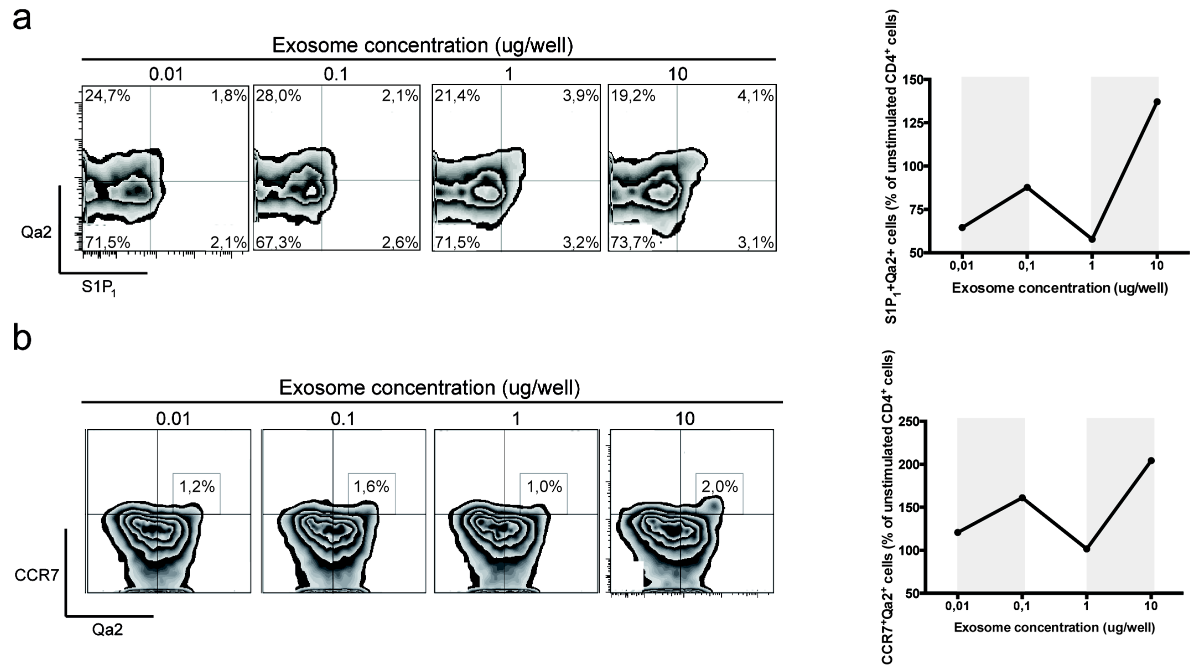

Figure S2. Thymocyte maturation is dependent on thymus exosome concentration

After three days in culture with thymic CD11c^{+} DCs and indicated concentrations of thymic exosomes, thymocytes were stained for surface expression of CD4^{+}S1P$_{1}$$^{+}$Qa2^{+} (a) and CD4^{+}CCR7^{+}Qa2^{+} (b). FACS plots are representative from two individual experiments and shown as % of CD4^{+}CD25^{-} cells.

Supplementary figure S3

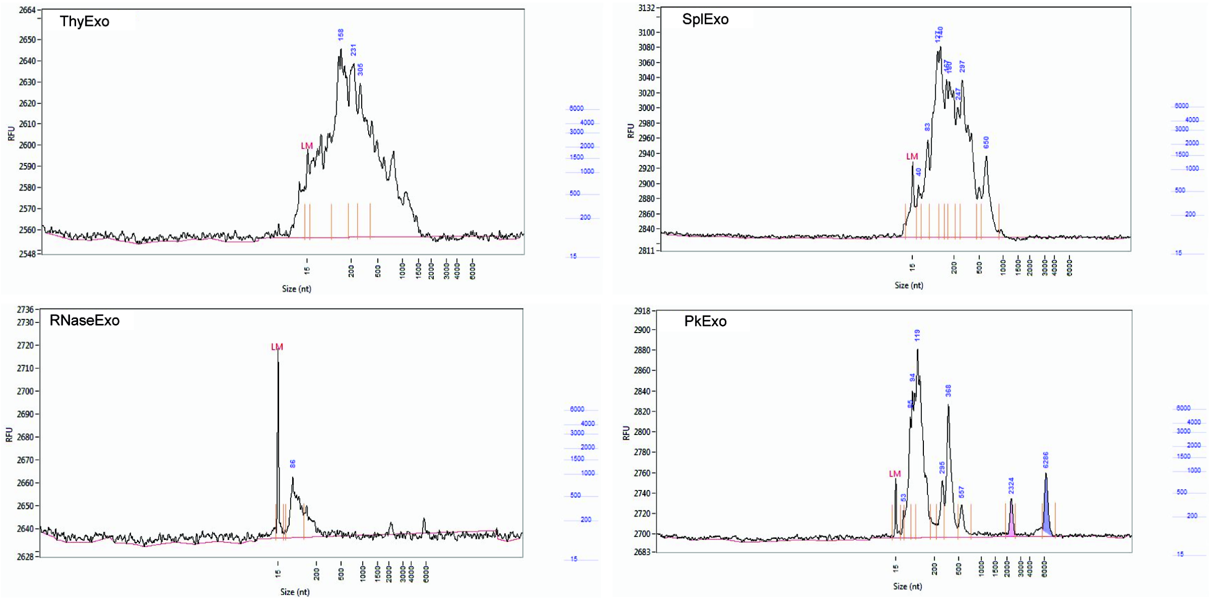

Figure S3. Total RNA analysis of treated and untreated thymic exosomes, or spleen exosomes using a Fragment Analyzer

Thymic and spleen exosomes were isolated as previously described and washed by ultracentrifugation, 100 000xg for 70 min (top row). In addition, thymic exosomes were incubated with RNAse or Proteinase K in 37°C for 30 min followed by ultracentrifugation, 100 000xg for 70 min (bottom row). RNA was then extracted and diluted to 2.5ng/ul and analyzed with Fragment Analyzer. Relative fluorescence units are seen on the y-axis and RNA size distribution (nt) is seen on the x-axis. Lower marker (LM) has a size of 15nt.

Supplementary figure S4

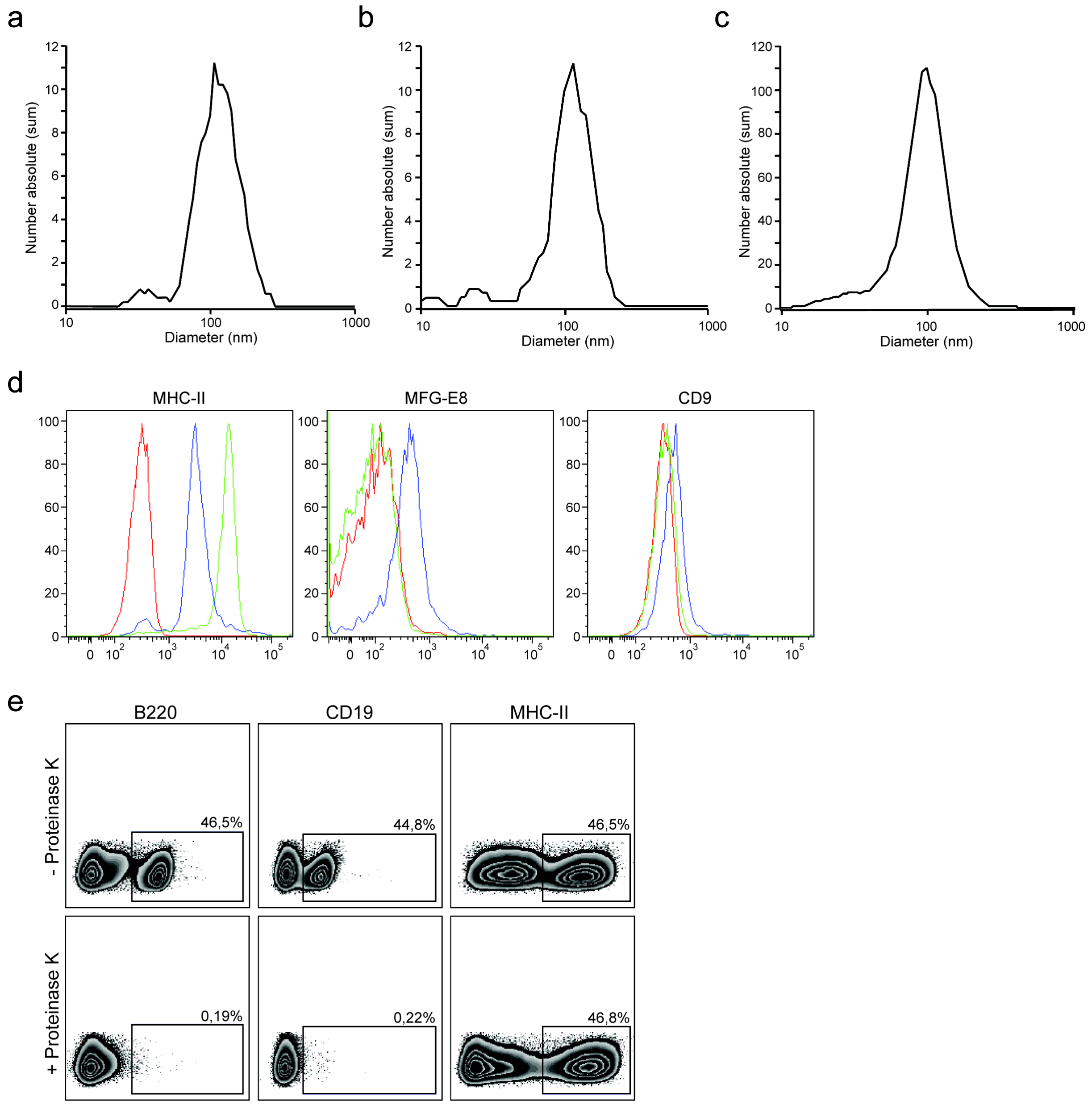

Figure S4. Analysis of exosomes and thymus cells treated with Proteinase K and RNAse

Zetaview analysis of exosomes treated with Proteinase K (a), Saponin and RNAse (b) or 100nm beads used as reference (c). (d) Thymus exosome surface expression of MHC-II, MGF-E8 or CD9 analyzed with FACS after treatment with Proteinase K (green), compared with untreated exosomes (red) or control beads (blue). (e) Splenocyte

expression of B220, CD19 or MHC-II analyzed with FACS after treatment with (bottom) or without (top) Proteinase K.

Supplementary table S1

Monoclonal antibodies used for exosome characterisation and cell surface staining

Primary antibodies	Company	Cat no.
CD3 PE	BD Bioscience, Franklin lakes, NJ	553064
CD9 biotin	BD Bioscience, Franklin lakes, NJ	558749
CD45 FITC	BD Bioscience, Franklin lakes, NJ	553080
CDllc PE	eBioscience, San Diego, CA, USA	12-0114-81
CD70 PE	Biolegend, San Diego, CA	104606
TSG101	Santa Cruz Biotechnology,, Dallas, TX	sc-6037
MFG-E8	RnDSystems, Minneapolis, MN	MAB2805
MHC-II biotin	eBioscience,San Diego, CA, USA	13-5321-82
TGFb biotin	BD Bioscience, Franklin lakes, NJ	555053
EpCam PE	Abcam, Cambridge, UK	ab93545
Ly51 FITC	BD Bioscience, Franklin lakes, NJ	553160
LAMP-1 FITC	BD Bioscience, Franklin lakes, NJ	553793
CD4PECy7	BD Bioscience, Franklin lakes, NJ	552775
CD8 APC	BD Bioscience, Franklin lakes, NJ	553035
CD25 PE	BD Bioscience, Franklin lakes, NJ	
$S1P_1$ APC	RnDSystems, Minneapolis, MN	FAB708A
Qa2 FITC	eBioscience,San Diego, CA, USA	11-5996-81
CCR7 PE	Biolegend, San Diego, CA	120105
CCR7 APC	eBioscience,San Diego, CA, USA	17-1971-81
FoxP3 PE	eBioscience,San Diego, CA, USA	12-4771-82
CD11c PE	eBioscience,San Diego, CA, USA	12-0114-82
CD11c biotin	Biolegend, San Diego, CA	117304
MHC-II APC-eFluor780	eBioscience,San Diego, CA, USA	47-5321-82
B220 PerCP	BD Bioscience, Franklin lakes, NJ	553093
PDA1 PE	eBioscience,San Diego, CA, USA	12-3171-82

Secondary antibodies	Company	Cat no.
Streptavidin PE	BD Bioscience, Franklin lakes, NJ	554061
Sheep anti-rat biotin	AbD Serotech, , Kidlington, UK	AAR10B
Rabbit anti-goat biotin	DAKO, Glostrup, Denmark	E0466

Accessories	Company	Cat no.
FoxP3 buffer set	BD Bioscience, Franklin lakes, NJ	560409
Viability dye eFluor 780	eBioscience,San Diego, CA, USA	65-0865-14

Supplemenatry table S2

Proteomic profile of thymic exosomes

Accession	Description	Score	Coverage	# Proteins	Unique Peptid	# Peptides	# PSMs	# AAs	MW [kDa]	calc. pI
P09405	Nucleolin OS=Mus musculus GN=Ncl PE=1 SV=2 - [NUCL_MOUSE]	21484,69	47,67	1	38	38	698	707	76,7	4,75
P42932	T-complex protein 1 subunit theta OS=Mus musculus GN=Cct8 PE=1 SV=3 - [TCPQ_MOUSE]	20607,19	51,64	1	31	31	667	548	59,5	5,62
Q9JHU4	Cytoplasmic dynein 1 heavy chain 1 OS=Mus musculus GN=Dync1h1 PE=1 SV=2 - [DYHC1_MOUSE]	20312,67	43,20	1	177	177	775	4644	531,7	6,42
Q9D0E1	Heterogeneous nuclear ribonucleoprotein M OS=Mus musculus GN=Hnrnpm PE=1 SV=3 - [HNRPM_MOUSE]	20127,20	47,33	1	28	28	699	729	77,6	8,63
P60710	Actin, cytoplasmic 1 OS=Mus musculus GN=Actb PE=1 SV=1 - [ACTB_MOUSE]	19372,48	54,93	2	6	20	777	375	41,7	5,48
Q68FD5	Clathrin heavy chain 1 OS=Mus musculus GN=Cltc PE=1 SV=3 - [CLH_MOUSE]	19324,05	44,36	1	63	63	715	1675	191,4	5,69
P80314	T-complex protein 1 subunit beta OS=Mus musculus GN=Cct2 PE=1 SV=4 - [TCPB_MOUSE]	18190,87	67,85	1	30	30	477	535	57,4	6,40
P19096	Fatty acid synthase OS=Mus musculus GN=Fasn PE=1 SV=2 - [FAS_MOUSE]	16811,77	38,74	1	76	77	576	2504	272,3	6,58
P80313	T-complex protein 1 subunit eta OS=Mus musculus GN=Cct7 PE=1 SV=1 - [TCPH_MOUSE]	14185,33	45,59	1	23	23	430	544	59,6	7,84
P99024	Tubulin beta-5 chain OS=Mus musculus GN=Tubb5 PE=1 SV=1 - [TBB5_MOUSE]	14077,52	51,35	1	4	20	470	444	49,6	4,89
P14869	60S acidic ribosomal protein P0 OS=Mus musculus GN=Rplp0 PE=1 SV=3 - [RLA0_MOUSE]	13994,75	53,94	1	19	19	443	317	34,2	6,25
P62908	40S ribosomal protein S3 OS=Mus musculus GN=Rps3 PE=1 SV=1 - [RS3_MOUSE]	13847,66	69,14	1	20	20	601	243	26,7	9,66
P11983	T-complex protein 1 subunit alpha OS=Mus musculus GN=Tcp1 PE=1 SV=3 - [TCPA_MOUSE]	13783,81	51,80	1	25	25	440	556	60,4	6,16
P80318	T-complex protein 1 subunit gamma OS=Mus musculus GN=Cct3 PE=1 SV=1 - [TCPG_MOUSE]	12302,19	42,39	1	25	25	459	545	60,6	6,70
P47911	60S ribosomal protein L6 OS=Mus musculus GN=Rpl6 PE=1 SV=3 - [RL6_MOUSE]	12079,93	51,35	1	21	21	416	296	33,5	10,70
P68372	Tubulin beta-2C chain OS=Mus musculus GN=Tubb2c PE=1 SV=1 - [TBB2C_MOUSE]	11864,68	51,24	1	3	20	412	445	49,8	4,89
Q8VEK3	Heterogeneous nuclear ribonucleoprotein U OS=Mus musculus GN=Hnrnpu PE=1 SV=1 - [HNRPU_MOUSE]	11610,07	24,88	1	20	20	415	800	87,9	6,24
O35286	Putative pre-mRNA-splicing factor ATP-dependent RNA helicase DHX15 OS=Mus musculus GN=Dhx15 PE=2 SV=2 - [DHX15_MOUSE]	11545,41	43,90	1	34	35	406	795	90,9	7,46
P29341	Polyadenylate-binding protein 1 OS=Mus musculus GN=Pabpc1 PE=1 SV=2 - [PABP1_MOUSE]	11217,37	42,30	1	27	27	445	636	70,6	9,50
P80315	T-complex protein 1 subunit delta OS=Mus musculus GN=Cct4 PE=1 SV=3 - [TCPD_MOUSE]	10995,95	53,06	1	22	22	349	539	58,0	8,02
P35979	60S ribosomal protein L12 OS=Mus musculus GN=Rpl12 PE=1 SV=2 - [RL12_MOUSE]	10377,82	69,70	1	9	9	333	165	17,8	9,42
Q8C3J5	Dedicator of cytokinesis protein 2 OS=Mus musculus GN=Dock2 PE=1 SV=3 - [DOCK2_MOUSE]	10301,57	30,31	1	48	49	451	1828	211,6	6,99
Q9EQK5	Major vault protein OS=Mus musculus GN=Mvp PE=1 SV=4 - [MVP_MOUSE]	10111,70	56,79	1	39	39	360	861	95,9	5,59
P47963	60S ribosomal protein L13 OS=Mus musculus GN=Rpl13 PE=2 SV=3 - [RL13_MOUSE]	10047,40	51,18	1	13	13	432	211	24,3	11,55
Q01853	Transitional endoplasmic reticulum ATPase OS=Mus musculus GN=Vcp PE=1 SV=4 - [TERA_MOUSE]	10009,54	50,74	1	37	38	350	806	89,3	5,26
P62754	40S ribosomal protein S6 OS=Mus musculus GN=Rps6 PE=1 SV=1 - [RS6_MOUSE]	9970,35	40,16	1	13	13	269	249	28,7	10,84
Q9CWF2	Tubulin beta-2B chain OS=Mus musculus GN=Tubb2b PE=1 SV=1 - [TBB2B_MOUSE]	9849,44	51,24	1	3	20	355	445	49,9	4,89
Q8CGC7	Bifunctional aminoacyl-tRNA synthetase OS=Mus musculus GN=Eprs PE=2 SV=4 - [SYEP_MOUSE]	9845,30	31,68	1	41	41	353	1512	170,0	7,66
P62702	40S ribosomal protein S4, X isoform OS=Mus musculus GN=Rps4x PE=2 SV=2 - [RS4X_MOUSE]	9691,62	56,65	1	19	19	422	263	29,6	10,15
P35980	60S ribosomal protein L18 OS=Mus musculus GN=Rpl18 PE=2 SV=3 - [RL18_MOUSE]	9685,61	47,34	1	13	13	248	188	21,6	11,78
Q921M3	Splicing factor 3B subunit 3 OS=Mus musculus GN=Sf3b3 PE=2 SV=1 - [SF3B3_MOUSE]	9586,65	36,57	1	35	35	258	1217	135,5	5,26
P80316	T-complex protein 1 subunit epsilon OS=Mus musculus GN=Cct5 PE=1 SV=1 - [TCPE_MOUSE]	9576,43	52,68	1	29	29	345	541	59,6	6,02
P62242	40S ribosomal protein S8 OS=Mus musculus GN=Rps8 PE=1 SV=2 - [RS8_MOUSE]	9564,95	45,19	1	12	12	377	208	24,2	10,32
Q62167	ATP-dependent RNA helicase DDX3X OS=Mus musculus GN=Ddx3x PE=1 SV=3 - [DDX3X_MOUSE]	9558,79	35,80	1	22	23	334	662	73,1	7,18
P27659	60S ribosomal protein L3 OS=Mus musculus GN=Rpl3 PE=2 SV=3 - [RL3_MOUSE]	9211,23	51,12	1	20	20	353	403	46,1	10,21
P14131	40S ribosomal protein S16 OS=Mus musculus GN=Rps16 PE=2 SV=4 - [RS16_MOUSE]	9201,98	60,27	1	13	13	427	146	16,4	10,21
Q8BP67	60S ribosomal protein L24 OS=Mus musculus GN=Rpl24 PE=2 SV=2 - [RL24_MOUSE]	9131,03	32,48	1	7	7	259	157	17,8	11,25
Q9CR57	60S ribosomal protein L14 OS=Mus musculus GN=Rpl14 PE=2 SV=3 - [RL14_MOUSE]	9022,19	31,80	1	9	9	208	217	23,5	11,02
Q99PV0	Pre-mRNA-processing-splicing factor 8 OS=Mus musculus GN=Prpf8 PE=1 SV=2 - [PRP8_MOUSE]	9012,07	37,60	1	75	75	443	2335	273,4	8,84
P62264	40S ribosomal protein S14 OS=Mus musculus GN=Rps14 PE=2 SV=3 - [RS14_MOUSE]	8962,66	40,40	1	8	8	387	151	16,3	10,05
Q9D8E6	60S ribosomal protein L4 OS=Mus musculus GN=Rpl4 PE=1 SV=3 - [RL4_MOUSE]	8822,17	42,72	1	23	23	442	419	47,1	11,00
Q6ZWV3	60S ribosomal protein L10 OS=Mus musculus GN=Rpl10 PE=2 SV=3 - [RL10_MOUSE]	8794,81	35,51	1	10	10	357	214	24,6	10,08
P68373	Tubulin alpha-1C chain OS=Mus musculus GN=Tuba1c PE=1 SV=1 - [TBA1C_MOUSE]	8671,19	42,32	2	15	15	273	449	49,9	5,10
P63017	Heat shock cognate 71 kDa protein OS=Mus musculus GN=Hspa8 PE=1 SV=1 - [HSP7C_MOUSE]	8509,51	48,14	1	25	29	312	646	70,8	5,52
P62960	Nuclease-sensitive element-binding protein 1 OS=Mus musculus GN=Ybx1 PE=1 SV=3 - [YBOX1_MOUSE]	8337,45	52,48	1	8	12	249	322	35,7	9,88
P68033	Actin, alpha cardiac muscle 1 OS=Mus musculus GN=Actc1 PE=1 SV=1 - [ACTC_MOUSE]	8299,03	40,58	2	4	16	381	377	42,0	5,39
P61979	Heterogeneous nuclear ribonucleoprotein K OS=Mus musculus GN=Hnrnpk PE=1 SV=1 - [HNRPK_MOUSE]	8198,02	46,44	1	18	18	240	463	50,9	5,54
Q61656	Probable ATP-dependent RNA helicase DDX5 OS=Mus musculus GN=Ddx5 PE=1 SV=2 - [DDX5_MOUSE]	8008,42	33,88	1	17	23	349	614	69,2	8,92
P12970	60S ribosomal protein L7a OS=Mus musculus GN=Rpl7a PE=2 SV=2 - [RL7A_MOUSE]	7987,72	40,60	1	14	14	323	266	30,0	10,56
Q7TMK9	Heterogeneous nuclear ribonucleoprotein Q OS=Mus musculus GN=Syncrip PE=1 SV=2 - [HNRPQ_MOUSE]	7936,63	38,20	1	23	23	328	623	69,6	8,59
P80317	T-complex protein 1 subunit zeta OS=Mus musculus GN=Cct6a PE=1 SV=3 - [TCPZ_MOUSE]	7746,08	45,01	1	25	25	300	531	58,0	7,08
Q8CG48	Structural maintenance of chromosomes protein 2 OS=Mus musculus GN=Smc2 PE=1 SV=2 - [SMC2_MOUSE]	7707,96	41,23	1	44	44	298	1191	134,2	8,41
P16858	Glyceraldehyde-3-phosphate dehydrogenase OS=Mus musculus GN=Gapdh PE=1 SV=2 - [G3P_MOUSE]	7699,34	44,14	1	11	11	247	333	35,8	8,25
P26041	Moesin OS=Mus musculus GN=Msn PE=1 SV=3 - [MOES_MOUSE]	7637,81	55,46	1	25	34	300	577	67,7	6,60
P97351	40S ribosomal protein S3a OS=Mus musculus GN=Rps3a PE=1 SV=3 - [RS3A_MOUSE]	7620,79	71,21	1	25	25	350	264	29,9	9,73
Q501J6	Probable ATP-dependent RNA helicase DDX17 OS=Mus musculus GN=Ddx17 PE=2 SV=1 - [DDX17_MOUSE]	7510,69	36,62	1	18	24	283	650	72,4	8,59
P62751	60S ribosomal protein L23a OS=Mus musculus GN=Rpl23a PE=1 SV=1 - [RL23A_MOUSE]	7379,20	53,85	1	13	13	274	156	17,7	10,45
Q91V92	ATP-citrate synthase OS=Mus musculus GN=Acly PE=1 SV=1 - [ACLY_MOUSE]	7217,63	36,02	1	32	32	220	1091	119,7	7,44
Q8BG05	Heterogeneous nuclear ribonucleoprotein A3 OS=Mus musculus GN=Hnrnpa3 PE=1 SV=1 - [ROA3_MOUSE]	7037,56	39,31	1	17	18	228	379	39,6	9,01
Q6ZWN5	40S ribosomal protein S9 OS=Mus musculus GN=Rps9 PE=2 SV=3 - [RS9_MOUSE]	6987,11	62,37	1	20	20	374	194	22,6	10,65
Q9Z2X1	Heterogeneous nuclear ribonucleoprotein F OS=Mus musculus GN=Hnrnpf PE=1 SV=3 - [HNRPF_MOUSE]	6981,60	39,52	1	11	13	236	415	45,7	5,49
Q7TNC4	Putative RNA-binding protein Luc7-like 2 OS=Mus musculus GN=Luc7l2 PE=1 SV=1 - [LC7L2_MOUSE]	6972,71	26,53	1	8	11	169	392	46,6	10,10
P62830	60S ribosomal protein L23 OS=Mus musculus GN=Rpl23 PE=2 SV=1 - [RL23_MOUSE]	6961,12	62,14	1	8	8	269	140	14,9	10,51
P13864	DNA (cytosine-5)-methyltransferase 1 OS=Mus musculus GN=Dnmt1 PE=1 SV=5 - [DNMT1_MOUSE]	6863,12	27,35	1	37	37	256	1620	183,1	7,74
P14148	60S ribosomal protein L7 OS=Mus musculus GN=Rpl7 PE=2 SV=2 - [RL7_MOUSE]	6806,50	49,26	1	22	22	295	270	31,4	10,89
Q9JIK5	Nucleolar RNA helicase 2 OS=Mus musculus GN=Ddx21 PE=1 SV=3 - [DDX21_MOUSE]	6796,65	37,37	1	25	25	259	851	93,5	9,11
Q64514	Tripeptidyl-peptidase 2 OS=Mus musculus GN=Tpp2 PE=2 SV=3 - [TPP2_MOUSE]	6748,09	35,90	1	38	38	291	1262	139,8	6,58
P25444	40S ribosomal protein S2 OS=Mus musculus GN=Rps2 PE=1 SV=3 - [RS2_MOUSE]	6744,92	47,10	1	12	12	334	293	31,2	10,24
P23116	Eukaryotic translation initiation factor 3 subunit A OS=Mus musculus GN=Eif3a PE=1 SV=5 - [EIF3A_MOUSE]	6729,45	33,18	1	41	41	316	1344	161,8	6,77
P62270	40S ribosomal protein S18 OS=Mus musculus GN=Rps18 PE=2 SV=3 - [RS18_MOUSE]	6613,41	52,63	1	11	11	276	152	17,7	10,99
Q9CPR4	60S ribosomal protein L17 OS=Mus musculus GN=Rpl17 PE=2 SV=3 - [RL17_MOUSE]	6567,82	51,09	1	12	12	275	184	21,4	10,18
Q8VDM4	26S proteasome non-ATPase regulatory subunit 2 OS=Mus musculus GN=Psmd2 PE=1 SV=1 - [PSMD2_MOUSE]	6529,55	37,33	1	30	30	234	908	100,1	5,17
Q922B2	Aspartyl-tRNA synthetase, cytoplasmic OS=Mus musculus GN=Dars PE=2 SV=2 - [SYDC_MOUSE]	6486,15	53,29	1	24	24	227	501	57,1	6,49
Q8BTI8	Serine/arginine repetitive matrix protein 2 OS=Mus musculus GN=Srrm2 PE=1 SV=3 - [SRRM2_MOUSE]	6453,91	19,27	1	35	35	216	2703	294,7	12,03
P11499	Heat shock protein HSP 90-beta OS=Mus musculus GN=Hsp90ab1 PE=1 SV=3 - [HS90B_MOUSE]	6307,48	38,81	1	14	29	228	724	83,2	5,03
Q8CG47	Structural maintenance of chromosomes protein 4 OS=Mus musculus GN=Smc4 PE=1 SV=1 - [SMC4_MOUSE]	6128,01	34,14	1	42	42	240	1286	146,8	7,30
P27546	Microtubule-associated protein 4 OS=Mus musculus GN=Map4 PE=1 SV=3 - [MAP4_MOUSE]	6079,70	46,40	1	36	36	285	1125	117,4	4,98
E9Q555	Eukaryotic translation initiation factor 3 subunit C OS=Mus musculus GN=Eif3c PE=1 SV=1 - [EIF3C_MOUSE]	5980,69	19,98	1	19	19	177	911	105,5	5,78
P47962	60S ribosomal protein L5 OS=Mus musculus GN=Rpl5 PE=1 SV=3 - [RL5_MOUSE]	5947,48	38,05	1	15	15	314	297	34,4	9,77
Q6ZWX6	Eukaryotic translation initiation factor 2 subunit 1 OS=Mus musculus GN=Eif2s1 PE=1 SV=3 - [IF2A_MOUSE]	5932,54	57,46	1	22	22	168	315	36,1	5,08
Q7TPV4	Myb-binding protein 1A OS=Mus musculus GN=Mybbp1a PE=1 SV=2 - [MBB1A_MOUSE]	5892,67	28,27	1	33	33	162	1344	151,9	8,95
Q62318	Transcription intermediary factor 1-beta OS=Mus musculus GN=Trim28 PE=1 SV=3 - [TIF1B_MOUSE]	5890,82	29,38	1	19	19	243	834	88,8	5,77
Q9D0I9	Arginyl-tRNA synthetase, cytoplasmic OS=Mus musculus GN=Rars PE=2 SV=2 - [SYRC_MOUSE]	5733,31	41,21	1	25	25	212	660	75,6	7,55
Q8R081	Heterogeneous nuclear ribonucleoprotein L OS=Mus musculus GN=Hnrnpl PE=1 SV=2 - [HNRPL_MOUSE]	5691,65	48,29	1	18	18	302	586	63,9	8,10
E9Q555	E3 ubiquitin-protein ligase RNF213 OS=Mus musculus GN=Rnf213 PE=2 SV=1 - [RN213_MOUSE]	5683,82	20,04	1	83	83	224	5150	584,1	6,77
Q99020	Heterogeneous nuclear ribonucleoprotein A/B OS=Mus musculus GN=Hnrnpab PE=1 SV=1 - [ROAA_MOUSE]	5585,41	36,84	1	13	13	159	285	30,8	7,91
Q9Z204	Heterogeneous nuclear ribonucleoproteins C1/C2 OS=Mus musculus GN=Hnrnpc PE=1 SV=1 - [HNRPC_MOUSE]	5551,79	36,10	1	15	15	176	313	34,4	5,05
P53026	60S ribosomal protein L10a OS=Mus musculus GN=Rpl10a PE=1 SV=3 - [RL10A_MOUSE]	5515,98	38,71	1	11	11	270	217	24,9	9,98
P04104	Keratin, type II cytoskeletal 1 OS=Mus musculus GN=Krt1 PE=1 SV=4 - [K2C1_MOUSE]	5492,21	8,01	1	3	7	168	637	65,6	8,15
P62918	60S ribosomal protein L8 OS=Mus musculus GN=Rpl8 PE=2 SV=2 - [RL8_MOUSE]	5488,76	42,80	1	13	13	159	257	28,0	11,03
A2AN08	E3 ubiquitin-protein ligase UBR4 OS=Mus musculus GN=Ubr4 PE=1 SV=1 - [UBR4_MOUSE]	5431,13	18,47	1	70	70	218	5180	571,9	6,06
P62301	40S ribosomal protein S13 OS=Mus musculus GN=Rps13 PE=1 SV=2 - [RS13_MOUSE]	5331,54	63,58	1	14	14	246	151	17,2	10,54
Q8BFZ3	Beta-actin-like protein 2 OS=Mus musculus GN=Actbl2 PE=1 SV=1 - [ACTBL_MOUSE]	5290,05	31,38	1	2	10	313	376	42,0	5,49
P62245	40S ribosomal protein S15a OS=Mus musculus GN=Rps15a PE=2 SV=2 - [RS15A_MOUSE]	5279,77	70,00	1	12	12	289	130	14,8	10,13
P62196	26S protease regulatory subunit 8 OS=Mus musculus GN=Psmc5 PE=1 SV=1 - [PRS8_MOUSE]	5276,10	56,16	1	19	20	189	406	45,6	7,55
O35737	Heterogeneous nuclear ribonucleoprotein H OS=Mus musculus GN=Hnrnph1 PE=1 SV=3 - [HNRH1_MOUSE]	5200,60	30,51	1	3	9	180	449	49,2	6,30
P10126	Elongation factor 1-alpha 1 OS=Mus musculus GN=Eef1a1 PE=1 SV=3 - [EF1A1_MOUSE]	5115,57	29,87	1	14	14	238	462	50,1	9,01
O70133	ATP-dependent RNA helicase A OS=Mus musculus GN=Dhx9 PE=1 SV=2 - [DHX9_MOUSE]	4990,21	33,26	1	35	35	224	1380	149,4	6,83
P62849	40S ribosomal protein S24 OS=Mus musculus GN=Rps24 PE=1 SV=1 - [RS24_MOUSE]	4972,85	34,59	1	6	6	165	133	15,4	10,78
Q6NVF9	Cleavage and polyadenylation specificity factor subunit 6 OS=Mus musculus GN=Cpsf6 PE=1 SV=1 - [CPSF6_MOUSE]	4914,78	16,88	1	10	10	122	551	59,1	7,15
Q8BU30	Isoleucyl-tRNA synthetase, cytoplasmic OS=Mus musculus GN=Iars PE=2 SV=2 - [SYIC_MOUSE]	4890,70	30,98	1	31	31	241	1262	144,2	6,55
Q8VDD5	Myosin-9 OS=Mus musculus GN=Myh9 PE=1 SV=4 - [MYH9_MOUSE]	4834,77	22,50	1	29	35	169	1960	226,2	5,66
P14483	H-2 class II histocompatibility antigen, A beta chain OS=Mus musculus GN=H2-Ab1 PE=1 SV=1 - [HB2A_MOUSE]	4824,11	40,00	1	10	10	186	265	30,1	7,24
Q8CIE6	Coatomer subunit alpha OS=Mus musculus GN=Copa PE=1 SV=2 - [COPA_MOUSE]	4776,72	25,98	1	26	26	171	1224	138,3	7,65
P07901	Heat shock protein HSP 90-alpha OS=Mus musculus GN=Hsp90aa1 PE=1 SV=4 - [HS90A_MOUSE]	4723,76	35,88	1	15	28	171	733	84,7	5,01
O08810	116 kDa U5 small nuclear ribonucleoprotein component OS=Mus musculus GN=Eftud2 PE=2 SV=1 - [U5S1_MOUSE]	4708,45	35,63	1	27	28	179	971	109,3	5,00
Q99NB9	Splicing factor 3B subunit 1 OS=Mus musculus GN=Sf3b1 PE=1 SV=1 - [SF3B1_MOUSE]	4638,60	40,95	1	39	39	224	1304	145,7	7,09
Q9CZM2	60S ribosomal protein L15 OS=Mus musculus GN=Rpl15 PE=2 SV=4 - [RL15_MOUSE]	4609,72	48,04	1	9	10	228	204	24,1	11,62
P17225	Polypyrimidine tract-binding protein 1 OS=Mus musculus GN=Ptbp1 PE=1 SV=2 - [PTBP1_MOUSE]	4579,29	41,37	1	10	12	164	527	56,4	8,34
Q6A0A9	Constitutive coactivator of PPAR-gamma-like protein 1 OS=Mus musculus GN=FAM120A PE=1 SV=2 - [F120A_MOUSE]	4573,59	23,56	1	20	20	170	1112	121,6	8,92
Q99MN1	Lysyl-tRNA synthetase OS=Mus musculus GN=Kars PE=1 SV=1 - [SYK_MOUSE]	4540,74	36,47	1	23	23	173	595	67,8	5,94
Q62093	Serine/arginine-rich splicing factor 2 OS=Mus musculus GN=Srsf2 PE=1 SV=4 - [SRSF2_MOUSE]	4506,97	34,39	1	7	7	114	221	25,5	11,85
Q8BMJ2	Leucyl-tRNA synthetase, cytoplasmic OS=Mus musculus GN=Lars PE=2 SV=2 - [SYLC_MOUSE]	4460,61	23,43	1	26	27	176	1178	134,1	7,05
P14685	26S proteasome non-ATPase regulatory subunit 3 OS=Mus musculus GN=Psmd3 PE=2 SV=3 - [PSMD3_MOUSE]	4454,66	59,43	1	29	29	193	530	60,7	8,44
O08582	GTP-binding protein 1 OS=Mus musculus GN=Gtpbp1 PE=1 SV=2 - [GTPB1_MOUSE]	4409,91	28,44	1	14	14	130	668	72,3	8,29
P02535	Keratin, type I cytoskeletal 10 OS=Mus musculus GN=Krt10 PE=1 SV=3 - [K1C10_MOUSE]	4403,59	12,28	1	6	8	114	570	57,7	5,11
P14206	40S ribosomal protein SA OS=Mus musculus GN=Rpsa PE=1 SV=4 - [RSSA_MOUSE]	4352,97	41,69	1	11	11	128	295	32,8	4,87
P26040	Ezrin OS=Mus musculus GN=Ezr PE=1 SV=3 - [EZRI_MOUSE]	4348,66	30,38	1	9	18	168	586	69,4	6,10

Q3TXS7	26S proteasome non-ATPase regulatory subunit 1 OS=Mus musculus GN=Psmd1 PE=1 SV=1 - [PSMD1_MOUSE]	4333,44	35,47	1	25	25	180	953	105,7	5,39
P08752	Guanine nucleotide-binding protein G(i) subunit alpha-2 OS=Mus musculus GN=Gnai2 PE=1 SV=5 - [GNAI2_MOUSE]	4273,46	31,83	1	6	9	129	355	40,5	5,45
P28063	Proteasome subunit beta type-8 OS=Mus musculus GN=Psmb8 PE=1 SV=2 - [PSB8_MOUSE]	4264,64	44,93	1	11	11	145	276	30,2	6,68
Q01320	DNA topoisomerase 2-alpha OS=Mus musculus GN=Top2a PE=1 SV=2 - [TOP2A_MOUSE]	4254,80	20,75	1	21	29	159	1528	172,7	8,60
P26043	Radixin OS=Mus musculus GN=Rdx PE=1 SV=3 - [RADI_MOUSE]	4253,42	33,45	1	12	22	163	583	68,5	6,20
Q9QUM9	Proteasome subunit alpha type-6 OS=Mus musculus GN=Psma6 PE=1 SV=1 - [PSA6_MOUSE]	4241,42	38,21	1	10	10	131	246	27,4	6,76
P46471	26S protease regulatory subunit 7 OS=Mus musculus GN=Psmc2 PE=1 SV=5 - [PRS7_MOUSE]	4188,69	41,11	1	17	17	152	433	48,6	5,95
Q8JZQ9	Eukaryotic translation initiation factor 3 subunit B OS=Mus musculus GN=Eif3b PE=1 SV=1 - [EIF3B_MOUSE]	4180,70	42,09	1	25	25	169	803	91,3	5,02
Q6ZQ08	CCR4-NOT transcription complex subunit 1 OS=Mus musculus GN=Cnot1 PE=1 SV=2 - [CNOT1_MOUSE]	4168,91	18,74	1	39	39	192	2375	266,6	7,11
P20029	78 kDa glucose-regulated protein OS=Mus musculus GN=Hspa5 PE=1 SV=3 - [GRP78_MOUSE]	4157,70	43,36	1	24	26	127	655	72,4	5,16
O70435	Proteasome subunit alpha type-3 OS=Mus musculus GN=Psma3 PE=1 SV=3 - [PSA3_MOUSE]	4147,04	56,47	1	13	13	145	255	28,4	5,44
O88569	Heterogeneous nuclear ribonucleoproteins A2/B1 OS=Mus musculus GN=Hnrnpa2b1 PE=1 SV=2 - [ROA2_MOUSE]	4136,92	56,37	1	17	19	174	353	37,4	8,95
Q9Z1Q9	Valyl-tRNA synthetase OS=Mus musculus GN=Vars PE=2 SV=1 - [SYVC_MOUSE]	4105,21	21,06	1	20	21	129	1263	140,1	7,77
Q9EPU0	Regulator of nonsense transcripts 1 OS=Mus musculus GN=Upf1 PE=1 SV=2 - [RENT1_MOUSE]	4098,45	30,25	1	25	25	167	1124	123,9	6,61
P49718	DNA replication licensing factor MCM5 OS=Mus musculus GN=Mcm5 PE=2 SV=1 - [MCM5_MOUSE]	4065,90	46,93	1	28	28	170	733	82,3	8,43
Q61584	Fragile X mental retardation syndrome-related protein 1 OS=Mus musculus GN=Fxr1 PE=1 SV=2 - [FXR1_MOUSE]	4019,62	41,65	1	21	22	148	677	76,2	6,98
Q8VH51	RNA-binding protein 39 OS=Mus musculus GN=Rbm39 PE=1 SV=2 - [RBM39_MOUSE]	3993,13	35,47	1	14	14	119	530	59,4	10,10
P62855	40S ribosomal protein S26 OS=Mus musculus GN=Rps26 PE=2 SV=3 - [RS26_MOUSE]	3917,20	50,43	1	5	5	160	115	13,0	11,00
Q91VR5	ATP-dependent RNA helicase DDX1 OS=Mus musculus GN=Ddx1 PE=2 SV=1 - [DDX1_MOUSE]	3899,66	35,14	1	23	23	181	740	82,4	7,21
P26039	Talin-1 OS=Mus musculus GN=Tln1 PE=1 SV=2 - [TLN1_MOUSE]	3896,79	18,18	1	33	33	132	2541	269,7	6,18
Q8VDP4	Protein KIAA1967 homolog OS=Mus musculus PE=1 SV=2 - [K1967_MOUSE]	3862,13	33,41	1	22	22	137	922	102,9	5,25
Q68FL6	Methionyl-tRNA synthetase, cytoplasmic OS=Mus musculus GN=Mars PE=2 SV=1 - [SYMC_MOUSE]	3854,59	30,82	1	23	23	202	902	101,4	7,14
P84099	60S ribosomal protein L19 OS=Mus musculus GN=Rpl19 PE=1 SV=1 - [RL19_MOUSE]	3832,89	36,73	1	10	10	96	196	23,5	11,47
Q922F4	Tubulin beta-6 chain OS=Mus musculus GN=Tubb6 PE=1 SV=1 - [TBB6_MOUSE]	3800,91	37,81	1	6	16	147	447	50,1	4,89
Q60865	Caprin-1 OS=Mus musculus GN=Caprin1 PE=1 SV=2 - [CAPR1_MOUSE]	3796,67	22,21	1	10	10	135	707	78,1	5,25
Q9CXW4	60S ribosomal protein L11 OS=Mus musculus GN=Rpl11 PE=1 SV=4 - [RL11_MOUSE]	3796,47	38,76	1	8	8	135	178	20,2	9,60
Q5SUF2	Luc7-like protein 3 OS=Mus musculus GN=Luc7l3 PE=1 SV=1 - [LC7L3_MOUSE]	3782,07	17,59	1	7	7	73	432	51,4	9,77
P14115	60S ribosomal protein L27a OS=Mus musculus GN=Rpl27a PE=2 SV=5 - [RL27A_MOUSE]	3779,53	54,73	1	9	9	163	148	16,6	11,12
Q8BG32	26S proteasome non-ATPase regulatory subunit 11 OS=Mus musculus GN=Psmd11 PE=1 SV=3 - [PSD11_MOUSE]	3758,23	56,87	1	22	22	120	422	47,4	6,48
P19253	60S ribosomal protein L13a OS=Mus musculus GN=Rpl13a PE=1 SV=4 - [RL13A_MOUSE]	3696,86	53,20	1	15	15	155	203	23,4	11,02
Q61033	Lamina-associated polypeptide 2, isoforms alpha/zeta OS=Mus musculus GN=Tmpo PE=1 SV=4 - [LAP2A_MOUSE]	3692,49	34,34	1	17	17	132	693	75,1	8,05
P50580	Proliferation-associated protein 2G4 OS=Mus musculus GN=Pa2g4 PE=1 SV=3 - [PA2G4_MOUSE]	3685,52	47,46	1	19	19	185	394	43,7	6,86
O08788	Dynactin subunit 1 OS=Mus musculus GN=Dctn1 PE=1 SV=3 - [DCTN1_MOUSE]	3678,50	32,79	1	31	31	148	1281	141,6	5,90
P10853	Histone H2B type 1-F/J/L OS=Mus musculus GN=Hist1h2bf PE=1 SV=2 - [H2B1F_MOUSE]	3677,19	53,17	8	10	10	176	126	13,9	10,32
Q6PDQ2	Chromodomain-helicase-DNA-binding protein 4 OS=Mus musculus GN=Chd4 PE=1 SV=1 - [CHD4_MOUSE]	3659,97	25,64	1	38	38	188	1915	217,6	5,81
P70333	Heterogeneous nuclear ribonucleoprotein H2 OS=Mus musculus GN=Hnrnph2 PE=1 SV=1 - [HNRH2_MOUSE]	3653,57	26,06	1	3	8	130	449	49,2	6,30
O88685	26S protease regulatory subunit 6A OS=Mus musculus GN=Psmc3 PE=2 SV=2 - [PRS6A_MOUSE]	3648,09	53,17	1	20	20	155	442	49,5	5,19
Q61937	Nucleophosmin OS=Mus musculus GN=Npm1 PE=1 SV=1 - [NPM_MOUSE]	3644,37	37,67	1	10	10	128	292	32,5	4,77
P62267	40S ribosomal protein S23 OS=Mus musculus GN=Rps23 PE=2 SV=3 - [RS23_MOUSE]	3641,33	39,16	1	7	7	118	143	15,8	10,49
O88487	Cytoplasmic dynein 1 intermediate chain 2 OS=Mus musculus GN=Dync1i2 PE=2 SV=1 - [DC1I2_MOUSE]	3621,81	24,67	1	13	13	85	612	68,4	5,29
Q9Z315	U4/U6.U5 tri-snRNP-associated protein 1 OS=Mus musculus GN=Sart1 PE=2 SV=1 - [SNUT1_MOUSE]	3605,31	23,57	1	13	14	92	806	90,8	5,82
P29391	Ferritin light chain 1 OS=Mus musculus GN=Ftl1 PE=1 SV=2 - [FRIL1_MOUSE]	3576,34	63,93	1	11	11	98	183	20,8	6,00
P51410	60S ribosomal protein L9 OS=Mus musculus GN=Rpl9 PE=2 SV=2 - [RL9_MOUSE]	3532,05	63,54	1	12	12	139	192	21,9	9,95
P43277	Histone H1.3 OS=Mus musculus GN=Hist1h1d PE=1 SV=2 - [H13_MOUSE]	3491,06	32,58	1	1	10	127	221	22,1	11,03
P62852	40S ribosomal protein S25 OS=Mus musculus GN=Rps25 PE=2 SV=1 - [RS25_MOUSE]	3456,89	29,60	1	5	5	152	125	13,7	10,11
Q99LF4	UPF0027 protein C22orf28 homolog OS=Mus musculus GN=D10Wsu52e PE=2 SV=1 - [CV028_MOUSE]	3453,11	48,91	1	20	20	157	505	55,2	7,23
Q61838	Alpha-2-macroglobulin OS=Mus musculus GN=A2m PE=1 SV=3 - [A2M_MOUSE]	3429,87	28,70	1	35	35	162	1495	165,7	6,68
P63325	40S ribosomal protein S10 OS=Mus musculus GN=Rps10 PE=1 SV=1 - [RS10_MOUSE]	3424,94	49,70	1	11	11	142	165	18,9	10,15
P15864	Histone H1.2 OS=Mus musculus GN=Hist1h1c PE=1 SV=2 - [H12_MOUSE]	3406,09	41,04	1	3	12	127	212	21,3	11,00
Q9CYI4	Putative RNA-binding protein Luc7-like 1 OS=Mus musculus GN=Luc7l PE=2 SV=2 - [LUC7L_MOUSE]	3401,02	25,61	1	7	10	84	371	43,9	9,88
P99027	60S acidic ribosomal protein P2 OS=Mus musculus GN=Rplp2 PE=1 SV=3 - [RLA2_MOUSE]	3371,61	79,13	1	7	7	112	115	11,6	4,54
Q8BTM8	Filamin-A OS=Mus musculus GN=Flna PE=1 SV=5 - [FLNA_MOUSE]	3328,38	19,76	1	33	35	128	2647	281,0	6,04
Q61781	Keratin, type I cytoskeletal 14 OS=Mus musculus GN=Krt14 PE=1 SV=2 - [K1C14_MOUSE]	3269,77	26,24	1	1	13	106	484	52,8	5,17
P99026	Proteasome subunit beta type-4 OS=Mus musculus GN=Psmb4 PE=1 SV=1 - [PSB4_MOUSE]	3216,40	50,38	1	9	9	92	264	29,1	5,64
Q6ZWU9	40S ribosomal protein S27 OS=Mus musculus GN=Rps27 PE=1 SV=3 - [RS27_MOUSE]	3199,20	45,24	1	3	6	111	84	9,5	9,45
Q9R190	Metastasis-associated protein MTA2 OS=Mus musculus GN=Mta2 PE=1 SV=1 - [MTA2_MOUSE]	3136,36	32,93	1	16	19	119	668	75,0	9,67
Q05D44	Eukaryotic translation initiation factor 5B OS=Mus musculus GN=Eif5b PE=1 SV=2 - [IF2P_MOUSE]	3135,58	24,42	1	23	23	141	1216	137,5	5,59
P97855	Ras GTPase-activating protein-binding protein 1 OS=Mus musculus GN=G3bp1 PE=1 SV=1 - [G3BP1_MOUSE]	3117,71	52,04	1	15	16	117	465	51,8	5,59
Q8BPU7	Engulfment and cell motility protein 1 OS=Mus musculus GN=Elmo1 PE=1 SV=2 - [ELMO1_MOUSE]	3108,92	35,63	1	20	23	118	727	83,9	6,28
O09167	60S ribosomal protein L21 OS=Mus musculus GN=Rpl21 PE=2 SV=3 - [RL21_MOUSE]	3058,96	43,13	1	8	8	144	160	18,6	10,49
Q6ZWY3	40S ribosomal protein S27-like OS=Mus musculus GN=Rps27l PE=2 SV=3 - [RS27L_MOUSE]	3026,94	40,48	1	2	5	93	84	9,5	9,45
Q9D8W5	26S proteasome non-ATPase regulatory subunit 12 OS=Mus musculus GN=Psmd12 PE=2 SV=4 - [PSD12_MOUSE]	3001,30	38,82	1	17	17	90	456	52,9	7,06
Q9R1P1	Proteasome subunit beta type-3 OS=Mus musculus GN=Psmb3 PE=1 SV=1 - [PSB3_MOUSE]	2998,10	50,24	1	11	11	98	205	22,9	6,55
P62334	26S protease regulatory subunit 10B OS=Mus musculus GN=Psmc6 PE=1 SV=1 - [PRS10_MOUSE]	2984,10	49,87	1	16	16	107	389	44,1	7,49
P01872	Ig mu chain C region secreted form OS=Mus musculus GN=Igh-6 PE=1 SV=2 - [IGHM_MOUSE]	2963,35	22,91	2	8	8	78	454	49,9	7,01
O09061	Proteasome subunit beta type-1 OS=Mus musculus GN=Psmb1 PE=1 SV=1 - [PSB1_MOUSE]	2962,38	52,92	1	12	12	92	240	26,4	7,81
P50446	Keratin, type II cytoskeletal 6A OS=Mus musculus GN=Krt6a PE=2 SV=3 - [K2C6A_MOUSE]	2957,59	18,81	1	8	13	82	553	59,3	7,94
Q9Z0N1	Eukaryotic translation initiation factor 2 subunit 3, X-linked OS=Mus musculus GN=Eif2s3x PE=1 SV=2 - [IF2G_MOUSE]	2883,83	40,25	1	15	15	130	472	51,0	8,40
Q6P542	ATP-binding cassette sub-family F member 1 OS=Mus musculus GN=Abcf1 PE=1 SV=1 - [ABCF1_MOUSE]	2874,52	24,13	1	16	16	104	837	94,9	6,51
P60867	40S ribosomal protein S20 OS=Mus musculus GN=Rps20 PE=1 SV=1 - [RS20_MOUSE]	2870,50	23,53	1	4	4	159	119	13,4	9,94
P49722	Proteasome subunit alpha type-2 OS=Mus musculus GN=Psma2 PE=1 SV=3 - [PSA2_MOUSE]	2862,11	54,70	1	12	12	86	234	25,9	7,43
Q9JKF1	Ras GTPase-activating-like protein IQGAP1 OS=Mus musculus GN=Iqgap1 PE=1 SV=2 - [IQGA1_MOUSE]	2858,47	21,97	1	30	30	119	1657	188,6	6,48
Q8QZY1	Eukaryotic translation initiation factor 3 subunit L OS=Mus musculus GN=Eif3l PE=1 SV=1 - [EIF3L_MOUSE]	2854,72	44,68	1	25	25	95	564	66,6	6,44
P62806	Histone H4 OS=Mus musculus GN=Hist1h4a PE=1 SV=2 - [H4_MOUSE]	2853,10	60,19	1	11	11	112	103	11,4	11,36
Q99PL5	Ribosome-binding protein 1 OS=Mus musculus GN=Rrbp1 PE=2 SV=2 - [RRBP1_MOUSE]	2833,60	18,01	1	23	23	99	1605	172,8	9,33
Q9CZX8	40S ribosomal protein S19 OS=Mus musculus GN=Rps19 PE=1 SV=3 - [RS19_MOUSE]	2812,97	60,69	1	12	12	143	145	16,1	10,40
P62889	60S ribosomal protein L30 OS=Mus musculus GN=Rpl30 PE=2 SV=2 - [RL30_MOUSE]	2798,16	73,91	1	8	8	100	115	12,8	9,63
Q60668	Heterogeneous nuclear ribonucleoprotein D0 OS=Mus musculus GN=Hnrnpd PE=1 SV=2 - [HNRPD_MOUSE]	2797,47	32,68	1	12	12	115	355	38,3	7,81
P70372	ELAV-like protein 1 OS=Mus musculus GN=Elavl1 PE=1 SV=2 - [ELAV1_MOUSE]	2786,98	46,93	1	14	14	92	326	36,1	9,04
Q8R1Q8	Cytoplasmic dynein 1 light intermediate chain 1 OS=Mus musculus GN=Dync1li1 PE=1 SV=1 - [DC1L1_MOUSE]	2786,01	43,40	1	17	17	95	523	56,6	6,42
P01831	Thy-1 membrane glycoprotein OS=Mus musculus GN=Thy1 PE=1 SV=1 - [THY1_MOUSE]	2772,63	33,33	1	6	6	145	162	18,1	8,97
P49312	Heterogeneous nuclear ribonucleoprotein A1 OS=Mus musculus GN=Hnrnpa1 PE=1 SV=2 - [ROA1_MOUSE]	2769,29	43,13	1	12	14	117	320	34,2	9,23
P61164	Alpha-centractin OS=Mus musculus GN=Actr1a PE=2 SV=1 - [ACTZ_MOUSE]	2747,20	53,99	1	6	15	85	376	42,6	6,64
Q8K4Z5	Splicing factor 3A subunit 1 OS=Mus musculus GN=Sf3a1 PE=1 SV=1 - [SF3A1_MOUSE]	2702,02	25,54	1	17	17	96	791	88,5	5,22
P62192	26S protease regulatory subunit 4 OS=Mus musculus GN=Psmc1 PE=1 SV=1 - [PRS4_MOUSE]	2698,70	51,82	1	17	18	147	440	49,2	6,21
P56480	ATP synthase subunit beta, mitochondrial OS=Mus musculus GN=Atp5b PE=1 SV=2 - [ATPB_MOUSE]	2687,22	57,09	1	19	19	84	529	56,3	5,34
Q6A026	Sister chromatid cohesion protein PDS5 homolog A OS=Mus musculus GN=Pds5a PE=2 SV=3 - [PDS5A_MOUSE]	2649,42	11,86	1	11	12	66	1332	150,2	7,85
Q9CU62	Structural maintenance of chromosomes protein 1A OS=Mus musculus GN=Smc1a PE=1 SV=4 - [SMC1A_MOUSE]	2647,54	29,03	1	33	33	98	1233	143,1	7,64
P67984	60S ribosomal protein L22 OS=Mus musculus GN=Rpl22 PE=2 SV=2 - [RL22_MOUSE]	2645,78	36,72	1	5	6	106	128	14,7	9,19
Q9CZU3	Superkiller viralicidic activity 2-like 2 OS=Mus musculus GN=Skiv2l2 PE=2 SV=1 - [SK2L2_MOUSE]	2640,77	28,17	1	25	25	98	1040	117,6	6,40
P62307	Small nuclear ribonucleoprotein F OS=Mus musculus GN=Snrpf PE=2 SV=1 - [RUXF_MOUSE]	2622,24	39,53	1	2	2	64	86	9,7	4,67
Q8R574	Phosphoribosyl pyrophosphate synthase-associated protein 2 OS=Mus musculus GN=Prpsap2 PE=1 SV=1 - [KPRB_MOUSE]	2599,07	49,05	1	11	13	111	369	40,9	7,17
P11103	Poly [ADP-ribose] polymerase 1 OS=Mus musculus GN=Parp1 PE=1 SV=3 - [PARP1_MOUSE]	2593,23	22,41	1	16	16	92	1013	113,0	8,95
P07724	Serum albumin OS=Mus musculus GN=Alb PE=1 SV=3 - [ALBU_MOUSE]	2587,56	23,03	1	12	12	79	608	68,6	6,07
Q9DBG3	AP-2 complex subunit beta OS=Mus musculus GN=Ap2b1 PE=1 SV=1 - [AP2B1_MOUSE]	2552,14	28,18	1	13	22	114	937	104,5	5,38
Q9R1P3	Proteasome subunit beta type-2 OS=Mus musculus GN=Psmb2 PE=1 SV=1 - [PSB2_MOUSE]	2547,71	47,26	1	10	10	95	201	22,9	7,02
Q9CW03	Structural maintenance of chromosomes protein 3 OS=Mus musculus GN=Smc3 PE=1 SV=2 - [SMC3_MOUSE]	2540,21	29,66	1	31	31	98	1217	141,5	7,18
P32067	Lupus La protein homolog OS=Mus musculus GN=Ssb PE=2 SV=1 - [LA_MOUSE]	2533,84	28,43	1	12	12	81	415	47,7	9,77
Q9Z2U0	Proteasome subunit alpha type-7 OS=Mus musculus GN=Psma7 PE=1 SV=1 - [PSA7_MOUSE]	2520,53	57,26	1	13	13	83	248	27,8	8,46
Q7TT37	Elongator complex protein 1 OS=Mus musculus GN=Ikbkap PE=2 SV=2 - [ELP1_MOUSE]	2519,42	14,63	1	16	16	96	1333	149,5	6,00
P62082	40S ribosomal protein S7 OS=Mus musculus GN=Rps7 PE=2 SV=1 - [RS7_MOUSE]	2513,67	69,59	1	14	14	106	194	22,1	10,10
Q9D6Z1	Nucleolar protein 56 OS=Mus musculus GN=Nop56 PE=1 SV=2 - [NOP56_MOUSE]	2504,42	25,34	1	14	14	63	580	64,4	9,14
Q00PI9	Heterogeneous nuclear ribonucleoprotein U-like protein 2 OS=Mus musculus GN=Hnrnpul2 PE=1 SV=2 - [HNRL2_MOUSE]	2477,17	19,19	1	17	17	82	745	84,9	4,89
Q5SSH7	Zinc finger ZZ-type and EF-hand domain-containing protein 1 OS=Mus musculus GN=Zzef1 PE=1 SV=2 - [ZZEF1_MOUSE]	2475,91	10,64	1	23	23	81	2924	328,1	6,11
Q9DCH4	Eukaryotic translation initiation factor 3 subunit F OS=Mus musculus GN=Eif3f PE=1 SV=2 - [EIF3F_MOUSE]	2470,14	33,52	1	10	10	80	361	38,0	5,58
Q99KJ8	Dynactin subunit 2 OS=Mus musculus GN=Dctn2 PE=1 SV=3 - [DCTN2_MOUSE]	2452,32	39,80	1	14	14	60	402	44,1	5,26
Q91VM5	Heterogeneous nuclear ribonucleoprotein G-like 1 OS=Mus musculus GN=Rbmxl1 PE=2 SV=1 - [RBMXL_MOUSE]	2439,24	22,42	1	1	9	94	388	42,1	9,99
Q6ZQK0	Condensin-2 complex subunit D3 OS=Mus musculus GN=Ncapd3 PE=1 SV=3 - [CNDD3_MOUSE]	2434,58	15,01	1	17	17	90	1506	169,3	7,15
Q8K310	Matrin-3 OS=Mus musculus GN=Matr3 PE=1 SV=1 - [MATR3_MOUSE]	2408,70	22,70	1	13	13	90	846	94,6	6,25
Q9WV02	RNA-binding motif protein, X chromosome OS=Mus musculus GN=Rbmx PE=1 SV=1 - [RBMX_MOUSE]	2408,00	28,64	1	2	10	94	391	42,3	10,05
P43274	Histone H1.4 OS=Mus musculus GN=Hist1h1e PE=1 SV=2 - [H14_MOUSE]	2407,18	32,88	1	4	10	84	219	22,0	11,11
Q3UEB3	Poly(U)-binding-splicing factor PUF60 OS=Mus musculus GN=Puf60 PE=2 SV=2 - [PUF60_MOUSE]	2402,57	43,09	1	17	17	79	564	60,2	5,29
Q9Z2K1	Keratin, type I cytoskeletal 16 OS=Mus musculus GN=Krt16 PE=1 SV=3 - [K1C16_MOUSE]	2395,95	18,55	1	6	10	89	469	51,6	5,20
Q8R5C5	Beta-centractin OS=Mus musculus GN=Actr1b PE=1 SV=1 - [ACTY_MOUSE]	2389,08	53,99	1	5	14	73	376	42,3	6,40
P06800	Receptor-type tyrosine-protein phosphatase C OS=Mus musculus GN=Ptprc PE=1 SV=3 - [PTPRC_MOUSE]	2367,53	12,55	1	14	15	89	1291	144,5	5,94
Q9JHW4	Selenocysteine-specific elongation factor OS=Mus musculus GN=Eefsec PE=2 SV=2 - [SELB_MOUSE]	2349,69	22,81	1	11	11	93	583	63,5	8,29
Q569Z6	Thyroid hormone receptor-associated protein 3 OS=Mus musculus GN=Thrap3 PE=1 SV=1 - [TR150_MOUSE]	2349,62	20,19	1	13	13	107	951	108,1	10,17
P26645	Myristoylated alanine-rich C-kinase substrate OS=Mus musculus GN=Marcks PE=1 SV=2 - [MARCS_MOUSE]	2331,55	49,19	1	9	9	91	309	29,6	4,34
P56959	RNA-binding protein FUS OS=Mus musculus GN=Fus PE=2 SV=1 - [FUS_MOUSE]	2323,00	13,51	1	7	7	138	518	52,6	9,36
Q9R1P4	Proteasome subunit alpha type-1 OS=Mus musculus GN=Psma1 PE=1 SV=1 - [PSA1_MOUSE]	2305,69	48,29	1	12	12	131	263	29,5	6,46
Q9QXZ0	Microtubule-actin cross-linking factor 1 OS=Mus musculus GN=Macf1 PE=1 SV=2 - [MACF1_MOUSE]	2283,32	6,24	1	34	34	80	7354	831,4	5,43

Q9Z1X4	Interleukin enhancer-binding factor 3 OS=Mus musculus GN=Ilf3 PE=1 SV=2 - [ILF3_MOUSE]	2273,24	32,52	1	19	22	82	898	96,0	8,76
Q9D0M1	Phosphoribosyl pyrophosphate synthase-associated protein 1 OS=Mus musculus GN=Prpsap1 PE=1 SV=1 - [KPRA_MOUSE]	2261,25	49,16	1	10	12	95	356	39,4	7,20
Q9CY58	Plasminogen activator inhibitor 1 RNA-binding protein OS=Mus musculus GN=Serbp1 PE=1 SV=2 - [PAIRB_MOUSE]	2260,02	39,07	1	13	13	132	407	44,7	8,54
P35564	Calnexin OS=Mus musculus GN=Canx PE=1 SV=1 - [CALX_MOUSE]	2258,69	24,37	1	13	13	65	591	67,2	4,64
P35922	Fragile X mental retardation protein 1 homolog OS=Mus musculus GN=Fmr1 PE=1 SV=1 - [FMR1_MOUSE]	2256,79	25,24	1	10	13	75	614	68,9	7,62
Q922U2	Keratin, type II cytoskeletal 5 OS=Mus musculus GN=Krt5 PE=1 SV=1 - [K2C5_MOUSE]	2256,73	15,52	1	4	10	61	580	61,7	7,75
Q9QWL7	Keratin, type I cytoskeletal 17 OS=Mus musculus GN=Krt17 PE=1 SV=3 - [K1C17_MOUSE]	2256,29	29,79	1	4	15	78	433	48,1	5,06
Q8BJW6	Eukaryotic translation initiation factor 2A OS=Mus musculus GN=Eif2a PE=2 SV=2 - [EIF2A_MOUSE]	2247,10	42,51	1	19	19	95	581	64,4	8,91
Q64511	DNA topoisomerase 2-beta OS=Mus musculus GN=Top2b PE=1 SV=2 - [TOP2B_MOUSE]	2244,38	12,10	1	9	17	86	1612	181,8	8,29
P62717	60S ribosomal protein L18a OS=Mus musculus GN=Rpl18a PE=1 SV=1 - [RL18A_MOUSE]	2243,08	60,23	1	15	15	134	176	20,7	10,71
Q8VE97	Serine/arginine-rich splicing factor 4 OS=Mus musculus GN=Srsf4 PE=2 SV=1 - [SRSF4_MOUSE]	2234,74	13,91	1	6	7	85	489	55,9	11,40
Q9JIX8	Apoptotic chromatin condensation inducer in the nucleus OS=Mus musculus GN=Acin1 PE=1 SV=3 - [ACINU_MOUSE]	2225,13	18,68	1	20	20	103	1338	150,6	5,91
P84104	Serine/arginine-rich splicing factor 3 OS=Mus musculus GN=Srsf3 PE=2 SV=1 - [SRSF3_MOUSE]	2192,52	40,85	1	6	7	110	164	19,3	11,65
Q9CS42	Ribose-phosphate pyrophosphokinase 2 OS=Mus musculus GN=Prps2 PE=1 SV=4 - [PRPS2_MOUSE]	2175,85	43,71	1	8	14	77	318	34,8	6,61
Q9JKB3	DNA-binding protein A OS=Mus musculus GN=Csda PE=1 SV=2 - [DBPA_MOUSE]	2141,40	44,88	1	5	9	61	361	38,8	9,69
Q9JIF7	Coatomer subunit beta OS=Mus musculus GN=Copb1 PE=1 SV=1 - [COPB_MOUSE]	2136,26	24,34	1	16	16	73	953	107,0	6,00
O88738	Baculoviral IAP repeat-containing protein 6 OS=Mus musculus GN=Birc6 PE=1 SV=2 - [BIRC6_MOUSE]	2129,54	7,82	1	28	28	82	4882	531,8	6,07
Q8CGP6	Histone H2A type 1-H OS=Mus musculus GN=Hist1h2ah PE=1 SV=3 - [H2A1H_MOUSE]	2122,23	36,72	6	4	6	95	128	13,9	11,03
Q9WTM5	RuvB-like 2 OS=Mus musculus GN=Ruvbl2 PE=2 SV=3 - [RUVB2_MOUSE]	2112,19	50,54	1	18	18	62	463	51,1	5,64
P31230	Aminoacyl tRNA synthase complex-interacting multifunctional protein 1 OS=Mus musculus GN=Aimp1 PE=1 SV=2 - [AIMP1_MOUSE]	2104,69	43,55	1	10	10	99	310	34,0	8,35
O35326	Serine/arginine-rich splicing factor 5 OS=Mus musculus GN=Srsf5 PE=1 SV=2 - [SRSF5_MOUSE]	2100,09	25,28	1	7	8	89	269	30,9	11,56
Q3TTY5	Keratin, type II cytoskeletal 2 epidermal OS=Mus musculus GN=Krt2 PE=1 SV=1 - [K22E_MOUSE]	2089,86	6,93	1	4	7	68	707	70,9	8,06
P24668	Cation-dependent mannose-6-phosphate receptor OS=Mus musculus GN=M6pr PE=1 SV=1 - [MPRD_MOUSE]	2087,86	21,94	1	4	4	55	278	31,2	5,39
Q6A068	Cell division cycle 5-related protein OS=Mus musculus GN=Cdc5l PE=1 SV=2 - [CDC5L_MOUSE]	2065,61	42,52	1	22	23	83	802	92,1	8,02
Q569Z5	Probable ATP-dependent RNA helicase DDX46 OS=Mus musculus GN=Ddx46 PE=1 SV=2 - [DDX46_MOUSE]	2063,12	22,38	1	23	24	79	1032	117,4	9,26
Q7TPH6	Probable E3 ubiquitin-protein ligase MYCBP2 OS=Mus musculus GN=Mycbp2 PE=1 SV=2 - [MYCB2_MOUSE]	2061,19	7,60	1	27	27	68	4711	517,4	7,12
Q6NV83	U2 snRNP-associated SURP motif-containing protein OS=Mus musculus GN=U2surp PE=1 SV=3 - [SR140_MOUSE]	2059,30	17,40	1	15	15	135	1029	118,2	8,47
Q91YR7	Pre-mRNA-processing factor 6 OS=Mus musculus GN=Prpf6 PE=2 SV=1 - [PRP6_MOUSE]	2049,53	12,54	1	10	10	72	941	106,7	8,09
Q61881	DNA replication licensing factor MCM7 OS=Mus musculus GN=Mcm7 PE=2 SV=1 - [MCM7_MOUSE]	2047,23	49,51	1	27	27	80	719	81,2	6,37
Q3UPF5	Zinc finger CCCH-type antiviral protein 1 OS=Mus musculus GN=Zc3hav1 PE=1 SV=1 - [ZCCHV_MOUSE]	2045,91	10,68	1	7	7	73	946	106,6	8,27
Q03265	ATP synthase subunit alpha, mitochondrial OS=Mus musculus GN=Atp5a1 PE=1 SV=1 - [ATPA_MOUSE]	2033,97	40,69	1	18	19	52	553	59,7	9,19
Q99KP6	Pre-mRNA-processing factor 19 OS=Mus musculus GN=Prpf19 PE=2 SV=1 - [PRP19_MOUSE]	2024,54	44,84	1	12	12	85	504	55,2	6,61
Q6P5D8	Structural maintenance of chromosomes flexible hinge domain-containing protein 1 OS=Mus musculus GN=Smchd1 PE=2 SV=2 - [SMHD1_	2021,62	16,94	1	26	26	98	2007	225,5	7,24
Q9Z2W0	Aspartyl aminopeptidase OS=Mus musculus GN=Dnpep PE=2 SV=2 - [DNPEP_MOUSE]	2014,68	29,18	1	11	11	77	473	52,2	7,25
Q8BL97	Serine/arginine-rich splicing factor 7 OS=Mus musculus GN=Srsf7 PE=1 SV=1 - [SRSF7_MOUSE]	2011,13	21,72	1	6	7	77	267	30,8	11,90
P61358	60S ribosomal protein L27 OS=Mus musculus GN=Rpl27 PE=2 SV=2 - [RL27_MOUSE]	2006,05	64,71	1	11	11	161	136	15,8	10,56
Q8BK67	Protein RCC2 OS=Mus musculus GN=Rcc2 PE=2 SV=1 - [RCC2_MOUSE]	1978,01	34,81	1	15	15	143	520	55,9	8,72
O55029	Coatomer subunit beta' OS=Mus musculus GN=Copb2 PE=2 SV=2 - [COPB2_MOUSE]	1976,04	31,05	1	20	20	85	905	102,4	5,30
Q60972	Histone-binding protein RBBP4 OS=Mus musculus GN=Rbbp4 PE=1 SV=5 - [RBBP4_MOUSE]	1969,40	28,94	1	9	9	73	425	47,6	4,89
Q8BX17	Gem-associated protein 5 OS=Mus musculus GN=Gemin5 PE=1 SV=2 - [GEMI5_MOUSE]	1939,00	16,44	1	20	20	117	1502	166,5	6,71
P61514	60S ribosomal protein L37a OS=Mus musculus GN=Rpl37a PE=2 SV=2 - [RL37A_MOUSE]	1935,00	40,22	1	3	3	35	92	10,3	10,43
O70194	Eukaryotic translation initiation factor 3 subunit D OS=Mus musculus GN=Eif3d PE=1 SV=2 - [EIF3D_MOUSE]	1932,18	25,91	1	13	13	75	548	63,9	6,05
P26516	26S proteasome non-ATPase regulatory subunit 7 OS=Mus musculus GN=Psmd7 PE=1 SV=2 - [PSD7_MOUSE]	1931,41	43,30	1	11	11	69	321	36,5	6,77
Q8BMF4	Dihydrolipoyllysine-residue acetyltransferase component of pyruvate dehydrogenase complex, mitochondrial OS=Mus musculus GN=Dlat	1922,54	21,18	1	9	9	56	642	67,9	8,57
Q99JI4	26S proteasome non-ATPase regulatory subunit 6 OS=Mus musculus GN=Psmd6 PE=2 SV=1 - [PSMD6_MOUSE]	1918,83	64,27	1	28	28	86	389	45,5	5,52
Q4VA53	Sister chromatid cohesion protein PDS5 homolog B OS=Mus musculus GN=Pds5b PE=1 SV=1 - [PDS5B_MOUSE]	1916,49	10,24	1	9	10	56	1446	164,3	8,50
Q8R323	Replication factor C subunit 3 OS=Mus musculus GN=Rfc3 PE=2 SV=1 - [RFC3_MOUSE]	1900,76	36,24	1	12	12	54	356	40,5	8,38
P97449	Aminopeptidase N OS=Mus musculus GN=Anpep PE=1 SV=4 - [AMPN_MOUSE]	1897,85	14,18	1	10	10	51	966	109,6	5,90
Q61735	Leukocyte surface antigen CD47 OS=Mus musculus GN=Cd47 PE=1 SV=2 - [CD47_MOUSE]	1896,51	8,91	1	2	2	36	303	33,1	8,63
Q8C0C7	Phenylalanyl-tRNA synthetase alpha chain OS=Mus musculus GN=Farsa PE=2 SV=1 - [SYFA_MOUSE]	1888,98	34,06	1	14	14	63	508	57,6	8,28
P09528	Ferritin heavy chain OS=Mus musculus GN=Fth1 PE=1 SV=2 - [FRIH_MOUSE]	1885,38	46,15	1	9	9	85	182	21,1	5,88
Q6PDM2	Serine/arginine-rich splicing factor 1 OS=Mus musculus GN=Srsf1 PE=1 SV=3 - [SRSF1_MOUSE]	1883,11	51,61	1	15	16	99	248	27,7	10,36
Q6PAM1	Alpha-taxilin OS=Mus musculus GN=Txlna PE=2 SV=1 - [TXLNA_MOUSE]	1882,61	13,72	1	6	6	46	554	62,3	6,74
P62843	40S ribosomal protein S15 OS=Mus musculus GN=Rps15 PE=2 SV=2 - [RS15_MOUSE]	1881,65	43,45	1	5	5	99	145	17,0	10,39
P17426	AP-2 complex subunit alpha-1 OS=Mus musculus GN=Ap2a1 PE=1 SV=1 - [AP2A1_MOUSE]	1880,99	19,45	1	13	17	90	977	107,6	7,03
Q9WVJ2	26S proteasome non-ATPase regulatory subunit 13 OS=Mus musculus GN=Psmd13 PE=1 SV=1 - [PSD13_MOUSE]	1879,64	49,20	1	17	18	109	376	42,8	5,71
P14434	H-2 class II histocompatibility antigen, A-B alpha chain OS=Mus musculus GN=H2-Aa PE=1 SV=2 - [HA2B_MOUSE]	1878,34	21,48	1	3	3	44	256	28,1	4,78
Q99K48	Non-POU domain-containing octamer-binding protein OS=Mus musculus GN=Nono PE=1 SV=3 - [NONO_MOUSE]	1861,28	20,51	1	7	8	86	473	54,5	8,95
Q99LE6	ATP-binding cassette sub-family F member 2 OS=Mus musculus GN=Abcf2 PE=2 SV=1 - [ABCF2_MOUSE]	1840,74	26,59	1	14	14	91	628	71,7	7,05
O35643	AP-1 complex subunit beta-1 OS=Mus musculus GN=Ap1b1 PE=1 SV=2 - [AP1B1_MOUSE]	1835,65	22,91	1	8	17	62	943	103,9	5,17
Q3UJB9	Enhancer of mRNA-decapping protein 4 OS=Mus musculus GN=Edc4 PE=1 SV=2 - [EDC4_MOUSE]	1834,00	15,08	1	15	15	63	1406	152,4	5,78
P57784	U2 small nuclear ribonucleoprotein A' OS=Mus musculus GN=Snrpa1 PE=1 SV=2 - [RU2A_MOUSE]	1818,65	44,31	1	15	15	50	255	28,3	8,62
P61255	60S ribosomal protein L26 OS=Mus musculus GN=Rpl26 PE=2 SV=1 - [RL26_MOUSE]	1815,74	52,41	1	15	15	125	145	17,2	10,55
Q3UYV9	Nuclear cap-binding protein subunit 1 OS=Mus musculus GN=Ncbp1 PE=1 SV=2 - [NCBP1_MOUSE]	1813,97	22,66	1	14	14	58	790	91,9	6,64
O09106	Histone deacetylase 1 OS=Mus musculus GN=Hdac1 PE=1 SV=1 - [HDAC1_MOUSE]	1805,68	20,75	1	5	8	91	482	55,0	5,48
G5E870	E3 ubiquitin-protein ligase TRIP12 OS=Mus musculus GN=Trip12 PE=1 SV=1 - [TRIPC_MOUSE]	1801,09	7,70	1	11	11	41	2025	224,0	8,35
Q5XJY5	Coatomer subunit delta OS=Mus musculus GN=Arcn1 PE=2 SV=2 - [COPD_MOUSE]	1797,33	36,79	1	15	15	72	511	57,2	6,21
Q6IFX2	Keratin, type I cytoskeletal 42 OS=Mus musculus GN=Krt42 PE=1 SV=1 - [K1C42_MOUSE]	1791,94	32,96	1	6	15	69	452	50,1	5,16
P27773	Protein disulfide-isomerase A3 OS=Mus musculus GN=Pdia3 PE=1 SV=2 - [PDIA3_MOUSE]	1788,61	43,76	1	20	20	70	505	56,6	6,21
Q52KI8	Serine/arginine repetitive matrix protein 1 OS=Mus musculus GN=Srrm1 PE=1 SV=2 - [SRRM1_MOUSE]	1786,21	15,86	1	13	13	76	946	106,8	11,87
Q9QXK3	Coatomer subunit gamma-2 OS=Mus musculus GN=Copg2 PE=2 SV=1 - [COPG2_MOUSE]	1782,09	8,38	1	5	6	47	871	97,6	5,80
Q9Z2U1	Proteasome subunit alpha type-5 OS=Mus musculus GN=Psma5 PE=1 SV=1 - [PSA5_MOUSE]	1781,96	46,06	1	10	10	67	241	26,4	4,79
Q7TPD0	Integrator complex subunit 3 OS=Mus musculus GN=Ints3 PE=1 SV=2 - [INT3_MOUSE]	1779,24	6,44	1	5	5	36	1041	117,9	5,80
Q99L45	Eukaryotic translation initiation factor 2 subunit 2 OS=Mus musculus GN=Eif2s2 PE=1 SV=1 - [IF2B_MOUSE]	1776,60	48,64	1	15	15	88	331	38,1	5,80
P17427	AP-2 complex subunit alpha-2 OS=Mus musculus GN=Ap2a2 PE=1 SV=2 - [AP2A2_MOUSE]	1761,03	23,24	1	13	18	74	938	104,0	6,93
P60229	Eukaryotic translation initiation factor 3 subunit E OS=Mus musculus GN=Eif3e PE=1 SV=1 - [EIF3E_MOUSE]	1751,04	46,29	1	19	19	75	445	52,2	6,04
Q8VDN2	Sodium/potassium-transporting ATPase subunit alpha-1 OS=Mus musculus GN=Atp1a1 PE=1 SV=1 - [AT1A1_MOUSE]	1732,68	14,76	1	11	11	56	1023	112,9	5,45
P08775	DNA-directed RNA polymerase II subunit RPB1 OS=Mus musculus GN=Polr2a PE=1 SV=3 - [RPB1_MOUSE]	1729,21	10,86	1	15	15	58	1970	217,0	7,37
P63154	Crooked neck-like protein 1 OS=Mus musculus GN=Crnkl1 PE=2 SV=1 - [CRNL1_MOUSE]	1727,76	29,57	1	19	19	51	690	83,4	6,93
Q9Z2C8	Y-box-binding protein 2 OS=Mus musculus GN=Ybx2 PE=1 SV=1 - [YBOX2_MOUSE]	1708,65	27,22	1	3	6	50	360	38,2	10,95
Q8C2K5	RAS protein activator like-3 OS=Mus musculus GN=Rasal3 PE=2 SV=1 - [RASL3_MOUSE]	1708,33	20,08	1	14	14	69	1041	114,7	8,98
Q9WUA2	Phenylalanyl-tRNA synthetase beta chain OS=Mus musculus GN=Farsb PE=2 SV=2 - [SYFB_MOUSE]	1700,89	30,05	1	15	15	87	589	65,7	7,12
Q64012	RNA-binding protein Raly OS=Mus musculus GN=Raly PE=1 SV=3 - [RALY_MOUSE]	1693,01	41,35	1	11	12	83	312	33,2	8,84
Q91VC3	Eukaryotic initiation factor 4A-III OS=Mus musculus GN=Eif4a3 PE=1 SV=3 - [IF4A3_MOUSE]	1692,62	32,12	1	13	14	72	411	46,8	6,73
P50516	V-type proton ATPase catalytic subunit A OS=Mus musculus GN=Atp6v1a PE=1 SV=2 - [VATA_MOUSE]	1692,31	39,38	1	18	19	67	617	68,3	5,58
P26369	Splicing factor U2AF 65 kDa subunit OS=Mus musculus GN=U2af2 PE=1 SV=3 - [U2AF2_MOUSE]	1690,12	27,16	1	8	8	72	475	53,5	9,09
P23249	Putative helicase MOV-10 OS=Mus musculus GN=Mov10 PE=1 SV=2 - [MOV10_MOUSE]	1687,07	19,22	1	15	15	61	1004	113,5	9,01
Q99MR6	Serrate RNA effector molecule homolog OS=Mus musculus GN=Srrt PE=1 SV=1 - [SRRT_MOUSE]	1668,95	7,09	1	7	7	50	875	100,4	5,97
P97310	DNA replication licensing factor MCM2 OS=Mus musculus GN=Mcm2 PE=1 SV=3 - [MCM2_MOUSE]	1663,67	27,10	1	17	17	57	904	102,0	5,72
Q61210	Rho guanine nucleotide exchange factor 1 OS=Mus musculus GN=Arhgef1 PE=1 SV=2 - [ARHG1_MOUSE]	1663,48	30,76	1	22	22	77	920	102,7	5,60
Q8VIJ6	Splicing factor, proline- and glutamine-rich OS=Mus musculus GN=Sfpq PE=1 SV=1 - [SFPQ_MOUSE]	1660,61	29,76	1	14	15	93	699	75,4	9,44
Q99KN9	Clathrin interactor 1 OS=Mus musculus GN=Clint1 PE=1 SV=2 - [EPN4_MOUSE]	1658,71	22,66	1	11	11	71	631	68,5	6,25
Q9QZE5	Coatomer subunit gamma OS=Mus musculus GN=Copg PE=2 SV=1 - [COPG_MOUSE]	1648,58	17,73	1	10	11	44	874	97,5	5,35
P25206	DNA replication licensing factor MCM3 OS=Mus musculus GN=Mcm3 PE=1 SV=2 - [MCM3_MOUSE]	1628,97	30,91	1	17	18	69	812	91,5	5,55
Q3UMY5	Echinoderm microtubule-associated protein-like 4 OS=Mus musculus GN=Eml4 PE=1 SV=1 - [EMAL4_MOUSE]	1625,04	12,75	1	11	11	59	988	110,0	6,54
P43275	Histone H1.1 OS=Mus musculus GN=Hist1h1a PE=2 SV=2 - [H11_MOUSE]	1614,59	26,76	1	6	8	60	213	21,8	10,93
O35609	Secretory carrier-associated membrane protein 3 OS=Mus musculus GN=Scamp3 PE=1 SV=3 - [SCAM3_MOUSE]	1594,96	14,90	1	3	3	26	349	38,4	7,64
P07356	Annexin A2 OS=Mus musculus GN=Anxa2 PE=1 SV=2 - [ANXA2_MOUSE]	1592,88	44,84	1	16	16	57	339	38,7	7,69
P01899	H-2 class I histocompatibility antigen, D-B alpha chain OS=Mus musculus GN=H2-D1 PE=1 SV=2 - [HA11_MOUSE]	1586,41	34,81	1	8	11	77	362	40,8	6,73
Q9DC51	Guanine nucleotide-binding protein G(k) subunit alpha OS=Mus musculus GN=Gnai3 PE=1 SV=3 - [GNAI3_MOUSE]	1579,82	19,49	1	2	5	61	354	40,5	5,69
P62900	60S ribosomal protein L31 OS=Mus musculus GN=Rpl31 PE=2 SV=1 - [RL31_MOUSE]	1576,12	50,40	1	8	8	72	125	14,5	10,54
Q6NZF1	Zinc finger CCCH domain-containing protein 11A OS=Mus musculus GN=Zc3h11a PE=1 SV=1 - [ZC11A_MOUSE]	1574,78	16,79	1	9	9	56	792	86,4	8,13
Q9JLI8	Squamous cell carcinoma antigen recognized by T-cells 3 OS=Mus musculus GN=Sart3 PE=2 SV=1 - [SART3_MOUSE]	1573,61	22,66	1	19	19	56	962	109,5	5,24
Q9CXY6	Interleukin enhancer-binding factor 2 OS=Mus musculus GN=Ilf2 PE=1 SV=1 - [ILF2_MOUSE]	1566,21	40,51	1	11	12	52	390	43,0	5,26
Q62351	Transferrin receptor protein 1 OS=Mus musculus GN=Tfrc PE=1 SV=1 - [TFR1_MOUSE]	1559,11	14,42	1	9	9	41	763	85,7	6,57
Q99JF8	PC4 and SFRS1-interacting protein OS=Mus musculus GN=Psip1 PE=1 SV=1 - [PSIP1_MOUSE]	1554,11	24,24	1	10	10	47	528	59,7	9,13
Q91WK2	Eukaryotic translation initiation factor 3 subunit H OS=Mus musculus GN=Eif3h PE=1 SV=1 - [EIF3H_MOUSE]	1551,30	52,84	1	15	15	71	352	39,8	6,67
P43276	Histone H1.5 OS=Mus musculus GN=Hist1h1b PE=1 SV=2 - [H15_MOUSE]	1535,03	28,25	1	8	9	62	223	22,6	10,92
P41105	60S ribosomal protein L28 OS=Mus musculus GN=Rpl28 PE=1 SV=2 - [RL28_MOUSE]	1533,93	52,55	1	11	11	94	137	15,7	12,02
Q9DB77	Cytochrome b-c1 complex subunit 2, mitochondrial OS=Mus musculus GN=Uqcrc2 PE=1 SV=1 - [QCR2_MOUSE]	1524,39	39,51	1	13	13	46	453	48,2	9,25
Q80TP3	E3 ubiquitin-protein ligase UBR5 OS=Mus musculus GN=Ubr5 PE=1 SV=2 - [UBR5_MOUSE]	1521,58	7,59	1	18	18	68	2792	308,2	5,85
Q9CQF3	Cleavage and polyadenylation specificity factor subunit 5 OS=Mus musculus GN=Nudt21 PE=2 SV=1 - [CPSF5_MOUSE]	1505,94	45,37	1	10	10	54	227	26,2	8,82
Q571I9	Aldehyde dehydrogenase family 16 member A1 OS=Mus musculus GN=Aldh16a1 PE=2 SV=2 - [A16A1_MOUSE]	1505,18	15,96	1	10	10	38	802	84,7	6,32
Q05CL8	La-related protein 7 OS=Mus musculus GN=Larp7 PE=1 SV=2 - [LARP7_MOUSE]	1503,72	29,65	1	15	15	47	570	64,8	9,54
P63101	14-3-3 protein zeta/delta OS=Mus musculus GN=Ywhaz PE=1 SV=1 - [1433Z_MOUSE]	1490,58	33,47	1	6	9	38	245	27,8	4,79
Q9WTQ5	A-kinase anchor protein 12 OS=Mus musculus GN=Akap12 PE=1 SV=1 - [AKA12_MOUSE]	1490,26	7,72	1	8	8	29	1684	180,6	4,44
Q64282	Interferon-induced protein with tetratricopeptide repeats 1 OS=Mus musculus GN=Ifit1 PE=2 SV=2 - [IFIT1_MOUSE]	1486,46	34,34	1	15	15	48	463	53,7	7,52
Q05144	Ras-related C3 botulinum toxin substrate 2 OS=Mus musculus GN=Rac2 PE=2 SV=1 - [RAC2_MOUSE]	1482,16	11,98	1	2	3	76	192	21,4	7,61
P63276	40S ribosomal protein S17 OS=Mus musculus GN=Rps17 PE=1 SV=2 - [RS17_MOUSE]	1480,15	43,70	1	4	4	53	135	15,5	9,85
Q9R1P0	Proteasome subunit alpha type-4 OS=Mus musculus GN=Psma4 PE=1 SV=1 - [PSA4_MOUSE]	1478,51	35,25	1	9	9	60	261	29,5	7,72
O35593	26S proteasome non-ATPase regulatory subunit 14 OS=Mus musculus GN=Psmd14 PE=1 SV=2 - [PSDE_MOUSE]	1473,20	61,94	1	12	13	63	310	34,6	6,52

O35295	Transcriptional activator protein Pur-beta OS=Mus musculus GN=Purb PE=1 SV=3 - [PURB_MOUSE]	1467,56	36,11	1	8	9	45	324	33,9	5,43
Q9D7G0	Ribose-phosphate pyrophosphokinase 1 OS=Mus musculus GN=Prps1 PE=1 SV=4 - [PRPS1_MOUSE]	1465,99	24,84	1	2	8	59	318	34,8	6,98
Q08583	THO complex subunit 4 OS=Mus musculus GN=Thoc4 PE=1 SV=3 - [THOC4_MOUSE]	1455,94	31,37	1	5	5	46	255	26,9	11,15
Q08288	Cell growth-regulating nucleolar protein OS=Mus musculus GN=Lyar PE=1 SV=2 - [LYAR_MOUSE]	1454,17	23,71	1	7	7	67	388	43,7	9,50
Q9QZD9	Eukaryotic translation initiation factor 3 subunit I OS=Mus musculus GN=Eif3i PE=1 SV=1 - [EIF3I_MOUSE]	1444,12	50,46	1	13	13	65	325	36,4	5,64
A2AGT5	Cytoskeleton-associated protein 5 OS=Mus musculus GN=Ckap5 PE=2 SV=1 - [CKAP5_MOUSE]	1440,62	9,60	1	14	14	59	2032	225,5	7,96
P57776	Elongation factor 1-delta OS=Mus musculus GN=Eef1d PE=1 SV=3 - [EF1D_MOUSE]	1440,37	34,52	1	7	7	38	281	31,3	5,02
Q6PDI5	Proteasome-associated protein ECM29 homolog OS=Mus musculus GN=Ecm29 PE=1 SV=3 - [ECM29_MOUSE]	1433,20	13,70	1	19	19	63	1840	203,6	7,06
Q99JX4	Eukaryotic translation initiation factor 3 subunit M OS=Mus musculus GN=Eif3m PE=2 SV=1 - [EIF3M_MOUSE]	1429,63	22,73	1	7	7	51	374	42,5	5,74
P97789	5'-3' exoribonuclease 1 OS=Mus musculus GN=Xrn1 PE=1 SV=1 - [XRN1_MOUSE]	1429,00	9,08	1	11	11	62	1719	194,2	7,50
Q9CQE8	UPF0568 protein C14orf166 homolog OS=Mus musculus PE=2 SV=1 - [CN166_MOUSE]	1427,76	55,74	1	12	12	34	244	28,1	6,89
P70288	Histone deacetylase 2 OS=Mus musculus GN=Hdac2 PE=1 SV=1 - [HDAC2_MOUSE]	1423,53	14,96	1	2	5	68	488	55,3	5,91
P11835	Integrin beta-2 OS=Mus musculus GN=Itgb2 PE=2 SV=2 - [ITB2_MOUSE]	1419,97	18,16	1	11	11	52	771	85,0	7,12
Q61696	Heat shock 70 kDa protein 1A OS=Mus musculus GN=Hspa1a PE=1 SV=2 - [HS71A_MOUSE]	1417,29	18,88	2	6	9	52	641	70,0	5,72
P40124	Adenylyl cyclase-associated protein 1 OS=Mus musculus GN=Cap1 PE=1 SV=4 - [CAP1_MOUSE]	1404,45	37,97	1	12	12	51	474	51,5	7,52
Q8R0Y6	10-formyltetrahydrofolate dehydrogenase OS=Mus musculus GN=Aldh1l1 PE=2 SV=1 - [FTHFD_MOUSE]	1401,83	24,06	1	18	18	50	902	98,6	5,91
Q9DBD5	Proline-, glutamic acid- and leucine-rich protein 1 OS=Mus musculus GN=Pelp1 PE=1 SV=2 - [PELP1_MOUSE]	1401,11	10,06	1	8	8	59	1123	118,0	4,36
Q61510	E3 ubiquitin/ISG15 ligase TRIM25 OS=Mus musculus GN=Trim25 PE=2 SV=2 - [TRI25_MOUSE]	1400,83	21,45	1	14	14	64	634	71,7	8,28
E9PZJ8	Activating signal cointegrator 1 complex subunit 3 OS=Mus musculus GN=Ascc3 PE=2 SV=1 - [ASCC3_MOUSE]	1400,47	6,60	1	12	12	52	2198	250,4	7,02
Q3TLH4	Protein BAT2-like 2 OS=Mus musculus GN=Bat2l2 PE=1 SV=2 - [BA2L2_MOUSE]	1398,43	4,81	1	10	10	60	2828	308,7	8,97
Q9WUK4	Replication factor C subunit 2 OS=Mus musculus GN=Rfc2 PE=2 SV=1 - [RFC2_MOUSE]	1385,01	28,37	1	7	7	31	349	38,7	6,44
O55142	60S ribosomal protein L35a OS=Mus musculus GN=Rpl35a PE=2 SV=2 - [RL35A_MOUSE]	1367,36	48,18	1	9	9	127	110	12,5	10,89
Q6P4S8	Integrator complex subunit 1 OS=Mus musculus GN=Ints1 PE=1 SV=2 - [INT1_MOUSE]	1352,79	7,29	1	14	14	90	2195	245,0	6,28
Q8CCF0	U4/U6 small nuclear ribonucleoprotein Prp31 OS=Mus musculus GN=Prpf31 PE=2 SV=3 - [PRP31_MOUSE]	1350,40	34,07	1	11	11	42	499	55,4	5,67
Q9CX86	Heterogeneous nuclear ribonucleoprotein A0 OS=Mus musculus GN=Hnrnpa0 PE=1 SV=1 - [ROA0_MOUSE]	1347,80	26,23	1	6	7	45	305	30,5	9,31
Q7TMY8	E3 ubiquitin-protein ligase HUWE1 OS=Mus musculus GN=Huwe1 PE=1 SV=5 - [HUWE1_MOUSE]	1337,96	5,53	1	18	18	35	4377	482,3	5,22
P0C0S6	Histone H2A.Z OS=Mus musculus GN=H2afz PE=1 SV=2 - [H2AZ_MOUSE]	1337,22	31,25	2	3	5	63	128	13,5	10,58
A2A4P0	ATP-dependent RNA helicase DHX8 OS=Mus musculus GN=Dhx8 PE=2 SV=1 - [DHX8_MOUSE]	1337,19	14,07	1	12	13	48	1244	142,5	8,12
P14873	Microtubule-associated protein 1B OS=Mus musculus GN=Map1b PE=1 SV=2 - [MAP1B_MOUSE]	1336,81	11,49	1	18	18	49	2464	270,1	4,83
Q99ME9	Nucleolar GTP-binding protein 1 OS=Mus musculus GN=Gtpbp4 PE=2 SV=3 - [NOG1_MOUSE]	1325,99	20,82	1	11	11	68	634	74,1	9,52
Q6DFV1	Condensin-2 complex subunit G2 OS=Mus musculus GN=Ncapg2 PE=2 SV=2 - [CNDG2_MOUSE]	1325,84	8,88	1	9	9	55	1138	130,8	6,93
Q921G8	Gamma-tubulin complex component 2 OS=Mus musculus GN=Tubgcp2 PE=2 SV=2 - [GCP2_MOUSE]	1323,79	19,56	1	14	14	36	905	103,2	6,77
P62281	40S ribosomal protein S11 OS=Mus musculus GN=Rps11 PE=2 SV=3 - [RS11_MOUSE]	1321,39	50,00	1	11	11	92	158	18,4	10,30
P47955	60S acidic ribosomal protein P1 OS=Mus musculus GN=Rplp1 PE=1 SV=1 - [RLA1_MOUSE]	1309,92	28,95	1	2	2	45	114	11,5	4,32
P60122	RuvB-like 1 OS=Mus musculus GN=Ruvbl1 PE=1 SV=1 - [RUVB1_MOUSE]	1302,47	45,61	1	17	17	44	456	50,2	6,42
Q6ZWV7	60S ribosomal protein L35 OS=Mus musculus GN=Rpl35 PE=2 SV=1 - [RL35_MOUSE]	1285,80	30,08	1	5	5	55	123	14,5	11,05
P13020	Gelsolin OS=Mus musculus GN=Gsn PE=1 SV=3 - [GELS_MOUSE]	1283,84	27,05	1	14	15	51	780	85,9	6,18
Q3V3V9	Leucine-rich repeat-containing protein 16C OS=Mus musculus GN=Rltpr PE=2 SV=2 - [LR16C_MOUSE]	1277,74	10,96	1	11	11	51	1296	141,3	7,18
Q9D554	Splicing factor 3A subunit 3 OS=Mus musculus GN=Sf3a3 PE=2 SV=2 - [SF3A3_MOUSE]	1263,07	24,35	1	11	11	49	501	58,8	5,34
P62911	60S ribosomal protein L32 OS=Mus musculus GN=Rpl32 PE=2 SV=2 - [RL32_MOUSE]	1261,58	42,22	1	8	8	56	135	15,8	11,33
Q8CFQ3	Intron-binding protein aquarius OS=Mus musculus GN=Aqr PE=2 SV=2 - [AQR_MOUSE]	1254,63	21,81	1	26	26	75	1481	170,2	6,27
Q60875	Rho guanine nucleotide exchange factor 2 OS=Mus musculus GN=Arhgef2 PE=1 SV=4 - [ARHG2_MOUSE]	1254,35	18,98	1	15	15	62	985	111,9	7,25
P40142	Transketolase OS=Mus musculus GN=Tkt PE=1 SV=1 - [TKT_MOUSE]	1253,45	35,15	1	16	16	48	623	67,6	7,50
P54775	26S protease regulatory subunit 6B OS=Mus musculus GN=Psmc4 PE=1 SV=2 - [PRS6B_MOUSE]	1245,05	38,28	1	13	13	76	418	47,4	5,21
P68040	Guanine nucleotide-binding protein subunit beta-2-like 1 OS=Mus musculus GN=Gnb2l1 PE=1 SV=3 - [GBLP_MOUSE]	1234,87	23,34	1	7	7	46	317	35,1	7,69
P63094	Guanine nucleotide-binding protein G(s) subunit alpha isoforms short OS=Mus musculus GN=Gnas PE=1 SV=1 - [GNAS2_MOUSE]	1222,34	9,39	2	2	3	53	394	45,6	5,96
P02088	Hemoglobin subunit beta-1 OS=Mus musculus GN=Hbb-b1 PE=1 SV=2 - [HBB1_MOUSE]	1216,62	55,78	1	7	7	38	147	15,8	7,65
P35601	Replication factor C subunit 1 OS=Mus musculus GN=Rfc1 PE=1 SV=2 - [RFC1_MOUSE]	1215,95	11,14	1	9	9	64	1131	125,9	9,33
Q921F2	TAR DNA-binding protein 43 OS=Mus musculus GN=Tardbp PE=1 SV=1 - [TADBP_MOUSE]	1214,82	21,01	1	7	7	34	414	44,5	6,70
Q920B9	FACT complex subunit SPT16 OS=Mus musculus GN=Supt16h PE=1 SV=2 - [SP16H_MOUSE]	1214,71	18,72	1	14	14	50	1047	119,7	5,66
Q80UP3	Diacylglycerol kinase zeta OS=Mus musculus GN=Dgkz PE=1 SV=2 - [DGKZ_MOUSE]	1212,85	13,67	1	9	9	52	929	104,0	8,09
Q923D4	Splicing factor 3B subunit 5 OS=Mus musculus GN=Sf3b5 PE=2 SV=1 - [SF3B5_MOUSE]	1211,94	26,74	1	2	2	41	86	10,1	6,35
Q8C147	Dedicator of cytokinesis protein 8 OS=Mus musculus GN=Dock8 PE=2 SV=4 - [DOCK8_MOUSE]	1210,75	7,10	1	12	14	69	2100	238,8	6,96
Q3TKT4	Transcription activator BRG1 OS=Mus musculus GN=Smarca4 PE=1 SV=1 - [SMCA4_MOUSE]	1207,95	12,77	1	16	16	50	1613	181,3	8,00
Q91YP3	Putative deoxyribose-phosphate aldolase OS=Mus musculus GN=Dera PE=2 SV=1 - [DEOC_MOUSE]	1199,81	42,45	1	10	10	29	318	35,0	8,72
Q91WN1	DnaJ homolog subfamily C member 9 OS=Mus musculus GN=Dnajc9 PE=2 SV=2 - [DNJC9_MOUSE]	1198,49	40,54	1	12	12	46	259	30,0	5,94
Q5SQX6	Cytoplasmic FMR1-interacting protein 2 OS=Mus musculus GN=Cyfip2 PE=1 SV=2 - [CYFP2_MOUSE]	1197,76	10,06	1	10	11	46	1253	145,6	7,05
O35682	Myeloid-associated differentiation marker OS=Mus musculus GN=Myadm PE=2 SV=2 - [MYADM_MOUSE]	1192,91	7,19	1	2	2	19	320	35,3	8,31
P60843	Eukaryotic initiation factor 4A-I OS=Mus musculus GN=Eif4a1 PE=2 SV=1 - [IF4A1_MOUSE]	1190,18	28,33	1	8	9	37	406	46,1	5,48
Q61545	RNA-binding protein EWS OS=Mus musculus GN=Ewsr1 PE=1 SV=2 - [EWS_MOUSE]	1188,71	8,09	1	3	4	41	655	68,4	9,33
Q9D0F6	Replication factor C subunit 5 OS=Mus musculus GN=Rfc5 PE=2 SV=1 - [RFC5_MOUSE]	1182,83	39,23	1	9	9	30	339	38,1	7,81
Q3TJZ6	Protein FAM98A OS=Mus musculus GN=Fam98a PE=2 SV=1 - [FA98A_MOUSE]	1175,24	12,43	1	3	4	22	515	55,0	8,95
Q9WVR4	Fragile X mental retardation syndrome-related protein 2 OS=Mus musculus GN=Fxr2 PE=1 SV=1 - [FXR2_MOUSE]	1171,10	18,28	1	6	8	64	673	73,7	6,23
P97379	Ras GTPase-activating protein-binding protein 2 OS=Mus musculus GN=G3bp2 PE=1 SV=2 - [G3BP2_MOUSE]	1170,67	27,59	1	9	10	72	482	54,1	5,62
Q8CFI7	DNA-directed RNA polymerase II subunit RPB2 OS=Mus musculus GN=Polr2b PE=2 SV=2 - [RPB2_MOUSE]	1168,19	14,74	1	13	13	62	1174	133,8	6,87
Q80VD1	Protein FAM98B OS=Mus musculus GN=Fam98b PE=2 SV=1 - [FA98B_MOUSE]	1167,84	15,15	1	4	5	38	429	45,3	8,50
Q9CPN8	Insulin-like growth factor 2 mRNA-binding protein 3 OS=Mus musculus GN=Igf2bp3 PE=1 SV=1 - [IF2B3_MOUSE]	1162,35	24,87	1	10	12	44	579	63,5	8,87
P70398	Probable ubiquitin carboxyl-terminal hydrolase FAF-X OS=Mus musculus GN=Usp9x PE=1 SV=2 - [USP9X_MOUSE]	1161,26	7,27	1	16	16	42	2559	290,5	5,87
O55234	Proteasome subunit beta type-5 OS=Mus musculus GN=Psmb5 PE=1 SV=3 - [PSB5_MOUSE]	1157,91	27,27	1	7	7	34	264	28,5	7,02
P24369	Peptidyl-prolyl cis-trans isomerase B OS=Mus musculus GN=Ppib PE=2 SV=2 - [PPIB_MOUSE]	1153,97	51,39	1	9	9	38	216	23,7	9,55
Q9DCE5	p21-activated protein kinase-interacting protein 1 OS=Mus musculus GN=Pak1ip1 PE=2 SV=2 - [PK1IP_MOUSE]	1152,58	22,51	1	7	7	37	382	42,1	8,47
O35226	26S proteasome non-ATPase regulatory subunit 4 OS=Mus musculus GN=Psmd4 PE=1 SV=1 - [PSMD4_MOUSE]	1148,57	22,34	1	7	7	31	376	40,7	4,79
Q3TEA8	Heterochromatin protein 1-binding protein 3 OS=Mus musculus GN=Hp1bp3 PE=1 SV=1 - [HP1B3_MOUSE]	1146,13	27,98	1	13	13	41	554	60,8	9,70
O70551	Serine/threonine-protein kinase SRPK1 OS=Mus musculus GN=Srpk1 PE=1 SV=2 - [SRPK1_MOUSE]	1142,02	29,78	1	13	15	47	648	73,0	6,19
Q9Z1Z2	Serine-threonine kinase receptor-associated protein OS=Mus musculus GN=Strap PE=1 SV=2 - [STRAP_MOUSE]	1137,17	30,86	1	8	8	23	350	38,4	5,12
Q6DFW4	Nucleolar protein 58 OS=Mus musculus GN=Nop58 PE=1 SV=1 - [NOP58_MOUSE]	1134,34	19,03	1	7	7	28	536	60,3	8,34
O08638	Myosin-11 OS=Mus musculus GN=Myh11 PE=1 SV=1 - [MYH11_MOUSE]	1133,93	4,67	1	2	8	37	1972	226,9	5,45
P62983	Ubiquitin-40S ribosomal protein S27a OS=Mus musculus GN=Rps27a PE=1 SV=2 - [RS27A_MOUSE]	1131,41	39,74	1	7	7	70	156	17,9	9,64
P28740	Kinesin-like protein KIF2A OS=Mus musculus GN=Kif2a PE=1 SV=2 - [KIF2A_MOUSE]	1128,21	16,60	1	11	11	44	705	79,7	6,73
Q8VDM6	Heterogeneous nuclear ribonucleoprotein U-like protein 1 OS=Mus musculus GN=Hnrnpul1 PE=1 SV=1 - [HNRL1_MOUSE]	1127,30	13,85	1	8	8	53	859	95,9	6,58
Q9D8N0	Elongation factor 1-gamma OS=Mus musculus GN=Eef1g PE=1 SV=3 - [EF1G_MOUSE]	1124,54	14,19	1	7	7	38	437	50,0	6,74
Q3UZ39	Leucine-rich repeat flightless-interacting protein 1 OS=Mus musculus GN=Lrrfip1 PE=1 SV=2 - [LRRF1_MOUSE]	1121,57	29,08	1	12	12	35	729	79,2	4,82
O35955	Proteasome subunit beta type-10 OS=Mus musculus GN=Psmb10 PE=1 SV=1 - [PSB10_MOUSE]	1120,87	41,76	1	8	8	45	273	29,0	6,87
Q05512	Serine/threonine-protein kinase MARK2 OS=Mus musculus GN=Mark2 PE=1 SV=3 - [MARK2_MOUSE]	1103,34	19,85	1	11	12	50	776	86,3	9,67
P62962	Profilin-1 OS=Mus musculus GN=Pfn1 PE=1 SV=2 - [PROF1_MOUSE]	1099,94	59,29	1	7	7	30	140	14,9	8,28
Q80WQ2	Protein VAC14 homolog OS=Mus musculus GN=Vac14 PE=1 SV=1 - [VAC14_MOUSE]	1098,22	18,54	1	12	12	40	782	88,0	6,13
Q8QZY9	Splicing factor 3B subunit 4 OS=Mus musculus GN=Sf3b4 PE=2 SV=1 - [SF3B4_MOUSE]	1090,44	18,40	1	4	4	26	424	44,3	8,56
Q6ZQ58	La-related protein 1 OS=Mus musculus GN=Larp1 PE=1 SV=2 - [LARP1_MOUSE]	1087,40	17,72	1	13	13	84	1072	121,1	8,79
Q8CCP0	Serologically defined colon cancer antigen 1 homolog OS=Mus musculus GN=Sdccag1 PE=1 SV=2 - [SDCG1_MOUSE]	1083,79	13,06	1	12	12	35	1064	121,1	6,80
Q9WVA3	Mitotic checkpoint protein BUB3 OS=Mus musculus GN=Bub3 PE=2 SV=2 - [BUB3_MOUSE]	1082,84	34,97	1	9	10	38	326	36,9	6,84
Q9JMH9	Myosin-XVIIIa OS=Mus musculus GN=Myo18a PE=1 SV=2 - [MY18A_MOUSE]	1081,25	11,27	1	17	17	47	2050	232,6	6,28
P27048	Small nuclear ribonucleoprotein-associated protein B OS=Mus musculus GN=Snrpb PE=1 SV=1 - [RSMB_MOUSE]	1080,18	25,54	1	7	7	41	231	23,6	10,90
Q80SY5	Pre-mRNA-splicing factor 38B OS=Mus musculus GN=Prpf38b PE=1 SV=1 - [PR38B_MOUSE]	1077,98	20,30	1	11	11	62	542	63,7	10,54
Q6PDG5	SWI/SNF complex subunit SMARCC2 OS=Mus musculus GN=Smarcc2 PE=1 SV=2 - [SMRC2_MOUSE]	1076,37	15,17	1	9	15	45	1213	132,5	5,59
P97311	DNA replication licensing factor MCM6 OS=Mus musculus GN=Mcm6 PE=1 SV=1 - [MCM6_MOUSE]	1075,83	22,78	1	14	14	32	821	92,8	5,45
Q8BRT1	CLIP-associating protein 2 OS=Mus musculus GN=Clasp2 PE=1 SV=1 - [CLAP2_MOUSE]	1072,32	7,23	1	6	7	24	1286	140,7	8,63
Q8C129	Leucyl-cystinyl aminopeptidase OS=Mus musculus GN=Lnpep PE=1 SV=1 - [LCAP_MOUSE]	1071,53	12,20	1	10	10	43	1025	117,2	5,96
P70195	Proteasome subunit beta type-7 OS=Mus musculus GN=Psmb7 PE=1 SV=1 - [PSB7_MOUSE]	1070,31	27,44	1	9	9	44	277	29,9	7,99
A2AJI0	MAP7 domain-containing protein 1 OS=Mus musculus GN=Map7d1 PE=1 SV=1 - [MA7D1_MOUSE]	1055,21	13,48	1	8	8	33	846	93,2	10,15
Q3U1J4	DNA damage-binding protein 1 OS=Mus musculus GN=Ddb1 PE=1 SV=2 - [DDB1_MOUSE]	1055,09	19,65	1	17	17	50	1140	126,8	5,26
O89053	Coronin-1A OS=Mus musculus GN=Coro1a PE=1 SV=5 - [COR1A_MOUSE]	1054,13	8,24	1	4	4	38	461	51,0	6,48
Q91XV3	Brain acid soluble protein 1 OS=Mus musculus GN=Basp1 PE=1 SV=3 - [BASP1_MOUSE]	1046,62	49,12	1	6	6	32	226	22,1	4,51
Q9JJ28	Protein flightless-1 homolog OS=Mus musculus GN=Flii PE=1 SV=1 - [FLII_MOUSE]	1045,67	8,42	1	7	8	39	1271	144,7	6,06
P56960	Exosome component 10 OS=Mus musculus GN=Exosc10 PE=1 SV=2 - [EXOSX_MOUSE]	1045,57	14,66	1	11	11	35	887	100,9	8,29
O08553	Dihydropyrimidinase-related protein 2 OS=Mus musculus GN=Dpysl2 PE=1 SV=2 - [DPYL2_MOUSE]	1033,14	28,67	1	13	13	30	572	62,2	6,38
Q8K2Z4	Condensin complex subunit 1 OS=Mus musculus GN=Ncapd2 PE=1 SV=2 - [CND1_MOUSE]	1027,00	8,84	1	12	12	61	1392	155,6	6,38
P35278	Ras-related protein Rab-5C OS=Mus musculus GN=Rab5c PE=1 SV=2 - [RAB5C_MOUSE]	1024,09	33,33	1	3	6	38	216	23,4	8,41
Q62465	Synaptic vesicle membrane protein VAT-1 homolog OS=Mus musculus GN=Vat1 PE=1 SV=3 - [VAT1_MOUSE]	1021,76	16,01	1	5	5	33	406	43,1	6,37
P47964	60S ribosomal protein L36 OS=Mus musculus GN=Rpl36 PE=2 SV=2 - [RL36_MOUSE]	1016,10	11,43	1	2	2	67	105	12,2	11,34
Q99JX7	Nuclear RNA export factor 1 OS=Mus musculus GN=Nxf1 PE=1 SV=3 - [NXF1_MOUSE]	1011,03	27,83	1	14	15	37	618	70,3	8,73
Q5BLK4	Terminal uridylyltransferase 7 OS=Mus musculus GN=Zcchc6 PE=1 SV=3 - [TUT7_MOUSE]	1009,38	8,92	1	10	10	46	1491	169,0	6,65
Q60605	Myosin light polypeptide 6 OS=Mus musculus GN=Myl6 PE=1 SV=3 - [MYL6_MOUSE]	1001,97	50,99	1	7	7	39	151	16,9	4,65
P14106	Complement C1q subcomponent subunit B OS=Mus musculus GN=C1qb PE=1 SV=2 - [C1QB_MOUSE]	1000,46	20,95	1	4	4	31	253	26,7	8,15
Q91V41	Ras-related protein Rab-14 OS=Mus musculus GN=Rab14 PE=1 SV=3 - [RAB14_MOUSE]	996,53	50,23	1	7	8	39	215	23,9	6,21
O35522	Proteasome subunit beta type-9 OS=Mus musculus bactrianus GN=Psmb9 PE=1 SV=1 - [PSB9_MUSMB]	991,24	25,57	2	5	5	40	219	23,4	5,22
O88532	Zinc finger RNA-binding protein OS=Mus musculus GN=Zfr PE=1 SV=2 - [ZFR_MOUSE]	982,92	4,66	1	3	3	17	1074	116,8	9,04
Q99PU8	Putative ATP-dependent RNA helicase DHX30 OS=Mus musculus GN=Dhx30 PE=2 SV=1 - [DHX30_MOUSE]	982,19	13,80	1	15	15	48	1217	136,6	8,75
Q8BMS1	Trifunctional enzyme subunit alpha, mitochondrial OS=Mus musculus GN=Hadha PE=1 SV=1 - [ECHA_MOUSE]	980,24	20,31	1	12	12	36	763	82,6	9,14
Q99J62	Replication factor C subunit 4 OS=Mus musculus GN=Rfc4 PE=1 SV=1 - [RFC4_MOUSE]	978,77	32,69	1	11	11	31	364	39,8	6,70
Q8BK63	Casein kinase I isoform alpha OS=Mus musculus GN=Csnk1a1 PE=2 SV=2 - [KC1A_MOUSE]	965,08	37,69	1	10	10	38	337	38,9	9,57
Q9CX56	26S proteasome non-ATPase regulatory subunit 8 OS=Mus musculus GN=Psmd8 PE=2 SV=2 - [PSMD8_MOUSE]	963,01	30,03	1	9	9	47	353	39,9	9,58

Q7TNV0	Protein DEK OS=Mus musculus GN=Dek PE=1 SV=1 - [DEK_MOUSE]	959,81	28,42	1	8	8	35	380	43,1	6,86
P17742	Peptidyl-prolyl cis-trans isomerase A OS=Mus musculus GN=Ppia PE=1 SV=2 - [PPIA_MOUSE]	957,61	35,37	1	7	7	47	164	18,0	7,90
Q60692	Proteasome subunit beta type-6 OS=Mus musculus GN=Psmb6 PE=1 SV=3 - [PSB6_MOUSE]	955,44	16,81	1	4	4	27	238	25,4	5,11
P32233	Developmentally-regulated GTP-binding protein 1 OS=Mus musculus GN=Drg1 PE=1 SV=1 - [DRG1_MOUSE]	953,76	37,60	1	12	13	28	367	40,5	8,90
Q60767	Lymphocyte antigen 75 OS=Mus musculus GN=Ly75 PE=1 SV=2 - [LY75_MOUSE]	950,04	6,21	1	9	9	38	1723	197,2	6,39
P47915	60S ribosomal protein L29 OS=Mus musculus GN=Rpl29 PE=2 SV=2 - [RL29_MOUSE]	944,94	26,88	1	5	5	50	160	17,6	11,84
Q3UPL0	Protein transport protein Sec31A OS=Mus musculus GN=Sec31a PE=1 SV=2 - [SC31A_MOUSE]	943,66	11,46	1	10	11	45	1230	133,5	6,76
O89079	Coatomer subunit epsilon OS=Mus musculus GN=Cope PE=2 SV=3 - [COPE_MOUSE]	942,35	34,74	1	8	8	30	308	34,5	5,06
Q8BHD7	Regulator of differentiation 1 OS=Mus musculus GN=Rod1 PE=2 SV=1 - [ROD1_MOUSE]	937,02	23,14	1	5	7	47	523	56,7	9,13
Q6PGC1	ATP-dependent RNA helicase Dhx29 OS=Mus musculus GN=Dhx29 PE=2 SV=1 - [DHX29_MOUSE]	929,71	11,87	1	12	12	42	1365	153,9	7,94
Q9D0R4	Probable ATP-dependent RNA helicase DDX56 OS=Mus musculus GN=Ddx56 PE=2 SV=1 - [DDX56_MOUSE]	928,31	4,40	1	2	2	17	546	61,2	9,17
Q9EPU4	Cleavage and polyadenylation specificity factor subunit 1 OS=Mus musculus GN=Cpsf1 PE=1 SV=1 - [CPSF1_MOUSE]	926,90	13,46	1	17	17	51	1441	160,7	6,39
Q8K363	ATP-dependent RNA helicase DDX18 OS=Mus musculus GN=Ddx18 PE=2 SV=1 - [DDX18_MOUSE]	920,58	19,39	1	12	12	35	660	74,1	9,52
Q80X82	Symplekin OS=Mus musculus GN=Sympk PE=1 SV=1 - [SYMPK_MOUSE]	919,72	11,92	1	10	11	36	1284	142,2	6,05
D0QMC3	Myeloid cell nuclear differentiation antigen-like protein OS=Mus musculus GN=Mndal PE=2 SV=1 - [MNDAL_MOUSE]	913,71	30,11	1	12	12	36	538	60,5	9,28
Q9JHI7	Exosome complex exonuclease RRP45 OS=Mus musculus GN=Exosc9 PE=2 SV=1 - [EXOS9_MOUSE]	909,52	15,07	1	5	5	24	438	48,9	5,11
P62317	Small nuclear ribonucleoprotein Sm D2 OS=Mus musculus GN=Snrpd2 PE=2 SV=1 - [SMD2_MOUSE]	904,64	44,92	1	7	7	35	118	13,5	9,91
P35550	rRNA 2'-O-methyltransferase fibrillarin OS=Mus musculus GN=Fbl PE=1 SV=2 - [FBRL_MOUSE]	895,10	49,54	1	10	10	61	327	34,3	10,24
Q04750	DNA topoisomerase 1 OS=Mus musculus GN=Top1 PE=1 SV=2 - [TOP1_MOUSE]	893,81	15,65	1	10	10	59	767	90,8	9,33
Q9D1D4	Transmembrane emp24 domain-containing protein 10 OS=Mus musculus GN=Tmed10 PE=2 SV=1 - [TMEDA_MOUSE]	890,08	34,70	1	6	6	28	219	24,9	6,70
Q8VDJ3	Vigilin OS=Mus musculus GN=Hdlbp PE=1 SV=1 - [VIGLN_MOUSE]	888,17	14,51	1	14	14	29	1268	141,7	6,87
Q3TIV5	Zinc finger CCCH domain-containing protein 15 OS=Mus musculus GN=Zc3h15 PE=1 SV=2 - [ZC3HF_MOUSE]	886,62	17,37	1	5	6	21	426	48,3	5,30
Q9R0U0	Serine/arginine-rich splicing factor 10 OS=Mus musculus GN=Srsf10 PE=1 SV=2 - [SRS10_MOUSE]	874,35	30,92	1	8	8	47	262	31,3	11,27
P54823	Probable ATP-dependent RNA helicase DDX6 OS=Mus musculus GN=Ddx6 PE=2 SV=1 - [DDX6_MOUSE]	869,19	26,71	1	10	10	30	483	54,2	8,66
Q6PE01	U5 small nuclear ribonucleoprotein 40 kDa protein OS=Mus musculus GN=Snrnp40 PE=2 SV=1 - [SNR40_MOUSE]	862,88	40,78	1	9	9	34	358	39,3	8,10
P47757	F-actin-capping protein subunit beta OS=Mus musculus GN=Capzb PE=1 SV=3 - [CAPZB_MOUSE]	862,68	27,80	1	7	7	23	277	31,3	5,74
Q9D824	Pre-mRNA 3'-end-processing factor FIP1 OS=Mus musculus GN=Fip1l1 PE=1 SV=1 - [FIP1_MOUSE]	859,10	20,83	1	8	8	24	581	64,9	5,77
P24063	Integrin alpha-L OS=Mus musculus GN=Itgal PE=1 SV=2 - [ITAL_MOUSE]	857,52	9,03	1	9	9	36	1163	128,2	6,11
Q60932	Voltage-dependent anion-selective channel protein 1 OS=Mus musculus GN=Vdac1 PE=1 SV=3 - [VDAC1_MOUSE]	856,08	36,82	1	7	7	27	296	32,3	8,43
Q8BSP2	Condensin-2 complex subunit H2 OS=Mus musculus GN=Ncaph2 PE=1 SV=1 - [CNDH2_MOUSE]	854,13	9,72	1	5	5	50	607	68,9	4,67
P97461	40S ribosomal protein S5 OS=Mus musculus GN=Rps5 PE=2 SV=3 - [RS5_MOUSE]	851,09	14,22	1	2	2	27	204	22,9	9,72
Q3THE2	Myosin regulatory light chain 12B OS=Mus musculus GN=Myl12b PE=1 SV=2 - [ML12B_MOUSE]	848,41	40,12	1	7	7	21	172	19,8	4,84
Q8BMC4	Pumilio domain-containing protein C14orf21 homolog OS=Mus musculus PE=2 SV=1 - [CN021_MOUSE]	845,85	12,26	1	6	6	20	636	70,0	7,27
P70168	Importin subunit beta-1 OS=Mus musculus GN=Kpnb1 PE=1 SV=2 - [IMB1_MOUSE]	844,33	13,93	1	9	9	32	876	97,1	4,78
P47753	F-actin-capping protein subunit alpha-1 OS=Mus musculus GN=Capza1 PE=1 SV=4 - [CAZA1_MOUSE]	837,25	49,30	1	7	9	35	286	32,9	5,55
Q8R550	SH3 domain-containing kinase-binding protein 1 OS=Mus musculus GN=Sh3kbp1 PE=1 SV=1 - [SH3K1_MOUSE]	835,82	23,13	1	11	11	21	709	78,1	7,55
Q62376	U1 small nuclear ribonucleoprotein 70 kDa OS=Mus musculus GN=Snrnp70 PE=1 SV=2 - [RU17_MOUSE]	834,85	26,79	1	12	12	57	448	52,0	9,94
Q99LD9	Translation initiation factor eIF-2B subunit beta OS=Mus musculus GN=Eif2b2 PE=2 SV=1 - [EI2BB_MOUSE]	831,38	22,51	1	8	8	22	351	38,9	6,24
P16406	Glutamyl aminopeptidase OS=Mus musculus GN=Enpep PE=1 SV=1 - [AMPE_MOUSE]	830,68	21,27	1	17	17	51	945	107,9	5,44
P01901	H-2 class I histocompatibility antigen, K-B alpha chain OS=Mus musculus GN=H2-K1 PE=1 SV=1 - [HA1B_MOUSE]	826,97	17,62	1	3	6	44	369	41,3	6,39
Q9CPT5	Nucleolar protein 16 OS=Mus musculus GN=Nop16 PE=2 SV=1 - [NOP16_MOUSE]	816,28	52,81	1	9	9	30	178	21,1	9,91
P62996	Transformer-2 protein homolog beta OS=Mus musculus GN=Tra2b PE=1 SV=1 - [TRA2B_MOUSE]	809,93	21,53	1	7	7	32	288	33,6	11,25
P54103	DnaJ homolog subfamily C member 2 OS=Mus musculus GN=Dnajc2 PE=1 SV=2 - [DNJC2_MOUSE]	809,18	23,35	1	14	14	33	621	71,7	8,70
P08113	Endoplasmin OS=Mus musculus GN=Hsp90b1 PE=1 SV=2 - [ENPL_MOUSE]	808,75	6,86	1	3	5	22	802	92,4	4,82
Q76MZ3	Serine/threonine-protein phosphatase 2A 65 kDa regulatory subunit A alpha isoform OS=Mus musculus GN=Ppp2r1a PE=1 SV=3 - [2AAA_M	804,69	22,58	1	10	10	41	589	65,3	5,11
P97496	SWI/SNF complex subunit SMARCC1 OS=Mus musculus GN=Smarcc1 PE=1 SV=2 - [SMRC1_MOUSE]	804,48	15,31	1	8	14	41	1104	122,8	5,73
P30681	High mobility group protein B2 OS=Mus musculus GN=Hmgb2 PE=1 SV=3 - [HMGB2_MOUSE]	802,59	23,81	1	5	7	33	210	24,1	7,31
Q5F2E7	Nuclear fragile X mental retardation-interacting protein 2 OS=Mus musculus GN=Nufip2 PE=1 SV=1 - [NUFP2_MOUSE]	801,88	17,92	1	9	9	23	692	75,6	8,70
O70310	Glycylpeptide N-tetradecanoyltransferase 1 OS=Mus musculus GN=Nmt1 PE=1 SV=1 - [NMT1_MOUSE]	801,39	21,17	1	8	9	31	496	56,9	8,00
Q922K7	Putative ribosomal RNA methyltransferase NOP2 OS=Mus musculus GN=Nop2 PE=2 SV=1 - [NOP2_MOUSE]	801,19	12,86	1	7	7	23	793	86,7	9,22
P33215	Protein NEDD1 OS=Mus musculus GN=Nedd1 PE=2 SV=2 - [NEDD1_MOUSE]	796,03	9,39	1	4	4	13	660	71,2	7,84
Q08943	FACT complex subunit SSRP1 OS=Mus musculus GN=Ssrp1 PE=1 SV=2 - [SSRP1_MOUSE]	793,53	22,74	1	14	14	33	708	80,8	6,76
P63001	Ras-related C3 botulinum toxin substrate 1 OS=Mus musculus GN=Rac1 PE=1 SV=1 - [RAC1_MOUSE]	793,14	11,98	1	2	3	55	192	21,4	8,50
Q9Z1D1	Eukaryotic translation initiation factor 3 subunit G OS=Mus musculus GN=Eif3g PE=1 SV=1 - [EIF3G_MOUSE]	791,00	27,50	1	8	8	27	320	35,6	5,90
P58252	Elongation factor 2 OS=Mus musculus GN=Eef2 PE=1 SV=2 - [EF2_MOUSE]	789,71	20,05	1	15	16	43	858	95,3	6,83
Q8CDN6	Thioredoxin-like protein 1 OS=Mus musculus GN=Txnl1 PE=1 SV=3 - [TXNL1_MOUSE]	789,09	48,79	1	11	11	34	289	32,2	4,96
Q91WQ3	Tyrosyl-tRNA synthetase, cytoplasmic OS=Mus musculus GN=Yars PE=2 SV=3 - [SYYC_MOUSE]	789,01	37,50	1	19	19	39	528	59,1	7,01
O08663	Methionine aminopeptidase 2 OS=Mus musculus GN=Metap2 PE=1 SV=1 - [AMPM2_MOUSE]	788,68	16,53	1	6	6	26	478	52,9	5,82
P47856	Glucosamine--fructose-6-phosphate aminotransferase [isomerizing] 1 OS=Mus musculus GN=Gfpt1 PE=1 SV=3 - [GFPT1_MOUSE]	784,10	16,93	1	9	9	27	697	78,5	6,84
Q61749	Translation initiation factor eIF-2B subunit delta OS=Mus musculus GN=Eif2b4 PE=2 SV=2 - [EI2BD_MOUSE]	780,74	20,23	1	8	8	26	524	57,6	9,25
Q6A4J8	Ubiquitin carboxyl-terminal hydrolase 7 OS=Mus musculus GN=Usp7 PE=1 SV=1 - [UBP7_MOUSE]	778,60	12,15	1	11	11	48	1103	128,4	5,50
Q9D0I8	mRNA turnover protein 4 homolog OS=Mus musculus GN=Mrto4 PE=2 SV=1 - [MRT4_MOUSE]	776,83	41,42	1	10	10	39	239	27,5	8,54
Q6NZN0	RNA-binding protein 26 OS=Mus musculus GN=Rbm26 PE=1 SV=2 - [RBM26_MOUSE]	775,30	11,96	1	10	10	31	1012	114,1	9,16
Q8K4B0	Metastasis-associated protein MTA1 OS=Mus musculus GN=Mta1 PE=1 SV=1 - [MTA1_MOUSE]	774,33	16,50	1	6	9	32	715	80,7	9,29
P35700	Peroxiredoxin-1 OS=Mus musculus GN=Prdx1 PE=1 SV=1 - [PRDX1_MOUSE]	770,40	35,18	1	8	8	40	199	22,2	8,12
Q8VC03	Echinoderm microtubule-associated protein-like 3 OS=Mus musculus GN=Eml3 PE=2 SV=1 - [EMAL3_MOUSE]	769,56	9,92	1	7	7	17	897	95,6	7,05
Q3TIX9	U4/U6.U5 tri-snRNP-associated protein 2 OS=Mus musculus GN=Usp39 PE=2 SV=2 - [SNUT2_MOUSE]	769,28	22,16	1	9	9	33	564	65,1	8,90
Q9DBG6	Dolichyl-diphosphooligosaccharide--protein glycosyltransferase subunit 2 OS=Mus musculus GN=Rpn2 PE=2 SV=1 - [RPN2_MOUSE]	769,06	24,25	1	9	9	21	631	69,0	5,81
P27601	Guanine nucleotide-binding protein subunit alpha-13 OS=Mus musculus GN=Gna13 PE=1 SV=1 - [GNA13_MOUSE]	767,08	8,75	1	2	3	35	377	44,0	8,21
Q8CHY6	Transcriptional repressor p66 alpha OS=Mus musculus GN=Gatad2a PE=1 SV=2 - [P66A_MOUSE]	762,65	18,28	1	9	9	25	629	67,3	9,89
Q3UKJ7	WD40 repeat-containing protein SMU1 OS=Mus musculus GN=Smu1 PE=2 SV=2 - [SMU1_MOUSE]	760,04	24,37	1	9	9	28	513	57,5	7,18
P47754	F-actin-capping protein subunit alpha-2 OS=Mus musculus GN=Capza2 PE=1 SV=3 - [CAZA2_MOUSE]	755,64	58,39	1	8	10	26	286	32,9	5,85
Q9CYZ2	Tumor protein D54 OS=Mus musculus GN=Tpd52l2 PE=1 SV=1 - [TPD54_MOUSE]	754,00	19,55	1	3	3	19	220	24,0	6,15
P60335	Poly(rC)-binding protein 1 OS=Mus musculus GN=Pcbp1 PE=1 SV=1 - [PCBP1_MOUSE]	753,14	27,81	1	5	6	29	356	37,5	7,09
Q8R3N6	THO complex subunit 1 OS=Mus musculus GN=Thoc1 PE=1 SV=1 - [THOC1_MOUSE]	752,64	18,57	1	10	10	32	657	75,4	4,97
Q6VN19	Ran-binding protein 10 OS=Mus musculus GN=Ranbp10 PE=1 SV=2 - [RBP10_MOUSE]	752,33	20,48	1	8	8	21	620	67,1	6,58
Q69ZQ2	Pre-mRNA-splicing factor ISY1 homolog OS=Mus musculus GN=Isy1 PE=1 SV=2 - [ISY1_MOUSE]	751,06	27,72	1	8	8	23	285	33,0	5,17
P42669	Transcriptional activator protein Pur-alpha OS=Mus musculus GN=Pura PE=1 SV=1 - [PURA_MOUSE]	746,16	26,48	1	6	6	40	321	34,9	6,44
Q62189	U1 small nuclear ribonucleoprotein A OS=Mus musculus GN=Snrpa PE=2 SV=3 - [SNRPA_MOUSE]	745,26	29,62	1	6	8	39	287	31,8	9,80
Q922V4	Pleiotropic regulator 1 OS=Mus musculus GN=Plrg1 PE=2 SV=1 - [PLRG1_MOUSE]	744,08	30,21	1	10	10	31	513	56,9	9,17
Q9DAW6	U4/U6 small nuclear ribonucleoprotein Prp4 OS=Mus musculus GN=Prpf4 PE=2 SV=1 - [PRP4_MOUSE]	734,10	23,22	1	11	11	35	521	58,3	7,28
Q60737	Casein kinase II subunit alpha OS=Mus musculus GN=Csnk2a1 PE=1 SV=2 - [CSK21_MOUSE]	732,96	35,04	1	8	9	24	391	45,1	7,74
P61161	Actin-related protein 2 OS=Mus musculus GN=Actr2 PE=1 SV=1 - [ARP2_MOUSE]	732,21	23,86	1	8	8	27	394	44,7	6,74
Q91YQ5	Dolichyl-diphosphooligosaccharide--protein glycosyltransferase subunit 1 OS=Mus musculus GN=Rpn1 PE=2 SV=1 - [RPN1_MOUSE]	729,35	27,63	1	12	13	33	608	68,5	6,46
Q9JKY0	Cell differentiation protein RCD1 homolog OS=Mus musculus GN=Rqcd1 PE=1 SV=1 - [RCD1_MOUSE]	728,28	25,42	1	6	6	22	299	33,6	8,03
Q9QY06	Myosin-IXb OS=Mus musculus GN=Myo9b PE=1 SV=2 - [MYO9B_MOUSE]	727,72	4,35	1	8	8	25	2114	238,7	8,63
Q9JIF0	Protein arginine N-methyltransferase 1 OS=Mus musculus GN=Prmt1 PE=1 SV=1 - [ANM1_MOUSE]	724,58	31,27	1	12	12	30	371	42,4	5,43
Q9D753	Exosome complex exonuclease RRP43 OS=Mus musculus GN=Exosc8 PE=2 SV=1 - [EXOS8_MOUSE]	724,36	28,26	1	6	6	22	276	29,9	5,20
Q9QX47	Protein SON OS=Mus musculus GN=Son PE=1 SV=2 - [SON_MOUSE]	724,08	4,71	1	9	9	32	2444	265,5	5,60
Q61316	Heat shock 70 kDa protein 4 OS=Mus musculus GN=Hspa4 PE=1 SV=1 - [HSP74_MOUSE]	720,91	19,74	1	10	10	28	841	94,1	5,24
Q3U7R1	Extended synaptotagmin-1 OS=Mus musculus GN=Esyt1 PE=2 SV=2 - [ESYT1_MOUSE]	719,84	10,62	1	8	8	30	1092	121,5	5,95
P98086	Complement C1q subcomponent subunit A OS=Mus musculus GN=C1qa PE=1 SV=2 - [C1QA_MOUSE]	718,46	20,82	1	4	4	16	245	26,0	9,11
P47740	Fatty aldehyde dehydrogenase OS=Mus musculus GN=Aldh3a2 PE=2 SV=2 - [AL3A2_MOUSE]	714,51	15,70	1	6	6	20	484	53,9	8,35
Q4VBE8	WD repeat-containing protein 18 OS=Mus musculus GN=Wdr18 PE=2 SV=1 - [WDR18_MOUSE]	713,88	20,19	1	7	7	29	431	47,2	6,89
O54724	Polymerase I and transcript release factor OS=Mus musculus GN=Ptrf PE=1 SV=1 - [PTRF_MOUSE]	713,25	23,47	1	6	6	18	392	43,9	5,52
A2BE28	Protein LAS1 homolog OS=Mus musculus GN=Las1l PE=2 SV=1 - [LAS1L_MOUSE]	713,03	17,27	1	11	11	32	776	89,4	4,44
P62835	Ras-related protein Rap-1A OS=Mus musculus GN=Rap1a PE=2 SV=1 - [RAP1A_MOUSE]	708,79	19,57	2	5	5	35	184	21,0	6,67
Q8K4I3	Rho guanine nucleotide exchange factor 6 OS=Mus musculus GN=Arhgef6 PE=1 SV=1 - [ARHG6_MOUSE]	707,94	7,39	1	4	5	23	771	87,0	5,87
P16125	L-lactate dehydrogenase B chain OS=Mus musculus GN=Ldhb PE=1 SV=2 - [LDHB_MOUSE]	705,99	30,54	1	9	9	24	334	36,5	6,05
Q3U487	E3 ubiquitin-protein ligase HECTD3 OS=Mus musculus GN=Hectd3 PE=1 SV=2 - [HECD3_MOUSE]	699,31	16,14	1	11	11	28	861	97,3	5,47
O54734	Dolichyl-diphosphooligosaccharide--protein glycosyltransferase 48 kDa subunit OS=Mus musculus GN=Ddost PE=1 SV=2 - [OST48_MOUSE]	695,45	25,85	1	9	9	33	441	49,0	5,83
Q8R010	Aminoacyl tRNA synthase complex-interacting multifunctional protein 2 OS=Mus musculus GN=Aimp2 PE=1 SV=2 - [AIMP2_MOUSE]	694,96	33,75	1	5	5	39	320	35,4	7,83
A2RSY6	TRMT1-like protein OS=Mus musculus GN=Trmt1l PE=2 SV=1 - [TRM1L_MOUSE]	693,63	21,02	1	10	10	24	728	80,8	7,50
Q9CQW9	Interferon-induced transmembrane protein 3 OS=Mus musculus GN=Ifitm3 PE=1 SV=1 - [IFM3_MOUSE]	691,98	32,12	1	3	3	14	137	14,9	7,40
P62320	Small nuclear ribonucleoprotein Sm D3 OS=Mus musculus GN=Snrpd3 PE=1 SV=1 - [SMD3_MOUSE]	691,17	34,13	1	4	4	30	126	13,9	10,32
Q99JY9	Actin-related protein 3 OS=Mus musculus GN=Actr3 PE=1 SV=3 - [ARP3_MOUSE]	691,16	34,93	1	11	11	32	418	47,3	5,88
Q9CWZ3	RNA-binding protein 8A OS=Mus musculus GN=Rbm8a PE=1 SV=3 - [RBM8A_MOUSE]	687,65	17,24	1	2	2	29	174	19,9	6,16
P48036	Annexin A5 OS=Mus musculus GN=Anxa5 PE=1 SV=1 - [ANXA5_MOUSE]	686,74	29,78	1	8	8	19	319	35,7	4,96
P49717	DNA replication licensing factor MCM4 OS=Mus musculus GN=Mcm4 PE=2 SV=1 - [MCM4_MOUSE]	685,81	15,08	1	11	11	25	862	96,7	7,20
P62748	Hippocalcin-like protein 1 OS=Mus musculus GN=Hpcal1 PE=2 SV=2 - [HPCL1_MOUSE]	682,00	34,20	1	6	6	23	193	22,3	5,50
Q9D7M1	Protein C20orf11 homolog OS=Mus musculus PE=2 SV=1 - [CT011_MOUSE]	681,92	48,68	1	9	9	18	228	26,8	4,97
Q9D2E2	Target of EGR1 protein 1 OS=Mus musculus GN=Toe1 PE=2 SV=1 - [TOE1_MOUSE]	681,46	22,90	1	8	8	20	511	56,8	6,64
Q9CZ44	NSFL1 cofactor p47 OS=Mus musculus GN=Nsfl1c PE=1 SV=1 - [NSF1C_MOUSE]	678,05	22,43	1	6	6	15	370	40,7	5,15
P05064	Fructose-bisphosphate aldolase A OS=Mus musculus GN=Aldoa PE=1 SV=2 - [ALDOA_MOUSE]	678,03	29,40	1	7	7	20	364	39,3	8,09
O54941	SWI/SNF-related matrix-associated actin-dependent regulator chromatin subfamily E member 1 OS=Mus musculus GN=Smarce1 PE=1 SV=	669,36	29,44	1	8	8	22	411	46,6	4,88
Q3U0V1	Far upstream element-binding protein 2 OS=Mus musculus GN=Khsrp PE=1 SV=2 - [FUBP2_MOUSE]	668,63	18,72	1	10	10	23	748	76,7	7,33
Q8BTV2	Cleavage and polyadenylation specificity factor subunit 7 OS=Mus musculus GN=Cpsf7 PE=1 SV=2 - [CPSF7_MOUSE]	668,13	17,41	1	7	7	29	471	52,0	8,00
O89086	Putative RNA-binding protein 3 OS=Mus musculus GN=Rbm3 PE=1 SV=1 - [RBM3_MOUSE]	663,65	37,25	1	5	5	35	153	16,6	7,50
Q9CZX0	Elongator complex protein 3 OS=Mus musculus GN=Elp3 PE=2 SV=1 - [ELP3_MOUSE]	662,17	34,73	1	15	15	47	547	62,3	8,90
Q8BG41	Proteasome subunit beta type-11 OS=Mus musculus GN=Psmb11 PE=1 SV=1 - [PSB11_MOUSE]	660,40	19,87	1	5	5	25	302	33,2	6,81
Q60710	SAM domain and HD domain-containing protein 1 OS=Mus musculus GN=Samhd1 PE=1 SV=2 - [SAMH1_MOUSE]	656,34	19,14	1	8	9	25	627	72,6	7,96

Q9JJI8	60S ribosomal protein L38 OS=Mus musculus GN=Rpl38 PE=2 SV=3 - [RL38_MOUSE]	656,17	51,43	1	6	6	27	70	8,2	10,10
P04441	H-2 class II histocompatibility antigen gamma chain OS=Mus musculus GN=Cd74 PE=1 SV=3 - [HG2A_MOUSE]	655,50	18,28	1	5	5	24	279	31,5	8,34
P69566	Ran-binding protein 9 OS=Mus musculus GN=Ranbp9 PE=1 SV=1 - [RANB9_MOUSE]	654,34	18,99	1	10	10	27	653	71,0	6,84
Q99JY0	Trifunctional enzyme subunit beta, mitochondrial OS=Mus musculus GN=Hadhb PE=1 SV=1 - [ECHB_MOUSE]	653,06	12,63	1	5	6	42	475	51,4	9,38
O08547	Vesicle-trafficking protein SEC22b OS=Mus musculus GN=Sec22b PE=1 SV=3 - [SC22B_MOUSE]	651,94	33,95	1	6	6	19	215	24,7	8,51
Q5SWU9	Acetyl-CoA carboxylase 1 OS=Mus musculus GN=Acaca PE=1 SV=1 - [ACACA_MOUSE]	651,16	8,14	1	15	15	44	2345	265,1	6,39
P62814	V-type proton ATPase subunit B, brain isoform OS=Mus musculus GN=Atp6v1b2 PE=1 SV=1 - [VATB2_MOUSE]	645,22	23,68	1	8	8	18	511	56,5	5,81
Q8BVL9	Janus kinase and microtubule-interacting protein 1 OS=Mus musculus GN=Jakmip1 PE=1 SV=2 - [JKIP1_MOUSE]	643,70	20,93	1	9	10	26	626	73,1	6,09
Q99JR8	SWI/SNF-related matrix-associated actin-dependent regulator of chromatin subfamily D member 2 OS=Mus musculus GN=Smarcd2 PE=2 S	642,86	17,89	1	7	7	23	531	59,0	9,64
Q8BI72	CDKN2A-interacting protein OS=Mus musculus GN=Cdkn2aip PE=2 SV=1 - [CARF_MOUSE]	642,83	16,70	1	7	7	15	563	59,7	9,16
Q9DCD2	Pre-mRNA-splicing factor SYF1 OS=Mus musculus GN=Xab2 PE=2 SV=1 - [SYF1_MOUSE]	641,02	25,73	1	17	17	30	855	99,9	6,23
Q8VBV3	Exosome complex exonuclease RRP4 OS=Mus musculus GN=Exosc2 PE=2 SV=1 - [EXOS2_MOUSE]	640,89	27,99	1	6	6	18	293	32,6	7,50
Q9CY57	Friend of PRMT1 protein OS=Mus musculus GN=Fop PE=2 SV=2 - [FOP_MOUSE]	638,55	25,70	1	4	4	16	249	26,6	12,23
O88796	Ribonuclease P protein subunit p30 OS=Mus musculus GN=Rpp30 PE=2 SV=1 - [RPP30_MOUSE]	631,16	45,90	1	9	9	17	268	29,5	9,06
Q91W50	Cold shock domain-containing protein E1 OS=Mus musculus GN=Csde1 PE=2 SV=1 - [CSDE1_MOUSE]	630,92	16,54	1	12	12	40	798	88,7	6,37
Q9ERU9	E3 SUMO-protein ligase RanBP2 OS=Mus musculus GN=Ranbp2 PE=1 SV=2 - [RBP2_MOUSE]	630,92	2,13	1	4	4	13	3053	340,9	6,18
Q6WVG3	BTB/POZ domain-containing protein KCTD12 OS=Mus musculus GN=Kctd12 PE=1 SV=1 - [KCD12_MOUSE]	629,88	31,50	1	10	10	30	327	35,9	5,81
P62915	Transcription initiation factor IIB OS=Mus musculus GN=Gtf2b PE=1 SV=1 - [TF2B_MOUSE]	629,13	34,81	1	9	9	19	316	34,8	8,35
Q8VBV7	COP9 signalosome complex subunit 8 OS=Mus musculus GN=Cops8 PE=1 SV=1 - [CSN8_MOUSE]	626,42	36,36	1	5	5	19	209	23,2	5,20
Q7TMF2	3'-5' exoribonuclease 1 OS=Mus musculus GN=Eri1 PE=1 SV=2 - [ERI1_MOUSE]	625,20	19,13	1	4	4	12	345	39,5	8,31
P62482	Voltage-gated potassium channel subunit beta-2 OS=Mus musculus GN=Kcnab2 PE=1 SV=1 - [KCAB2_MOUSE]	623,99	33,79	1	8	8	18	367	41,0	9,00
Q8CBY8	Dynactin subunit 4 OS=Mus musculus GN=Dctn4 PE=2 SV=1 - [DCTN4_MOUSE]	611,56	18,42	1	6	6	16	467	53,0	7,72
Q922U1	U4/U6 small nuclear ribonucleoprotein Prp3 OS=Mus musculus GN=Prpf3 PE=1 SV=1 - [PRPF3_MOUSE]	611,54	18,89	1	11	11	24	683	77,4	9,50
Q9CQT2	RNA-binding protein 7 OS=Mus musculus GN=Rbm7 PE=1 SV=1 - [RBM7_MOUSE]	611,26	43,02	1	8	8	29	265	30,1	9,38
Q8BH15	CCR4-NOT transcription complex subunit 10 OS=Mus musculus GN=Cnot10 PE=2 SV=1 - [CNOTA_MOUSE]	610,04	22,45	1	12	12	24	744	81,8	7,68
P62309	Small nuclear ribonucleoprotein G OS=Mus musculus GN=Snrpg PE=1 SV=1 - [RUXG_MOUSE]	610,00	38,16	1	4	4	17	76	8,5	8,88
Q9CQD1	Ras-related protein Rab-5A OS=Mus musculus GN=Rab5a PE=1 SV=1 - [RAB5A_MOUSE]	607,74	26,98	1	2	5	24	215	23,6	8,15
P23198	Chromobox protein homolog 3 OS=Mus musculus GN=Cbx3 PE=1 SV=2 - [CBX3_MOUSE]	604,77	23,50	1	4	4	13	183	20,8	5,22
P62305	Small nuclear ribonucleoprotein E OS=Mus musculus GN=Snrpe PE=2 SV=1 - [RUXE_MOUSE]	604,31	45,65	1	5	5	27	92	10,8	9,44
P10852	4F2 cell-surface antigen heavy chain OS=Mus musculus GN=Slc3a2 PE=1 SV=1 - [4F2_MOUSE]	600,44	19,01	1	8	8	19	526	58,3	5,91
Q60817	Nascent polypeptide-associated complex subunit alpha OS=Mus musculus GN=Naca PE=1 SV=1 - [NACA_MOUSE]	600,02	25,58	2	4	4	10	215	23,4	4,56
P97376	Protein FRG1 OS=Mus musculus GN=Frg1 PE=1 SV=2 - [FRG1_MOUSE]	599,83	18,60	1	3	3	13	258	29,1	8,92
Q923G2	DNA-directed RNA polymerases I, II, and III subunit RPABC3 OS=Mus musculus GN=Polr2h PE=2 SV=3 - [RPAB3_MOUSE]	599,80	26,67	1	4	4	18	150	17,1	4,68
P11438	Lysosome-associated membrane glycoprotein 1 OS=Mus musculus GN=Lamp1 PE=1 SV=2 - [LAMP1_MOUSE]	598,09	5,67	1	2	2	25	406	43,8	8,40
Q8VHR5	Transcriptional repressor p66-beta OS=Mus musculus GN=Gatad2b PE=1 SV=1 - [P66B_MOUSE]	597,26	29,12	1	11	11	35	594	65,4	9,70
Q9JHE7	Protein TSSC4 OS=Mus musculus GN=Tssc4 PE=2 SV=1 - [TSSC4_MOUSE]	595,49	11,99	1	3	3	9	317	33,5	5,14
Q9CQR2	40S ribosomal protein S21 OS=Mus musculus GN=Rps21 PE=2 SV=1 - [RS21_MOUSE]	591,61	48,19	1	5	5	17	83	9,1	8,51
Q99ME2	WD repeat-containing protein 6 OS=Mus musculus GN=Wdr6 PE=2 SV=1 - [WDR6_MOUSE]	588,05	4,27	1	4	4	28	1125	121,8	6,98
Q9CSN1	SNW domain-containing protein 1 OS=Mus musculus GN=Snw1 PE=1 SV=3 - [SNW1_MOUSE]	586,92	14,18	1	6	6	16	536	61,4	9,48
Q9DBZ5	Eukaryotic translation initiation factor 3 subunit K OS=Mus musculus GN=Eif3k PE=1 SV=1 - [EIF3K_MOUSE]	586,70	28,90	1	5	5	20	218	25,1	4,93
Q8CH18	Cell division cycle and apoptosis regulator protein 1 OS=Mus musculus GN=Ccar1 PE=1 SV=1 - [CCAR1_MOUSE]	585,69	10,47	1	9	9	35	1146	132,0	5,76
Q99LL5	Periodic tryptophan protein 1 homolog OS=Mus musculus GN=Pwp1 PE=1 SV=1 - [PWP1_MOUSE]	584,04	29,74	1	8	8	15	501	55,6	4,82
Q9Z108	Double-stranded RNA-binding protein Staufen homolog 1 OS=Mus musculus GN=Stau1 PE=2 SV=1 - [STAU1_MOUSE]	580,17	21,15	1	8	8	22	487	53,9	9,52
Q8JZX4	Splicing factor 45 OS=Mus musculus GN=Rbm17 PE=1 SV=1 - [SPF45_MOUSE]	579,60	31,36	1	11	11	25	405	45,3	5,82
Q99J87	Probable ATP-dependent RNA helicase DHX58 OS=Mus musculus GN=Dhx58 PE=1 SV=2 - [DHX58_MOUSE]	578,13	20,94	1	11	11	22	678	76,7	8,19
Q80VI1	Tripartite motif-containing protein 56 OS=Mus musculus GN=Trim56 PE=1 SV=1 - [TRI56_MOUSE]	577,45	12,26	1	7	7	24	734	79,5	7,96
Q80YV3	Transformation/transcription domain-associated protein OS=Mus musculus GN=Trrap PE=1 SV=2 - [TRRAP_MOUSE]	577,37	5,61	1	11	11	25	2565	291,4	8,48
Q8BV49	Pyrin and HIN domain-containing protein 1 OS=Mus musculus GN=Pyhin1 PE=2 SV=1 - [IFIX_MOUSE]	573,01	21,90	1	5	5	16	420	46,9	9,45
B1AZI6	THO complex subunit 2 OS=Mus musculus GN=Thoc2 PE=3 SV=1 - [THOC2_MOUSE]	569,08	6,09	1	6	7	23	1594	182,7	8,44
E9Q557	Desmoplakin OS=Mus musculus GN=Dsp PE=3 SV=1 - [DESP_MOUSE]	568,87	3,88	1	10	10	17	2883	332,7	6,80
Q6PHZ2	Calcium/calmodulin-dependent protein kinase type II subunit delta OS=Mus musculus GN=Camk2d PE=1 SV=1 - [KCC2D_MOUSE]	568,84	18,24	1	5	7	20	499	56,3	7,25
Q9CWX9	Probable ATP-dependent RNA helicase DDX47 OS=Mus musculus GN=Ddx47 PE=2 SV=2 - [DDX47_MOUSE]	567,45	22,64	1	8	8	22	455	50,6	9,10
**Q99M28**	RNA-binding protein with serine-rich domain 1 OS=Mus musculus GN=Rnps1 PE=2 SV=1 - [RNPS1_MOUSE]	567,27	21,64	1	5	5	21	305	34,2	11,84
Q9DBR3	Armadillo repeat-containing protein 8 OS=Mus musculus GN=Armc8 PE=1 SV=2 - [ARMC8_MOUSE]	567,00	11,59	1	7	7	23	673	75,3	6,73
Q9WUP7	Ubiquitin carboxyl-terminal hydrolase isozyme L5 OS=Mus musculus GN=Uchl5 PE=2 SV=2 - [UCHL5_MOUSE]	563,59	27,05	1	8	8	22	329	37,6	5,33
P23475	X-ray repair cross-complementing protein 6 OS=Mus musculus GN=Xrcc6 PE=2 SV=5 - [XRCC6_MOUSE]	563,51	24,34	1	11	11	23	608	69,4	6,79
Q1HFZ0	tRNA (cytosine-5-)-methyltransferase NSUN2 OS=Mus musculus GN=Nsun2 PE=1 SV=2 - [NSUN2_MOUSE]	563,10	12,29	1	8	8	23	757	85,4	6,58
Q3UHJ0	AP2-associated protein kinase 1 OS=Mus musculus GN=Aak1 PE=1 SV=2 - [AAK1_MOUSE]	562,95	6,15	1	4	4	13	959	103,3	6,70
Q7TSC1	Large proline-rich protein BAT2 OS=Mus musculus GN=Bat2 PE=1 SV=1 - [BAT2_MOUSE]	562,03	2,64	1	3	3	14	2158	229,1	9,39
Q3URQ0	Testis-expressed sequence 10 protein OS=Mus musculus GN=Tex10 PE=2 SV=1 - [TEX10_MOUSE]	561,36	6,90	1	5	5	19	928	105,1	9,16
Q8K019	Bcl-2-associated transcription factor 1 OS=Mus musculus GN=Bclaf1 PE=1 SV=2 - [BCLF1_MOUSE]	557,59	11,53	1	8	8	30	919	105,9	9,99
Q8CHW4	Translation initiation factor eIF-2B subunit epsilon OS=Mus musculus GN=Eif2b5 PE=1 SV=1 - [EI2BE_MOUSE]	556,42	11,72	1	7	7	15	717	80,0	5,07
Q80UG5	Septin-9 OS=Mus musculus GN=Sept9 PE=1 SV=1 - [SEPT9_MOUSE]	556,11	12,35	1	6	6	20	583	65,5	8,90
P12242	Mitochondrial brown fat uncoupling protein 1 OS=Mus musculus GN=Ucp1 PE=2 SV=2 - [UCP1_MOUSE]	555,98	8,47	1	2	2	16	307	33,2	9,14
Q99LI7	Cleavage stimulation factor subunit 3 OS=Mus musculus GN=Cstf3 PE=1 SV=1 - [CSTF3_MOUSE]	553,74	9,62	1	6	6	15	717	82,8	8,12
Q9CWK3	CD2 antigen cytoplasmic tail-binding protein 2 OS=Mus musculus GN=Cd2bp2 PE=1 SV=1 - [CD2B2_MOUSE]	551,02	19,59	1	5	5	14	342	37,7	4,60
Q80YR5	Scaffold attachment factor B2 OS=Mus musculus GN=Safb2 PE=1 SV=2 - [SAFB2_MOUSE]	550,10	6,96	1	3	4	13	991	111,8	6,38
P67778	Prohibitin OS=Mus musculus GN=Phb PE=1 SV=1 - [PHB_MOUSE]	548,61	36,40	1	7	7	14	272	29,8	5,76
Q91ZW3	SWI/SNF-related matrix-associated actin-dependent regulator of chromatin subfamily A member 5 OS=Mus musculus GN=Smarca5 PE=1 S	548,30	19,12	1	18	18	30	1051	121,6	8,15
P29788	Vitronectin OS=Mus musculus GN=Vtn PE=1 SV=2 - [VTNC_MOUSE]	546,73	11,51	1	5	5	22	478	54,8	5,88
Q9JJY4	Probable ATP-dependent RNA helicase DDX20 OS=Mus musculus GN=Ddx20 PE=1 SV=2 - [DDX20_MOUSE]	542,34	9,82	1	6	6	25	825	91,7	6,74
Q9D883	Splicing factor U2AF 35 kDa subunit OS=Mus musculus GN=U2af1 PE=2 SV=4 - [U2AF1_MOUSE]	542,10	16,32	1	3	3	19	239	27,8	8,81
Q6ZQ38	Cullin-associated NEDD8-dissociated protein 1 OS=Mus musculus GN=Cand1 PE=2 SV=2 - [CAND1_MOUSE]	540,25	12,85	1	12	13	24	1230	136,2	5,78
Q03963	Interferon-induced, double-stranded RNA-activated protein kinase OS=Mus musculus GN=Eif2ak2 PE=1 SV=2 - [E2AK2_MOUSE]	536,77	11,07	1	4	4	13	515	58,2	8,57
P62715	Serine/threonine-protein phosphatase 2A catalytic subunit beta isoform OS=Mus musculus GN=Ppp2cb PE=1 SV=1 - [PP2AB_MOUSE]	532,07	8,41	2	2	2	11	309	35,6	5,43
Q8CBE3	WD repeat-containing protein 37 OS=Mus musculus GN=Wdr37 PE=2 SV=1 - [WDR37_MOUSE]	529,97	11,49	1	5	5	13	496	55,0	7,23
Q91WG4	Elongator complex protein 2 OS=Mus musculus GN=Elp2 PE=1 SV=1 - [ELP2_MOUSE]	528,65	8,42	1	6	6	13	831	93,0	5,82
O55131	Septin-7 OS=Mus musculus GN=Sept7 PE=1 SV=1 - [SEPT7_MOUSE]	527,05	18,81	1	5	6	18	436	50,5	8,57
Q9CQI7	U2 small nuclear ribonucleoprotein B'' OS=Mus musculus GN=Snrpb2 PE=2 SV=1 - [RU2B_MOUSE]	526,75	25,33	1	4	6	22	225	25,3	9,72
P62862	40S ribosomal protein S30 OS=Mus musculus GN=Fau PE=3 SV=1 - [RS30_MOUSE]	526,21	20,34	1	3	3	43	59	6,6	12,15
P61924	Coatomer subunit zeta-1 OS=Mus musculus GN=Copz1 PE=2 SV=1 - [COPZ1_MOUSE]	525,69	36,16	1	6	6	16	177	20,2	4,81
Q8BFQ4	WD repeat-containing protein 82 OS=Mus musculus GN=Wdr82 PE=1 SV=1 - [WDR82_MOUSE]	520,43	23,64	1	7	7	16	313	35,1	7,69
P59708	Pre-mRNA branch site protein p14 OS=Mus musculus GN=Sf3b14 PE=2 SV=1 - [PM14_MOUSE]	519,44	29,60	1	4	4	13	125	14,6	9,38
P17047	Lysosome-associated membrane glycoprotein 2 OS=Mus musculus GN=Lamp2 PE=2 SV=2 - [LAMP2_MOUSE]	517,72	8,67	1	4	4	28	415	45,7	7,39
Q921F4	Heterogeneous nuclear ribonucleoprotein L-like OS=Mus musculus GN=Hnrpll PE=1 SV=3 - [HNRLL_MOUSE]	516,81	15,74	1	6	6	29	591	64,1	5,85
B2RY56	RNA-binding protein 25 OS=Mus musculus GN=Rbm25 PE=1 SV=1 - [RBM25_MOUSE]	516,47	14,39	1	11	11	34	841	99,9	6,40
Q9ES28	Rho guanine nucleotide exchange factor 7 OS=Mus musculus GN=Arhgef7 PE=1 SV=2 - [ARHG7_MOUSE]	516,01	5,10	1	3	4	15	862	97,0	6,80
P83887	Tubulin gamma-1 chain OS=Mus musculus GN=Tubg1 PE=1 SV=1 - [TBG1_MOUSE]	512,40	32,59	1	11	11	26	451	51,1	6,02
P60762	Mortality factor 4-like protein 1 OS=Mus musculus GN=Morf4l1 PE=2 SV=2 - [MO4L1_MOUSE]	512,37	25,14	1	8	8	20	362	41,5	9,32
P16546	Spectrin alpha chain, brain OS=Mus musculus GN=Sptan1 PE=1 SV=4 - [SPTA2_MOUSE]	512,19	7,69	1	14	14	25	2472	284,4	5,33
Q9R1C7	Pre-mRNA-processing factor 40 homolog A OS=Mus musculus GN=Prpf40a PE=1 SV=1 - [PR40A_MOUSE]	511,67	9,55	1	7	7	21	953	108,4	7,69
Q61171	Peroxiredoxin-2 OS=Mus musculus GN=Prdx2 PE=1 SV=3 - [PRDX2_MOUSE]	511,27	31,82	1	6	6	17	198	21,8	5,41
Q9CQV8	14-3-3 protein beta/alpha OS=Mus musculus GN=Ywhab PE=1 SV=3 - [1433B_MOUSE]	510,47	28,86	1	4	7	20	246	28,1	4,83
Q9CVB6	Actin-related protein 2/3 complex subunit 2 OS=Mus musculus GN=Arpc2 PE=1 SV=3 - [ARPC2_MOUSE]	508,91	35,00	1	11	11	28	300	34,3	7,36
P11352	Glutathione peroxidase 1 OS=Mus musculus GN=Gpx1 PE=1 SV=2 - [GPX1_MOUSE]	508,24	46,77	1	6	6	22	201	22,3	7,21
Q9D3D9	ATP synthase subunit delta, mitochondrial OS=Mus musculus GN=Atp5d PE=1 SV=1 - [ATPD_MOUSE]	506,99	13,69	1	2	2	7	168	17,6	5,08
Q99LX5	Multiple myeloma tumor-associated protein 2 homolog OS=Mus musculus GN=Mmtag2 PE=2 SV=1 - [MMTA2_MOUSE]	506,47	10,38	1	2	2	12	260	29,3	9,86
Q9D1Q1	M-phase phosphoprotein 6 OS=Mus musculus GN=Mphosph6 PE=1 SV=1 - [MPH6_MOUSE]	506,00	20,50	1	4	4	17	161	19,1	5,30
Q8BFZ9	Erlin-2 OS=Mus musculus GN=Erlin2 PE=1 SV=1 - [ERLN2_MOUSE]	501,24	29,71	1	9	9	15	340	37,8	5,50
Q9D706	RNA polymerase II-associated protein 3 OS=Mus musculus GN=Rpap3 PE=1 SV=1 - [RPAP3_MOUSE]	501,23	21,82	1	10	11	24	660	74,1	7,99
Q8CCS6	Polyadenylate-binding protein 2 OS=Mus musculus GN=Pabpn1 PE=2 SV=3 - [PABP2_MOUSE]	500,85	11,59	1	5	5	19	302	32,3	5,17
P63158	High mobility group protein B1 OS=Mus musculus GN=Hmgb1 PE=1 SV=2 - [HMGB1_MOUSE]	500,67	18,14	1	5	6	25	215	24,9	5,74
P62315	Small nuclear ribonucleoprotein Sm D1 OS=Mus musculus GN=Snrpd1 PE=2 SV=1 - [SMD1_MOUSE]	498,88	37,82	1	4	4	19	119	13,3	11,56
Q8BHL5	Engulfment and cell motility protein 2 OS=Mus musculus GN=Elmo2 PE=1 SV=1 - [ELMO2_MOUSE]	496,72	15,85	1	8	11	32	732	83,8	5,95
Q9Z1M8	Protein Red OS=Mus musculus GN=Ik PE=2 SV=2 - [RED_MOUSE]	496,09	23,34	1	10	11	27	557	65,6	6,64
P17182	Alpha-enolase OS=Mus musculus GN=Eno1 PE=1 SV=3 - [ENOA_MOUSE]	496,02	22,35	1	7	7	15	434	47,1	6,80
P48193	Protein 4.1 OS=Mus musculus GN=Epb41 PE=1 SV=2 - [41_MOUSE]	490,73	8,74	1	5	5	25	858	95,9	5,60
P55937	Golgin subfamily A member 3 OS=Mus musculus GN=Golga3 PE=1 SV=3 - [GOGA3_MOUSE]	490,60	2,96	1	2	3	7	1487	167,1	5,40
Q8VI93	2'-5'-oligoadenylate synthase 3 OS=Mus musculus GN=Oas3 PE=2 SV=1 - [OAS3_MOUSE]	490,52	8,96	1	7	7	33	1138	126,3	8,72
P62274	40S ribosomal protein S29 OS=Mus musculus GN=Rps29 PE=2 SV=2 - [RS29_MOUSE]	486,62	33,93	1	2	2	31	56	6,7	10,13
Q91ZU6	Bullous pemphigoid antigen 1 OS=Mus musculus GN=Dst PE=1 SV=1 - [BPA1_MOUSE]	483,79	1,41	1	7	8	14	7389	833,1	5,31
Q8K284	General transcription factor 3C polypeptide 1 OS=Mus musculus GN=Gtf3c1 PE=2 SV=2 - [TF3C1_MOUSE]	483,49	7,95	1	11	11	31	2101	237,3	7,25
Q9DB05	Alpha-soluble NSF attachment protein OS=Mus musculus GN=Napa PE=1 SV=1 - [SNAA_MOUSE]	482,71	31,53	1	6	6	15	295	33,2	5,45
P61963	DDB1- and CUL4-associated factor 7 OS=Mus musculus GN=Dcaf7 PE=2 SV=1 - [DCAF7_MOUSE]	480,35	16,96	1	4	4	10	342	38,9	5,52
Q921I9	Exosome complex exonuclease RRP41 OS=Mus musculus GN=Exosc4 PE=2 SV=3 - [EXOS4_MOUSE]	479,97	27,35	1	5	5	18	245	26,2	6,15
Q8BGC0	HIV Tat-specific factor 1 homolog OS=Mus musculus GN=Htatsf1 PE=1 SV=1 - [HTSF1_MOUSE]	479,03	10,96	1	8	8	22	757	86,2	4,40
Q9D287	Pre-mRNA-splicing factor SPF27 OS=Mus musculus GN=Bcas2 PE=1 SV=1 - [SPF27_MOUSE]	478,68	39,11	1	7	7	16	225	26,1	5,66
O35129	Prohibitin-2 OS=Mus musculus GN=Phb2 PE=1 SV=1 - [PHB2_MOUSE]	476,62	21,74	1	5	5	14	299	33,3	9,83
O08784	Treacle protein OS=Mus musculus GN=Tcof1 PE=1 SV=1 - [TCOF_MOUSE]	471,60	10,76	1	10	10	26	1320	134,9	9,35
Q9EQ61	Pescadillo homolog OS=Mus musculus GN=Pes1 PE=1 SV=1 - [PESC_MOUSE]	471,57	9,08	1	5	5	11	584	67,8	6,84
Q9D7S7	60S ribosomal protein L22-like 1 OS=Mus musculus GN=Rpl22l1 PE=1 SV=1 - [RL22L_MOUSE]	468,21	26,23	1	2	3	19	122	14,5	9,45

Q99KF1	Transmembrane emp24 domain-containing protein 9 OS=Mus musculus GN=Tmed9 PE=2 SV=2 - [TMED9_MOUSE]	467,68	15,32	1	5	5	17	235	27,1	8,41
Q5SF07	Insulin-like growth factor 2 mRNA-binding protein 2 OS=Mus musculus GN=Igf2bp2 PE=1 SV=1 - [IF2B2_MOUSE]	467,65	8,78	1	2	4	16	592	65,5	8,03
Q9QXK7	Cleavage and polyadenylation specificity factor subunit 3 OS=Mus musculus GN=Cpsf3 PE=1 SV=2 - [CPSF3_MOUSE]	465,97	18,27	1	10	10	28	684	77,5	5,60
A2AF47	Dedicator of cytokinesis protein 11 OS=Mus musculus GN=Dock11 PE=1 SV=1 - [DOC11_MOUSE]	464,65	6,27	1	11	11	30	2073	237,6	7,78
Q8BTW3	Exosome complex exonuclease MTR3 OS=Mus musculus GN=Exosc6 PE=2 SV=1 - [EXOS6_MOUSE]	462,07	23,81	1	3	3	12	273	28,4	6,11
O70251	Elongation factor 1-beta OS=Mus musculus GN=Eef1b PE=1 SV=5 - [EF1B_MOUSE]	461,56	12,44	1	2	2	8	225	24,7	4,69
P19783	Cytochrome c oxidase subunit 4 isoform 1, mitochondrial OS=Mus musculus GN=Cox4i1 PE=1 SV=2 - [COX41_MOUSE]	460,56	31,36	1	5	5	15	169	19,5	9,23
Q3UMC0	Spermatogenesis-associated protein 5 OS=Mus musculus GN=Spata5 PE=2 SV=2 - [SPAT5_MOUSE]	460,41	7,73	1	5	6	33	893	97,2	8,24
Q9R0Q3	Transmembrane emp24 domain-containing protein 2 OS=Mus musculus GN=Tmed2 PE=1 SV=1 - [TMED2_MOUSE]	457,95	16,92	1	3	3	14	201	22,7	5,17
Q921I1	Serotransferrin OS=Mus musculus GN=Tf PE=1 SV=1 - [TRFE_MOUSE]	457,82	13,92	1	8	8	20	697	76,7	7,18
P62204	Calmodulin OS=Mus musculus GN=Calm1 PE=1 SV=2 - [CALM_MOUSE]	456,66	45,64	1	4	4	18	149	16,8	4,22
Q8BTZ4	Anaphase-promoting complex subunit 5 OS=Mus musculus GN=Anapc5 PE=2 SV=1 - [APC5_MOUSE]	455,51	10,27	1	6	6	12	740	83,0	6,71
Q80TV8	CLIP-associating protein 1 OS=Mus musculus GN=Clasp1 PE=1 SV=2 - [CLAP1_MOUSE]	455,39	3,39	1	3	3	15	1535	169,1	9,03
Q6PDL0	Cytoplasmic dynein 1 light intermediate chain 2 OS=Mus musculus GN=Dync1li2 PE=1 SV=2 - [DC1L2_MOUSE]	455,02	15,65	1	7	7	18	492	54,2	6,28
P12787	Cytochrome c oxidase subunit 5A, mitochondrial OS=Mus musculus GN=Cox5a PE=1 SV=2 - [COX5A_MOUSE]	453,04	22,60	1	4	4	14	146	16,1	6,54
P14824	Annexin A6 OS=Mus musculus GN=Anxa6 PE=1 SV=3 - [ANXA6_MOUSE]	451,45	20,65	1	11	11	36	673	75,8	5,50
P06240	Proto-oncogene tyrosine-protein kinase LCK OS=Mus musculus GN=Lck PE=1 SV=4 - [LCK_MOUSE]	449,98	16,90	1	6	7	29	509	57,9	5,25
P68254	14-3-3 protein theta OS=Mus musculus GN=Ywhaq PE=1 SV=1 - [1433T_MOUSE]	449,74	28,57	1	3	6	15	245	27,8	4,78
Q8K1N4	Spermatogenesis-associated serine-rich protein 2 OS=Mus musculus GN=Spats2 PE=1 SV=1 - [SPAS2_MOUSE]	449,23	9,72	1	4	4	14	545	58,9	9,00
Q7TPR4	Alpha-actinin-1 OS=Mus musculus GN=Actn1 PE=2 SV=1 - [ACTN1_MOUSE]	448,19	9,42	1	7	7	12	892	103,0	5,38
Q91VN6	Probable ATP-dependent RNA helicase DDX41 OS=Mus musculus GN=Ddx41 PE=1 SV=2 - [DDX41_MOUSE]	445,71	27,49	1	13	13	26	622	69,8	6,70
P11679	Keratin, type II cytoskeletal 8 OS=Mus musculus GN=Krt8 PE=1 SV=4 - [K2C8_MOUSE]	444,75	12,45	1	4	7	17	490	54,5	5,82
P12815	Programmed cell death protein 6 OS=Mus musculus GN=Pdcd6 PE=1 SV=2 - [PDCD6_MOUSE]	444,63	28,27	1	6	6	17	191	21,9	5,40
Q02257	Junction plakoglobin OS=Mus musculus GN=Jup PE=1 SV=3 - [PLAK_MOUSE]	443,80	18,12	1	11	11	19	745	81,7	6,14
Q9ERA6	Tuftelin-interacting protein 11 OS=Mus musculus GN=Tfip11 PE=1 SV=1 - [TFP11_MOUSE]	441,26	8,23	1	5	5	13	838	96,2	5,90
Q9CYL5	Golgi-associated plant pathogenesis-related protein 1 OS=Mus musculus GN=Glipr2 PE=2 SV=3 - [GAPR1_MOUSE]	441,09	31,17	1	3	3	9	154	17,1	9,51
Q03141	MAP/microtubule affinity-regulating kinase 3 OS=Mus musculus GN=Mark3 PE=1 SV=2 - [MARK3_MOUSE]	437,50	13,55	1	6	7	21	753	84,3	9,51
P42208	Septin-2 OS=Mus musculus GN=Sept2 PE=1 SV=2 - [SEPT2_MOUSE]	437,00	25,76	1	6	6	13	361	41,5	6,55
Q9CRA8	Exosome complex exonuclease RRP46 OS=Mus musculus GN=Exosc5 PE=1 SV=1 - [EXOS5_MOUSE]	436,85	17,02	1	4	4	11	235	25,2	7,56
P53986	Monocarboxylate transporter 1 OS=Mus musculus GN=Slc16a1 PE=1 SV=1 - [MOT1_MOUSE]	436,29	6,69	1	2	2	11	493	53,2	7,47
P45481	CREB-binding protein OS=Mus musculus GN=Crebbp PE=1 SV=3 - [CBP_MOUSE]	435,51	2,17	1	2	3	21	2441	265,3	8,54
P97371	Proteasome activator complex subunit 1 OS=Mus musculus GN=Psme1 PE=2 SV=2 - [PSME1_MOUSE]	434,53	29,32	1	6	6	14	249	28,7	5,97
B2RRE7	OTU domain-containing protein 4 OS=Mus musculus GN=Otud4 PE=1 SV=1 - [OTUD4_MOUSE]	433,37	5,24	1	4	4	21	1107	123,0	6,76
Q9D1G1	Ras-related protein Rab-1B OS=Mus musculus GN=Rab1b PE=1 SV=1 - [RAB1B_MOUSE]	431,87	38,81	1	4	6	20	201	22,2	5,73
Q8CJG0	Protein argonaute-2 OS=Mus musculus GN=Eif2c2 PE=1 SV=3 - [AGO2_MOUSE]	431,39	14,19	1	9	9	26	860	97,2	9,19
O54962	Barrier-to-autointegration factor OS=Mus musculus GN=Banf1 PE=1 SV=1 - [BAF_MOUSE]	431,29	43,82	1	4	4	19	89	10,1	6,09
Q9CQ22	RhoA activator C11orf59 homolog OS=Mus musculus PE=1 SV=1 - [CK059_MOUSE]	430,18	31,06	1	3	3	9	161	17,7	5,15
P01942	Hemoglobin subunit alpha OS=Mus musculus GN=Hba PE=1 SV=2 - [HBA_MOUSE]	430,14	36,62	1	4	4	17	142	15,1	8,22
P10107	Annexin A1 OS=Mus musculus GN=Anxa1 PE=1 SV=2 - [ANXA1_MOUSE]	429,71	22,54	1	5	5	8	346	38,7	7,37
P28843	Dipeptidyl peptidase 4 OS=Mus musculus GN=Dpp4 PE=1 SV=3 - [DPP4_MOUSE]	429,08	11,05	1	8	8	28	760	87,4	6,42
P29387	Guanine nucleotide-binding protein subunit beta-4 OS=Mus musculus GN=Gnb4 PE=2 SV=4 - [GBB4_MOUSE]	428,51	6,18	3	2	2	27	340	37,4	6,16
Q9ESX5	H/ACA ribonucleoprotein complex subunit 4 OS=Mus musculus GN=Dkc1 PE=1 SV=4 - [DKC1_MOUSE]	427,80	14,15	1	5	5	14	509	57,4	9,28
Q61233	Plastin-2 OS=Mus musculus GN=Lcp1 PE=1 SV=4 - [PLSL_MOUSE]	427,41	12,60	1	7	7	14	627	70,1	5,33
Q9ER69	Pre-mRNA-splicing regulator WTAP OS=Mus musculus GN=Wtap PE=2 SV=3 - [FL2D_MOUSE]	426,66	14,90	1	4	4	10	396	44,1	5,25
Q9JL26	Formin-like protein 1 OS=Mus musculus GN=Fmnl1 PE=1 SV=1 - [FMNL_MOUSE]	426,43	6,95	1	6	6	21	1094	122,0	5,82
P51881	ADP/ATP translocase 2 OS=Mus musculus GN=Slc25a5 PE=1 SV=3 - [ADT2_MOUSE]	426,07	17,79	1	4	5	22	298	32,9	9,73
Q505F5	Leucine-rich repeat-containing protein 47 OS=Mus musculus GN=Lrrc47 PE=1 SV=1 - [LRC47_MOUSE]	425,03	11,19	1	5	5	10	581	63,6	8,10
P15105	Glutamine synthetase OS=Mus musculus GN=Glul PE=1 SV=6 - [GLNA_MOUSE]	424,81	12,87	1	4	4	11	373	42,1	7,08
P97372	Proteasome activator complex subunit 2 OS=Mus musculus GN=Psme2 PE=2 SV=4 - [PSME2_MOUSE]	424,23	17,15	1	3	3	10	239	27,0	5,76
Q99J72	DNA dC->dU-editing enzyme APOBEC-3 OS=Mus musculus GN=Apobec3 PE=2 SV=2 - [ABEC3_MOUSE]	423,18	20,05	1	8	8	21	429	50,9	8,94
Q9JM76	Actin-related protein 2/3 complex subunit 3 OS=Mus musculus GN=Arpc3 PE=1 SV=3 - [ARPC3_MOUSE]	422,87	23,60	1	4	4	16	178	20,5	8,59
Q91VD9	NADH-ubiquinone oxidoreductase 75 kDa subunit, mitochondrial OS=Mus musculus GN=Ndufs1 PE=1 SV=2 - [NDUS1_MOUSE]	420,80	9,35	1	5	5	14	727	79,7	5,72
Q9R233	Tapasin OS=Mus musculus GN=Tapbp PE=2 SV=2 - [TPSN_MOUSE]	418,32	8,39	1	3	3	10	465	49,7	8,50
P53995	Anaphase-promoting complex subunit 1 OS=Mus musculus GN=Anapc1 PE=1 SV=2 - [APC1_MOUSE]	418,30	5,25	1	8	8	23	1944	215,9	6,35
Q8BZR9	Uncharacterized protein C17orf85 homolog OS=Mus musculus PE=1 SV=1 - [CQ085_MOUSE]	416,74	8,46	1	4	4	9	615	70,0	5,80
Q9D1R9	60S ribosomal protein L34 OS=Mus musculus GN=Rpl34 PE=3 SV=2 - [RL34_MOUSE]	415,58	25,64	1	6	6	33	117	13,3	11,47
O55013	Trafficking protein particle complex subunit 3 OS=Mus musculus GN=Trappc3 PE=1 SV=1 - [TPPC3_MOUSE]	414,64	30,56	1	5	5	18	180	20,3	4,96
Q3TBD2	Minor histocompatibility protein HA-1 OS=Mus musculus GN=Hmha1 PE=1 SV=2 - [HMHA1_MOUSE]	413,70	3,67	1	3	3	12	1116	122,8	5,94
Q8CIG8	Protein arginine N-methyltransferase 5 OS=Mus musculus GN=Prmt5 PE=1 SV=3 - [ANM5_MOUSE]	413,26	12,87	1	8	8	17	637	72,6	6,42
P02301	Histone H3.3C OS=Mus musculus GN=H3f3c PE=1 SV=3 - [H3C_MOUSE]	411,68	22,79	4	6	6	24	136	15,3	11,14
Q9D1M4	Eukaryotic translation elongation factor 1 epsilon-1 OS=Mus musculus GN=Eef1e1 PE=2 SV=1 - [MCA3_MOUSE]	409,79	56,90	1	9	9	22	174	19,8	8,59
P28656	Nucleosome assembly protein 1-like 1 OS=Mus musculus GN=Nap1l1 PE=1 SV=2 - [NP1L1_MOUSE]	409,48	17,65	1	5	7	20	391	45,3	4,46
Q8R146	Acylamino-acid-releasing enzyme OS=Mus musculus GN=Apeh PE=2 SV=3 - [APEH_MOUSE]	406,90	17,08	1	9	9	16	732	81,5	5,59
Q924K8	Metastasis-associated protein MTA3 OS=Mus musculus GN=Mta3 PE=1 SV=1 - [MTA3_MOUSE]	406,82	12,69	1	2	6	26	591	67,0	8,88
Q8CI33	CWF19-like protein 1 OS=Mus musculus GN=Cwf19l1 PE=2 SV=2 - [C19L1_MOUSE]	405,88	13,04	1	6	6	14	537	60,2	7,14
Q8BZX4	Splicing regulatory glutamine/lysine-rich protein 1 OS=Mus musculus GN=Srek1 PE=2 SV=1 - [SREK1_MOUSE]	405,80	14,78	1	4	4	8	494	56,7	10,48
Q8CI11	Guanine nucleotide-binding protein-like 3 OS=Mus musculus GN=Gnl3 PE=1 SV=2 - [GNL3_MOUSE]	405,50	15,99	1	6	6	22	538	60,7	9,11
Q9DB20	ATP synthase subunit O, mitochondrial OS=Mus musculus GN=Atp5o PE=1 SV=1 - [ATPO_MOUSE]	403,47	27,23	1	5	5	18	213	23,3	9,99
Q8C156	Condensin complex subunit 2 OS=Mus musculus GN=Ncaph PE=2 SV=1 - [CND2_MOUSE]	403,19	6,98	1	4	4	16	731	82,3	4,96
Q9JLV5	Cullin-3 OS=Mus musculus GN=Cul3 PE=1 SV=1 - [CUL3_MOUSE]	402,73	10,81	1	7	7	10	768	88,9	8,46
P37040	NADPH--cytochrome P450 reductase OS=Mus musculus GN=Por PE=1 SV=2 - [NCPR_MOUSE]	401,44	4,72	1	3	3	17	678	77,0	5,53
Q8BH43	Wiskott-Aldrich syndrome protein family member 2 OS=Mus musculus GN=Wasf2 PE=1 SV=1 - [WASF2_MOUSE]	399,86	16,50	1	8	8	13	497	54,0	5,53
Q78PY7	Staphylococcal nuclease domain-containing protein 1 OS=Mus musculus GN=Snd1 PE=1 SV=1 - [SND1_MOUSE]	399,55	8,46	1	6	6	13	910	102,0	7,43
Q99P72	Reticulon-4 OS=Mus musculus GN=Rtn4 PE=1 SV=2 - [RTN4_MOUSE]	398,56	12,13	1	6	6	35	1162	126,5	4,54
Q8VI94	2'-5'-oligoadenylate synthase-like protein 1 OS=Mus musculus GN=Oasl1 PE=2 SV=1 - [OASL1_MOUSE]	398,32	13,89	1	4	5	14	511	59,1	7,14
Q9QWV9	Cyclin-T1 OS=Mus musculus GN=Ccnt1 PE=1 SV=3 - [CCNT1_MOUSE]	398,23	13,67	1	6	6	13	724	80,5	8,68
Q99J95	Cell division protein kinase 9 OS=Mus musculus GN=Cdk9 PE=2 SV=1 - [CDK9_MOUSE]	397,09	14,52	1	4	5	17	372	42,7	8,79
Q8BGZ4	Cell division cycle protein 23 homolog OS=Mus musculus GN=Cdc23 PE=1 SV=2 - [CDC23_MOUSE]	397,07	16,42	1	9	9	14	597	68,5	7,18
Q9CXG3	Peptidyl-prolyl cis-trans isomerase-like 4 OS=Mus musculus GN=Ppil4 PE=2 SV=2 - [PPIL4_MOUSE]	395,84	12,80	1	5	5	13	492	57,2	6,11
Q9JLN9	Serine/threonine-protein kinase mTOR OS=Mus musculus GN=Mtor PE=1 SV=2 - [MTOR_MOUSE]	395,78	4,28	1	8	8	18	2549	288,6	7,17
Q62203	Splicing factor 3A subunit 2 OS=Mus musculus GN=Sf3a2 PE=2 SV=2 - [SF3A2_MOUSE]	394,46	15,58	1	5	5	17	475	49,9	9,54
P62858	40S ribosomal protein S28 OS=Mus musculus GN=Rps28 PE=2 SV=1 - [RS28_MOUSE]	393,29	52,17	1	5	5	19	69	7,8	10,70
P62259	14-3-3 protein epsilon OS=Mus musculus GN=Ywhae PE=1 SV=1 - [1433E_MOUSE]	393,04	35,29	1	6	9	19	255	29,2	4,74
P61205	ADP-ribosylation factor 3 OS=Mus musculus GN=Arf3 PE=2 SV=2 - [ARF3_MOUSE]	391,62	46,96	2	3	6	14	181	20,6	7,43
Q61550	Double-strand-break repair protein rad21 homolog OS=Mus musculus GN=Rad21 PE=1 SV=3 - [RAD21_MOUSE]	391,14	14,33	1	6	6	12	635	72,0	4,64
Q8BJY1	26S proteasome non-ATPase regulatory subunit 5 OS=Mus musculus GN=Psmd5 PE=2 SV=4 - [PSMD5_MOUSE]	387,90	28,77	1	11	13	24	504	55,9	5,21
Q8K3A9	7SK snRNA methylphosphate capping enzyme OS=Mus musculus GN=Mepce PE=1 SV=2 - [MEPCE_MOUSE]	387,07	15,77	1	7	7	18	666	72,0	9,25
P84091	AP-2 complex subunit mu OS=Mus musculus GN=Ap2m1 PE=1 SV=1 - [AP2M1_MOUSE]	386,69	22,76	1	8	8	30	435	49,6	9,54
Q9D2G2	Dihydrolipoyllysine-residue succinyltransferase component of 2-oxoglutarate dehydrogenase complex, mitochondrial OS=Mus musculus G	386,40	8,81	1	4	4	16	454	49,0	8,95
O35691	Pinin OS=Mus musculus GN=Pnn PE=1 SV=4 - [PININ_MOUSE]	386,32	10,21	1	7	7	24	725	82,4	7,01
P97760	DNA-directed RNA polymerase II subunit RPB3 OS=Mus musculus GN=Polr2c PE=2 SV=1 - [RPB3_MOUSE]	384,89	24,36	1	4	4	10	275	31,3	4,97
P05784	Keratin, type I cytoskeletal 18 OS=Mus musculus GN=Krt18 PE=1 SV=5 - [K1C18_MOUSE]	384,24	15,84	1	4	6	19	423	47,5	5,33
P68181	cAMP-dependent protein kinase catalytic subunit beta OS=Mus musculus GN=Prkacb PE=1 SV=2 - [KAPCB_MOUSE]	379,15	27,35	1	3	8	15	351	40,7	8,56
Q91ZA3	Propionyl-CoA carboxylase alpha chain, mitochondrial OS=Mus musculus GN=Pcca PE=2 SV=2 - [PCCA_MOUSE]	378,58	9,81	1	5	5	9	724	79,9	7,25
P61027	Ras-related protein Rab-10 OS=Mus musculus GN=Rab10 PE=1 SV=1 - [RAB10_MOUSE]	378,05	22,50	1	2	4	21	200	22,5	8,38
Q9WV55	Vesicle-associated membrane protein-associated protein A OS=Mus musculus GN=Vapa PE=1 SV=2 - [VAPA_MOUSE]	377,75	18,07	1	4	5	14	249	27,8	8,40
Q60872	Eukaryotic translation initiation factor 1A OS=Mus musculus GN=Eif1a PE=2 SV=3 - [IF1A_MOUSE]	376,69	26,39	2	4	4	15	144	16,5	5,24
Q8K224	N-acetyltransferase 10 OS=Mus musculus GN=Nat10 PE=2 SV=1 - [NAT10_MOUSE]	375,04	2,93	1	2	2	9	1024	115,3	8,32
P55258	Ras-related protein Rab-8A OS=Mus musculus GN=Rab8a PE=1 SV=2 - [RAB8A_MOUSE]	374,74	20,29	1	1	4	19	207	23,7	9,07
P61028	Ras-related protein Rab-8B OS=Mus musculus GN=Rab8b PE=1 SV=1 - [RAB8B_MOUSE]	373,71	27,05	1	2	5	19	207	23,6	9,07
P16254	Signal recognition particle 14 kDa protein OS=Mus musculus GN=Srp14 PE=1 SV=1 - [SRP14_MOUSE]	371,46	46,36	1	6	6	13	110	12,5	10,17
Q9DBC7	cAMP-dependent protein kinase type I-alpha regulatory subunit OS=Mus musculus GN=Prkar1a PE=1 SV=3 - [KAP0_MOUSE]	370,94	16,01	1	6	6	11	381	43,2	5,35
P52480	Pyruvate kinase isozymes M1/M2 OS=Mus musculus GN=Pkm2 PE=1 SV=4 - [KPYM_MOUSE]	369,65	23,16	1	8	8	21	531	57,8	7,47
Q9QZH3	Peptidyl-prolyl cis-trans isomerase E OS=Mus musculus GN=Ppie PE=2 SV=2 - [PPIE_MOUSE]	369,11	28,57	1	6	6	16	301	33,4	5,60
Q6PFR5	Transformer-2 protein homolog alpha OS=Mus musculus GN=Tra2a PE=1 SV=1 - [TRA2A_MOUSE]	367,07	12,10	1	3	3	13	281	32,3	11,28
P27641	X-ray repair cross-complementing protein 5 OS=Mus musculus GN=Xrcc5 PE=2 SV=4 - [XRCC5_MOUSE]	366,92	15,03	1	9	9	15	732	83,0	5,16
Q05816	Fatty acid-binding protein, epidermal OS=Mus musculus GN=Fabp5 PE=1 SV=3 - [FABP5_MOUSE]	363,00	22,22	1	3	3	12	135	15,1	6,54
Q8K0V4	CCR4-NOT transcription complex subunit 3 OS=Mus musculus GN=Cnot3 PE=1 SV=1 - [CNOT3_MOUSE]	362,73	9,85	1	6	6	11	751	81,9	6,20
Q61191	Host cell factor 1 OS=Mus musculus GN=Hcfc1 PE=1 SV=2 - [HCFC1_MOUSE]	361,93	3,67	1	6	6	14	2045	210,3	7,18
P61965	WD repeat-containing protein 5 OS=Mus musculus GN=Wdr5 PE=1 SV=1 - [WDR5_MOUSE]	360,94	12,28	1	3	3	8	334	36,6	8,27
P62141	Serine/threonine-protein phosphatase PP1-beta catalytic subunit OS=Mus musculus GN=Ppp1cb PE=1 SV=3 - [PP1B_MOUSE]	360,18	19,88	1	6	6	13	327	37,2	6,19
Q5U4D9	THO complex subunit 6 homolog OS=Mus musculus GN=Thoc6 PE=2 SV=1 - [THOC6_MOUSE]	359,58	26,69	1	7	7	13	341	37,3	7,12
Q9QXA5	U6 snRNA-associated Sm-like protein LSm4 OS=Mus musculus GN=Lsm4 PE=2 SV=1 - [LSM4_MOUSE]	358,53	25,55	1	4	4	17	137	15,1	10,05
Q9DCT2	NADH dehydrogenase [ubiquinone] iron-sulfur protein 3, mitochondrial OS=Mus musculus GN=Ndufs3 PE=1 SV=2 - [NDUS3_MOUSE]	357,59	24,71	1	5	5	8	263	30,1	7,17
P06151	L-lactate dehydrogenase A chain OS=Mus musculus GN=Ldha PE=1 SV=3 - [LDHA_MOUSE]	357,27	19,58	1	7	7	19	332	36,5	7,74
P63024	Vesicle-associated membrane protein 3 OS=Mus musculus GN=Vamp3 PE=1 SV=1 - [VAMP3_MOUSE]	355,68	23,30	2	2	2	8	103	11,5	8,50
Q0P678	Zinc finger CCCH domain-containing protein 18 OS=Mus musculus GN=Zc3h18 PE=1 SV=1 - [ZCH18_MOUSE]	355,29	12,55	1	6	6	16	948	105,6	7,80
P00405	Cytochrome c oxidase subunit 2 OS=Mus musculus GN=Mtco2 PE=1 SV=1 - [COX2_MOUSE]	355,24	20,26	1	3	3	15	227	26,0	4,73
Q9JJZ6	Krueppel-like factor 13 OS=Mus musculus GN=Klf13 PE=2 SV=2 - [KLF13_MOUSE]	353,53	17,30	1	3	3	8	289	31,1	9,64
O70591	Prefoldin subunit 2 OS=Mus musculus GN=Pfdn2 PE=2 SV=2 - [PFD2_MOUSE]	352,17	16,88	1	2	2	6	154	16,5	6,58
Q9CQA1	Trafficking protein particle complex subunit 5 OS=Mus musculus GN=Trappc5 PE=1 SV=1 - [TPPC5_MOUSE]	348,71	31,91	1	6	6	11	188	20,8	9,66

P63037	DnaJ homolog subfamily A member 1 OS=Mus musculus GN=Dnaja1 PE=1 SV=1 - [DNJA1_MOUSE]	347,99	23,17	1	5	6	12	397	44,8	7,08
Q09044	Synaptosomal-associated protein 23 OS=Mus musculus GN=Snap23 PE=1 SV=1 - [SNP23_MOUSE]	346,75	43,33	1	6	6	11	210	23,2	4,98
D3YXK2	Scaffold attachment factor B1 OS=Mus musculus GN=Safb PE=2 SV=2 - [SAFB1_MOUSE]	346,52	5,55	1	2	3	12	937	105,0	5,35
Q8CFE3	REST corepressor 1 OS=Mus musculus GN=Rcor1 PE=1 SV=2 - [RCOR1_MOUSE]	344,82	13,21	1	5	5	10	477	52,5	7,03
Q923D5	WW domain-binding protein 11 OS=Mus musculus GN=Wbp11 PE=1 SV=2 - [WBP11_MOUSE]	344,71	14,82	1	8	8	16	641	69,8	8,40
O70439	Syntaxin-7 OS=Mus musculus GN=Stx7 PE=1 SV=3 - [STX7_MOUSE]	344,38	16,09	1	3	3	12	261	29,8	5,78
Q9JKV1	Proteasomal ubiquitin receptor ADRM1 OS=Mus musculus GN=Adrm1 PE=1 SV=2 - [ADRM1_MOUSE]	344,06	7,86	1	4	4	32	407	42,0	5,07
Q99M31	Heat shock 70 kDa protein 14 OS=Mus musculus GN=Hspa14 PE=2 SV=2 - [HSP7E_MOUSE]	343,55	12,57	1	5	5	9	509	54,6	5,92
P52432	DNA-directed RNA polymerases I and III subunit RPAC1 OS=Mus musculus GN=Polr1c PE=1 SV=3 - [RPAC1_MOUSE]	342,73	19,65	1	5	5	10	346	39,1	5,21
Q9JHJ0	Tropomodulin-3 OS=Mus musculus GN=Tmod3 PE=1 SV=1 - [TMOD3_MOUSE]	342,40	22,16	1	6	6	11	352	39,5	5,14
O88544	COP9 signalosome complex subunit 4 OS=Mus musculus GN=Cops4 PE=1 SV=1 - [CSN4_MOUSE]	341,68	15,02	1	5	5	10	406	46,3	5,83
Q923T9	Calcium/calmodulin-dependent protein kinase type II subunit gamma OS=Mus musculus GN=Camk2g PE=1 SV=1 - [KCC2G_MOUSE]	340,99	10,02	1	2	4	11	529	59,6	7,58
P09055	Integrin beta-1 OS=Mus musculus GN=Itgb1 PE=1 SV=1 - [ITB1_MOUSE]	338,60	7,77	1	6	6	20	798	88,2	5,94
Q8BKT7	THO complex subunit 5 homolog OS=Mus musculus GN=Thoc5 PE=1 SV=2 - [THOC5_MOUSE]	337,77	10,98	1	7	7	14	683	78,6	7,12
Q9WTX2	Interferon-inducible double stranded RNA-dependent protein kinase activator A OS=Mus musculus GN=Prkra PE=1 SV=1 - [PRKRA_MOUSE	337,70	15,97	1	4	4	18	313	34,3	8,43
P61750	ADP-ribosylation factor 4 OS=Mus musculus GN=Arf4 PE=2 SV=2 - [ARF4_MOUSE]	336,26	42,78	1	2	5	12	180	20,4	7,14
Q3TZX8	Polynucleotide 5'-hydroxyl-kinase NOL9 OS=Mus musculus GN=Nol9 PE=2 SV=1 - [NOL9_MOUSE]	336,00	4,48	1	4	4	15	714	80,8	9,45
P63242	Eukaryotic translation initiation factor 5A-1 OS=Mus musculus GN=Eif5a PE=1 SV=2 - [IF5A1_MOUSE]	335,86	33,77	1	4	4	15	154	16,8	5,24
P01786	Ig heavy chain V region MOPC 47A OS=Mus musculus PE=1 SV=1 - [HVM17_MOUSE]	335,56	21,37	1	2	2	7	117	13,0	8,84
Q6P8I4	PEST proteolytic signal-containing nuclear protein OS=Mus musculus GN=Pcnp PE=1 SV=1 - [PCNP_MOUSE]	335,09	15,73	1	2	2	11	178	19,0	7,49
Q5SSI6	U3 small nucleolar RNA-associated protein 18 homolog OS=Mus musculus GN=Utp18 PE=1 SV=1 - [UTP18_MOUSE]	333,92	10,87	1	4	4	9	552	61,2	8,78
Q6RT24	Centromere-associated protein E OS=Mus musculus GN=Cenpe PE=1 SV=1 - [CENPE_MOUSE]	333,30	1,82	1	3	3	9	2474	286,3	5,31
P05132	cAMP-dependent protein kinase catalytic subunit alpha OS=Mus musculus GN=Prkaca PE=1 SV=3 - [KAPCA_MOUSE]	332,57	27,35	1	3	8	14	351	40,5	8,79
Q9CY50	Translocon-associated protein subunit alpha OS=Mus musculus GN=Ssr1 PE=1 SV=1 - [SSRA_MOUSE]	332,30	11,89	1	3	3	9	286	32,0	4,45
Q9D7Z3	Nucleolar protein 7 OS=Mus musculus GN=Nol7 PE=1 SV=1 - [NOL7_MOUSE]	331,44	18,90	1	4	4	7	254	29,0	9,61
Q8C6G8	WD repeat-containing protein 26 OS=Mus musculus GN=Wdr26 PE=1 SV=3 - [WDR26_MOUSE]	331,27	7,64	1	5	5	14	641	70,5	6,16
Q9Z2D8	Methyl-CpG-binding domain protein 3 OS=Mus musculus GN=Mbd3 PE=1 SV=1 - [MBD3_MOUSE]	330,33	14,04	1	4	4	10	285	32,1	5,82
Q8C854	Myelin expression factor 2 OS=Mus musculus GN=Myef2 PE=1 SV=1 - [MYEF2_MOUSE]	329,69	10,49	1	6	6	16	591	63,3	8,87
Q4VC33	Macrophage erythroblast attacher OS=Mus musculus GN=Maea PE=1 SV=1 - [MAEA_MOUSE]	329,65	22,98	1	7	7	12	396	45,3	8,69
P61021	Ras-related protein Rab-5B OS=Mus musculus GN=Rab5b PE=1 SV=1 - [RAB5B_MOUSE]	329,01	27,91	1	2	5	16	215	23,7	8,13
Q02105	Complement C1q subcomponent subunit C OS=Mus musculus GN=C1qc PE=2 SV=2 - [C1QC_MOUSE]	328,09	20,33	1	4	4	11	246	26,0	8,54
Q9D0R2	Threonyl-tRNA synthetase, cytoplasmic OS=Mus musculus GN=Tars PE=1 SV=2 - [SYTC_MOUSE]	325,23	14,27	1	9	9	17	722	83,3	7,36
Q8R016	Bleomycin hydrolase OS=Mus musculus GN=Blmh PE=2 SV=1 - [BLMH_MOUSE]	324,99	12,09	1	3	4	21	455	52,5	6,48
Q8BJ05	Zinc finger CCCH domain-containing protein 14 OS=Mus musculus GN=Zc3h14 PE=1 SV=1 - [ZC3HE_MOUSE]	324,17	10,48	1	5	5	19	735	82,4	7,37
Q80T69	Round spermatid basic protein 1 OS=Mus musculus GN=Rsbn1 PE=1 SV=3 - [RSBN1_MOUSE]	324,11	4,91	1	3	3	8	795	89,2	8,65
Q61103	Zinc finger protein ubi-d4 OS=Mus musculus GN=Dpf2 PE=1 SV=1 - [REQU_MOUSE]	324,09	11,76	1	3	3	5	391	44,2	6,47
P62627	Dynein light chain roadblock-type 1 OS=Mus musculus GN=Dynlrb1 PE=1 SV=3 - [DLRB1_MOUSE]	323,05	55,21	1	4	4	11	96	11,0	7,25
O55100	Synaptogyrin-1 OS=Mus musculus GN=Syngr1 PE=1 SV=2 - [SNG1_MOUSE]	323,04	10,26	1	2	2	21	234	25,6	4,65
Q9CXL3	Uncharacterized protein C7orf50 homolog OS=Mus musculus PE=2 SV=3 - [CG050_MOUSE]	322,17	45,13	1	5	5	10	195	22,2	9,39
Q9CQU1	Microfibrillar-associated protein 1 OS=Mus musculus GN=Mfap1 PE=1 SV=1 - [MFAP1_MOUSE]	320,86	12,76	1	5	5	16	439	51,9	4,98
Q9D903	Probable rRNA-processing protein EBP2 OS=Mus musculus GN=Ebna1bp2 PE=2 SV=1 - [EBP2_MOUSE]	319,98	21,57	1	5	5	10	306	34,7	10,08
Q91ZJ5	UTP--glucose-1-phosphate uridylyltransferase OS=Mus musculus GN=Ugp2 PE=2 SV=3 - [UGPA_MOUSE]	319,88	11,42	1	4	4	8	508	56,9	7,61
Q8BG81	Polymerase delta-interacting protein 3 OS=Mus musculus GN=Poldip3 PE=2 SV=1 - [PDIP3_MOUSE]	319,40	18,33	1	5	5	16	420	46,1	10,05
Q99PT1	Rho GDP-dissociation inhibitor 1 OS=Mus musculus GN=Arhgdia PE=1 SV=3 - [GDIR1_MOUSE]	319,09	22,06	1	3	3	9	204	23,4	5,20
P68510	14-3-3 protein eta OS=Mus musculus GN=Ywhah PE=1 SV=2 - [1433F_MOUSE]	316,17	23,58	1	2	6	16	246	28,2	4,89
Q01768	Nucleoside diphosphate kinase B OS=Mus musculus GN=Nme2 PE=1 SV=1 - [NDKB_MOUSE]	314,77	40,79	1	2	5	19	152	17,4	7,50
P63005	Platelet-activating factor acetylhydrolase IB subunit alpha OS=Mus musculus GN=Pafah1b1 PE=1 SV=2 - [LIS1_MOUSE]	314,12	13,41	1	3	4	12	410	46,6	7,37
P01887	Beta-2-microglobulin OS=Mus musculus GN=B2m PE=1 SV=2 - [B2MG_MOUSE]	312,48	22,69	1	3	3	18	119	13,8	8,44
Q9Z2N8	Actin-like protein 6A OS=Mus musculus GN=Actl6a PE=1 SV=2 - [ACL6A_MOUSE]	311,35	22,61	1	6	6	11	429	47,4	5,60
Q9Z1R2	Large proline-rich protein BAT3 OS=Mus musculus GN=Bat3 PE=1 SV=1 - [BAT3_MOUSE]	309,97	5,63	1	3	3	12	1154	121,0	5,71
Q8K212	Phosphofurin acidic cluster sorting protein 1 OS=Mus musculus GN=Pacs1 PE=1 SV=2 - [PACS1_MOUSE]	309,73	15,61	1	10	10	16	961	104,8	7,74
O35892	Nuclear autoantigen Sp-100 OS=Mus musculus GN=Sp100 PE=1 SV=2 - [SP100_MOUSE]	309,53	12,66	1	6	6	13	482	54,7	8,66
Q8R2T8	General transcription factor 3C polypeptide 5 OS=Mus musculus GN=Gtf3c5 PE=2 SV=2 - [TF3C5_MOUSE]	309,28	8,08	1	4	4	9	520	60,5	6,73
Q9ERH4	Nucleolar and spindle-associated protein 1 OS=Mus musculus GN=Nusap1 PE=1 SV=1 - [NUSAP_MOUSE]	308,38	9,84	1	3	3	13	427	48,5	9,89
Q6PA06	Atlastin-2 OS=Mus musculus GN=Atl2 PE=1 SV=1 - [ATLA2_MOUSE]	308,04	9,43	1	4	4	13	583	66,2	5,43
Q91YD3	mRNA-decapping enzyme 1A OS=Mus musculus GN=Dcp1a PE=1 SV=1 - [DCP1A_MOUSE]	307,33	14,29	1	6	6	11	602	65,2	6,99
Q9CPW4	Actin-related protein 2/3 complex subunit 5 OS=Mus musculus GN=Arpc5 PE=2 SV=3 - [ARPC5_MOUSE]	305,82	26,49	1	3	3	7	151	16,3	5,67
O54781	Serine/threonine-protein kinase SRPK2 OS=Mus musculus GN=Srpk2 PE=1 SV=2 - [SRPK2_MOUSE]	305,71	8,08	1	3	5	16	681	76,7	4,91
Q06185	ATP synthase subunit e, mitochondrial OS=Mus musculus GN=Atp5i PE=1 SV=2 - [ATP5I_MOUSE]	305,58	60,56	1	5	5	14	71	8,2	9,35
Q8VDV8	MIT domain-containing protein 1 OS=Mus musculus GN=Mitd1 PE=1 SV=1 - [MITD1_MOUSE]	304,64	17,27	1	4	4	7	249	28,8	8,06
Q9JLV6	Bifunctional polynucleotide phosphatase/kinase OS=Mus musculus GN=Pnkp PE=1 SV=2 - [PNKP_MOUSE]	304,41	7,28	1	3	3	8	522	57,2	7,90
Q8R5H1	Ubiquitin carboxyl-terminal hydrolase 15 OS=Mus musculus GN=Usp15 PE=2 SV=1 - [UBP15_MOUSE]	303,36	9,28	1	8	8	15	981	112,3	5,17
Q91WM1	Spermatid perinuclear RNA-binding protein OS=Mus musculus GN=Strbp PE=1 SV=1 - [STRBP_MOUSE]	300,54	11,46	1	4	7	16	672	73,7	8,72
Q8C1B7	Septin-11 OS=Mus musculus GN=Sept11 PE=1 SV=4 - [SEP11_MOUSE]	300,35	19,26	1	4	7	17	431	49,7	6,68
Q60749	KH domain-containing, RNA-binding, signal transduction-associated protein 1 OS=Mus musculus GN=Khdrbs1 PE=1 SV=2 - [KHDR1_MOUSE	297,51	7,45	1	3	3	24	443	48,3	8,72
P10639	Thioredoxin OS=Mus musculus GN=Txn PE=1 SV=3 - [THIO_MOUSE]	297,29	31,43	1	3	3	7	105	11,7	4,92
Q8C9B9	Death-inducer obliterator 1 OS=Mus musculus GN=Dido1 PE=1 SV=4 - [DIDO1_MOUSE]	297,21	2,44	1	3	3	8	2256	247,0	7,91
Q8C5L3	CCR4-NOT transcription complex subunit 2 OS=Mus musculus GN=Cnot2 PE=2 SV=2 - [CNOT2_MOUSE]	296,01	6,11	1	2	2	12	540	59,7	7,66
Q6ZPZ3	Zinc finger CCCH domain-containing protein 4 OS=Mus musculus GN=Zc3h4 PE=1 SV=2 - [ZC3H4_MOUSE]	295,99	13,57	1	9	9	28	1304	140,9	6,27
Q9EP97	Sentrin-specific protease 3 OS=Mus musculus GN=Senp3 PE=1 SV=1 - [SENP3_MOUSE]	294,92	11,09	1	4	4	8	568	64,4	8,75
P50518	V-type proton ATPase subunit E 1 OS=Mus musculus GN=Atp6v1e1 PE=1 SV=2 - [VATE1_MOUSE]	294,50	21,24	1	4	4	11	226	26,1	8,43
Q99LC8	Translation initiation factor eIF-2B subunit alpha OS=Mus musculus GN=Eif2b1 PE=2 SV=1 - [EI2BA_MOUSE]	291,53	19,67	1	4	4	8	305	33,8	8,32
Q61598	Rab GDP dissociation inhibitor beta OS=Mus musculus GN=Gdi2 PE=1 SV=1 - [GDIB_MOUSE]	291,30	17,75	1	6	6	13	445	50,5	6,25
Q99LB2	Dehydrogenase/reductase SDR family member 4 OS=Mus musculus GN=Dhrs4 PE=2 SV=3 - [DHRS4_MOUSE]	290,85	15,41	1	5	5	10	279	29,9	9,38
Q61136	Serine/threonine-protein kinase PRP4 homolog OS=Mus musculus GN=Prpf4b PE=1 SV=3 - [PRP4B_MOUSE]	290,66	6,95	1	5	6	10	1007	116,9	10,23
Q6PHN9	Ras-related protein Rab-35 OS=Mus musculus GN=Rab35 PE=1 SV=1 - [RAB35_MOUSE]	290,64	18,41	1	1	3	15	201	23,0	8,29
P57716	Nicastrin OS=Mus musculus GN=Ncstn PE=1 SV=3 - [NICA_MOUSE]	290,43	7,91	1	5	5	9	708	78,4	6,09
Q9CWJ9	Bifunctional purine biosynthesis protein PURH OS=Mus musculus GN=Atic PE=1 SV=2 - [PUR9_MOUSE]	290,34	18,58	1	8	8	12	592	64,2	6,76
Q8K194	U4/U6.U5 small nuclear ribonucleoprotein 27 kDa protein OS=Mus musculus GN=Snrnp27 PE=2 SV=1 - [SNR27_MOUSE]	290,23	17,42	1	3	3	10	155	18,9	11,62
Q9WTX5	S-phase kinase-associated protein 1 OS=Mus musculus GN=Skp1 PE=1 SV=3 - [SKP1_MOUSE]	289,02	30,06	1	5	5	8	163	18,7	4,54
Q9CZ13	Cytochrome b-c1 complex subunit 1, mitochondrial OS=Mus musculus GN=Uqcrc1 PE=1 SV=2 - [QCR1_MOUSE]	287,41	17,71	1	9	9	17	480	52,8	6,21
P18760	Cofilin-1 OS=Mus musculus GN=Cfl1 PE=1 SV=3 - [COF1_MOUSE]	284,97	31,93	1	3	3	11	166	18,5	8,09
Q8R3C0	Mini-chromosome maintenance complex-binding protein OS=Mus musculus GN=Mcmbp PE=2 SV=1 - [MCMBP_MOUSE]	284,81	7,79	1	4	4	6	642	72,8	5,66
P60824	Cold-inducible RNA-binding protein OS=Mus musculus GN=Cirbp PE=1 SV=1 - [CIRBP_MOUSE]	284,01	27,33	1	4	4	8	172	18,6	9,61
Q8BQZ5	Cleavage and polyadenylation specificity factor subunit 4 OS=Mus musculus GN=Cpsf4 PE=2 SV=1 - [CPSF4_MOUSE]	283,79	9,00	1	2	2	4	211	23,6	8,47
Q6KCD5	Nipped-B-like protein OS=Mus musculus GN=Nipbl PE=1 SV=1 - [NIPBL_MOUSE]	283,69	1,36	1	3	3	6	2798	315,3	7,91
Q9WV32	Actin-related protein 2/3 complex subunit 1B OS=Mus musculus GN=Arpc1b PE=1 SV=4 - [ARC1B_MOUSE]	282,13	7,80	1	3	3	8	372	41,0	8,35
P61327	Protein mago nashi homolog OS=Mus musculus GN=Magoh PE=2 SV=1 - [MGN_MOUSE]	281,77	28,08	2	4	4	10	146	17,2	6,11
Q9Z1N5	Spliceosome RNA helicase Bat1 OS=Mus musculus GN=Bat1 PE=1 SV=1 - [UAP56_MOUSE]	281,71	16,82	1	7	7	15	428	49,0	5,67
Q9JM93	ADP-ribosylation factor-like protein 6-interacting protein 4 OS=Mus musculus GN=Arl6ip4 PE=1 SV=1 - [AR6P4_MOUSE]	280,98	12,23	1	2	2	5	229	25,5	11,19
Q9EQH3	Vacuolar protein sorting-associated protein 35 OS=Mus musculus GN=Vps35 PE=1 SV=1 - [VPS35_MOUSE]	280,60	11,43	1	7	7	12	796	91,7	5,44
Q80X90	Filamin-B OS=Mus musculus GN=Flnb PE=1 SV=3 - [FLNB_MOUSE]	280,56	1,84	1	2	4	9	2602	277,7	5,71
P50247	Adenosylhomocysteinase OS=Mus musculus GN=Ahcy PE=1 SV=3 - [SAHH_MOUSE]	280,46	14,35	1	6	6	10	432	47,7	6,54
Q80UW8	DNA-directed RNA polymerases I, II, and III subunit RPABC1 OS=Mus musculus GN=Polr2e PE=2 SV=1 - [RPAB1_MOUSE]	280,31	19,52	1	4	4	10	210	24,6	5,95
P63038	60 kDa heat shock protein, mitochondrial OS=Mus musculus GN=Hspd1 PE=1 SV=1 - [CH60_MOUSE]	279,98	12,74	1	5	5	9	573	60,9	6,18
P58854	Gamma-tubulin complex component 3 OS=Mus musculus GN=Tubgcp3 PE=2 SV=2 - [GCP3_MOUSE]	278,62	9,61	1	7	7	10	905	103,4	8,32
Q91YE7	RNA-binding protein 5 OS=Mus musculus GN=Rbm5 PE=1 SV=1 - [RBM5_MOUSE]	277,49	4,66	1	3	3	6	815	92,3	6,21
Q7TQK1	Integrator complex subunit 7 OS=Mus musculus GN=Ints7 PE=1 SV=1 - [INT7_MOUSE]	276,60	8,90	1	7	7	10	966	106,8	8,22
O70325	Phospholipid hydroperoxide glutathione peroxidase, mitochondrial OS=Mus musculus GN=Gpx4 PE=1 SV=4 - [GPX41_MOUSE]	276,27	22,84	1	4	4	11	197	22,2	8,46
Q99MJ9	ATP-dependent RNA helicase DDX50 OS=Mus musculus GN=Ddx50 PE=2 SV=1 - [DDX50_MOUSE]	276,23	8,86	1	5	5	11	734	82,1	9,25
O35900	U6 snRNA-associated Sm-like protein LSm2 OS=Mus musculus GN=Lsm2 PE=2 SV=1 - [LSM2_MOUSE]	275,71	28,42	1	2	2	7	95	10,8	6,52
Q8CHP5	Partner of Y14 and mago OS=Mus musculus GN=Wibg PE=1 SV=2 - [WIBG_MOUSE]	275,39	28,08	1	3	3	14	203	22,7	9,50
Q62426	Cystatin-B OS=Mus musculus GN=Cstb PE=1 SV=1 - [CYTB_MOUSE]	272,56	41,84	1	3	3	10	98	11,0	7,39
Q9Z0H8	CAP-Gly domain-containing linker protein 2 OS=Mus musculus GN=Clip2 PE=1 SV=2 - [CLIP2_MOUSE]	272,13	9,55	1	7	7	22	1047	115,8	6,48
Q9CPQ8	ATP synthase subunit g, mitochondrial OS=Mus musculus GN=Atp5l PE=1 SV=1 - [ATP5L_MOUSE]	270,93	37,86	1	3	3	10	103	11,4	9,74
P15532	Nucleoside diphosphate kinase A OS=Mus musculus GN=Nme1 PE=1 SV=1 - [NDKA_MOUSE]	269,51	38,16	1	2	5	17	152	17,2	7,37
Q8R1N0	Zinc finger protein 830 OS=Mus musculus GN=Znf830 PE=1 SV=1 - [ZN830_MOUSE]	268,91	13,77	1	3	3	8	363	40,6	5,40
Q9CQE6	Histone chaperone ASF1A OS=Mus musculus GN=Asf1a PE=2 SV=1 - [ASF1A_MOUSE]	268,65	16,67	1	2	3	8	204	22,9	4,41
Q01405	Protein transport protein Sec23A OS=Mus musculus GN=Sec23a PE=1 SV=2 - [SC23A_MOUSE]	268,47	7,32	1	4	4	8	765	86,1	7,08
Q7TMQ7	WD repeat-containing protein 91 OS=Mus musculus GN=Wdr91 PE=1 SV=1 - [WDR91_MOUSE]	268,31	6,68	1	4	4	13	748	83,4	6,64
Q9CYA6	Zinc finger CCHC domain-containing protein 8 OS=Mus musculus GN=Zcchc8 PE=2 SV=3 - [ZCHC8_MOUSE]	267,22	9,59	1	4	4	11	709	78,0	4,73
P55194	SH3 domain-binding protein 1 OS=Mus musculus GN=Sh3bp1 PE=1 SV=2 - [3BP1_MOUSE]	266,95	5,82	1	2	3	7	601	65,2	5,68
Q60520	Paired amphipathic helix protein Sin3a OS=Mus musculus GN=Sin3a PE=1 SV=3 - [SIN3A_MOUSE]	265,75	6,83	1	7	7	12	1274	145,0	7,25
P84096	Rho-related GTP-binding protein RhoG OS=Mus musculus GN=Rhog PE=2 SV=1 - [RHOG_MOUSE]	265,51	24,08	1	3	3	11	191	21,3	8,12
Q80TJ7	Histone lysine demethylase PHF8 OS=Mus musculus GN=Phf8 PE=1 SV=2 - [PHF8_MOUSE]	263,73	3,32	1	1	2	4	1023	113,5	8,44
O55135	Eukaryotic translation initiation factor 6 OS=Mus musculus GN=Eif6 PE=1 SV=2 - [IF6_MOUSE]	262,55	17,14	1	3	3	16	245	26,5	4,74
P97313	DNA-dependent protein kinase catalytic subunit OS=Mus musculus GN=Prkdc PE=1 SV=3 - [PRKDC_MOUSE]	262,43	1,65	1	6	6	10	4128	471,2	7,12
Q9D0M0	Exosome complex exonuclease RRP42 OS=Mus musculus GN=Exosc7 PE=2 SV=2 - [EXOS7_MOUSE]	259,80	15,81	1	3	3	8	291	31,8	5,19
Q5SSW2	Proteasome activator complex subunit 4 OS=Mus musculus GN=Psme4 PE=1 SV=1 - [PSME4_MOUSE]	259,54	3,20	1	5	5	14	1843	211,1	7,01
Q61548	Clathrin coat assembly protein AP180 OS=Mus musculus GN=Snap91 PE=1 SV=1 - [AP180_MOUSE]	259,43	3,88	1	3	3	6	901	91,8	4,88
P83940	Transcription elongation factor B polypeptide 1 OS=Mus musculus GN=Tceb1 PE=1 SV=1 - [ELOC_MOUSE]	258,90	29,46	1	3	3	7	112	12,5	4,78

P10518	Delta-aminolevulinic acid dehydratase OS=Mus musculus GN=Alad PE=1 SV=1 - [HEM2_MOUSE]	257,46	16,67	1	4	4	7	330	36,0	6,79
Q9JL16	Interferon-stimulated gene 20 kDa protein OS=Mus musculus GN=Isg20 PE=1 SV=1 - [ISG20_MOUSE]	256,42	18,33	1	5	5	13	300	32,5	8,15
P68404	Protein kinase C beta type OS=Mus musculus GN=Prkcb PE=1 SV=4 - [KPCB_MOUSE]	256,30	7,75	1	5	5	9	671	76,7	7,01
Q62186	Translocon-associated protein subunit delta OS=Mus musculus GN=Ssr4 PE=2 SV=1 - [SSRD_MOUSE]	255,39	13,95	1	2	2	6	172	18,9	5,78
Q8R3C6	Probable RNA-binding protein 19 OS=Mus musculus GN=Rbm19 PE=1 SV=1 - [RBM19_MOUSE]	254,87	3,05	1	2	2	3	952	106,0	6,57
Q9D0T1	NHP2-like protein 1 OS=Mus musculus GN=Nhp2l1 PE=2 SV=4 - [NH2L1_MOUSE]	254,14	23,44	1	4	4	9	128	14,2	8,46
O88890	SH2 domain-containing protein 1A OS=Mus musculus GN=Sh2d1a PE=1 SV=1 - [SH21A_MOUSE]	253,83	44,44	1	3	3	6	126	13,9	8,59
Q99MU3	Double-stranded RNA-specific adenosine deaminase OS=Mus musculus GN=Adar PE=1 SV=2 - [DSRAD_MOUSE]	252,26	12,31	1	9	10	20	1178	130,4	8,70
Q9D1M0	Protein SEC13 homolog OS=Mus musculus GN=Sec13 PE=2 SV=3 - [SEC13_MOUSE]	251,84	8,70	1	2	2	4	322	35,5	5,38
Q9CQQ7	ATP synthase subunit b, mitochondrial OS=Mus musculus GN=Atp5f1 PE=1 SV=1 - [AT5F1_MOUSE]	251,69	13,67	1	3	3	7	256	28,9	9,06
Q6PCM2	Integrator complex subunit 6 OS=Mus musculus GN=Ints6 PE=2 SV=1 - [INT6_MOUSE]	251,36	4,19	1	3	3	5	883	99,6	8,78
P24547	Inosine-5'-monophosphate dehydrogenase 2 OS=Mus musculus GN=Impdh2 PE=1 SV=2 - [IMDH2_MOUSE]	250,86	13,04	1	6	6	12	514	55,8	7,28
Q9JKX4	Protein AATF OS=Mus musculus GN=Aatf PE=1 SV=1 - [AATF_MOUSE]	249,58	6,65	1	3	3	6	526	59,4	4,93
P52293	Importin subunit alpha-2 OS=Mus musculus GN=Kpna2 PE=1 SV=2 - [IMA2_MOUSE]	248,76	9,07	1	3	3	6	529	57,9	5,68
Q6ZQI3	Malectin OS=Mus musculus GN=Mlec PE=2 SV=2 - [MLEC_MOUSE]	248,51	16,49	1	3	3	6	291	32,3	6,05
Q78ZA7	Nucleosome assembly protein 1-like 4 OS=Mus musculus GN=Nap1l4 PE=1 SV=1 - [NP1L4_MOUSE]	248,50	17,33	1	4	6	13	375	42,7	4,67
P06745	Glucose-6-phosphate isomerase OS=Mus musculus GN=Gpi PE=1 SV=4 - [G6PI_MOUSE]	248,32	10,39	1	4	4	4	558	62,7	8,13
O70404	Vesicle-associated membrane protein 8 OS=Mus musculus GN=Vamp8 PE=1 SV=1 - [VAMP8_MOUSE]	248,03	32,67	1	4	4	19	101	11,4	8,19
Q9WVM1	Rac GTPase-activating protein 1 OS=Mus musculus GN=Racgap1 PE=2 SV=1 - [RGAP1_MOUSE]	247,23	7,96	1	4	4	9	628	70,1	8,51
Q9JMK2	Casein kinase I isoform epsilon OS=Mus musculus GN=Csnk1e PE=1 SV=2 - [KC1E_MOUSE]	246,96	18,51	1	6	6	10	416	47,3	9,66
Q8BIQ5	Cleavage stimulation factor subunit 2 OS=Mus musculus GN=Cstf2 PE=1 SV=2 - [CSTF2_MOUSE]	246,59	7,76	1	4	4	16	580	61,3	6,83
Q61768	Kinesin-1 heavy chain OS=Mus musculus GN=Kif5b PE=1 SV=3 - [KINH_MOUSE]	246,45	10,59	1	8	8	12	963	109,5	6,44
Q9DCA5	Ribosome biogenesis protein BRX1 homolog OS=Mus musculus GN=Brix1 PE=2 SV=3 - [BRX1_MOUSE]	246,40	15,58	1	5	5	10	353	41,2	10,02
O54825	Bystin OS=Mus musculus GN=Bysl PE=1 SV=3 - [BYST_MOUSE]	246,40	13,53	1	5	5	9	436	49,8	7,78
P62869	Transcription elongation factor B polypeptide 2 OS=Mus musculus GN=Tceb2 PE=1 SV=1 - [ELOB_MOUSE]	246,05	49,15	1	4	4	6	118	13,2	5,01
Q8VHK9	Probable ATP-dependent RNA helicase DHX36 OS=Mus musculus GN=Dhx36 PE=2 SV=2 - [DHX36_MOUSE]	245,71	9,39	1	6	7	10	1001	113,8	8,29
Q9JJ80	Ribosome production factor 2 homolog OS=Mus musculus GN=Rpf2 PE=2 SV=2 - [RPF2_MOUSE]	245,21	11,11	1	3	3	6	306	35,3	10,04
Q3UQA7	Selenoprotein H OS=Mus musculus GN=Selh PE=2 SV=2 - [SELH_MOUSE]	245,20	63,79	1	6	6	16	116	13,0	9,80
Q8K1B8	Fermitin family homolog 3 OS=Mus musculus GN=Fermt3 PE=1 SV=1 - [URP2_MOUSE]	245,02	7,22	1	3	3	6	665	75,6	7,05
Q8CE46	Pseudouridylate synthase 7 homolog-like protein OS=Mus musculus GN=Pus7l PE=1 SV=1 - [PUS7L_MOUSE]	244,99	10,97	1	7	7	10	702	79,1	7,81
Q9DCG9	tRNA methyltransferase 112 homolog OS=Mus musculus GN=Trmt112 PE=2 SV=1 - [TR112_MOUSE]	244,51	31,20	1	3	3	9	125	14,1	5,27
Q9Z2F2	2'-5'-oligoadenylate synthase-like protein 2 OS=Mus musculus GN=Oasl2 PE=1 SV=2 - [OASL2_MOUSE]	244,45	19,09	1	6	8	20	508	58,7	6,40
Q64337	Sequestosome-1 OS=Mus musculus GN=Sqstm1 PE=1 SV=1 - [SQSTM_MOUSE]	242,57	7,01	1	2	2	9	442	48,1	5,21
P60766	Cell division control protein 42 homolog OS=Mus musculus GN=Cdc42 PE=1 SV=2 - [CDC42_MOUSE]	242,51	21,47	1	3	3	10	191	21,2	6,55
P18572	Basigin OS=Mus musculus GN=Bsg PE=1 SV=2 - [BASI_MOUSE]	242,26	12,60	1	4	4	15	389	42,4	5,85
P21981	Protein-glutamine gamma-glutamyltransferase 2 OS=Mus musculus GN=Tgm2 PE=1 SV=4 - [TGM2_MOUSE]	240,64	8,89	1	5	5	8	686	77,0	5,10
Q9DAA6	3'-5' exoribonuclease CSL4 homolog OS=Mus musculus GN=Exosc1 PE=2 SV=1 - [EXOS1_MOUSE]	240,26	18,97	1	3	3	11	195	21,4	8,24
P62700	Protein yippee-like 5 OS=Mus musculus GN=Ypel5 PE=2 SV=1 - [YPEL5_MOUSE]	240,24	29,75	1	4	4	9	121	13,8	7,31
Q9Z321	DNA topoisomerase 3-beta-1 OS=Mus musculus GN=Top3b PE=2 SV=1 - [TOP3B_MOUSE]	238,46	7,54	1	5	5	14	862	96,9	7,93
Q9DBR1	5'-3' exoribonuclease 2 OS=Mus musculus GN=Xrn2 PE=1 SV=1 - [XRN2_MOUSE]	236,91	5,57	1	4	4	9	951	108,6	7,59
P04919	Band 3 anion transport protein OS=Mus musculus GN=Slc4a1 PE=1 SV=1 - [B3AT_MOUSE]	236,12	5,49	1	4	4	16	929	103,1	5,45
Q9DCN2	NADH-cytochrome b5 reductase 3 OS=Mus musculus GN=Cyb5r3 PE=1 SV=3 - [NB5R3_MOUSE]	235,71	17,28	1	4	4	8	301	34,1	8,38
P53994	Ras-related protein Rab-2A OS=Mus musculus GN=Rab2a PE=1 SV=1 - [RAB2A_MOUSE]	234,68	18,87	1	3	3	5	212	23,5	6,54
Q8CHI8	E1A-binding protein p400 OS=Mus musculus GN=Ep400 PE=1 SV=3 - [EP400_MOUSE]	234,63	0,98	1	2	2	9	3072	337,0	9,14
Q9QY76	Vesicle-associated membrane protein-associated protein B OS=Mus musculus GN=Vapb PE=2 SV=3 - [VAPB_MOUSE]	234,54	16,46	1	2	3	7	243	26,9	7,78
P21279	Guanine nucleotide-binding protein G(q) subunit alpha OS=Mus musculus GN=Gnaq PE=1 SV=4 - [GNAQ_MOUSE]	233,10	19,78	1	5	5	8	359	42,1	5,68
Q9DBG5	Perilipin-3 OS=Mus musculus GN=Plin3 PE=1 SV=1 - [PLIN3_MOUSE]	232,51	9,38	1	3	3	6	437	47,2	5,62
P59999	Actin-related protein 2/3 complex subunit 4 OS=Mus musculus GN=Arpc4 PE=1 SV=3 - [ARPC4_MOUSE]	231,84	16,07	1	3	3	16	168	19,7	8,43
Q8CES0	N-alpha-acetyltransferase 30 OS=Mus musculus GN=Naa30 PE=2 SV=2 - [NAA30_MOUSE]	231,47	9,07	1	2	2	4	364	39,4	5,64
Q810D6	Glutamate-rich WD repeat-containing protein 1 OS=Mus musculus GN=Grwd1 PE=2 SV=2 - [GRWD1_MOUSE]	231,05	15,02	1	6	6	9	446	49,2	4,81
Q6NZJ6	Eukaryotic translation initiation factor 4 gamma 1 OS=Mus musculus GN=Eif4g1 PE=1 SV=1 - [IF4G1_MOUSE]	231,02	5,38	1	5	5	18	1600	176,0	5,40
Q9CQS8	Protein transport protein Sec61 subunit beta OS=Mus musculus GN=Sec61b PE=1 SV=3 - [SC61B_MOUSE]	230,37	26,04	1	2	2	11	96	10,0	11,56
Q9DC48	Pre-mRNA-processing factor 17 OS=Mus musculus GN=Cdc40 PE=1 SV=1 - [PRP17_MOUSE]	229,48	15,54	1	6	6	15	579	65,4	7,06
Q3TSG4	Probable alpha-ketoglutarate-dependent dioxygenase ABH5 OS=Mus musculus GN=Alkbh5 PE=1 SV=2 - [ALKB5_MOUSE]	229,44	14,18	1	3	3	5	395	44,4	9,09
Q9D898	Actin-related protein 2/3 complex subunit 5-like protein OS=Mus musculus GN=Arpc5l PE=1 SV=1 - [ARP5L_MOUSE]	229,26	24,84	1	2	3	6	153	17,0	6,80
Q9D0B0	Serine/arginine-rich splicing factor 9 OS=Mus musculus GN=Srsf9 PE=1 SV=1 - [SRSF9_MOUSE]	229,00	40,54	1	7	8	18	222	25,6	8,65
Q920Q6	RNA-binding protein Musashi homolog 2 OS=Mus musculus GN=Msi2 PE=1 SV=1 - [MSI2H_MOUSE]	228,99	8,67	1	2	2	6	346	36,9	8,47
P21107	Tropomyosin alpha-3 chain OS=Mus musculus GN=Tpm3 PE=1 SV=2 - [TPM3_MOUSE]	228,80	13,03	1	3	3	7	284	32,8	4,72
O35344	Importin subunit alpha-3 OS=Mus musculus GN=Kpna3 PE=1 SV=1 - [IMA3_MOUSE]	227,73	6,33	1	3	3	6	521	57,7	4,94
Q60930	Voltage-dependent anion-selective channel protein 2 OS=Mus musculus GN=Vdac2 PE=1 SV=2 - [VDAC2_MOUSE]	226,61	21,36	1	5	5	14	295	31,7	7,49
Q9WTU0	Lysine-specific demethylase PHF2 OS=Mus musculus GN=Phf2 PE=1 SV=2 - [PHF2_MOUSE]	225,95	3,47	1	2	3	5	1096	120,7	9,17
O70172	Phosphatidylinositol-5-phosphate 4-kinase type-2 alpha OS=Mus musculus GN=Pip4k2a PE=1 SV=1 - [PI42A_MOUSE]	225,59	14,81	1	5	5	9	405	46,1	6,99
Q9D0R8	Protein LSM12 homolog OS=Mus musculus GN=Lsm12 PE=1 SV=1 - [LSM12_MOUSE]	224,75	18,97	1	2	2	5	195	21,7	7,74
Q8BP48	Methionine aminopeptidase 1 OS=Mus musculus GN=Metap1 PE=2 SV=1 - [AMPM1_MOUSE]	223,99	13,47	1	4	4	8	386	43,2	7,17
Q8BNV1	tRNA (uracil-5-)-methyltransferase homolog A OS=Mus musculus GN=Trmt2a PE=2 SV=1 - [TRM2A_MOUSE]	223,21	6,97	1	4	4	8	574	63,3	7,25
Q99NH0	Ankyrin repeat domain-containing protein 17 OS=Mus musculus GN=Ankrd17 PE=1 SV=2 - [ANR17_MOUSE]	222,74	2,07	1	4	4	14	2603	274,0	6,52
P41216	Long-chain-fatty-acid--CoA ligase 1 OS=Mus musculus GN=Acsl1 PE=1 SV=2 - [ACSL1_MOUSE]	222,65	3,00	1	2	2	9	699	77,9	7,15
Q811L6	Microtubule-associated serine/threonine-protein kinase 4 OS=Mus musculus GN=Mast4 PE=1 SV=3 - [MAST4_MOUSE]	222,56	3,86	1	6	7	20	2618	283,8	8,62
Q9D4J7	PHD finger protein 6 OS=Mus musculus GN=Phf6 PE=1 SV=1 - [PHF6_MOUSE]	221,41	17,86	1	5	5	21	364	41,1	8,73
Q6A065	Centrosomal protein of 170 kDa OS=Mus musculus GN=Cep170 PE=1 SV=2 - [CE170_MOUSE]	221,20	2,64	1	3	3	9	1588	174,9	7,17
Q9ER00	Syntaxin-12 OS=Mus musculus GN=Stx12 PE=1 SV=1 - [STX12_MOUSE]	220,68	14,96	1	3	3	6	274	31,2	5,44
Q9DB85	Ribosomal RNA-processing protein 8 OS=Mus musculus GN=Rrp8 PE=1 SV=1 - [RRP8_MOUSE]	220,63	8,97	1	2	2	4	457	51,0	9,76
P62331	ADP-ribosylation factor 6 OS=Mus musculus GN=Arf6 PE=1 SV=2 - [ARF6_MOUSE]	220,41	21,14	1	3	3	7	175	20,1	8,95
P62743	AP-2 complex subunit sigma OS=Mus musculus GN=Ap2s1 PE=1 SV=1 - [AP2S1_MOUSE]	220,29	30,28	1	4	4	10	142	17,0	6,18
Q8BWY3	Eukaryotic peptide chain release factor subunit 1 OS=Mus musculus GN=Etf1 PE=1 SV=4 - [ERF1_MOUSE]	220,18	7,32	1	2	3	9	437	49,0	5,71
Q8R149	BUD13 homolog OS=Mus musculus GN=Bud13 PE=1 SV=1 - [BUD13_MOUSE]	219,13	7,22	1	3	3	5	637	72,1	9,95
Q07113	Cation-independent mannose-6-phosphate receptor OS=Mus musculus GN=Igf2r PE=1 SV=1 - [MPRI_MOUSE]	218,56	1,81	1	4	4	11	2483	273,6	5,71
Q9CRD2	Tetratricopeptide repeat protein 35 OS=Mus musculus GN=Ttc35 PE=2 SV=1 - [TTC35_MOUSE]	217,81	7,41	1	2	2	5	297	34,9	6,81
B2RX14	Terminal uridylyltransferase 4 OS=Mus musculus GN=Zcchc11 PE=1 SV=2 - [TUT4_MOUSE]	217,05	4,50	1	6	6	13	1644	184,5	8,19
Q8BKX6	Serine/threonine-protein kinase SMG1 OS=Mus musculus GN=Smg1 PE=1 SV=3 - [SMG1_MOUSE]	216,73	1,45	1	4	4	7	3658	409,5	6,40
Q8K4M5	COMM domain-containing protein 1 OS=Mus musculus GN=Commd1 PE=2 SV=2 - [COMD1_MOUSE]	216,51	12,77	1	2	2	4	188	21,0	7,59
Q00547	Hyaluronan mediated motility receptor OS=Mus musculus GN=Hmmr PE=1 SV=4 - [HMMR_MOUSE]	216,50	12,47	1	5	5	8	794	91,7	5,52
Q9CRB9	Coiled-coil-helix-coiled-coil-helix domain-containing protein 3, mitochondrial OS=Mus musculus GN=Chchd3 PE=1 SV=1 - [CHCH3_MOUSE]	216,20	11,45	1	2	2	4	227	26,3	8,37
Q9D0M5	Dynein light chain 2, cytoplasmic OS=Mus musculus GN=Dynll2 PE=1 SV=1 - [DYL2_MOUSE]	214,90	26,97	1	3	3	8	89	10,3	7,37
P52431	DNA polymerase delta catalytic subunit OS=Mus musculus GN=Pold1 PE=1 SV=2 - [DPOD1_MOUSE]	214,75	5,88	1	5	6	9	1105	123,7	7,61
P01592	Immunoglobulin J chain OS=Mus musculus GN=Igj PE=2 SV=4 - [IGJ_MOUSE]	212,45	14,47	1	2	2	5	159	18,0	4,89
Q3TCH7	Cullin-4A OS=Mus musculus GN=Cul4a PE=1 SV=1 - [CUL4A_MOUSE]	212,42	6,85	1	5	5	9	759	87,7	8,35
Q9QXL1	Kinesin-like protein KIF21B OS=Mus musculus GN=Kif21b PE=1 SV=2 - [KI21B_MOUSE]	212,02	3,36	1	3	3	5	1668	186,1	7,08
P61290	Proteasome activator complex subunit 3 OS=Mus musculus GN=Psme3 PE=1 SV=1 - [PSME3_MOUSE]	211,45	14,57	1	3	3	12	254	29,5	5,95
Q7TQK4	Exosome complex exonuclease RRP40 OS=Mus musculus GN=Exosc3 PE=2 SV=3 - [EXOS3_MOUSE]	210,71	20,80	1	4	4	7	274	29,5	8,06
Q9CXE2	B-cell CLL/lymphoma 7 protein family member A OS=Mus musculus GN=Bcl7a PE=2 SV=1 - [BCL7A_MOUSE]	210,23	21,90	1	3	3	11	210	22,8	5,06
Q7TND5	Ribosome production factor 1 OS=Mus musculus GN=Rpf1 PE=2 SV=2 - [RPF1_MOUSE]	209,30	15,47	1	4	4	7	349	40,0	9,99
Q8CIM8	Integrator complex subunit 4 OS=Mus musculus GN=Ints4 PE=2 SV=1 - [INT4_MOUSE]	208,54	3,63	1	3	3	7	964	108,1	6,42
Q5DTM8	E3 ubiquitin-protein ligase BRE1A OS=Mus musculus GN=Rnf20 PE=1 SV=2 - [BRE1A_MOUSE]	208,25	2,88	1	2	2	4	973	113,5	5,96
Q9QYB5	Gamma-adducin OS=Mus musculus GN=Add3 PE=1 SV=2 - [ADDG_MOUSE]	208,13	7,22	1	4	4	6	706	78,7	5,95
Q91YU8	Suppressor of SWI4 1 homolog OS=Mus musculus GN=Ppan PE=2 SV=2 - [SSF1_MOUSE]	207,91	7,02	1	2	2	5	470	52,7	10,32
O88485	Cytoplasmic dynein 1 intermediate chain 1 OS=Mus musculus GN=Dync1i1 PE=1 SV=2 - [DC1I1_MOUSE]	207,70	10,51	1	4	4	8	628	70,7	5,12
Q60611	DNA-binding protein SATB1 OS=Mus musculus GN=Satb1 PE=1 SV=2 - [SATB1_MOUSE]	207,53	7,20	1	3	3	11	764	85,8	6,54
Q9Z0M6	CD97 antigen OS=Mus musculus GN=Cd97 PE=1 SV=2 - [CD97_MOUSE]	206,90	5,99	1	4	4	7	818	90,4	7,39
P04117	Fatty acid-binding protein, adipocyte OS=Mus musculus GN=Fabp4 PE=1 SV=3 - [FABP4_MOUSE]	206,44	27,27	1	2	2	5	132	14,6	8,40
Q9D8X5	CCR4-NOT transcription complex subunit 8 OS=Mus musculus GN=Cnot8 PE=2 SV=1 - [CNOT8_MOUSE]	205,76	12,33	1	3	3	6	292	33,6	4,81
Q8VE18	Protein SMG8 OS=Mus musculus GN=Smg8 PE=2 SV=1 - [SMG8_MOUSE]	205,55	4,54	1	3	3	5	991	109,6	7,77
O54957	Linker for activation of T-cells family member 1 OS=Mus musculus GN=Lat PE=1 SV=1 - [LAT_MOUSE]	203,23	23,14	1	3	3	7	242	26,0	3,99
O88543	COP9 signalosome complex subunit 3 OS=Mus musculus GN=Cops3 PE=1 SV=3 - [CSN3_MOUSE]	202,76	12,77	1	3	3	8	423	47,8	6,65
Q3UFM5	Nucleolar MIF4G domain-containing protein 1 OS=Mus musculus GN=Nom1 PE=2 SV=2 - [NOM1_MOUSE]	202,71	4,92	1	2	2	4	854	95,9	8,87
P62313	U6 snRNA-associated Sm-like protein LSm6 OS=Mus musculus GN=Lsm6 PE=2 SV=1 - [LSM6_MOUSE]	202,67	48,75	1	7	7	11	80	9,1	9,58
P62046	Leucine-rich repeat and calponin homology domain-containing protein 1 OS=Mus musculus GN=Lrch1 PE=1 SV=2 - [LRCH1_MOUSE]	202,22	10,72	1	6	6	8	709	79,0	6,13
P09103	Protein disulfide-isomerase OS=Mus musculus GN=P4hb PE=1 SV=2 - [PDIA1_MOUSE]	201,98	8,64	1	4	4	11	509	57,0	4,88
P29351	Tyrosine-protein phosphatase non-receptor type 6 OS=Mus musculus GN=Ptpn6 PE=1 SV=2 - [PTN6_MOUSE]	201,52	10,59	1	5	5	7	595	67,5	7,81
Q9CZ04	COP9 signalosome complex subunit 7a OS=Mus musculus GN=Cops7a PE=1 SV=2 - [CSN7A_MOUSE]	200,89	9,45	1	2	2	4	275	30,2	7,87
P46638	Ras-related protein Rab-11B OS=Mus musculus GN=Rab11b PE=1 SV=3 - [RB11B_MOUSE]	199,26	18,35	1	4	4	12	218	24,5	5,94
Q8BJ90	Zinc finger protein 771 OS=Mus musculus GN=Znf771 PE=2 SV=1 - [ZN771_MOUSE]	198,35	7,89	1	2	2	9	317	35,7	8,82
Q60809	CCR4-NOT transcription complex subunit 7 OS=Mus musculus GN=Cnot7 PE=1 SV=1 - [CNOT7_MOUSE]	198,12	14,39	1	3	3	7	285	32,7	4,84
P01027	Complement C3 OS=Mus musculus GN=C3 PE=1 SV=3 - [CO3_MOUSE]	197,93	3,19	1	4	4	6	1663	186,4	6,73
Q8CGZ0	Calcium homeostasis endoplasmic reticulum protein OS=Mus musculus GN=Cherp PE=1 SV=1 - [CHERP_MOUSE]	197,79	8,97	1	8	8	12	936	106,1	9,14
B2RWS6	Histone acetyltransferase p300 OS=Mus musculus GN=Ep300 PE=1 SV=1 - [EP300_MOUSE]	197,06	2,07	1	2	3	15	2415	263,6	8,51
Q9D945	Protein LLP homolog OS=Mus musculus GN=Llph PE=2 SV=1 - [LLPH_MOUSE]	196,56	29,23	1	3	3	9	130	15,4	10,36
P63082	V-type proton ATPase 16 kDa proteolipid subunit OS=Mus musculus GN=Atp6v0c PE=2 SV=1 - [VATL_MOUSE]	196,20	31,61	1	2	2	9	155	15,8	9,13
P16330	2',3'-cyclic-nucleotide 3'-phosphodiesterase OS=Mus musculus GN=Cnp PE=1 SV=3 - [CN37_MOUSE]	196,16	12,14	1	4	4	9	420	47,1	8,97
Q9CQX2	Cytochrome b5 type B OS=Mus musculus GN=Cyb5b PE=1 SV=1 - [CYB5B_MOUSE]	195,98	50,68	1	4	4	9	146	16,3	4,89
Q61699	Heat shock protein 105 kDa OS=Mus musculus GN=Hsph1 PE=1 SV=2 - [HS105_MOUSE]	195,27	6,64	1	3	4	6	858	96,3	5,53

Q91VL8	Telomeric repeat-binding factor 2-interacting protein 1 OS=Mus musculus GN=Terf2ip PE=1 SV=1 - [TE2IP_MOUSE]	195,01	21,63	1	5	5	6	393	43,3	4,81
Q8BKS9	Pumilio domain-containing protein KIAA0020 OS=Mus musculus GN=Kiaa0020 PE=2 SV=2 - [K0020_MOUSE]	194,84	12,98	1	7	7	12	647	72,8	9,66
Q8BVE3	V-type proton ATPase subunit H OS=Mus musculus GN=Atp6v1h PE=1 SV=1 - [VATH_MOUSE]	194,43	16,15	1	5	5	8	483	55,8	6,61
O08585	Clathrin light chain A OS=Mus musculus GN=Clta PE=1 SV=2 - [CLCA_MOUSE]	194,27	8,51	1	2	2	8	235	25,6	4,58
Q8BH57	WD repeat-containing protein 48 OS=Mus musculus GN=Wdr48 PE=1 SV=1 - [WDR48_MOUSE]	193,28	9,02	1	4	4	8	676	76,0	7,17
Q5SWD9	Pre-rRNA-processing protein TSR1 homolog OS=Mus musculus GN=Tsr1 PE=2 SV=1 - [TSR1_MOUSE]	193,12	7,35	1	5	5	8	803	92,0	6,99
Q99MN9	Propionyl-CoA carboxylase beta chain, mitochondrial OS=Mus musculus GN=Pccb PE=1 SV=2 - [PCCB_MOUSE]	193,03	8,50	1	3	3	6	541	58,4	7,66
Q9JJK2	LanC-like protein 2 OS=Mus musculus GN=Lancl2 PE=1 SV=1 - [LANC2_MOUSE]	192,86	5,56	1	2	2	4	450	50,7	7,28
Q9QZ88	Vacuolar protein sorting-associated protein 29 OS=Mus musculus GN=Vps29 PE=1 SV=1 - [VPS29_MOUSE]	191,79	17,03	1	3	3	6	182	20,5	6,79
Q921N6	Probable ATP-dependent RNA helicase DDX27 OS=Mus musculus GN=Ddx27 PE=1 SV=3 - [DDX27_MOUSE]	191,44	9,47	1	6	6	13	760	85,9	9,25
Q9Z1T1	AP-3 complex subunit beta-1 OS=Mus musculus GN=Ap3b1 PE=1 SV=2 - [AP3B1_MOUSE]	190,80	6,33	1	6	6	11	1105	122,7	5,66
Q6ZQ03	Formin-binding protein 4 OS=Mus musculus GN=Fnbp4 PE=1 SV=2 - [FNBP4_MOUSE]	190,77	3,59	1	4	4	7	1031	111,2	4,73
A2AGH6	Mediator of RNA polymerase II transcription subunit 12 OS=Mus musculus GN=Med12 PE=2 SV=1 - [MED12_MOUSE]	190,76	1,87	1	3	3	7	2190	244,4	7,17
O70422	General transcription factor IIH subunit 4 OS=Mus musculus GN=Gtf2h4 PE=2 SV=1 - [TF2H4_MOUSE]	190,53	15,77	1	4	4	6	463	52,2	9,04
P57746	V-type proton ATPase subunit D OS=Mus musculus GN=Atp6v1d PE=1 SV=1 - [VATD_MOUSE]	190,05	10,53	1	2	2	4	247	28,4	9,45
Q91W18	Tudor domain-containing protein 3 OS=Mus musculus GN=Tdrd3 PE=1 SV=4 - [TDRD3_MOUSE]	189,90	10,77	1	5	5	12	743	82,2	9,11
Q3UFS4	Coiled-coil domain-containing protein 75 OS=Mus musculus GN=Ccdc75 PE=2 SV=2 - [CCD75_MOUSE]	189,37	8,78	1	2	2	6	262	30,6	5,08
P70388	DNA repair protein RAD50 OS=Mus musculus GN=Rad50 PE=1 SV=1 - [RAD50_MOUSE]	189,22	6,63	1	7	7	10	1312	153,4	6,95
Q9DBH5	Vesicular integral-membrane protein VIP36 OS=Mus musculus GN=Lman2 PE=2 SV=2 - [LMAN2_MOUSE]	189,07	16,48	1	4	5	6	358	40,4	6,95
Q9D868	Peptidyl-prolyl cis-trans isomerase H OS=Mus musculus GN=Ppih PE=2 SV=1 - [PPIH_MOUSE]	189,01	29,26	1	4	4	8	188	20,5	8,09
Q4FK66	Pre-mRNA-splicing factor 38A OS=Mus musculus GN=Prpf38a PE=1 SV=1 - [PR38A_MOUSE]	188,90	9,62	1	3	3	6	312	37,4	10,01
Q8BGW0	Protein THEMIS OS=Mus musculus GN=Themis PE=1 SV=1 - [THMS1_MOUSE]	188,80	3,77	1	2	2	4	636	72,7	6,13
P31428	Dipeptidase 1 OS=Mus musculus GN=Dpep1 PE=1 SV=2 - [DPEP1_MOUSE]	188,10	12,44	1	4	4	9	410	45,7	6,37
Q99PM9	Uridine-cytidine kinase 2 OS=Mus musculus GN=Uck2 PE=1 SV=1 - [UCK2_MOUSE]	188,05	6,90	1	2	2	6	261	29,4	6,55
P35279	Ras-related protein Rab-6A OS=Mus musculus GN=Rab6a PE=1 SV=4 - [RAB6A_MOUSE]	187,62	15,87	1	2	3	11	208	23,6	5,54
Q8BKN5	Gamma-tubulin complex component 5 OS=Mus musculus GN=Tubgcp5 PE=2 SV=2 - [GCP5_MOUSE]	187,59	4,20	1	2	2	13	1024	117,9	5,99
Q3U1G5	Interferon-stimulated 20 kDa exonuclease-like 2 OS=Mus musculus GN=Isg20l2 PE=2 SV=2 - [I20L2_MOUSE]	187,13	9,78	1	2	2	4	368	41,0	10,01
Q62425	NADH dehydrogenase [ubiquinone] 1 alpha subcomplex subunit 4 OS=Mus musculus GN=Ndufa4 PE=1 SV=2 - [NDUA4_MOUSE]	187,11	36,59	1	4	4	8	82	9,3	9,52
Q62261	Spectrin beta chain, brain 1 OS=Mus musculus GN=Sptbn1 PE=1 SV=2 - [SPTB2_MOUSE]	187,09	2,33	1	4	4	12	2363	274,1	5,58
Q8VCM7	Fibrinogen gamma chain OS=Mus musculus GN=Fgg PE=2 SV=1 - [FIBG_MOUSE]	186,77	9,86	1	3	3	6	436	49,4	5,86
P17918	Proliferating cell nuclear antigen OS=Mus musculus GN=Pcna PE=1 SV=2 - [PCNA_MOUSE]	186,34	13,41	1	2	4	9	261	28,8	4,77
P61967	AP-1 complex subunit sigma-1A OS=Mus musculus GN=Ap1s1 PE=1 SV=1 - [AP1S1_MOUSE]	185,60	20,25	1	3	3	5	158	18,7	5,73
P01635	Ig kappa chain V-V region K2 (Fragment) OS=Mus musculus PE=1 SV=1 - [KV5A3_MOUSE]	184,46	20,00	1	2	2	4	115	12,6	8,31
P83870	PHD finger-like domain-containing protein 5A OS=Mus musculus GN=Phf5a PE=1 SV=1 - [PHF5A_MOUSE]	184,22	13,64	1	2	2	6	110	12,4	8,41
P09838	DNA nucleotidylexotransferase OS=Mus musculus GN=Dntt PE=1 SV=3 - [TDT_MOUSE]	183,26	5,66	1	2	2	4	530	60,3	7,90
P11440	Cell division protein kinase 1 OS=Mus musculus GN=Cdk1 PE=1 SV=3 - [CDK1_MOUSE]	183,20	11,78	1	2	3	9	297	34,1	8,43
Q61739	Integrin alpha-6 OS=Mus musculus GN=Itga6 PE=1 SV=3 - [ITA6_MOUSE]	182,97	2,84	1	2	2	4	1091	122,1	7,03
P24270	Catalase OS=Mus musculus GN=Cat PE=1 SV=4 - [CATA_MOUSE]	181,65	7,97	1	3	3	8	527	59,8	7,88
P35282	Ras-related protein Rab-21 OS=Mus musculus GN=Rab21 PE=1 SV=4 - [RAB21_MOUSE]	180,84	12,16	1	2	2	5	222	24,1	7,94
Q02053	Ubiquitin-like modifier-activating enzyme 1 OS=Mus musculus GN=Uba1 PE=1 SV=1 - [UBA1_MOUSE]	180,70	6,99	1	6	6	7	1058	117,7	5,66
Q9CQ75	NADH dehydrogenase [ubiquinone] 1 alpha subcomplex subunit 2 OS=Mus musculus GN=Ndufa2 PE=1 SV=3 - [NDUA2_MOUSE]	180,53	28,28	1	2	2	5	99	10,9	9,99
Q91WM3	U3 small nucleolar RNA-interacting protein 2 OS=Mus musculus GN=Rrp9 PE=1 SV=1 - [U3IP2_MOUSE]	180,42	14,53	1	6	6	9	475	52,1	7,88
Q9D787	Peptidyl-prolyl cis-trans isomerase-like 2 OS=Mus musculus GN=Ppil2 PE=2 SV=2 - [PPIL2_MOUSE]	180,31	8,64	1	3	3	5	521	59,0	8,38
Q8K2R5	Zinc finger protein 668 OS=Mus musculus GN=Znf668 PE=2 SV=1 - [ZN668_MOUSE]	180,08	6,62	1	4	4	9	619	68,3	8,87
Q9D8Z1	Activating signal cointegrator 1 complex subunit 1 OS=Mus musculus GN=Ascc1 PE=2 SV=1 - [ASCC1_MOUSE]	179,62	15,73	1	5	5	9	356	41,3	6,54
Q9D483	DNA-directed RNA polymerase III subunit RPC3 OS=Mus musculus GN=Polr3c PE=2 SV=1 - [RPC3_MOUSE]	179,55	5,25	1	2	2	3	533	60,7	7,58
P62311	U6 snRNA-associated Sm-like protein LSm3 OS=Mus musculus GN=Lsm3 PE=2 SV=2 - [LSM3_MOUSE]	179,47	13,73	1	2	2	8	102	11,8	4,70
Q9ESZ8	General transcription factor II-I OS=Mus musculus GN=Gtf2i PE=1 SV=3 - [GTF2I_MOUSE]	179,16	3,71	1	3	3	7	998	112,2	6,55
O55022	Membrane-associated progesterone receptor component 1 OS=Mus musculus GN=Pgrmc1 PE=1 SV=4 - [PGRC1_MOUSE]	178,43	17,95	1	3	3	8	195	21,7	4,70
Q9JKK1	Syntaxin-6 OS=Mus musculus GN=Stx6 PE=2 SV=1 - [STX6_MOUSE]	178,37	16,47	1	3	3	5	255	29,0	4,92
Q91Z49	UAP56-interacting factor OS=Mus musculus GN=Fyttd1 PE=1 SV=1 - [UIF_MOUSE]	177,93	14,83	1	4	4	8	317	35,9	11,84
Q91YL3	Uridine-cytidine kinase-like 1 OS=Mus musculus GN=Uckl1 PE=1 SV=1 - [UCKL1_MOUSE]	177,81	16,42	1	6	6	7	548	60,8	7,15
P51807	Dynein light chain Tctex-type 1 OS=Mus musculus GN=Dynlt1 PE=1 SV=1 - [DYLT1_MOUSE]	177,45	30,09	1	2	2	3	113	12,5	5,08
P99029	Peroxiredoxin-5, mitochondrial OS=Mus musculus GN=Prdx5 PE=1 SV=2 - [PRDX5_MOUSE]	177,19	23,81	1	4	4	9	210	21,9	8,85
B2RR83	Probable ATP-dependent RNA helicase YTHDC2 OS=Mus musculus GN=Ythdc2 PE=2 SV=1 - [YTDC2_MOUSE]	177,01	3,18	1	3	3	5	1445	161,0	8,51
Q6KAR6	Exocyst complex component 3 OS=Mus musculus GN=Exoc3 PE=2 SV=2 - [EXOC3_MOUSE]	176,30	5,03	1	3	3	5	755	86,4	6,20
Q9JIY5	Serine protease HTRA2, mitochondrial OS=Mus musculus GN=Htra2 PE=1 SV=2 - [HTRA2_MOUSE]	176,04	5,46	1	2	2	4	458	49,3	9,60
Q9CQR6	Serine/threonine-protein phosphatase 6 catalytic subunit OS=Mus musculus GN=Ppp6c PE=2 SV=1 - [PPP6_MOUSE]	175,85	7,21	1	2	2	7	305	35,1	5,69
Q9CQN6	Transmembrane protein 14C OS=Mus musculus GN=Tmem14c PE=1 SV=1 - [TM14C_MOUSE]	175,11	24,56	1	2	2	3	114	11,6	9,70
Q9DB90	Protein SMG9 OS=Mus musculus GN=Smg9 PE=2 SV=1 - [SMG9_MOUSE]	174,77	14,42	1	4	4	6	520	57,6	7,01
A2BH40	AT-rich interactive domain-containing protein 1A OS=Mus musculus GN=Arid1a PE=1 SV=1 - [ARI1A_MOUSE]	174,71	2,63	1	4	4	11	2283	241,9	6,68
P28352	DNA-(apurinic or apyrimidinic site) lyase OS=Mus musculus GN=Apex1 PE=1 SV=2 - [APEX1_MOUSE]	174,60	13,56	1	3	3	5	317	35,5	7,91
O54946	DnaJ homolog subfamily B member 6 OS=Mus musculus GN=Dnajb6 PE=1 SV=4 - [DNJB6_MOUSE]	174,22	7,95	1	3	3	8	365	39,8	9,36
Q8C9V1	Carabin OS=Mus musculus GN=Tbc1d10c PE=2 SV=1 - [TB10C_MOUSE]	174,05	13,51	1	5	5	7	444	49,9	8,90
Q6P1F6	Serine/threonine-protein phosphatase 2A 55 kDa regulatory subunit B alpha isoform OS=Mus musculus GN=Ppp2r2a PE=1 SV=1 - [2ABA_M	173,73	10,07	1	4	4	8	447	51,7	6,20
Q91YH5	Atlastin-3 OS=Mus musculus GN=Atl3 PE=2 SV=1 - [ATLA3_MOUSE]	173,33	6,84	1	3	3	5	541	60,5	6,10
Q60596	DNA repair protein XRCC1 OS=Mus musculus GN=Xrcc1 PE=1 SV=2 - [XRCC1_MOUSE]	173,13	7,45	1	4	4	9	631	68,9	6,33
Q922R8	Protein disulfide-isomerase A6 OS=Mus musculus GN=Pdia6 PE=1 SV=3 - [PDIA6_MOUSE]	173,09	6,14	1	2	2	3	440	48,1	5,14
Q9Z1E4	Glycogen [starch] synthase, muscle OS=Mus musculus GN=Gys1 PE=1 SV=2 - [GYS1_MOUSE]	172,99	4,34	1	3	3	5	738	83,9	6,11
O55102	Biogenesis of lysosome-related organelles complex 1 subunit 1 OS=Mus musculus GN=Bloc1s1 PE=1 SV=2 - [BL1S1_MOUSE]	172,90	17,60	1	2	2	5	125	14,3	8,15
O88545	COP9 signalosome complex subunit 6 OS=Mus musculus GN=Cops6 PE=1 SV=1 - [CSN6_MOUSE]	172,86	21,30	1	5	5	12	324	35,9	5,73
Q8BVF2	Phosducin-like protein 3 OS=Mus musculus GN=Pdcl3 PE=1 SV=1 - [PDCL3_MOUSE]	171,54	27,92	1	7	7	12	240	27,6	4,72
Q8BX09	Retinoblastoma-binding protein 5 OS=Mus musculus GN=Rbbp5 PE=1 SV=2 - [RBBP5_MOUSE]	171,35	5,95	1	3	3	5	538	59,1	5,10
Q3V300	Kinesin-like protein KIF22 OS=Mus musculus GN=Kif22 PE=2 SV=2 - [KIF22_MOUSE]	170,96	3,64	1	2	2	4	660	73,1	9,25
Q923B1	Lariat debranching enzyme OS=Mus musculus GN=Dbr1 PE=1 SV=2 - [DBR1_MOUSE]	170,65	4,36	1	2	2	6	550	62,3	5,22
P38647	Stress-70 protein, mitochondrial OS=Mus musculus GN=Hspa9 PE=1 SV=3 - [GRP75_MOUSE]	170,19	7,95	1	4	4	5	679	73,4	6,07
**Q6A0A2**	La-related protein 4B OS=Mus musculus GN=Larp4b PE=1 SV=2 - [LAR4B_MOUSE]	170,05	3,64	1	2	2	3	741	81,6	7,49
P63085	Mitogen-activated protein kinase 1 OS=Mus musculus GN=Mapk1 PE=1 SV=3 - [MK01_MOUSE]	169,23	17,60	1	5	5	9	358	41,2	6,98
Q8VHN8	Protein syndesmos OS=Mus musculus GN=Nudt16l1 PE=1 SV=2 - [SDOS_MOUSE]	169,03	18,01	1	3	3	4	211	23,4	9,26
Q62187	Transcription termination factor 1 OS=Mus musculus GN=Ttf1 PE=1 SV=2 - [TTF1_MOUSE]	168,26	5,70	1	2	3	10	859	97,7	9,38
Q4QQM4	Tumor protein p53-inducible protein 11 OS=Mus musculus GN=Trp53i11 PE=1 SV=1 - [P5I11_MOUSE]	167,89	13,76	1	3	3	6	189	20,9	9,41
P63323	40S ribosomal protein S12 OS=Mus musculus GN=Rps12 PE=1 SV=2 - [RS12_MOUSE]	167,75	17,42	1	3	3	9	132	14,5	7,24
Q6ZQ88	Lysine-specific histone demethylase 1A OS=Mus musculus GN=Kdm1a PE=1 SV=2 - [KDM1A_MOUSE]	166,83	6,80	1	4	4	5	853	92,8	6,52
Q8CB77	Transcription elongation factor B polypeptide 3 OS=Mus musculus GN=Tceb3 PE=1 SV=3 - [ELOA1_MOUSE]	166,79	4,27	1	2	2	4	773	87,1	9,61
P06801	NADP-dependent malic enzyme OS=Mus musculus GN=Me1 PE=1 SV=2 - [MAOX_MOUSE]	166,55	12,06	1	4	4	5	572	63,9	7,44
P97390	Vacuolar protein sorting-associated protein 45 OS=Mus musculus GN=Vps45 PE=1 SV=1 - [VPS45_MOUSE]	166,07	12,98	1	7	7	10	570	65,0	8,25
O35601	FYN-binding protein OS=Mus musculus GN=Fyb PE=1 SV=2 - [FYB_MOUSE]	165,09	4,15	1	2	2	5	819	90,0	6,98
P19157	Glutathione S-transferase P 1 OS=Mus musculus GN=Gstp1 PE=1 SV=2 - [GSTP1_MOUSE]	165,03	23,33	1	3	3	5	210	23,6	7,87
Q8BHS3	Pre-mRNA-splicing factor RBM22 OS=Mus musculus GN=Rbm22 PE=2 SV=1 - [RBM22_MOUSE]	164,24	12,14	1	4	4	10	420	46,9	8,54
Q91VR2	ATP synthase subunit gamma, mitochondrial OS=Mus musculus GN=Atp5c1 PE=1 SV=1 - [ATPG_MOUSE]	164,01	7,72	1	2	2	4	298	32,9	9,01
P04231	H-2 class II histocompatibility antigen, E-S beta chain (Fragment) OS=Mus musculus GN=H2-Eb1 PE=1 SV=1 - [HB23_MOUSE]	163,11	12,50	3	3	3	6	232	26,6	5,62
Q61990	Poly(rC)-binding protein 2 OS=Mus musculus GN=Pcbp2 PE=1 SV=1 - [PCBP2_MOUSE]	162,34	12,15	1	2	3	9	362	38,2	6,79
Q9D7H3	RNA 3'-terminal phosphate cyclase OS=Mus musculus GN=Rtcd1 PE=2 SV=2 - [RTC1_MOUSE]	161,34	20,49	1	5	5	6	366	39,2	7,90
P97473	RISC-loading complex subunit TARBP2 OS=Mus musculus GN=Tarbp2 PE=1 SV=2 - [TRBP2_MOUSE]	160,55	9,59	1	3	3	8	365	38,8	6,01
P62827	GTP-binding nuclear protein Ran OS=Mus musculus GN=Ran PE=1 SV=3 - [RAN_MOUSE]	160,54	30,09	1	4	5	11	216	24,4	7,49
Q922H4	Mannose-1-phosphate guanyltransferase alpha OS=Mus musculus GN=Gmppa PE=2 SV=1 - [GMPPA_MOUSE]	160,47	6,90	1	2	2	6	420	46,2	7,62
Q8BFY9	Transportin-1 OS=Mus musculus GN=Tnpo1 PE=1 SV=2 - [TNPO1_MOUSE]	160,35	6,35	1	5	5	13	898	102,3	4,98
Q3UQU0	Bromodomain-containing protein 9 OS=Mus musculus GN=Brd9 PE=2 SV=1 - [BRD9_MOUSE]	159,87	8,05	1	3	3	7	596	66,8	6,27
Q9ERG2	Striatin-3 OS=Mus musculus GN=Strn3 PE=1 SV=1 - [STRN3_MOUSE]	159,84	4,27	1	3	3	5	796	87,1	5,29
Q9D1N9	39S ribosomal protein L21, mitochondrial OS=Mus musculus GN=Mrpl21 PE=2 SV=1 - [RM21_MOUSE]	159,48	9,09	1	2	2	7	209	23,4	9,99
Q922S8	Kinesin-like protein KIF2C OS=Mus musculus GN=Kif2c PE=1 SV=1 - [KIF2C_MOUSE]	158,57	3,05	1	2	2	6	721	81,0	7,97
Q9D0M3	Cytochrome c1, heme protein, mitochondrial OS=Mus musculus GN=Cyc1 PE=1 SV=1 - [CY1_MOUSE]	158,01	18,15	1	4	4	9	325	35,3	9,16
Q9DBX2	Phosducin-like protein OS=Mus musculus GN=Pdcl PE=1 SV=1 - [PHLP_MOUSE]	157,72	22,92	1	5	5	7	301	34,4	4,87
O88874	Cyclin-K OS=Mus musculus GN=Ccnk PE=1 SV=3 - [CCNK_MOUSE]	156,94	12,27	1	4	4	13	554	61,3	8,41
Q497V5	S1 RNA-binding domain-containing protein 1 OS=Mus musculus GN=Srbd1 PE=1 SV=2 - [SRBD1_MOUSE]	156,78	4,14	1	4	5	12	1015	114,0	8,78
Q9CQS2	H/ACA ribonucleoprotein complex subunit 3 OS=Mus musculus GN=Nop10 PE=2 SV=1 - [NOP10_MOUSE]	156,32	62,50	1	4	4	9	64	7,7	9,99
O70311	Glycylpeptide N-tetradecanoyltransferase 2 OS=Mus musculus GN=Nmt2 PE=1 SV=1 - [NMT2_MOUSE]	155,82	8,70	1	2	3	5	529	60,4	7,80
Q9DBY8	Nuclear valosin-containing protein-like OS=Mus musculus GN=Nvl PE=2 SV=1 - [NVL_MOUSE]	155,32	5,38	1	2	3	5	855	94,4	6,35
Q6ZPL9	ATP-dependent RNA helicase DDX55 OS=Mus musculus GN=Ddx55 PE=2 SV=2 - [DDX55_MOUSE]	155,03	4,83	1	3	3	6	600	68,4	9,38
P56395	Cytochrome b5 OS=Mus musculus GN=Cyb5a PE=1 SV=2 - [CYB5_MOUSE]	154,82	41,04	1	4	4	10	134	15,2	5,07
Q6P5E4	UDP-glucose:glycoprotein glucosyltransferase 1 OS=Mus musculus GN=Uggt1 PE=1 SV=4 - [UGGG1_MOUSE]	153,66	3,68	1	4	5	7	1551	176,3	5,62
Q9Z0Y1	Dynactin subunit 3 OS=Mus musculus GN=Dctn3 PE=2 SV=2 - [DCTN3_MOUSE]	153,43	14,52	1	3	3	10	186	21,0	6,06
P22892	AP-1 complex subunit gamma-1 OS=Mus musculus GN=Ap1g1 PE=1 SV=3 - [AP1G1_MOUSE]	153,41	3,28	1	3	3	8	822	91,3	6,80
Q9JLQ2	ARF GTPase-activating protein GIT2 OS=Mus musculus GN=Git2 PE=1 SV=2 - [GIT2_MOUSE]	153,27	8,19	1	3	4	8	708	78,7	7,72
O55128	Histone deacetylase complex subunit SAP18 OS=Mus musculus GN=Sap18 PE=2 SV=1 - [SAP18_MOUSE]	153,23	17,65	1	2	2	5	153	17,6	9,35
P97499	Telomerase protein component 1 OS=Mus musculus GN=Tep1 PE=1 SV=1 - [TEP1_MOUSE]	152,49	3,20	1	7	7	10	2629	291,3	7,24
P20491	High affinity immunoglobulin epsilon receptor subunit gamma OS=Mus musculus GN=Fcer1g PE=1 SV=1 - [FCERG_MOUSE]	152,47	15,12	1	2	2	6	86	9,6	7,97
Q99JP6	Homer protein homolog 3 OS=Mus musculus GN=Homer3 PE=1 SV=2 - [HOME3_MOUSE]	151,90	11,80	1	3	3	6	356	39,7	5,36
Q8K1X1	WD repeat-containing protein 11 OS=Mus musculus GN=Wdr11 PE=2 SV=1 - [WDR11_MOUSE]	151,11	2,78	1	3	3	5	1223	135,9	7,11
Q9R0L7	A-kinase anchor protein 8-like OS=Mus musculus GN=Akap8l PE=1 SV=1 - [AKP8L_MOUSE]	150,46	4,05	1	2	2	3	642	71,4	5,05
Q80UU9	Membrane-associated progesterone receptor component 2 OS=Mus musculus GN=Pgrmc2 PE=1 SV=2 - [PGRC2_MOUSE]	150,25	17,97	1	2	2	2	217	23,3	5,15

Q9QZB7	Actin-related protein 10 OS=Mus musculus GN=Actr10 PE=1 SV=2 - [ARP10_MOUSE]	148,90	16,79	1	4	4	6	417	46,2	7,61
Q99LD4	COP9 signalosome complex subunit 1 OS=Mus musculus GN=Gps1 PE=1 SV=1 - [CSN1_MOUSE]	148,38	11,04	1	4	4	9	471	53,4	6,84
Q8BMA6	Signal recognition particle 68 kDa protein OS=Mus musculus GN=Srp68 PE=2 SV=2 - [SRP68_MOUSE]	148,19	8,48	1	4	4	6	625	70,5	8,57
Q8CH25	SAFB-like transcription modulator OS=Mus musculus GN=Sltm PE=1 SV=1 - [SLTM_MOUSE]	147,39	4,27	1	3	3	4	1031	116,8	7,72
Q8CGC6	RNA-binding protein 28 OS=Mus musculus GN=Rbm28 PE=1 SV=4 - [RBM28_MOUSE]	147,35	5,87	1	5	5	7	750	84,2	9,54
Q9CWS4	Integrator complex subunit 11 OS=Mus musculus GN=Cpsf3l PE=2 SV=1 - [INT11_MOUSE]	147,23	5,67	1	3	3	7	600	67,8	7,94
Q8BXC6	COMM domain-containing protein 2 OS=Mus musculus GN=Commd2 PE=1 SV=1 - [COMD2_MOUSE]	147,18	8,04	1	2	2	7	199	22,8	6,62
Q921E6	Polycomb protein EED OS=Mus musculus GN=Eed PE=1 SV=1 - [EED_MOUSE]	146,35	9,30	1	3	3	9	441	50,2	7,03
Q9D0D4	Probable dimethyladenosine transferase OS=Mus musculus GN=Dimt1l PE=2 SV=1 - [DIMT1_MOUSE]	146,21	18,85	1	5	5	9	313	35,3	9,99
P70296	Phosphatidylethanolamine-binding protein 1 OS=Mus musculus GN=Pebp1 PE=1 SV=3 - [PEBP1_MOUSE]	145,94	20,86	1	2	2	4	187	20,8	5,40
Q9D154	Leukocyte elastase inhibitor A OS=Mus musculus GN=Serpinb1a PE=1 SV=1 - [ILEUA_MOUSE]	145,74	13,19	1	4	4	6	379	42,5	6,21
Q99KC8	von Willebrand factor A domain-containing protein 5A OS=Mus musculus GN=Vwa5a PE=1 SV=2 - [VMA5A_MOUSE]	145,73	6,31	1	4	4	6	793	87,1	6,58
Q8R349	Cell division cycle protein 16 homolog OS=Mus musculus GN=Cdc16 PE=2 SV=1 - [CDC16_MOUSE]	145,70	5,81	1	4	4	7	620	71,4	5,76
Q99K70	Ras-related GTP-binding protein C OS=Mus musculus GN=Rragc PE=2 SV=1 - [RRAGC_MOUSE]	144,67	11,06	1	4	4	12	398	44,1	5,10
Q8C6B9	Active regulator of SIRT1 OS=Mus musculus GN=Rps19bp1 PE=1 SV=1 - [AROS_MOUSE]	144,66	22,38	1	2	2	4	143	16,0	10,83
Q8K2D3	Enhancer of mRNA-decapping protein 3 OS=Mus musculus GN=Edc3 PE=2 SV=1 - [EDC3_MOUSE]	144,57	3,94	1	2	2	3	508	55,9	7,09
Q9CQ62	2,4-dienoyl-CoA reductase, mitochondrial OS=Mus musculus GN=Decr1 PE=1 SV=1 - [DECR_MOUSE]	144,24	15,82	1	3	3	5	335	36,2	8,95
Q8VDG3	Poly(A)-specific ribonuclease PARN OS=Mus musculus GN=Parn PE=1 SV=1 - [PARN_MOUSE]	143,92	3,37	1	2	2	4	624	71,5	5,97
Q8K0E8	Fibrinogen beta chain OS=Mus musculus GN=Fgb PE=2 SV=1 - [FIBB_MOUSE]	143,85	6,03	1	2	2	3	481	54,7	7,08
Q3TLD5	Unconventional prefoldin RPB5 interactor OS=Mus musculus GN=Rmp PE=1 SV=2 - [RMP_MOUSE]	143,33	6,97	1	2	2	4	531	59,0	5,07
Q8CEC6	Peptidylprolyl isomerase domain and WD repeat-containing protein 1 OS=Mus musculus GN=Ppwd1 PE=2 SV=2 - [PPWD1_MOUSE]	143,16	7,74	1	3	3	5	646	73,3	7,15
A6PWY4	WD repeat-containing protein 76 OS=Mus musculus GN=Wdr76 PE=2 SV=1 - [WDR76_MOUSE]	143,03	7,72	1	4	4	8	622	69,0	9,36
Q9CWZ7	Gamma-soluble NSF attachment protein OS=Mus musculus GN=Napg PE=1 SV=1 - [SNAG_MOUSE]	142,73	9,62	1	2	2	3	312	34,7	5,41
B1AVZ0	Uracil phosphoribosyltransferase homolog OS=Mus musculus GN=Uprt PE=2 SV=1 - [UPP_MOUSE]	142,70	13,23	1	3	3	9	310	34,3	6,23
Q8BZQ7	Anaphase-promoting complex subunit 2 OS=Mus musculus GN=Anapc2 PE=1 SV=2 - [ANC2_MOUSE]	142,37	7,53	1	4	5	9	837	95,2	5,31
P61804	Dolichyl-diphosphooligosaccharide--protein glycosyltransferase subunit DAD1 OS=Mus musculus GN=Dad1 PE=2 SV=3 - [DAD1_MOUSE]	142,27	19,47	1	2	2	4	113	12,5	7,08
Q9EQU5	Protein SET OS=Mus musculus GN=Set PE=1 SV=1 - [SET_MOUSE]	142,16	7,96	1	2	2	5	289	33,4	4,32
Q99J09	Methylosome protein 50 OS=Mus musculus GN=Wdr77 PE=1 SV=1 - [MEP50_MOUSE]	141,84	6,43	1	2	2	6	342	36,9	5,27
Q9R0P6	Signal peptidase complex catalytic subunit SEC11A OS=Mus musculus GN=Sec11a PE=2 SV=1 - [SC11A_MOUSE]	141,22	9,50	1	2	2	4	179	20,6	9,33
Q9JIG7	Coiled-coil domain-containing protein 22 OS=Mus musculus GN=Ccdc22 PE=2 SV=1 - [CCD22_MOUSE]	140,98	7,97	1	4	4	6	627	70,8	6,01
Q9CWN7	UPF0760 protein C2orf29 homolog OS=Mus musculus GN=D1Bwg0212e PE=2 SV=1 - [CB029_MOUSE]	140,03	12,87	1	4	4	7	505	54,9	6,55
Q99LG0	Ubiquitin carboxyl-terminal hydrolase 16 OS=Mus musculus GN=Usp16 PE=1 SV=1 - [UBP16_MOUSE]	139,98	2,79	1	2	2	4	825	93,3	6,18
Q9CZ30	Obg-like ATPase 1 OS=Mus musculus GN=Ola1 PE=1 SV=1 - [OLA1_MOUSE]	139,97	6,57	1	2	2	3	396	44,7	7,81
Q9R1T4	Septin-6 OS=Mus musculus GN=Sept6 PE=1 SV=4 - [SEPT6_MOUSE]	139,43	11,75	1	3	5	10	434	49,6	6,43
Q9Z266	SNARE-associated protein Snapin OS=Mus musculus GN=Snapin PE=1 SV=1 - [SNAPN_MOUSE]	138,71	28,68	1	3	3	3	136	14,9	9,31
Q9DCX2	ATP synthase subunit d, mitochondrial OS=Mus musculus GN=Atp5h PE=1 SV=3 - [ATP5H_MOUSE]	137,80	28,57	1	4	4	8	161	18,7	5,69
Q61687	Transcriptional regulator ATRX OS=Mus musculus GN=Atrx PE=1 SV=3 - [ATRX_MOUSE]	137,07	1,33	1	2	2	5	2476	278,4	6,68
O08573	Galectin-9 OS=Mus musculus GN=Lgals9 PE=1 SV=1 - [LEG9_MOUSE]	136,11	6,23	1	2	2	5	353	40,0	9,31
Q9CQ49	Nuclear cap-binding protein subunit 2 OS=Mus musculus GN=Ncbp2 PE=1 SV=1 - [NCBP2_MOUSE]	135,80	10,26	1	2	2	4	156	18,0	7,81
P26350	Prothymosin alpha OS=Mus musculus GN=Ptma PE=1 SV=2 - [PTMA_MOUSE]	135,60	25,23	1	2	2	4	111	12,2	3,79
Q9Z0H4	CUGBP Elav-like family member 2 OS=Mus musculus GN=Celf2 PE=1 SV=1 - [CELF2_MOUSE]	135,51	4,33	1	3	3	7	508	54,2	8,76
Q61599	Rho GDP-dissociation inhibitor 2 OS=Mus musculus GN=Arhgdib PE=1 SV=3 - [GDIR2_MOUSE]	135,25	30,50	1	3	3	6	200	22,8	5,11
P84089	Enhancer of rudimentary homolog OS=Mus musculus GN=Erh PE=1 SV=1 - [ERH_MOUSE]	135,15	40,38	1	5	5	13	104	12,3	5,92
P39749	Flap endonuclease 1 OS=Mus musculus GN=Fen1 PE=1 SV=1 - [FEN1_MOUSE]	133,78	5,82	1	2	2	5	378	42,3	8,34
Q99LH1	Nucleolar GTP-binding protein 2 OS=Mus musculus GN=Gnl2 PE=2 SV=2 - [NOG2_MOUSE]	133,66	7,69	1	4	4	7	728	83,3	9,20
P54116	Erythrocyte band 7 integral membrane protein OS=Mus musculus GN=Stom PE=1 SV=3 - [STOM_MOUSE]	133,55	10,92	1	2	2	3	284	31,4	6,93
Q8R0X7	Sphingosine-1-phosphate lyase 1 OS=Mus musculus GN=Sgpl1 PE=2 SV=1 - [SGPL1_MOUSE]	133,52	8,27	1	3	4	12	568	63,6	9,10
Q9DB42	Zinc finger protein 593 OS=Mus musculus GN=Znf593 PE=2 SV=2 - [ZN593_MOUSE]	133,19	26,12	1	3	3	7	134	15,1	9,55
P27782	Lymphoid enhancer-binding factor 1 OS=Mus musculus GN=Lef1 PE=1 SV=1 - [LEF1_MOUSE]	133,08	5,54	4	2	2	8	397	44,0	7,42
Q8R344	Coiled-coil domain-containing protein 12 OS=Mus musculus GN=Ccdc12 PE=1 SV=2 - [CCD12_MOUSE]	132,99	29,52	1	3	3	6	166	18,9	7,21
Q9DBC3	Cap-specific mRNA (nucleoside-2'-O-)-methyltransferase 1 OS=Mus musculus GN=Ftsjd2 PE=1 SV=1 - [MTR1_MOUSE]	132,96	9,08	1	6	6	9	837	95,6	7,27
O55026	Ectonucleoside triphosphate diphosphohydrolase 2 OS=Mus musculus GN=Entpd2 PE=1 SV=2 - [ENTP2_MOUSE]	132,20	6,06	1	3	3	5	495	54,3	8,37
Q8K4Q0	Regulatory-associated protein of mTOR OS=Mus musculus GN=Rptor PE=1 SV=1 - [RPTOR_MOUSE]	131,80	2,40	1	2	2	3	1335	149,4	6,87
Q9ES56	Trafficking protein particle complex subunit 4 OS=Mus musculus GN=Trappc4 PE=1 SV=1 - [TPPC4_MOUSE]	131,23	19,18	1	4	4	6	219	24,4	6,21
P33174	Chromosome-associated kinesin KIF4 OS=Mus musculus GN=Kif4 PE=2 SV=3 - [KIF4_MOUSE]	131,07	2,27	1	3	3	9	1231	139,4	6,68
Q9Z1Q5	Chloride intracellular channel protein 1 OS=Mus musculus GN=Clic1 PE=1 SV=3 - [CLIC1_MOUSE]	130,99	16,18	1	3	3	5	241	27,0	5,17
O70252	Heme oxygenase 2 OS=Mus musculus GN=Hmox2 PE=2 SV=1 - [HMOX2_MOUSE]	130,09	21,90	1	4	4	6	315	35,7	5,87
P83510	Traf2 and NCK-interacting protein kinase OS=Mus musculus GN=Tnik PE=1 SV=2 - [TNIK_MOUSE]	129,27	2,49	1	2	4	15	1323	150,3	7,27
Q9CRB2	H/ACA ribonucleoprotein complex subunit 2 OS=Mus musculus GN=Nhp2 PE=2 SV=1 - [NHP2_MOUSE]	129,10	12,42	1	2	2	3	153	17,2	8,41
P49817	Caveolin-1 OS=Mus musculus GN=Cav1 PE=1 SV=1 - [CAV1_MOUSE]	128,69	13,48	1	2	2	4	178	20,5	6,02
P63321	Ras-related protein Ral-A OS=Mus musculus GN=Rala PE=1 SV=1 - [RALA_MOUSE]	127,67	15,53	1	3	3	6	206	23,5	7,11
Q9R0X0	Mediator of RNA polymerase II transcription subunit 20 OS=Mus musculus GN=Med20 PE=1 SV=1 - [MED20_MOUSE]	127,48	10,38	1	2	2	4	212	23,2	6,87
Q8BSQ9	Protein polybromo-1 OS=Mus musculus GN=Pbrm1 PE=1 SV=4 - [PB1_MOUSE]	126,67	2,94	1	2	4	14	1634	187,1	6,90
P42128	Forkhead box protein K1 OS=Mus musculus GN=Foxk1 PE=1 SV=2 - [FOXK1_MOUSE]	126,31	8,07	1	4	4	6	719	74,9	9,17
Q99LI9	Polyribonucleotide 5'-hydroxyl-kinase Clp1 OS=Mus musculus GN=Clp1 PE=2 SV=1 - [CLP1_MOUSE]	124,70	5,41	1	3	3	4	425	47,7	6,62
Q5NCR9	Coiled-coil domain-containing protein 55 OS=Mus musculus GN=Ccdc55 PE=1 SV=1 - [CCD55_MOUSE]	123,81	4,80	1	2	2	4	542	63,8	8,76
Q9QWT9	Kinesin-like protein KIFC1 OS=Mus musculus GN=Kifc1 PE=1 SV=2 - [KIFC1_MOUSE]	123,57	3,71	1	2	2	3	674	74,1	8,72
Q3UL36	Arginine and glutamate-rich protein 1 OS=Mus musculus GN=Arglu1 PE=1 SV=2 - [ARGL1_MOUSE]	123,45	5,90	1	2	2	7	271	32,9	10,36
P62257	Ubiquitin-conjugating enzyme E2 H OS=Mus musculus GN=Ube2h PE=2 SV=1 - [UBE2H_MOUSE]	123,33	18,58	1	3	3	8	183	20,6	4,67
Q9R0L6	Pericentriolar material 1 protein OS=Mus musculus GN=Pcm1 PE=1 SV=2 - [PCM1_MOUSE]	123,23	1,28	1	2	2	5	2025	228,7	5,01
P58871	182 kDa tankyrase-1-binding protein OS=Mus musculus GN=Tnks1bp1 PE=1 SV=2 - [TB182_MOUSE]	123,12	5,23	1	5	5	5	1720	181,7	4,88
B2RY04	Dedicator of cytokinesis protein 5 OS=Mus musculus GN=Dock5 PE=1 SV=2 - [DOCK5_MOUSE]	123,06	1,87	1	2	3	5	1868	214,3	7,55
P01837	Ig kappa chain C region OS=Mus musculus PE=1 SV=1 - [IGKC_MOUSE]	122,77	17,92	1	2	2	5	106	11,8	5,41
Q9CR20	Immediate early response 3-interacting protein 1 OS=Mus musculus GN=Ier3ip1 PE=2 SV=1 - [IR3IP_MOUSE]	122,56	34,15	1	2	2	6	82	9,0	8,22
O35130	Probable ribosome biogenesis protein NEP1 OS=Mus musculus GN=Emg1 PE=1 SV=1 - [NEP1_MOUSE]	122,43	10,25	1	2	2	3	244	27,0	8,91
Q99KV1	DnaJ homolog subfamily B member 11 OS=Mus musculus GN=Dnajb11 PE=1 SV=1 - [DJB11_MOUSE]	122,03	7,82	1	3	3	5	358	40,5	6,32
P54227	Stathmin OS=Mus musculus GN=Stmn1 PE=1 SV=2 - [STMN1_MOUSE]	121,52	24,83	1	3	3	6	149	17,3	5,97
Q4FZF3	Probable ATP-dependent RNA helicase DDX49 OS=Mus musculus GN=Ddx49 PE=2 SV=1 - [DDX49_MOUSE]	121,04	5,00	1	2	2	3	480	54,1	8,78
Q8R2U0	Nucleoporin SEH1 OS=Mus musculus GN=Seh1l PE=2 SV=1 - [SEH1_MOUSE]	120,60	8,61	1	2	2	4	360	39,7	8,05
Q61216	Double-strand break repair protein MRE11A OS=Mus musculus GN=Mre11a PE=2 SV=1 - [MRE11_MOUSE]	120,41	7,79	1	5	5	7	706	80,2	6,01
Q91W96	Anaphase-promoting complex subunit 4 OS=Mus musculus GN=Anapc4 PE=2 SV=1 - [APC4_MOUSE]	120,08	5,20	1	3	3	5	807	91,6	5,40
Q9DAP7	Histone chaperone ASF1B OS=Mus musculus GN=Asf1b PE=1 SV=1 - [ASF1B_MOUSE]	119,72	18,32	1	2	3	6	202	22,5	4,44
Q9CQ25	Mitotic-spindle organizing protein 2 OS=Mus musculus GN=Mzt2 PE=1 SV=1 - [MZT2_MOUSE]	119,43	22,01	1	2	2	2	159	16,5	10,18
Q9D1H7	Golgi to ER traffic protein 4 homolog OS=Mus musculus GN=Get4 PE=2 SV=2 - [GET4_MOUSE]	119,23	11,01	1	3	3	6	327	36,5	5,41
Q921K8	Protein FAM115C OS=Mus musculus GN=Fam115c PE=2 SV=1 - [F115C_MOUSE]	118,98	2,94	1	2	3	13	919	101,5	6,52
Q8VE10	N-alpha-acetyltransferase 40, NatD catalytic subunit OS=Mus musculus GN=Naa40 PE=2 SV=1 - [NAA40_MOUSE]	118,80	8,02	1	2	2	3	237	27,2	7,36
Q91ZX7	Prolow-density lipoprotein receptor-related protein 1 OS=Mus musculus GN=Lrp1 PE=1 SV=1 - [LRP1_MOUSE]	118,26	0,51	1	2	2	6	4545	504,4	5,36
Q8BHJ5	F-box-like/WD repeat-containing protein TBL1XR1 OS=Mus musculus GN=Tbl1xr1 PE=2 SV=1 - [TBL1R_MOUSE]	117,65	7,00	1	2	2	4	514	55,6	5,63
Q3UGS4	Protein FAM195B OS=Mus musculus GN=Fam195b PE=1 SV=1 - [F195B_MOUSE]	117,37	22,68	1	2	2	6	97	11,1	9,14
P67871	Casein kinase II subunit beta OS=Mus musculus GN=Csnk2b PE=1 SV=1 - [CSK2B_MOUSE]	117,06	25,58	1	4	5	8	215	24,9	5,55
Q8BTU1	UPF0468 protein C16orf80 homolog OS=Mus musculus GN=Gtl3 PE=2 SV=1 - [CP080_MOUSE]	116,62	11,92	1	2	2	4	193	22,7	9,76
P40694	DNA-binding protein SMUBP-2 OS=Mus musculus GN=Ighmbp2 PE=1 SV=1 - [SMBP2_MOUSE]	116,21	4,83	1	3	3	5	993	109,4	8,40
Q9CPY7	Cytosol aminopeptidase OS=Mus musculus GN=Lap3 PE=1 SV=3 - [AMPL_MOUSE]	115,44	7,32	1	2	3	5	519	56,1	7,72
Q8C5N3	Pre-mRNA-splicing factor CWC22 homolog OS=Mus musculus GN=Cwc22 PE=1 SV=1 - [CWC22_MOUSE]	115,38	4,52	1	3	4	7	908	104,7	8,09
P70452	Syntaxin-4 OS=Mus musculus GN=Stx4 PE=1 SV=1 - [STX4_MOUSE]	115,36	10,07	1	2	2	6	298	34,1	6,14
Q62446	Peptidyl-prolyl cis-trans isomerase FKBP3 OS=Mus musculus GN=Fkbp3 PE=1 SV=2 - [FKBP3_MOUSE]	115,21	11,61	1	2	2	5	224	25,1	9,28
Q8VE80	THO complex subunit 3 OS=Mus musculus GN=Thoc3 PE=2 SV=1 - [THOC3_MOUSE]	114,98	11,97	1	3	3	6	351	38,7	6,09
Q8BSA9	tRNA wybutosine-synthesizing protein 3 homolog OS=Mus musculus GN=Tyw3 PE=1 SV=1 - [TYW3_MOUSE]	114,69	12,84	1	2	2	3	257	28,6	7,78
Q9D1K2	V-type proton ATPase subunit F OS=Mus musculus GN=Atp6v1f PE=1 SV=2 - [VATF_MOUSE]	114,10	9,24	1	2	2	6	119	13,4	5,82
O54692	Centromere/kinetochore protein zw10 homolog OS=Mus musculus GN=Zw10 PE=2 SV=3 - [ZW10_MOUSE]	114,03	5,13	1	3	3	4	779	88,0	5,92
P63028	Translationally-controlled tumor protein OS=Mus musculus GN=Tpt1 PE=1 SV=1 - [TCTP_MOUSE]	113,86	15,70	1	2	2	4	172	19,4	4,86
Q8BKZ9	Pyruvate dehydrogenase protein X component, mitochondrial OS=Mus musculus GN=Pdhx PE=2 SV=1 - [ODPX_MOUSE]	113,75	4,59	1	2	2	4	501	54,0	7,75
Q9DCJ1	Target of rapamycin complex subunit LST8 OS=Mus musculus GN=Mlst8 PE=1 SV=1 - [LST8_MOUSE]	113,67	12,88	1	3	3	4	326	35,8	5,86
P42567	Epidermal growth factor receptor substrate 15 OS=Mus musculus GN=Eps15 PE=1 SV=1 - [EPS15_MOUSE]	113,63	4,01	1	3	3	6	897	98,4	4,60
O88958	Glucosamine-6-phosphate isomerase 1 OS=Mus musculus GN=Gnpda1 PE=2 SV=3 - [GNPI1_MOUSE]	113,31	7,27	1	2	2	5	289	32,5	6,60
P46412	Glutathione peroxidase 3 OS=Mus musculus GN=Gpx3 PE=2 SV=2 - [GPX3_MOUSE]	113,19	15,04	1	3	3	7	226	25,4	8,22
Q8BU31	Ras-related protein Rap-2c OS=Mus musculus GN=Rap2c PE=1 SV=1 - [RAP2C_MOUSE]	112,75	16,39	1	3	3	5	183	20,7	4,94
Q3TYS2	Uncharacterized protein C17orf62 homolog OS=Mus musculus PE=2 SV=2 - [CQ062_MOUSE]	112,48	20,32	1	3	3	8	187	20,9	8,59
Q80X19	Collagen alpha-1(XIV) chain OS=Mus musculus GN=Col14a1 PE=2 SV=2 - [COEA1_MOUSE]	112,05	2,73	1	4	4	6	1797	192,9	5,10
Q9EPA7	Nicotinamide mononucleotide adenylyltransferase 1 OS=Mus musculus GN=Nmnat1 PE=1 SV=2 - [NMNA1_MOUSE]	111,98	10,88	1	3	3	6	285	32,3	9,00
Q9CQS5	Serine/threonine-protein kinase RIO2 OS=Mus musculus GN=Riok2 PE=1 SV=1 - [RIOK2_MOUSE]	111,80	4,75	1	2	2	5	547	62,5	6,43
Q8VCG3	WD repeat-containing protein 74 OS=Mus musculus GN=Wdr74 PE=2 SV=1 - [WDR74_MOUSE]	111,57	11,20	1	3	3	4	384	42,6	7,78
Q8BTZ7	Mannose-1-phosphate guanyltransferase beta OS=Mus musculus GN=Gmppb PE=2 SV=1 - [GMPPB_MOUSE]	110,88	11,94	1	3	3	4	360	39,9	6,74
Q61878	Bone marrow proteoglycan OS=Mus musculus GN=Prg2 PE=1 SV=1 - [PRG2_MOUSE]	110,23	14,35	1	2	2	4	223	24,2	6,20
Q8BU88	39S ribosomal protein L22, mitochondrial OS=Mus musculus GN=Mrpl22 PE=2 SV=1 - [RM22_MOUSE]	108,62	12,14	1	2	2	3	206	23,8	9,86
Q9CR80	Protein FAM32A OS=Mus musculus GN=Fam32a PE=2 SV=1 - [FA32A_MOUSE]	107,77	19,64	1	2	2	6	112	13,2	9,94
P62774	Myotrophin OS=Mus musculus GN=Mtpn PE=1 SV=2 - [MTPN_MOUSE]	107,76	16,95	1	2	2	3	118	12,9	5,52
Q8BV13	COP9 signalosome complex subunit 7b OS=Mus musculus GN=Cops7b PE=1 SV=1 - [CSN7B_MOUSE]	107,58	14,02	1	3	3	5	264	29,7	6,32
P11942	T-cell surface glycoprotein CD3 gamma chain OS=Mus musculus GN=Cd3g PE=1 SV=1 - [CD3G_MOUSE]	107,55	10,99	1	2	2	4	182	20,2	8,85
Q9DB96	Neuroguidin OS=Mus musculus GN=Ngdn PE=1 SV=1 - [NGDN_MOUSE]	107,44	8,89	1	2	2	3	315	35,6	9,58
P42225	Signal transducer and activator of transcription 1 OS=Mus musculus GN=Stat1 PE=1 SV=1 - [STAT1_MOUSE]	107,44	3,07	1	2	2	3	749	87,1	5,58

P53395	Lipoamide acyltransferase component of branched-chain alpha-keto acid dehydrogenase complex, mitochondrial OS=Mus musculus GN=D	107,20	7,88	1	3	3	5	482	53,2	8,60
Q6NSQ7	Protein LTV1 homolog OS=Mus musculus GN=Ltv1 PE=2 SV=2 - [LTV1_MOUSE]	106,21	4,68	1	2	2	4	470	54,0	4,92
Q62311	Transcription initiation factor TFIID subunit 6 OS=Mus musculus GN=Taf6 PE=2 SV=1 - [TAF6_MOUSE]	106,12	8,55	1	4	4	5	678	72,6	8,60
A2ABV5	Mediator of RNA polymerase II transcription subunit 14 OS=Mus musculus GN=Med14 PE=1 SV=1 - [MED14_MOUSE]	105,60	3,50	1	3	3	6	1459	160,9	8,53
Q61466	SWI/SNF-related matrix-associated actin-dependent regulator of chromatin subfamily D member 1 OS=Mus musculus GN=Smarcd1 PE=1 S	105,50	6,21	1	2	2	4	515	58,2	9,25
Q9D211	CDKN2AIP N-terminal-like protein OS=Mus musculus GN=Cdkn2aipnl PE=2 SV=1 - [C2AIL_MOUSE]	105,27	19,83	1	3	3	5	116	13,2	5,29
Q91XI1	tRNA-dihydrouridine synthase 3-like OS=Mus musculus GN=Dus3l PE=2 SV=1 - [DUS3L_MOUSE]	104,42	9,73	1	4	4	6	637	71,0	7,97
Q9JJT9	Phosphorylated adapter RNA export protein OS=Mus musculus GN=Phax PE=1 SV=1 - [PHAX_MOUSE]	104,34	5,71	1	2	2	3	385	43,2	5,34
O54916	RalBP1-associated Eps domain-containing protein 1 OS=Mus musculus GN=Reps1 PE=1 SV=2 - [REPS1_MOUSE]	104,16	3,52	1	2	2	3	795	86,5	5,58
Q8R151	NFX1-type zinc finger-containing protein 1 OS=Mus musculus GN=Znfx1 PE=2 SV=3 - [ZNFX1_MOUSE]	104,02	1,52	1	2	2	4	1909	218,7	7,47
Q8BRF7	Sec1 family domain-containing protein 1 OS=Mus musculus GN=Scfd1 PE=2 SV=1 - [SCFD1_MOUSE]	103,26	8,45	1	3	3	4	639	72,3	6,38
Q08857	Platelet glycoprotein 4 OS=Mus musculus GN=Cd36 PE=2 SV=2 - [CD36_MOUSE]	102,81	4,24	1	2	2	4	472	52,7	8,35
Q06180	Tyrosine-protein phosphatase non-receptor type 2 OS=Mus musculus GN=Ptpn2 PE=2 SV=2 - [PTN2_MOUSE]	102,03	6,90	1	2	2	5	406	47,3	8,37
Q9WVF7	DNA polymerase epsilon catalytic subunit A OS=Mus musculus GN=Pole PE=2 SV=3 - [DPOE1_MOUSE]	101,76	2,37	1	4	5	6	2283	261,9	6,44
Q8R1A4	Dedicator of cytokinesis protein 7 OS=Mus musculus GN=Dock7 PE=1 SV=3 - [DOCK7_MOUSE]	101,74	2,82	1	2	5	13	2130	241,3	6,71
P58501	GC-rich sequence DNA-binding factor 1 OS=Mus musculus GN=Gcfc1 PE=1 SV=2 - [GCFC1_MOUSE]	101,51	5,34	1	4	4	8	917	104,5	5,58
Q9D289	Trafficking protein particle complex subunit 6B OS=Mus musculus GN=Trappc6b PE=2 SV=1 - [TPC6B_MOUSE]	101,40	10,76	1	2	2	3	158	17,9	8,68
Q0VGB7	Serine/threonine-protein phosphatase 4 regulatory subunit 2 OS=Mus musculus GN=Ppp4r2 PE=1 SV=1 - [PP4R2_MOUSE]	101,03	7,19	1	2	2	3	417	46,4	4,56
Q91VW3	SH3 domain-binding glutamic acid-rich-like protein 3 OS=Mus musculus GN=Sh3bgrl3 PE=1 SV=1 - [SH3L3_MOUSE]	101,01	31,18	1	2	2	4	93	10,5	5,14
Q9EPL8	Importin-7 OS=Mus musculus GN=Ipo7 PE=1 SV=2 - [IPO7_MOUSE]	100,33	5,20	1	4	4	5	1038	119,4	4,82
P22599	Alpha-1-antitrypsin 1-2 OS=Mus musculus GN=Serpina1b PE=1 SV=2 - [A1AT2_MOUSE]	100,31	8,72	1	2	2	2	413	45,9	5,54
Q3UHD9	Arf-GAP with GTPase, ANK repeat and PH domain-containing protein 2 OS=Mus musculus GN=Agap2 PE=1 SV=1 - [AGAP2_MOUSE]	100,00	2,02	1	2	2	3	1186	124,4	9,89
Q9WVA4	Transgelin-2 OS=Mus musculus GN=Tagln2 PE=1 SV=4 - [TAGL2_MOUSE]	99,51	18,09	1	3	3	5	199	22,4	8,24
Q99L47	Hsc70-interacting protein OS=Mus musculus GN=St13 PE=2 SV=1 - [F10A1_MOUSE]	99,33	6,20	1	2	2	3	371	41,6	5,26
P03930	ATP synthase protein 8 OS=Mus musculus GN=Mtatp8 PE=1 SV=1 - [ATP8_MOUSE]	98,85	34,33	1	2	2	3	67	7,8	9,88
Q8BG94	COMM domain-containing protein 7 OS=Mus musculus GN=Commd7 PE=2 SV=1 - [COMD7_MOUSE]	98,34	12,00	1	2	2	4	200	22,6	5,94
O35114	Lysosome membrane protein 2 OS=Mus musculus GN=Scarb2 PE=1 SV=3 - [SCRB2_MOUSE]	98,22	4,18	1	2	2	4	478	54,0	5,10
Q8C3Y4	Kinetochore-associated protein 1 OS=Mus musculus GN=Kntc1 PE=1 SV=3 - [KNTC1_MOUSE]	97,95	1,31	1	3	3	5	2207	250,2	6,00
P08226	Apolipoprotein E OS=Mus musculus GN=Apoe PE=1 SV=2 - [APOE_MOUSE]	97,17	10,93	1	3	3	3	311	35,8	5,68
Q924W5	Structural maintenance of chromosomes protein 6 OS=Mus musculus GN=Smc6 PE=2 SV=1 - [SMC6_MOUSE]	96,76	2,28	1	2	2	9	1097	127,1	7,17
P61022	Calcium-binding protein p22 OS=Mus musculus GN=Chp PE=2 SV=2 - [CHP1_MOUSE]	96,55	15,38	1	3	3	5	195	22,4	5,10
P14211	Calreticulin OS=Mus musculus GN=Calr PE=1 SV=1 - [CALR_MOUSE]	96,21	11,78	1	3	3	5	416	48,0	4,49
Q920Q8	Influenza virus NS1A-binding protein homolog OS=Mus musculus GN=Ivns1abp PE=1 SV=2 - [NS1BP_MOUSE]	96,10	3,43	1	2	2	3	642	71,5	5,47
P83882	60S ribosomal protein L36a OS=Mus musculus GN=Rpl36a PE=3 SV=2 - [RL36A_MOUSE]	96,02	29,25	1	5	5	9	106	12,4	10,58
Q9D3E6	Cohesin subunit SA-1 OS=Mus musculus GN=Stag1 PE=1 SV=3 - [STAG1_MOUSE]	95,69	3,74	1	4	5	8	1258	144,3	5,59
P14152	Malate dehydrogenase, cytoplasmic OS=Mus musculus GN=Mdh1 PE=1 SV=3 - [MDHC_MOUSE]	95,50	6,59	1	2	2	5	334	36,5	6,58
Q9DCJ5	NADH dehydrogenase [ubiquinone] 1 alpha subcomplex subunit 8 OS=Mus musculus GN=Ndufa8 PE=1 SV=3 - [NDUA8_MOUSE]	95,47	29,07	1	3	3	6	172	20,0	8,46
Q9CPQ1	Cytochrome c oxidase subunit 6C OS=Mus musculus GN=Cox6c PE=1 SV=3 - [COX6C_MOUSE]	95,31	51,32	1	4	4	10	76	8,5	10,14
P84444	C-C motif chemokine 21a OS=Mus musculus GN=Ccl21a PE=1 SV=1 - [CC21A_MOUSE]	95,08	13,53	2	3	3	10	133	14,5	9,88
Q3USH5	Splicing factor, suppressor of white-apricot homolog OS=Mus musculus GN=Sfswap PE=1 SV=2 - [SFSWA_MOUSE]	95,03	1,80	1	2	2	3	945	104,1	8,05
Q9JLQ0	CD2-associated protein OS=Mus musculus GN=Cd2ap PE=1 SV=3 - [CD2AP_MOUSE]	94,82	10,36	1	6	6	8	637	70,4	6,38
P97822	Acidic leucine-rich nuclear phosphoprotein 32 family member E OS=Mus musculus GN=Anp32e PE=1 SV=2 - [AN32E_MOUSE]	94,73	5,38	1	2	2	3	260	29,6	3,88
P55302	Alpha-2-macroglobulin receptor-associated protein OS=Mus musculus GN=Lrpap1 PE=1 SV=1 - [AMRP_MOUSE]	93,71	11,39	1	3	3	4	360	42,2	7,87
Q9CY66	H/ACA ribonucleoprotein complex subunit 1 OS=Mus musculus GN=Gar1 PE=2 SV=1 - [GAR1_MOUSE]	93,58	9,52	1	2	2	3	231	23,5	11,02
Q8C196	Carbamoyl-phosphate synthase [ammonia], mitochondrial OS=Mus musculus GN=Cps1 PE=1 SV=2 - [CPSM_MOUSE]	93,30	1,27	1	2	2	8	1500	164,5	6,92
P51660	Peroxisomal multifunctional enzyme type 2 OS=Mus musculus GN=Hsd17b4 PE=1 SV=3 - [DHB4_MOUSE]	93,09	6,39	1	3	3	5	735	79,4	8,57
P11031	Activated RNA polymerase II transcriptional coactivator p15 OS=Mus musculus GN=Sub1 PE=1 SV=3 - [TCP4_MOUSE]	92,78	20,47	1	2	2	3	127	14,4	9,60
Q61081	Hsp90 co-chaperone Cdc37 OS=Mus musculus GN=Cdc37 PE=2 SV=1 - [CDC37_MOUSE]	92,12	7,39	1	2	2	4	379	44,6	5,34
Q8C7V3	U3 small nucleolar RNA-associated protein 15 homolog OS=Mus musculus GN=Utp15 PE=2 SV=1 - [UTP15_MOUSE]	91,98	4,73	1	2	2	4	528	59,3	8,79
Q8CBW3	Abl interactor 1 OS=Mus musculus GN=Abi1 PE=1 SV=3 - [ABI1_MOUSE]	91,82	4,16	1	2	2	4	481	52,3	7,64
Q80TR8	Protein VPRBP OS=Mus musculus GN=Vprbp PE=2 SV=4 - [VPRBP_MOUSE]	91,57	1,59	1	2	2	3	1506	168,8	5,08
Q9DAS9	Guanine nucleotide-binding protein G(I)/G(S)/G(O) subunit gamma-12 OS=Mus musculus GN=Gng12 PE=1 SV=3 - [GBG12_MOUSE]	91,35	47,22	1	3	3	4	72	8,0	8,97
Q9D4F8	Gamma-tubulin complex component 4 OS=Mus musculus GN=Tubgcp4 PE=2 SV=2 - [GCP4_MOUSE]	91,04	3,45	1	2	2	3	667	76,1	6,65
Q9CR00	26S proteasome non-ATPase regulatory subunit 9 OS=Mus musculus GN=Psmd9 PE=1 SV=1 - [PSMD9_MOUSE]	90,25	11,71	1	2	2	3	222	24,7	6,43
Q8BHG9	CGG triplet repeat-binding protein 1 OS=Mus musculus GN=Cggbp1 PE=2 SV=1 - [CGBP1_MOUSE]	89,94	19,76	1	3	3	5	167	18,7	8,95
Q9WTX6	Cullin-1 OS=Mus musculus GN=Cul1 PE=1 SV=1 - [CUL1_MOUSE]	89,66	6,06	1	4	4	5	776	89,6	8,00
Q9CQ69	Cytochrome b-c1 complex subunit 8 OS=Mus musculus GN=Uqcrq PE=1 SV=3 - [QCR8_MOUSE]	89,47	29,27	1	3	3	5	82	9,8	10,26
P61202	COP9 signalosome complex subunit 2 OS=Mus musculus GN=Cops2 PE=1 SV=1 - [CSN2_MOUSE]	89,38	6,77	1	3	3	7	443	51,6	5,53
Q9D4H8	Cullin-2 OS=Mus musculus GN=Cul2 PE=1 SV=2 - [CUL2_MOUSE]	88,95	7,65	1	4	5	6	745	86,8	7,01
Q6P5D3	Putative ATP-dependent RNA helicase DHX57 OS=Mus musculus GN=Dhx57 PE=2 SV=2 - [DHX57_MOUSE]	88,45	4,68	1	4	4	5	1388	155,7	7,87
Q8BJU0	Small glutamine-rich tetratricopeptide repeat-containing protein alpha OS=Mus musculus GN=Sgta PE=1 SV=2 - [SGTA_MOUSE]	87,36	5,40	1	2	2	4	315	34,3	5,06
Q8QZV7	Cell cycle regulator Mat89Bb homolog OS=Mus musculus PE=2 SV=2 - [M89BB_MOUSE]	85,96	3,01	1	2	2	5	732	82,7	6,92
Q9JHS3	Ragulator complex protein LAMTOR2 OS=Mus musculus GN=Lamtor2 PE=1 SV=1 - [LTOR2_MOUSE]	85,58	23,20	1	3	3	4	125	13,5	5,40
O35218	Cleavage and polyadenylation specificity factor subunit 2 OS=Mus musculus GN=Cpsf2 PE=1 SV=1 - [CPSF2_MOUSE]	85,52	4,35	1	3	3	6	782	88,3	5,11
Q8K2X3	CST complex subunit STN1 OS=Mus musculus GN=Obfc1 PE=1 SV=2 - [STN1_MOUSE]	85,37	4,50	1	2	2	3	378	43,5	5,77
Q60953	Probable transcription factor PML OS=Mus musculus GN=Pml PE=1 SV=3 - [PML_MOUSE]	85,29	2,82	1	2	2	6	885	98,2	5,63
P25976	Nucleolar transcription factor 1 OS=Mus musculus GN=Ubtf PE=1 SV=1 - [UBF1_MOUSE]	85,14	4,71	1	3	3	5	765	89,5	5,76
O89050	Muskelin OS=Mus musculus GN=Mkln1 PE=2 SV=1 - [MKLN1_MOUSE]	85,09	6,80	1	3	3	5	735	84,8	6,34
P18654	Ribosomal protein S6 kinase alpha-3 OS=Mus musculus GN=Rps6ka3 PE=1 SV=2 - [KS6A3_MOUSE]	85,09	5,41	1	3	3	6	740	83,6	6,89
P42209	Septin-1 OS=Mus musculus GN=Sept1 PE=2 SV=2 - [SEPT1_MOUSE]	83,64	5,46	1	2	2	5	366	42,0	5,82
Q9EQM6	Microprocessor complex subunit DGCR8 OS=Mus musculus GN=Dgcr8 PE=2 SV=2 - [DGCR8_MOUSE]	83,43	1,29	1	2	2	3	773	86,3	6,06
Q9Z0H3	SWI/SNF-related matrix-associated actin-dependent regulator of chromatin subfamily B member 1 OS=Mus musculus GN=Smarcb1 PE=1 S	83,40	7,53	1	2	2	4	385	44,1	6,23
O54833	Casein kinase II subunit alpha' OS=Mus musculus GN=Csnk2a2 PE=2 SV=1 - [CSK22_MOUSE]	83,19	9,14	1	2	3	6	350	41,2	8,56
Q8VEE4	Replication protein A 70 kDa DNA-binding subunit OS=Mus musculus GN=Rpa1 PE=2 SV=1 - [RFA1_MOUSE]	82,55	3,85	1	2	2	3	623	69,0	7,91
P51863	V-type proton ATPase subunit d 1 OS=Mus musculus GN=Atp6v0d1 PE=1 SV=2 - [VA0D1_MOUSE]	82,52	7,69	1	3	3	6	351	40,3	5,00
Q5SRY7	F-box/WD repeat-containing protein 11 OS=Mus musculus GN=Fbxw11 PE=1 SV=1 - [FBW1B_MOUSE]	82,52	4,43	1	2	2	4	542	62,0	7,12
P56391	Cytochrome c oxidase subunit 6B1 OS=Mus musculus GN=Cox6b1 PE=1 SV=2 - [CX6B1_MOUSE]	82,42	29,07	1	2	2	4	86	10,1	8,72
Q9DCC4	Pyrroline-5-carboxylate reductase 3 OS=Mus musculus GN=Pycrl PE=2 SV=2 - [P5CR3_MOUSE]	82,18	7,30	1	2	2	4	274	28,7	7,27
Q9CQ79	Thioredoxin domain-containing protein 9 OS=Mus musculus GN=Txndc9 PE=1 SV=1 - [TXND9_MOUSE]	81,89	15,04	1	3	3	4	226	26,2	5,95
Q7TQH0	Ataxin-2-like protein OS=Mus musculus GN=Atxn2l PE=1 SV=1 - [ATX2L_MOUSE]	81,79	4,19	1	2	2	9	1049	110,6	8,85
Q9ERS2	NADH dehydrogenase [ubiquinone] 1 alpha subcomplex subunit 13 OS=Mus musculus GN=Ndufa13 PE=1 SV=3 - [NDUAD_MOUSE]	81,45	13,89	1	2	2	4	144	16,8	9,48
Q6PGB6	N-alpha-acetyltransferase 50 OS=Mus musculus GN=Naa50 PE=1 SV=1 - [NAA50_MOUSE]	81,40	14,20	1	2	2	3	169	19,4	8,81
Q9CQA3	Succinate dehydrogenase [ubiquinone] iron-sulfur subunit, mitochondrial OS=Mus musculus GN=Sdhb PE=1 SV=1 - [DHSB_MOUSE]	81,18	6,74	1	2	2	3	282	31,8	8,68
Q6NZC7	SEC23-interacting protein OS=Mus musculus GN=Sec23ip PE=1 SV=2 - [S23IP_MOUSE]	80,91	5,01	1	4	4	5	998	110,7	5,94
Q9CQC7	NADH dehydrogenase [ubiquinone] 1 beta subcomplex subunit 4 OS=Mus musculus GN=Ndufb4 PE=1 SV=3 - [NDUB4_MOUSE]	80,67	27,91	1	2	2	3	129	15,1	9,89
P46061	Ran GTPase-activating protein 1 OS=Mus musculus GN=Rangap1 PE=1 SV=2 - [RAGP1_MOUSE]	80,46	3,74	1	2	2	14	589	63,5	4,68
Q91V64	Isochorismatase domain-containing protein 1 OS=Mus musculus GN=Isoc1 PE=2 SV=1 - [ISOC1_MOUSE]	80,22	9,76	1	2	2	2	297	32,0	7,39
P52479	Ubiquitin carboxyl-terminal hydrolase 10 OS=Mus musculus GN=Usp10 PE=1 SV=3 - [UBP10_MOUSE]	80,03	4,42	1	3	3	5	792	87,0	5,17
Q6NVG5	Melanoregulin OS=Mus musculus GN=Mreg PE=1 SV=1 - [MREG_MOUSE]	79,85	8,41	1	2	2	4	214	25,0	7,20
Q9CQB5	CDGSH iron-sulfur domain-containing protein 2 OS=Mus musculus GN=Cisd2 PE=1 SV=1 - [CISD2_MOUSE]	79,84	15,56	1	2	2	4	135	15,2	9,51
Q91VS7	Microsomal glutathione S-transferase 1 OS=Mus musculus GN=Mgst1 PE=1 SV=3 - [MGST1_MOUSE]	79,78	18,06	1	2	2	3	155	17,5	9,66
P09411	Phosphoglycerate kinase 1 OS=Mus musculus GN=Pgk1 PE=1 SV=4 - [PGK1_MOUSE]	79,34	6,71	1	2	2	3	417	44,5	7,90
Q8R0F5	RNA-binding motif protein, X-linked 2 OS=Mus musculus GN=Rbmx2 PE=1 SV=1 - [RBMX2_MOUSE]	78,43	6,44	1	2	2	3	326	37,5	9,72
Q9CWK8	Sorting nexin-2 OS=Mus musculus GN=Snx2 PE=1 SV=2 - [SNX2_MOUSE]	77,64	4,82	1	2	2	2	519	58,4	5,12
Q61543	Golgi apparatus protein 1 OS=Mus musculus GN=Glg1 PE=1 SV=1 - [GSLG1_MOUSE]	77,61	2,04	1	2	2	3	1175	133,6	6,84
Q811M1	Rho GTPase-activating protein 15 OS=Mus musculus GN=Arhgap15 PE=2 SV=1 - [RHG15_MOUSE]	77,56	3,95	1	2	2	2	481	55,3	9,17
P51859	Hepatoma-derived growth factor OS=Mus musculus GN=Hdgf PE=1 SV=2 - [HDGF_MOUSE]	77,18	18,14	1	3	3	4	237	26,3	4,83
Q9D0W5	Peptidyl-prolyl cis-trans isomerase-like 1 OS=Mus musculus GN=Ppil1 PE=2 SV=1 - [PPIL1_MOUSE]	77,12	20,48	1	2	2	4	166	18,2	7,99
Q9BCZ4	Selenoprotein S OS=Mus musculus GN=Sels PE=2 SV=3 - [SELS_MOUSE]	76,22	16,84	1	2	2	3	190	21,5	9,39
Q8BKJ9	NAD-dependent deacetylase sirtuin-7 OS=Mus musculus GN=Sirt7 PE=2 SV=2 - [SIRT7_MOUSE]	75,88	10,20	1	2	2	2	402	45,1	9,58
P59325	Eukaryotic translation initiation factor 5 OS=Mus musculus GN=Eif5 PE=1 SV=1 - [IF5_MOUSE]	75,76	6,99	1	3	3	5	429	48,9	5,52
O88291	Zinc finger protein 326 OS=Mus musculus GN=Znf326 PE=1 SV=1 - [ZN326_MOUSE]	75,58	8,10	1	2	2	3	580	65,2	5,19
Q8K4Q6	Endonuclease 8-like 1 OS=Mus musculus GN=Neil1 PE=2 SV=3 - [NEIL1_MOUSE]	75,27	10,28	1	2	2	3	389	43,6	9,83
Q8R3P6	UPF0464 protein C15orf44 homolog OS=Mus musculus PE=2 SV=1 - [CO044_MOUSE]	75,09	7,57	1	3	3	5	515	57,2	5,17
O55106	Striatin OS=Mus musculus GN=Strn PE=1 SV=2 - [STRN_MOUSE]	74,97	2,95	1	2	2	3	780	85,9	5,27
Q9D735	Uncharacterized protein C19orf43 homolog OS=Mus musculus PE=2 SV=1 - [CS043_MOUSE]	74,84	22,54	1	3	3	4	173	18,4	9,67
Q9CQZ5	NADH dehydrogenase [ubiquinone] 1 alpha subcomplex subunit 6 OS=Mus musculus GN=Ndufa6 PE=1 SV=1 - [NDUA6_MOUSE]	74,43	27,48	1	3	3	6	131	15,3	10,11
Q6PG16	Holliday junction recognition protein OS=Mus musculus GN=Hjurp PE=2 SV=1 - [HJURP_MOUSE]	74,39	3,90	1	2	2	2	667	74,2	9,35
Q8K2T8	RNA polymerase II-associated factor 1 homolog OS=Mus musculus GN=Paf1 PE=2 SV=1 - [PAF1_MOUSE]	73,64	4,67	1	2	2	2	535	60,5	4,65
O35492	Dual specificity protein kinase CLK3 OS=Mus musculus GN=Clk3 PE=1 SV=2 - [CLK3_MOUSE]	73,07	8,46	1	4	4	5	638	73,8	9,94
Q09200	Beta-1,4 N-acetylgalactosaminyltransferase 1 OS=Mus musculus GN=B4galnt1 PE=2 SV=1 - [B4GN1_MOUSE]	72,81	3,94	1	2	2	3	533	59,2	8,59
Q9DC69	NADH dehydrogenase [ubiquinone] 1 alpha subcomplex subunit 9, mitochondrial OS=Mus musculus GN=Ndufa9 PE=1 SV=2 - [NDUA9_MO	71,24	8,49	1	2	2	2	377	42,5	9,74
Q9CQW2	ADP-ribosylation factor-like protein 8B OS=Mus musculus GN=Arl8b PE=2 SV=1 - [ARL8B_MOUSE]	71,05	9,68	1	2	2	5	186	21,5	8,43
Q8BZ20	Poly [ADP-ribose] polymerase 12 OS=Mus musculus GN=Parp12 PE=2 SV=3 - [PAR12_MOUSE]	70,77	4,08	1	2	2	4	711	79,9	8,54
Q5NCF2	Trafficking protein particle complex subunit 1 OS=Mus musculus GN=Trappc1 PE=1 SV=1 - [TPPC1_MOUSE]	70,65	13,79	1	2	2	3	145	16,9	9,16
Q9DBU6	Serine/Arginine-related protein 53 OS=Mus musculus GN=Rsrc1 PE=1 SV=1 - [RSRC1_MOUSE]	69,65	11,98	1	2	2	3	334	38,6	11,15
Q8K114	Integrator complex subunit 9 OS=Mus musculus GN=Ints9 PE=2 SV=1 - [INT9_MOUSE]	69,58	5,93	1	3	3	7	658	74,0	6,40
Q60854	Serpin B6 OS=Mus musculus GN=Serpinb6 PE=2 SV=1 - [SPB6_MOUSE]	69,27	7,94	1	2	2	2	378	42,6	5,74
Q8C4J7	Transducin beta-like protein 3 OS=Mus musculus GN=Tbl3 PE=2 SV=1 - [TBL3_MOUSE]	69,07	3,62	1	2	2	2	801	88,2	6,81
P13597	Intercellular adhesion molecule 1 OS=Mus musculus GN=Icam1 PE=1 SV=1 - [ICAM1_MOUSE]	68,74	3,54	1	2	2	3	537	58,8	6,13
Q9CX11	rRNA-processing protein UTP23 homolog OS=Mus musculus GN=Utp23 PE=2 SV=1 - [UTP23_MOUSE]	68,72	14,06	1	2	2	4	249	28,4	10,32

Q9CR08	Ribonuclease P protein subunit p29 OS=Mus musculus GN=Pop4 PE=2 SV=1 - [RPP29_MOUSE]	68,51	9,05	1	2	2	2	221	25,6	9,74
Q9CUX1	52 kDa repressor of the inhibitor of the protein kinase OS=Mus musculus GN=Prkrir PE=2 SV=2 - [P52K_MOUSE]	68,35	2,64	1	2	2	4	758	87,0	5,92
Q6PGG6	Guanine nucleotide-binding protein-like 3-like protein OS=Mus musculus GN=Gnl3l PE=2 SV=1 - [GNL3L_MOUSE]	67,55	5,37	1	2	2	3	577	65,2	8,60
Q9CSH3	Exosome complex exonuclease RRP44 OS=Mus musculus GN=Dis3 PE=2 SV=4 - [RRP44_MOUSE]	67,30	3,03	1	2	2	5	958	108,8	7,53
Q07832	Serine/threonine-protein kinase PLK1 OS=Mus musculus GN=Plk1 PE=1 SV=2 - [PLK1_MOUSE]	67,06	3,81	1	2	2	2	603	68,3	8,72
Q6ZWM4	N-alpha-acetyltransferase 38, NatC auxiliary subunit OS=Mus musculus GN=Naa38 PE=2 SV=3 - [NAA38_MOUSE]	66,97	27,08	1	2	2	3	96	10,4	4,48
Q8CAS9	Poly [ADP-ribose] polymerase 9 OS=Mus musculus GN=Parp9 PE=1 SV=2 - [PARP9_MOUSE]	66,78	2,42	1	2	2	2	866	96,6	7,09
Q9D1Q6	Endoplasmic reticulum resident protein 44 OS=Mus musculus GN=Erp44 PE=1 SV=1 - [ERP44_MOUSE]	66,77	8,37	1	2	3	6	406	46,8	5,27
P70280	Vesicle-associated membrane protein 7 OS=Mus musculus GN=Vamp7 PE=1 SV=1 - [VAMP7_MOUSE]	66,02	11,36	1	2	2	5	220	25,0	8,60
P56135	ATP synthase subunit f, mitochondrial OS=Mus musculus GN=Atp5j2 PE=1 SV=3 - [ATPK_MOUSE]	65,71	26,14	1	2	2	4	88	10,3	9,95
Q91VK1	Basic leucine zipper and W2 domain-containing protein 2 OS=Mus musculus GN=Bzw2 PE=1 SV=1 - [BZW2_MOUSE]	65,59	3,82	1	2	2	4	419	48,0	6,68
Q8VI33	Transcription initiation factor TFIID subunit 9 OS=Mus musculus GN=Taf9 PE=2 SV=1 - [TAF9_MOUSE]	65,56	21,59	1	4	4	5	264	29,0	7,91
Q8CH02	SURP and G-patch domain-containing protein 1 OS=Mus musculus GN=Sugp1 PE=1 SV=1 - [SUGP1_MOUSE]	65,20	4,67	1	2	2	3	643	72,6	7,64
Q7TSH2	Phosphorylase b kinase regulatory subunit beta OS=Mus musculus GN=Phkb PE=1 SV=1 - [KPBB_MOUSE]	65,18	2,49	1	2	2	2	1085	123,8	6,83
Q9Z2X8	Kelch-like ECH-associated protein 1 OS=Mus musculus GN=Keap1 PE=1 SV=1 - [KEAP1_MOUSE]	64,99	5,45	1	3	3	4	624	69,5	6,44
Q9CQX4	PCNA-associated factor OS=Mus musculus GN=Paf PE=2 SV=1 - [PAF_MOUSE]	64,49	30,00	1	2	2	4	110	12,0	9,89
O35683	NADH dehydrogenase [ubiquinone] 1 alpha subcomplex subunit 1 OS=Mus musculus GN=Ndufa1 PE=2 SV=1 - [NDUA1_MOUSE]	64,13	30,00	1	2	2	3	70	8,1	9,48
Q9WV60	Glycogen synthase kinase-3 beta OS=Mus musculus GN=Gsk3b PE=1 SV=2 - [GSK3B_MOUSE]	63,54	6,19	1	2	2	3	420	46,7	8,78
O35864	COP9 signalosome complex subunit 5 OS=Mus musculus GN=Cops5 PE=1 SV=3 - [CSN5_MOUSE]	63,45	15,57	1	4	4	7	334	37,5	6,54
Q99K85	Phosphoserine aminotransferase OS=Mus musculus GN=Psat1 PE=1 SV=1 - [SERC_MOUSE]	63,42	5,41	1	2	2	2	370	40,4	8,03
Q60994	Adiponectin OS=Mus musculus GN=Adipoq PE=1 SV=2 - [ADIPO_MOUSE]	63,42	8,50	1	2	2	3	247	26,8	5,57
Q64339	Ubiquitin-like protein ISG15 OS=Mus musculus GN=Isg15 PE=1 SV=4 - [ISG15_MOUSE]	63,37	16,77	1	2	2	5	161	17,9	7,90
Q9QZB9	Dynactin subunit 5 OS=Mus musculus GN=Dctn5 PE=1 SV=1 - [DCTN5_MOUSE]	62,55	9,89	1	2	2	4	182	20,1	8,02
Q9R1R2	Tripartite motif-containing protein 3 OS=Mus musculus GN=Trim3 PE=1 SV=1 - [TRIM3_MOUSE]	62,32	2,82	1	2	2	2	744	80,7	7,81
P53349	Mitogen-activated protein kinase kinase kinase 1 OS=Mus musculus GN=Map3k1 PE=1 SV=3 - [M3K1_MOUSE]	61,73	2,21	1	2	2	3	1493	161,2	7,90
Q9CR68	Cytochrome b-c1 complex subunit Rieske, mitochondrial OS=Mus musculus GN=Uqcrfs1 PE=1 SV=1 - [UCRI_MOUSE]	60,91	12,77	1	2	3	5	274	29,3	8,70
Q99KH8	Serine/threonine-protein kinase 24 OS=Mus musculus GN=Stk24 PE=1 SV=1 - [STK24_MOUSE]	60,19	4,64	1	2	2	3	431	47,9	5,43
Q8BGD9	Eukaryotic translation initiation factor 4B OS=Mus musculus GN=Eif4b PE=1 SV=1 - [IF4B_MOUSE]	59,76	2,29	1	2	2	3	611	68,8	5,67
Q8CIN4	Serine/threonine-protein kinase PAK 2 OS=Mus musculus GN=Pak2 PE=1 SV=1 - [PAK2_MOUSE]	59,60	10,31	1	3	3	5	524	57,9	5,77
Q8VDM1	Zinc finger CCCH-type with G patch domain-containing protein OS=Mus musculus GN=Zgpat PE=2 SV=1 - [ZGPAT_MOUSE]	59,35	7,24	1	3	3	6	511	56,4	5,33
P06330	Ig heavy chain V region AC38 205.12 OS=Mus musculus PE=1 SV=1 - [HVM51_MOUSE]	59,31	24,58	1	2	2	5	118	12,9	7,11
Q8K2L8	Trafficking protein particle complex subunit 12 OS=Mus musculus GN=Ttc15 PE=1 SV=2 - [TPC12_MOUSE]	58,97	3,64	1	3	3	5	797	87,6	4,81
P35293	Ras-related protein Rab-18 OS=Mus musculus GN=Rab18 PE=2 SV=2 - [RAB18_MOUSE]	58,28	10,68	1	2	2	3	206	23,0	5,36
Q80U70	Polycomb protein Suz12 OS=Mus musculus GN=Suz12 PE=1 SV=2 - [SUZ12_MOUSE]	58,27	2,56	1	2	2	2	741	83,0	8,81
Q91WD5	NADH dehydrogenase [ubiquinone] iron-sulfur protein 2, mitochondrial OS=Mus musculus GN=Ndufs2 PE=1 SV=1 - [NDUS2_MOUSE]	58,02	3,67	1	2	2	2	463	52,6	6,99
P20357	Microtubule-associated protein 2 OS=Mus musculus GN=Map2 PE=1 SV=2 - [MAP2_MOUSE]	57,75	1,59	1	2	2	3	1828	199,0	4,91
P11276	Fibronectin OS=Mus musculus GN=Fn1 PE=1 SV=4 - [FINC_MOUSE]	56,08	0,89	1	2	2	3	2477	272,4	5,59
Q99LC2	Cleavage stimulation factor subunit 1 OS=Mus musculus GN=Cstf1 PE=2 SV=1 - [CSTF1_MOUSE]	55,92	6,26	1	2	2	4	431	48,4	6,58
Q8K339	DNA/RNA-binding protein KIN17 OS=Mus musculus GN=Kin PE=2 SV=1 - [KIN17_MOUSE]	55,68	11,51	1	2	3	4	391	44,7	9,04
Q02614	SAP30-binding protein OS=Mus musculus GN=Sap30bp PE=2 SV=2 - [S30BP_MOUSE]	54,83	11,69	1	2	2	3	308	33,8	4,87
Q9D379	Epoxide hydrolase 1 OS=Mus musculus GN=Ephx1 PE=1 SV=2 - [HYEP_MOUSE]	54,75	9,23	1	4	4	5	455	52,5	8,35
Q80XC6	UPF0614 protein C14orf102 homolog OS=Mus musculus PE=2 SV=3 - [CN102_MOUSE]	54,21	2,13	1	2	2	2	1172	133,4	7,93
Q63829	COMM domain-containing protein 3 OS=Mus musculus GN=Commd3 PE=2 SV=1 - [COMD3_MOUSE]	53,93	22,05	1	3	3	3	195	22,0	5,59
Q61166	Microtubule-associated protein RP/EB family member 1 OS=Mus musculus GN=Mapre1 PE=1 SV=3 - [MARE1_MOUSE]	53,90	13,06	1	2	2	3	268	30,0	5,22
Q9D0F4	NF-kappa-B-activating protein OS=Mus musculus GN=Nkap PE=1 SV=1 - [NKAP_MOUSE]	53,81	6,27	1	2	2	4	415	47,2	10,13
Q8BGF7	PAB-dependent poly(A)-specific ribonuclease subunit 2 OS=Mus musculus GN=Pan2 PE=2 SV=1 - [PAN2_MOUSE]	52,93	3,67	1	3	4	5	1200	135,2	5,96
Q6P1G0	HEAT repeat-containing protein 6 OS=Mus musculus GN=Heatr6 PE=2 SV=1 - [HEAT6_MOUSE]	52,88	2,62	1	2	2	6	1184	128,8	7,24
P62897	Cytochrome c, somatic OS=Mus musculus GN=Cycs PE=1 SV=2 - [CYC_MOUSE]	52,64	23,81	1	2	2	2	105	11,6	9,58
Q3TXT3	SOSS complex subunit C OS=Mus musculus GN=Ssbip1 PE=2 SV=1 - [SOSSC_MOUSE]	52,35	25,96	1	2	2	6	104	11,4	9,25
Q8K4F6	Putative methyltransferase NSUN5 OS=Mus musculus GN=Nsun5 PE=2 SV=2 - [NSUN5_MOUSE]	51,74	6,67	1	2	2	3	465	51,0	9,19
Q9CWL8	Beta-catenin-like protein 1 OS=Mus musculus GN=Ctnnbl1 PE=1 SV=1 - [CTBL1_MOUSE]	51,40	3,37	1	2	2	2	563	64,9	5,07
O35066	Kinesin-like protein KIF3C OS=Mus musculus GN=Kif3c PE=2 SV=3 - [KIF3C_MOUSE]	51,36	5,15	1	2	2	3	796	89,9	8,06
Q9CQJ4	E3 ubiquitin-protein ligase RING2 OS=Mus musculus GN=Rnf2 PE=1 SV=1 - [RING2_MOUSE]	50,91	8,33	1	2	2	4	336	37,6	6,84
Q99N96	39S ribosomal protein L1, mitochondrial OS=Mus musculus GN=Mrpl1 PE=1 SV=2 - [RM01_MOUSE]	50,54	8,04	1	2	2	2	336	37,6	8,72
Q91VJ5	Polyglutamine-binding protein 1 OS=Mus musculus GN=Pqbp1 PE=2 SV=1 - [PQBP1_MOUSE]	49,88	12,93	1	2	2	4	263	30,6	6,23
Q61214	Dual specificity tyrosine-phosphorylation-regulated kinase 1A OS=Mus musculus GN=Dyrk1a PE=1 SV=1 - [DYR1A_MOUSE]	49,71	5,50	1	3	3	4	763	85,4	8,75
Q9CPS7	RNA-binding protein PNO1 OS=Mus musculus GN=Pno1 PE=2 SV=1 - [PNO1_MOUSE]	48,75	18,55	1	2	2	3	248	27,4	9,79
Q3U2K0	Protein FAM193B OS=Mus musculus GN=Fam193b PE=2 SV=2 - [F193B_MOUSE]	48,68	5,27	1	2	2	2	892	95,0	7,96
Q3UZ01	RNA-binding protein 40 OS=Mus musculus GN=Rnpc3 PE=2 SV=2 - [RBM40_MOUSE]	47,52	5,06	1	2	2	3	514	57,9	7,90
P00493	Hypoxanthine-guanine phosphoribosyltransferase OS=Mus musculus GN=Hprt1 PE=1 SV=3 - [HPRT_MOUSE]	46,89	9,63	1	2	2	2	218	24,6	6,68
Q9D855	Cytochrome b-c1 complex subunit 7 OS=Mus musculus GN=Uqcrb PE=1 SV=3 - [QCR7_MOUSE]	46,83	22,52	1	2	2	3	111	13,5	9,11
Q9CS00	Uncharacterized protein C19orf29 homolog OS=Mus musculus PE=1 SV=2 - [CS029_MOUSE]	46,60	4,53	1	3	3	3	772	90,6	9,79
Q64521	Glycerol-3-phosphate dehydrogenase, mitochondrial OS=Mus musculus GN=Gpd2 PE=1 SV=2 - [GPDM_MOUSE]	46,57	3,71	1	2	2	2	727	80,9	6,61
Q8BHZ4	Zinc finger protein 592 OS=Mus musculus GN=Znf592 PE=1 SV=3 - [ZN592_MOUSE]	46,29	2,77	1	2	2	3	1262	137,4	7,96
P35585	AP-1 complex subunit mu-1 OS=Mus musculus GN=Ap1m1 PE=1 SV=3 - [AP1M1_MOUSE]	46,16	12,06	1	4	4	5	423	48,5	7,30
P10300	T-cell surface glycoprotein CD8 beta chain OS=Mus musculus GN=Cd8b PE=1 SV=1 - [CD8B_MOUSE]	46,01	8,45	1	2	2	5	213	24,3	9,76
Q8CFE2	UPF0609 protein C4orf27 homolog OS=Mus musculus PE=2 SV=1 - [CD027_MOUSE]	45,80	6,36	1	2	2	3	346	39,3	7,69
Q9D051	Pyruvate dehydrogenase E1 component subunit beta, mitochondrial OS=Mus musculus GN=Pdhb PE=1 SV=1 - [ODPB_MOUSE]	45,65	8,64	1	3	3	4	359	38,9	6,87
Q9CQJ2	PIH1 domain-containing protein 1 OS=Mus musculus GN=Pih1d1 PE=2 SV=1 - [PIHD1_MOUSE]	44,57	8,97	1	2	2	3	290	32,2	5,21
Q3TZ89	Protein transport protein Sec31B OS=Mus musculus GN=Sec31b PE=1 SV=2 - [SC31B_MOUSE]	43,59	2,33	1	2	3	6	1158	125,6	7,77
Q9CQQ8	U6 snRNA-associated Sm-like protein LSm7 OS=Mus musculus GN=Lsm7 PE=2 SV=1 - [LSM7_MOUSE]	42,94	14,56	1	2	2	2	103	11,6	5,27
Q9D9K3	Cell death regulator Aven OS=Mus musculus GN=Aven PE=1 SV=2 - [AVEN_MOUSE]	42,13	11,99	1	2	2	4	342	37,2	4,98
Q7TQK5	Coiled-coil domain-containing protein 93 OS=Mus musculus GN=Ccdc93 PE=2 SV=1 - [CCD93_MOUSE]	41,89	4,61	1	2	2	3	629	72,6	8,29
Q93092	Transaldolase OS=Mus musculus GN=Taldo1 PE=1 SV=2 - [TALDO_MOUSE]	41,25	6,53	1	2	2	4	337	37,4	7,03
Q99KG3	RNA-binding protein 10 OS=Mus musculus GN=Rbm10 PE=1 SV=1 - [RBM10_MOUSE]	41,22	4,09	1	2	3	3	930	103,4	5,92
Q8BZN6	Dedicator of cytokinesis protein 10 OS=Mus musculus GN=Dock10 PE=1 SV=3 - [DOC10_MOUSE]	39,38	1,53	1	3	3	3	2150	245,6	7,05
Q3TWF6	WD repeat-containing protein 70 OS=Mus musculus GN=Wdr70 PE=1 SV=1 - [WDR70_MOUSE]	38,44	4,26	1	3	3	3	657	73,1	5,97
Q60864	Stress-induced-phosphoprotein 1 OS=Mus musculus GN=Stip1 PE=1 SV=1 - [STIP1_MOUSE]	36,30	4,05	1	2	2	3	543	62,5	6,80
Q62276	Mediator of RNA polymerase II transcription subunit 22 OS=Mus musculus GN=Med22 PE=2 SV=2 - [MED22_MOUSE]	36,26	12,50	1	2	2	2	200	22,3	4,68
Q99MQ4	Asporin OS=Mus musculus GN=Aspn PE=1 SV=1 - [ASPN_MOUSE]	36,01	6,70	1	2	2	2	373	42,5	8,57
Q8BSS9	Liprin-alpha-2 OS=Mus musculus GN=Ppfia2 PE=1 SV=2 - [LIPA2_MOUSE]	35,22	1,83	1	2	2	2	1257	143,1	6,11
Q8K3W3	Protein CASC3 OS=Mus musculus GN=Casc3 PE=1 SV=3 - [CASC3_MOUSE]	35,19	3,87	1	2	2	3	698	75,7	6,44
Q9DCU6	39S ribosomal protein L4, mitochondrial OS=Mus musculus GN=Mrpl4 PE=2 SV=1 - [RM04_MOUSE]	34,23	8,16	1	2	2	2	294	33,1	9,82
P70218	Mitogen-activated protein kinase kinase kinase kinase 1 OS=Mus musculus GN=Map4k1 PE=1 SV=1 - [M4K1_MOUSE]	33,94	3,14	1	2	2	4	827	91,5	7,85
Q80X41	Serine/threonine-protein kinase VRK1 OS=Mus musculus GN=Vrk1 PE=1 SV=2 - [VRK1_MOUSE]	33,30	5,00	1	2	2	2	440	49,7	8,94
Q99KY4	Cyclin-G-associated kinase OS=Mus musculus GN=Gak PE=1 SV=2 - [GAK_MOUSE]	32,70	2,68	1	3	3	4	1305	143,6	5,73
Q9CUU3	Synaptonemal complex protein 2 OS=Mus musculus GN=Sycp2 PE=1 SV=2 - [SYCP2_MOUSE]	31,74	1,00	1	2	2	4	1500	172,0	7,93
Q91W89	Alpha-mannosidase 2C1 OS=Mus musculus GN=Man2c1 PE=1 SV=1 - [MA2C1_MOUSE]	31,50	2,02	1	2	2	2	1039	115,6	6,51
O88384	Vesicle transport through interaction with t-SNAREs homolog 1B OS=Mus musculus GN=Vti1b PE=1 SV=1 - [VTI1B_MOUSE]	31,30	10,78	1	2	2	2	232	26,7	8,79
B2RXR6	Serine/threonine-protein phosphatase 6 regulatory ankyrin repeat subunit B OS=Mus musculus GN=Ankrd44 PE=2 SV=1 - [ANR44_MOUSE	30,55	2,92	1	2	2	4	993	107,3	6,42
Q8BP47	Asparaginyl-tRNA synthetase, cytoplasmic OS=Mus musculus GN=Nars PE=1 SV=2 - [SYNC_MOUSE]	30,50	4,65	1	2	2	2	559	64,2	5,86
Q9JHR7	Insulin-degrading enzyme OS=Mus musculus GN=Ide PE=2 SV=1 - [IDE_MOUSE]	30,00	1,86	1	2	2	2	1019	117,7	6,54
Q5HZJ0	Ribonuclease 3 OS=Mus musculus GN=Drosha PE=1 SV=1 - [RNC_MOUSE]	29,80	1,82	1	2	2	2	1373	158,7	7,87
Q61792	LIM and SH3 domain protein 1 OS=Mus musculus GN=Lasp1 PE=1 SV=1 - [LASP1_MOUSE]	29,21	9,89	1	2	2	3	263	30,0	7,05
Q3UGM2	Serine/threonine-protein kinase Nek10 OS=Mus musculus GN=Nek10 PE=2 SV=2 - [NEK10_MOUSE]	28,72	1,62	1	1	2	2	1111	126,0	6,57
P97440	Histone RNA hairpin-binding protein OS=Mus musculus GN=Slbp PE=1 SV=1 - [SLBP_MOUSE]	28,23	14,18	1	2	2	3	275	31,6	6,80
Q9JK23	Proteasome assembly chaperone 1 OS=Mus musculus GN=Psmg1 PE=1 SV=1 - [PSMG1_MOUSE]	28,17	9,69	1	2	2	2	289	33,1	6,37
Q9CXK8	60S ribosome subunit biogenesis protein NIP7 homolog OS=Mus musculus GN=Nip7 PE=2 SV=1 - [NIP7_MOUSE]	27,59	13,33	1	2	2	3	180	20,4	8,24
Q8BTT6	Digestive organ expansion factor homolog OS=Mus musculus GN=Def PE=2 SV=2 - [DEF_MOUSE]	27,42	3,24	1	2	2	3	772	88,8	5,74
Q8K2A7	Integrator complex subunit 10 OS=Mus musculus GN=Ints10 PE=1 SV=3 - [INT10_MOUSE]	27,32	2,39	1	2	2	2	710	82,0	7,33
P24161	T-cell surface glycoprotein CD3 zeta chain OS=Mus musculus GN=Cd247 PE=1 SV=1 - [CD3Z_MOUSE]	26,29	16,46	1	2	2	2	164	18,6	9,28
Q3UZY0	Protein SFI1 homolog OS=Mus musculus GN=Sfi1 PE=2 SV=1 - [SFI1_MOUSE]	25,84	1,73	1	2	2	2	1216	143,9	10,62
Q3UX61	N-alpha-acetyltransferase 11, NatA catalytic subunit OS=Mus musculus GN=Naa11 PE=2 SV=1 - [NAA11_MOUSE]	25,81	14,68	2	2	2	2	218	24,7	5,63
Q9CZ82	Mediator of RNA polymerase II transcription subunit 18 OS=Mus musculus GN=Med18 PE=2 SV=1 - [MED18_MOUSE]	25,67	12,50	1	2	2	2	208	23,6	6,54
P01851	T-cell receptor beta-2 chain C region OS=Mus musculus PE=1 SV=1 - [TCB2_MOUSE]	25,51	16,18	2	2	2	2	173	19,3	8,44
O35071	Kinesin-like protein KIF1C OS=Mus musculus GN=Kif1c PE=1 SV=2 - [KIF1C_MOUSE]	23,77	1,82	1	2	2	2	1100	122,4	7,03

<u>Supplementary table S3</u>

Total miRNA. Intensities as measured by Gene Scanner 3000

1: Raw data

2: Background substracted data

3: Global normalization

	**Customer sample name**	spleenExo 2			spleenExo 3			ThymusExo 1 (T1)			ThymusExo2 (T2)		
		1	2	3	1	2	3	1	2	3	1	2	3
mmu-miR-6240	MIMAT0024861	34031,1084	33892,239	17508,09384	10246,80104	10175,4666	13602,00085	46284	46208,46196	32187,69113	40810	40738,82893	30036,04625
mmu-miR-652-5p	MIMAT0017260	256,96383	118.094426	61,005362	93,775036	22,440599	29,997352	5364,235596	5288,697557	3683,978133	5375,033686	5303,862617	3910,447772
mmu-miR-1892	MIMAT0007871	202,373637	63,504233	32,805094	98,19596	26,861523	35,906998	1831,901559	1756,36352	1223,440125	1846,881049	1775,70998	1309,200791
mmu-miR-710	MIMAT0003500	159,216183	20,346779	10,510764	75,093002			378,896387	303,358347	211,312049	282,020154	210,849085	155,455447
mmu-miR-6930-5p	MIMAT0027760	160,95715	22,087746	11,410115	78,495158	7,160721	9,572056	567,927322	492,389282	342,986403	557,933466	486,762396	358,881643
mmu-miR-6933-5p	MIMAT0027766	170,651427	31,782023	16,417996	79,118613	7,784177	10,405457	554,621598	479,083558	333,717959	548,644285	477,473216	352,032888
mmu-miR-680	MIMAT0003457	431,197297	292,327893	151,011097	159,113128	87,778692	117,337699	3862,604471	3787,066432	2637,978401	3980,072183	3908,901114	2881,966362
mmu-miR-6404	MIMAT0025156	418,355283	279,485879	144,377154	153,661574	82,327137	110,050363	3111,90515	3036,367111	2115,059506	3824,680025	3753,508956	2767,398365
mmu-miR-7224-5p	MIMAT0028416	201,953401	63,083997	32,588008	88,591804	17,257368	23,068694	777,222504	701,684464	488,776338	765,456474	694,285405	511,88483
mmu-miR-6947-5p	MIMAT0027794	190,163507	51,294103	26,49757	82,794848	11,460412	15,319644	494,353126	418,815086	291,736406	652,769631	581,598562	428,802736
mmu-miR-705	MIMAT0003495	297,149791	158,280387	81,764674	121,171929	49,837492	66,620002	1548,082943	1472,544904	1025,738978	1810,791471	1739,620402	1282,592559
mmu-miR-7231-5p	MIMAT0028430	321,02725	182,157846	94,099321	129,542573	58,208136	77,809416	1642,979233	1567,441194	1091,841426	1924,132729	1852,961659	1366,157142
mmu-miR-6244	MIMAT0024864	679,496315	540,626911	279,277704	232,378691	161,044254	215,27505	5256,302355	5180,764315	3608,794462	4765,336396	4694,165327	3460,928322
mmu-miR-7046-5p	MIMAT0027996	369,325775	230,456371	119,049431	191,479939	120,144953	160,603127	2898,62648	2823,088441	1966,49477	2716,391593	2645,220523	1950,276138
mmu-miR-6900-5p	MIMAT0027700	246,894829	108,025425	55,803905	102,707137	31,372701	41,937291	1123,029618	1047,491579	729,657166	925,826628	854,655558	630,123019
mmu-miR-7030-5p	MIMAT0027964	283,001174	144,13177	74,455764	125,358091	54,023654	72,215832	1495,12309	1419,58505	988,848432	1477,026522	1405,855453	1036,513334
mmu-miR-5129-5p	MIMAT0020640	208,523505	69,654101	35,982	88,493232	17,158796	22,936929	650,243975	574,705935	400,326182	626,411593	555,240524	409,369402
mmu-miR-299b-3p	MIMAT0022837	151,092641	12,223237	6,314294	72,934213			137,843872	62,305833	43,400728	126,171805	55,000736	40,551108
mmu-miR-7684-3p	MIMAT0029891	756,81362	617,944216	319,218371	272,524841	201,190404	268,940203	5415,607678	5340,069639	3719,762677	5304,77278	5233,601711	3858,6456
mmu-miR-6954-5p	MIMAT0027808	260,907117	122,037713	63,042389	117,967347	46,632911	62,336295	1030,7298	955,191761	665,363357	1320,697673	1249,526603	921,25473
mmu-miR-92a-2-5p	MIMAT0004635	484,808104	345,9387	178,705432	190,895252	119,560816	159,822284	2961,753276	2886,215236	2010,467361	3125,926235	3054,755166	2252,219071
mmu-miR-6979-5p	MIMAT0027860	400,586118	261,716714	135,197937	162,351271	91,016834	121,666269	1906,750409	1831,212369	1275,578014	2629,487591	2558,316522	1886,203295
mmu-miR-6546-3p	MIMAT0029793	279,1545	140,285097	72,468645	126,275644	54,941207	73,442366	1413,597814	1338,059775	932,059907	1202,516034	1131,344965	834,121416
mmu-miR-200a-3p	MIMAT0000519	143,415585			77,181474	5,847037	7,815995	147,787098	72,249059	50,326938	130,860977	59,689908	44,008355
mmu-miR-3101-3p	MIMAT0014922	175,576456	36,707052	18,962173	82,245953	10,911516	14,585911	365,278954	289,740915	201,826477	335,093697	263,922628	194,585669
mmu-miR-1934-3p	MIMAT0017341	264,48936	125,619956	64,892909	128,180371	56,845934	75,9885	1122,599496	1047,061457	729,357554	1333,678775	1262,507706	930,825476
mmu-miR-6910-5p	MIMAT0027720	355,252363	216,382959	111,779371	151,6233	80,288864	107,325711	1770,9917	1695,45366	1181,011798	1920,83114	1849,660071	1363,722937
mmu-miR-6913-5p	MIMAT0027726	176,488044	37,61864	19,433082	78,333357	6,99892	9,355769	304,248971	228,710932	159,314474	294,776624	223,605555	164,860576
mmu-miR-5620-5p	MIMAT0022367	796,434657	657,565253	339,685854	299,564407	228,22997	305,085199	5401,825072	5326,287033	3710,162048	4801,976718	4730,805648	3487,942608
mmu-miR-181c-5p	MIMAT0000674	163,037129	24,167725	12,484593	82,888995	11,554558	15,445494	315,483675	239,945635	167,140296	248,64677	177,4757	130,849818
mmu-miR-6236	MIMAT0024857	887,819237	748,949833	386,893411	253,629926	182,29549	243,682526	4790,060394	4714,522354	3284,021647	4683,668006	4612,496937	3400,715606
mmu-miR-344g-3p	MIMAT0014930	205,023099	66,153695	34,173756	115,181578	43,847142	58,612433	834,463673	758,925633	528,649144	657,848774	586,677705	432,547501
mmu-miR-433-5p	MIMAT0001419	149,395876			77,562812	6,228375	8,325747	145,907229	70,369189	49,017466	120,533964	49,362895	36,394423
mmu-miR-128-3p	MIMAT0000140	164,251339	25,381935	13,111831	79,51747	8,183033	10,938626	275,47543	199,93739	139,271525	210,921386	139,750317	103,035534
mmu-miR-5627-5p	MIMAT0022385	648,229307	509,359903	263,125755	214,260715	142,926278	191,055942	3508,770419	3433,232379	2391,506203	3006,580937	2935,409867	2164,22781
mmu-miR-5114	MIMAT0020622	183,604663	44,735259	23,109394	93,987404	22,652967	30,281233	471,209708	395,671669	275,615265	392,844412	321,673343	237,164289
mmu-miR-8090	MIMAT0031391	430,407378	291,537974	150,603039	148,753671	77,419234	103,48975	1744,316936	1668,778897	1162,430806	1762,164691	1690,993622	1246,74086
mmu-miR-328-5p	MIMAT0017030	2372,157612	2233,288208	1153,674725	709,255897	637,92146	852,738119	12400,72511	12325,18707	8585,425651	14068,38788	13997,21681	10319,91008
mmu-miR-7036a-5p	MIMAT0027976	396,180972	257,311568	132,922321	169,943133	98,608696	131,81465	1740,538326	1665,000286	1159,798718	1859,842058	1788,670988	1318,756723
mmu-miR-1224-5p	MIMAT0005460	701,010294	562,14089	290,391421	274,245906	202,911469	271,240827	3883,933626	3808,395587	2652,835772	3400,341871	3329,170802	2454,541056
mmu-miR-677-3p	MIMAT0017246	473,52252	334,653116	172,875512	157,636168	86,301731	115,36338	2063,911799	1988,373759	1385,052817	1746,038107	1674,867037	1234,851004
mmu-miR-3102-5p	MIMAT0014933	2425,918136	2287,048732	1181,446402	876,681689	805,347252	1076,543655	14598,85456	14523,31652	10116,58918	13757,84447	13686,6734	10090,9517
mmu-miR-493-3p	MIMAT0004888	273,259214	134,38981	69,423251	110,388885	39,054448	52,205825	813,009342	737,471302	513,704579	765,902061	694,730991	512,213354
mmu-miR-6415	MIMAT0025169	149,408421			79,999061	8,664624	11,58239	152,608938	77,070898	53,685714	130,183029	59,01196	43,508515
mmu-miR-6917-5p	MIMAT0027734	279,395285	140,525881	72,59303	119,925836	48,5914	64,954295	770,264456	694,726416	483,929531	891,635689	820,464619	604,914621
mmu-miR-6997-5p	MIMAT0027896	203,258867	64,389463	33,262387	96,763973	25,429537	33,992798	399,155076	323,617036	225,423759	483,447997	412,276928	303,964772
mmu-miR-6922-5p	MIMAT0027744	241,424512	102,555108	52,978042	106,033484	34,699047	46,383766	611,177845	535,639805	373,113665	619,228026	548,056957	404,073081
mmu-miR-6941-5p	MIMAT0027782	215,423776	76,554372	39,54655	92,463483	21,129046	28,24414	350,767495	275,229456	191,718147	504,876903	433,705834	319,763941
mmu-miR-702-5p	MIMAT0022931	164,881946	26,012542	13,43759	82,032191	10,697754	14,300166	191,730193	116,192154	80,936629	240,534341	169,363272	124,868662
mmu-miR-7662-3p	MIMAT0029831	155,804745	16,935341	8,748479	77,431479	6,097042	8,150188	174,584625	99,046586	68,993443	146,454477	75,283408	55,505177
mmu-miR-7669-3p	MIMAT0029845	286,31756	147,448156	76,168947	114,184222	42,849785	57,279222	770,126895	694,588856	483,833709	745,686631	674,515561	497,308861
mmu-miR-135a-1-3p	MIMAT0004531	174,333134	35,46373	18,319897	82,161743	10,827306	14,473344	246,568509	171,03047	119,135667	226,092757	154,921688	114,221127
mmu-miR-7053-5p	MIMAT0028010	784,937576	646,068172	333,746678	213,191011	141,856574	189,626021	2337,187839	2261,6498	1575,410263	2959,325543	2888,154474	2129,387212
mmu-miR-6394	MIMAT0025144	252,39754	113,528136	58,646502	99,232902	27,898465	37,293125	494,763492	419,225452	292,022257	592,394787	521,223718	384,289389
mmu-miR-7237-3p	MIMAT0028443	138,008566			76,390561	5,056125	6,758748	106,954109	31,41607	21,883671	105,326319	34,15525	25,182086
mmu-miR-3109-3p	MIMAT0014950	155,250709	16,381305	8,462274	75,961668			91,083587	15,545547	10,82865	135,793397	64,622328	47,644944
mmu-miR-6909-5p	MIMAT0027718	982,839509	843,970105	435,979098	331,203436	259,868999	347,378502	3678,3274	3602,789361	2509,615474	3937,587028	3866,415959	2850,64278
mmu-miR-134-3p	MIMAT0016985	191,920799	53,051396	27,405354	91,316475	19,982038	26,710884	374,116513	298,578474	207,982505	289,064714	217,893645	160,649282
mmu-miR-7011-5p	MIMAT0027926	250,517061	111,647657	57,675082	107,144636	35,810199	47,869094	532,488133	456,950093	318,300325	609,832588	538,661519	397,145983
mmu-miR-7045-5p	MIMAT0027994	1295,956411	1157,087007	597,729406	579,742969	508,408532	679,612401	5629,708501	5554,170462	3868,900105	6567,504596	6496,333526	4789,636309
mmu-miR-6973a-5p	MIMAT0027848	170,659594	31,79019	16,422215	83,074952	11,740515	15,694071	207,477069	131,939029	91,90552	237,832873	166,661803	122,876915
mmu-miR-181a-5p	MIMAT0000210	301,226006	162,356602	83,87037	121,385628	50,051191	66,905663	830,977968	755,439929	526,221087	722,748304	651,577234	480,396822
mmu-miR-6990-5p	MIMAT0027882	219,827072	80,957668	41,82121	114,110543	42,776106	57,180733	530,423362	454,885322	316,862056	537,809818	466,638749	344,04482
mmu-miR-3092-3p	MIMAT0014906	174,070758	35,201354	18,184358	74,419779			148,775832	73,237792	51,015665	163,393436	92,222367	67,993984
mmu-miR-7069-5p	MIMAT0028044	287,295322	148,425918	76,67404	141,07803	69,743593	93,229377	865,361688	789,823648	550,17195	815,516319	744,34525	548,793103
mmu-miR-3087-5p	MIMAT0014895	295,043228	156,173824	80,676463	101,9009	30,566464	40,859558	577,106243	501,568203	349,380216	662,71959	591,54852	436,138671
mmu-miR-296-3p	MIMAT0004576	298,627387	159,757983	82,527972	124,561054	53,226617	71,150397	770,846575	695,308535	484,33502	741,23032	670,059251	494,023299
mmu-miR-1900	MIMAT0007870	151,941131	13,071727	6,752609	74,443341			112,516193	36,978154	25,758083	94,302066	23,130997	17,054091
mmu-miR-3061-5p	MIMAT0014828	229,670628	90,801224	46,906206	101,054895	29,720458	39,728665	474,451952	398,913912	277,873732	434,144454	362,973384	267,614108
mmu-miR-7076-5p	MIMAT0028058	404,564751	265,695347	137,253224	137,701117	66,36668	88,715307	1041,175886	965,637846	672,639846	1084,308395	1013,137326	746,968933
mmu-miR-1982-5p	MIMAT0009459	184,878107	46,008703	23,767232	88,457459	17,123022	22,889108	245,986329	170,448289	118,730134	305,070458	233,899389	172,450045
mmu-miR-291a-5p	MIMAT0000367	384,832201	245,962797	127,059759	151,811388	80,476951	107,577137	1230,092186	1154,554147	804,234348	961,179974	890,008905	656,188441
mmu-miR-290a-5p	MIMAT0000366	257,737101	118,867697	61,404819	128,850582	57,516145	76,884401	658,804716	583,266677	406,289386	682,350321	611,179252	450,612076
mmu-miR-7063-5p	MIMAT0028030	636,227225	497,357821	256,925705	187,158801	115,824364	154,827602	2024,728456	1949,190416	1357,758653	1681,362426	1610,191357	1187,166723
mmu-miR-486b-3p	MIMAT0014944	242,133482	103,264078	53,344282	108,461395	37,126958	49,629264	496,667284	421,129245	293,348393	534,716732	463,545663	341,76434
mmu-miR-200b-3p	MIMAT0000233	161,346351	22,476947	11,611168	78,520482	7,186045	9,605908	199,337392	123,799352	86,235619	131,694849	60,52378	44,623154
mmu-miR-129b-5p	MIMAT0029862	157,579672	18,710268	9,665373	73,3469			121,732558	46,194519	32,177979	108,053626	36,882557	27,192882
mmu-miR-181b-5p	MIMAT0000673	173,820804	34,951401	18,055237	81,661384	10,326947	13,804492	220,318244	144,780205	100,850371	199,358629	128,18756	94,510509
mmu-miR-1894-3p	MIMAT0007878	613,857509	474,988105	245,36993	201,445953	130,111516	173,925878	1802,379546	1726,841507	1202,875809	1918,280284	1847,109215	1361,842234
mmu-miR-1188-5p	MIMAT0005843	165,033979	26,164575	13,516128	75,863465			127,077368	51,539328	35,901043	134,126674	62,955605	46,416098
mmu-miR-3097-5p	MIMAT0014915	217,55488	78,685476	40,647438	105,272139	33,937702	45,366042	485,304849	409,766809	285,433597	391,75308	320,58201	236,359668
mmu-miR-291b-5p	MIMAT0003189	343,096591	204,227187	105,499927	150,020744	78,686307	105,183503	1016,64722	941,109181	655,553774	910,888338	839,717269	619,109272
mmu-miR-7655-3p	MIMAT0029817	193,098602	54,229198	28,013785	80,205486	8,871049	11,858328	238,016388	162,478349	113,178467	236,762023	165,590954	122,087396
mmu-miR-6971-5p	MIMAT0027844	305,630272	166,760868	86,145531	107,216265	35,881828	47,964843	550,370586	474,832547	330,756807	692,199646	621,028577	457,873816
mmu-miR-128-2-5p	MIMAT0017069	478,181302	339,311898	175,282151	176,659117	105,32468	140,792206	1297,978683	1222,440643	851,522431	1406,144544	1334,973475	984,253256
mmu-miR-7036b-3p	MIMAT0029809	279,181514	140,31211	72,482599	117,185472	45,851035	61,291127	611,053213	535,515173	373,02685	594,073587	522,902517	385,52714

mmu-miR-7003-5p	MIMAT0027910	795,872331	657,002927	339,395367	243,651951	172,317514	230,344521	2152,900741	2077,362701	1447,040351	2479,921024	2408,749955	1775,930407
mmu-miR-292a-5p	MIMAT0000369	161,704409	22,835006	11,796135	83,830541	12,496104	16,7041	180,85528	105,31724	73,361429	190,101789	118,93072	87,685598
mmu-miR-6924-3p	MIMAT0027749	141,632921			77,332476	5,998039	8,017846	110,237999	34,699959	24,171148	99,239052	28,067983	20,694047
mmu-miR-6931-5p	MIMAT0027762	4535,463675	4396,594271	2271,197989	1447,336003	1376,001567	1839,362775	15535,87756	15460,33952	10769,29662	16660,13179	16588,96073	12230,75883
mmu-miR-6239	MIMAT0024860	569,288649	430,419245	222,346495	212,442409	141,107973	188,625332	1707,410343	1631,872303	1136,722571	1630,337264	1559,166194	1149,546738
mmu-miR-7212-5p	MIMAT0028392	181,014228	42,144824	21,771224	78,982936	7,648499	10,224091	175,62209	100,08405	69,716116	217,640399	146,46933	107,989348
mmu-miR-6905-5p	MIMAT0027710	154,802846	15,933442	8,230917	74,999142			97,208896	21,670857	15,095392	112,529003	41,357934	30,492502
mmu-miR-3090-5p	MIMAT0014901	1080,744868	941,875464	486,555167	396,246263	324,911826	434,324155	3515,443981	3439,905941	2396,154844	3706,458681	3635,287612	2680,235778
mmu-miR-6908-5p	MIMAT0027716	600,337883	461,468479	238,385946	246,159911	174,825474	233,697022	1938,015333	1862,477294	1297,3564	1746,436477	1675,265407	1235,144715
mmu-miR-582-3p	MIMAT0005292	210,337569	71,468165	36,919111	86,520769	15,186332	20,300249	271,436107	195,898067	136,457831	298,591874	227,420805	167,673495
mmu-miR-1843b-5p	MIMAT0019345	160,905411	22,036007	11,383387	71,318195			123,399483	47,861443	33,339118	107,343294	36,172225	26,669167
mmu-miR-5626-5p	MIMAT0022381	158,419075	19,549671	10,098993	71,64321			114,729606	39,191566	27,299892	105,852577	34,681508	25,570086
mmu-miR-711	MIMAT0003501	3667,744777	3528,875373	1822,95071	1311,58772	1240,253283	1657,901979	14697,35269	14621,81465	10185,20059	10868,82003	10797,64897	7960,923084
mmu-miR-326-5p	MIMAT0017027	1419,694745	1280,825341	661,650305	476,344862	405,010425	541,395531	4034,016353	3958,478313	2757,37975	4679,260729	4608,089659	3397,466195
mmu-miR-6769b-5p	MIMAT0028040	1102,157027	963,287623	497,616286	396,825176	325,490739	435,098014	3278,527069	3202,989029	2231,124283	3494,488344	3423,317275	2523,95365
mmu-miR-7008-5p	MIMAT0027920	269,033106	130,163702	67,240123	108,53163	37,197193	49,72315	479,520522	403,982483	281,404375	488,220547	417,049477	307,483492
mmu-miR-6997-3p	MIMAT0027897	155,383654	16,51425	8,530951	72,830335			111,032866	35,494826	24,724833	95,684589	24,51352	18,073401
mmu-miR-183-3p	MIMAT0004539	239,396902	100,527498	51,930617	98,227759	26,893322	35,949505	389,221148	313,683108	218,504027	372,520386	301,349317	222,17973
mmu-miR-294-5p	MIMAT0004574	195,022718	56,153314	29,007747	92,742706	21,408269	28,617389	293,04963	217,51159	151,513285	257,249746	186,078677	137,192646
mmu-miR-181d-5p	MIMAT0004324	156,517884	17,64848	9,116873	76,630548	5,296111	7,079548	130,27196	54,733921	38,126319	129,48252	58,31145	42,992041
mmu-miR-770-3p	MIMAT0003891	665,163911	526,294507	271,873853	277,242362	205,907925	275,246324	1925,045919	1849,50788	1288,322222	2020,49395	1949,32288	1437,202633
mmu-miR-327	MIMAT0004867	162,213671	23,344267	12,05921	75,175899			113,581825	38,043785	26,500376	116,378159	45,207089	33,330419
mmu-miR-6418-5p	MIMAT0025173	213,475564	74,60616	38,54014	97,158855	25,824418	34,520654	309,506466	233,968427	162,976718	339,76691	268,59584	198,031149
mmu-miR-7068-5p	MIMAT0028042	162,124637	23,255233	12,013216	79,211592	7,877155	10,529745	136,880869	61,34283	42,729924	163,772802	92,601733	68,273684
mmu-miR-1963	MIMAT0009436	158,022694	19,15329	9,89423	74,066797			115,987603	40,449564	28,176183	98,783024	27,611955	20,357825
mmu-miR-7052-5p	MIMAT0028008	1866,206509	1727,337105	892,309892	697,626705	626,292268	837,192857	6260,594741	6185,056702	4308,360121	5642,072674	5570,901605	4107,33108
mmu-miR-8099	MIMAT0031401	249,231256	110,361852	57,010859	109,188729	37,854292	50,601523	447,074987	371,536948	258,803604	429,278323	358,107254	264,026392
mmu-miR-6968-5p	MIMAT0027838	3522,595749	3383,726345	1747,96945	894,791021	823,456584	1100,751208	10224,43811	10148,90007	7069,477036	8950,078223	8878,907154	6546,267354
mmu-miR-211-3p	MIMAT0017059	1207,359509	1068,490105	551,961911	481,991266	410,656829	548,943332	3791,937667	3716,399627	2588,753623	3686,312759	3615,14169	2665,382532
mmu-miR-1190	MIMAT0005847	149,060014			76,558468	5,224032	6,983196	105,393626	29,855587	20,796676	87,229181	16,058111	11,839373
mmu-miR-7062-5p	MIMAT0028028	149,38518			79,659171	8,324734	11,128045	118,612882	43,074842	30,004888	100,65711	29,48604	21,739556
mmu-miR-3072-5p	MIMAT0014852	1278,473086	1139,603682	588,697849	512,631157	441,29672	589,901043	3662,313106	3586,775066	2498,460305	4106,670194	4035,499124	2975,304924
mmu-miR-7085-5p	MIMAT0028076	421,003517	282,134114	145,745182	167,931865	96,597428	129,126098	983,738194	908,200155	632,630147	937,939044	866,767975	639,053298
mmu-miR-8113	MIMAT0031419	12458,55933	12319,68992	6364,120328	4483,584796	4412,250359	5898,052198	39138	39062,46196	27209,96127	37577	37505,82893	27652,41029
mmu-miR-194-1-3p	MIMAT0016999	153,10109	14,231686	7,351822	72,109208			103,569364	28,031324	19,525939	88,636019	17,46495	12,876611
mmu-miR-346-5p	MIMAT0000597	202,102697	63,233293	32,665131	85,17043	13,835993	18,495191	244,477664	168,939624	117,679235	214,852002	143,680933	105,93351
mmu-miR-665-5p	MIMAT0017238	207,483342	68,613938	35,444671	88,944868	17,610432	23,540651	252,252592	176,714552	123,095061	253,468396	182,297327	134,404721
mmu-miR-429-3p	MIMAT0001537	157,007653	18,138249	9,369879	67,052516			106,625893	31,087854	21,655043	96,950054	25,778985	19,006407
mmu-miR-696	MIMAT0003483	176,634213	37,764809	19,508591	77,679352	6,344915	8,481532	157,552556	82,014517	57,129318	158,284453	87,113384	64,227217
mmu-miR-1843a-5p	MIMAT0014805	152,818575	13,949171	7,205879	77,902701	6,568265	8,780093	126,789088	51,251048	35,700234	116,614692	45,443622	33,50481
mmu-miR-6943-5p	MIMAT0027786	156,641411	17,772007	9,180685	80,147831	8,813394	11,781258	131,371588	55,833549	38,892292	141,384191	70,213122	51,766941
mmu-miR-6988-5p	MIMAT0027878	2118,942108	1980,072704	1022,868353	690,248293	618,913856	827,329805	5105,461686	5029,923647	3503,722519	6149,925054	6078,753985	4481,76201
mmu-miR-370-3p	MIMAT0001095	1147,464647	1008,595244	521,021351	482,029579	410,695142	548,994546	3587,567312	3512,029272	2446,394203	2898,193352	2827,022283	2084,315486
mmu-miR-6396	MIMAT0025147	207,057235	68,187831	35,224552	84,355343	13,020906	17,405627	232,798646	157,260607	109,543915	224,291218	153,120149	112,892884
mmu-miR-30c-1-3p	MIMAT0004616	211,166351	72,296947	37,347244	84,151212	12,816775	17,132756	248,994398	173,456359	120,825482	219,202191	148,031122	109,14083
mmu-miR-149-3p	MIMAT0016990	5790,713028	5651,843624	2919,636219	1633,29412	1561,959683	2087,941298	14362,69239	14287,15435	9952,084355	14612,57986	14541,4088	10721,13359
mmu-miR-7048-5p	MIMAT0028000	438,521866	299,652462	154,794832	177,898059	106,563622	142,448355	950,008942	874,470902	609,135169	896,926309	825,75524	608,815305
mmu-miR-3104-5p	MIMAT0014939	689,480967	550,611563	284,435588	271,143235	199,808798	267,093348	1667,911096	1592,373057	1109,208356	1608,934277	1537,763208	1133,76668
mmu-miR-6982-5p	MIMAT0027866	333,516687	194,647283	100,551129	121,561504	50,227067	67,140765	524,325038	448,786999	312,614112	569,078034	497,906964	367,098343
mmu-miR-3070-2-3p	MIMAT0014849	172,985988	34,116584	17,623986	79,060159	7,725722	10,327318	156,042773	80,504733	56,07764	146,985702	75,814633	55,89684
mmu-miR-3547-5p	MIMAT0027832	4373,650354	4234,78095	2187,608268	1298,423396	1227,088959	1640,304639	10908,25666	10832,71862	7545,808413	10553,89865	10482,72758	7728,736898
mmu-miR-125a-3p	MIMAT0004528	251,408186	112,538783	58,13542	132,982715	61,648278	82,408008	455,020526	379,482486	264,338274	465,430064	394,258994	290,680456
mmu-miR-615-5p	MIMAT0004837	207,969719	69,100315	35,695924	100,583323	29,248886	39,098293	307,407417	231,869377	161,514571	250,056095	178,885026	131,88889
mmu-miR-712-5p	MIMAT0003502	241,374291	102,504887	52,952099	109,620715	38,286278	51,178979	386,693222	311,155183	216,743135	331,052294	259,881224	191,60601
mmu-miR-6917-3p	MIMAT0027735	153,419067	14,549663	7,516083	72,401974			92,702248	17,164208	11,95617	94,702303	23,531234	17,349179
mmu-miR-1981-5p	MIMAT0009458	154,516616	15,647212	8,083056	76,016661			98,044425	22,506386	15,677401	92,594954	21,423885	15,795466
mmu-miR-6926-5p	MIMAT0027752	169,518482	30,649078	15,832738	78,685827	7,35139	9,826932	134,441759	58,903719	41,030899	150,182691	79,011622	58,253926
mmu-miR-546	MIMAT0003166	212,26453	73,395126	37,914543	94,360936	23,026499	30,780551	235,573994	160,035955	111,477155	279,066969	207,8959	153,278114
mmu-miR-664-5p	MIMAT0017353	186,920211	48,050807	24,822144	86,56523	15,230793	20,359682	192,954343	117,416303	81,789342	194,352027	123,180958	90,819227
mmu-miR-6356	MIMAT0025099	154,773759	15,904355	8,215891	78,581949	7,247512	9,688073	131,740628	56,202588	39,149357	110,55642	39,38535	29,038149
mmu-miR-1231-3p	MIMAT0022358	213,421943	74,552539	38,512441	90,018916	18,684479	24,976378	266,755947	191,217907	133,197745	217,111097	145,940028	107,599102
mmu-miR-6938-3p	MIMAT0027777	159,683092	20,813688	10,75196	75,325038			107,338807	31,800767	22,151641	96,434288	25,263219	18,626142
mmu-miR-7010-5p	MIMAT0027924	157,911737	19,042333	9,836912	78,045366	6,710929	8,970798	127,552381	52,014342	36,231926	118,608259	47,437189	34,974634
mmu-miR-7019-5p	MIMAT0027942	204,994211	66,124808	34,158833	87,999348	16,664911	22,276731	201,006237	125,468197	87,398096	242,109964	170,938894	126,030342
mmu-miR-743b-5p	MIMAT0004839	139,101728			76,700702	5,366265	7,173326	97,488849	21,950809	15,2904	87,139659	15,968589	11,77337
mmu-miR-7242-5p	MIMAT0028452	177,43511	38,565706	19,922319	79,032704	7,698267	10,290617	138,070506	62,532467	43,558596	166,257093	95,086023	70,105309
mmu-miR-1901	MIMAT0007880	194,956569	56,087165	28,973575	87,033872	15,699435	20,986136	238,391835	162,853796	113,439995	171,448495	100,277426	73,932842
mmu-miR-7654-3p	MIMAT0029815	187,267324	48,39792	25,001456	81,751187	10,41675	13,924535	178,627661	103,089622	71,809724	171,635726	100,464657	74,070884
mmu-miR-92b-5p	MIMAT0017278	650,600439	511,731035	264,350637	300,83068	229,496244	306,777883	1536,726581	1461,188542	1017,828412	1497,704345	1426,533276	1051,758741
mmu-miR-200a-5p	MIMAT0004619	153,572766	14,703362	7,59548	73,070771			79,368753			107,995473	36,824404	27,150007
mmu-miR-7653-3p	MIMAT0029813	188,299337	49,429933	25,534575	88,176455	16,842018	22,513478	194,884334	119,346294	83,133727	189,753115	118,582046	87,428527
mmu-miR-7115-3p	MIMAT0028128	195,511906	56,642502	29,260452	91,118369	19,783932	26,446066	232,76444	157,226401	109,520088	188,79824	117,62717	86,724514
mmu-miR-7020-5p	MIMAT0027944	673,648728	534,779324	276,256951	248,403647	177,06921	236,696324	1285,522944	1209,984905	842,846066	1359,393704	1288,222635	949,784656
mmu-miR-351-5p	MIMAT0000609	424,161155	285,291752	147,376358	188,767463	117,433026	156,977973	911,818451	836,280412	582,532602	692,474758	621,303688	458,076651
mmu-miR-3060-5p	MIMAT0014826	152,466379	13,596975	7,023942	71,35655			84,584072	9,046032	6,301246	95,038849	23,86778	17,597309
mmu-miR-7016-5p	MIMAT0027936	414,718416	275,849012	142,498417	157,518828	86,184391	115,206527	587,519479	511,981439	356,633823	774,695945	703,524876	518,696935
mmu-miR-7031-5p	MIMAT0027966	300,178015	161,308611	83,328997	118,864801	47,530364	63,535961	415,372377	339,834337	236,720337	425,762726	354,591657	261,434403
mmu-miR-5110	MIMAT0020618	306,111873	167,24247	86,394317	123,187972	51,853535	69,314937	443,921992	368,383953	256,607305	432,083482	360,912413	266,094589
mmu-miR-8117	MIMAT0031423	3877,465722	3738,596318	1931,28861	1222,298778	1150,964341	1538,545461	7884,199829	7808,661789	5439,32395	8449,004863	8377,833793	6176,834481
mmu-miR-7221-3p	MIMAT0028411	330,650909	191,781505	99,070722	128,245095	56,910658	76,075019	392,136159	316,59812	220,534553	566,517883	495,346813	365,210787
mmu-miR-6973b-5p	MIMAT0027908	176,283302	37,413898	19,327317	85,341881	14,007444	18,724377	152,86027	77,32223	53,860785	170,358798	99,187728	73,129427
mmu-miR-6985-5p	MIMAT0027872	166,804479	27,935075	14,430735	84,844084	13,509647	18,058949	133,268505	57,730466	40,213639	163,180374	92,009305	67,836897
mmu-miR-490-5p	MIMAT0017261	132,668948			76,669784	5,335347	7,131997	92,825121	17,287082	12,041761	87,008896	15,837827	11,676961
mmu-miR-133a-3p	MIMAT0000145	161,36232	22,492916	11,619418	74,667571			106,343972	30,805933	21,458664	94,339066	23,167997	17,08137
mmu-miR-7050-5p	MIMAT0028004	191,391497	52,522093	27,131927	86,312132	14,977695	20,021354	182,234405	106,696366	74,322094	180,966798	109,795729	80,950525
mmu-miR-3960	MIMAT0019336	8058,235209	7919,365805	4090,995571	3552,12282	3480,788383	4652,925355	19691,92302	19616,38498	13664,29684	20044,64495	19973,47388	14726,10286
mmu-miR-1249-3p	MIMAT0010560	362,921284	224,05188	115,740992	139,826426	68,491989	91,556302	538,282938	462,744898	322,336845	546,576486	475,405417	350,508335
mmu-miR-7076-3p	MIMAT0028059	165,387394	26,51799	13,698695	74,746125			107,219599	31,681559	22,068604	101,480434	30,309364	22,346579
mmu-miR-7032-5p	MIMAT0027968	169,930542	31,061138	16,0456	84,257221	12,922784	17,274463	156,874685	81,336645	56,65713	140,852346	69,681277	51,374821
mmu-miR-432	MIMAT0012771	152,964057	14,094653	7,281033	80,559718	9,225281	12,331846	116,677983	41,139944	28,657085	118,404166	47,233097	34,82416
mmu-miR-3473f	MIMAT0031390	209,936843	71,067439	36,712104	89,865884	18,531447	24,771813	200,070354	124,532315	86,746183	221,046505	149,875436	110,500612
mmu-miR-331-3p	MIMAT0000571	157,587782	18,718378	9,669562	74,628335			102,559598	27,021558	18,82256	87,693333	16,522264	12,181584
mmu-miR-3099-3p	MIMAT0014816	153,653592	14,784188	7,637234	70,29199			95,992803	20,454763	14,248291	84,993437	13,822368	10,190997
mmu-miR-3572-5p	MIMAT0022986	417,482615	278,613211	143,92635	162,717734	91,383298	122,156137	576,272796	500,734756	348,799657	748,232243	677,061174	499,185698
mmu-miR-290b-3p	MIMAT0029901	228,93404	90,064636	46,525699	97,241302	25,906865	34,630865	225,084749	149,546709	104,170602	279,35682	208,18575	153,491816
mmu-miR-181c-3p	MIMAT0017068	154,193573	15,324169	7,916178	72,190324			94,807041	19,269001	13,422318	86,608603	15,437534	11,381831

mmu-miR-1930-3p	MIMAT0017340	154,948991	16,079587	8,306413	80,187067	8,85263	11,833707	116,928331	41,390291	28,831471	117,404842	46,233773	34,087375
mmu-miR-5132-5p	MIMAT0020643	1167,106224	1028,23682	531,167821	609,898214	538,563777	719,922264	2762,461663	2686,923623	1871,645669	2820,067362	2748,896292	2026,714521
mmu-miR-8104	MIMAT0031408	212,18329	73,313886	37,872576	84,884652	13,550215	18,113177	187,095358	111,557319	77,708116	202,369854	131,198784	96,730634
mmu-miR-6993-5p	MIMAT0027888	180,112415	41,243011	21,305365	85,410416	14,07598	18,815991	155,491698	79,953658	55,693774	165,002	93,830931	69,179951
mmu-miR-3067-5p	MIMAT0014840	160,217402	21,347999	11,027975	80,975868	9,641431	12,888131	125,970459	50,432419	35,129997	123,710816	52,539746	38,736662
mmu-miR-7221-5p	MIMAT0028410	156,803509	17,934105	9,264422	79,734263	8,399826	11,228423	120,357885	44,819846	31,220415	114,361967	43,190897	31,843915
mmu-miR-714	MIMAT0003505	170,975325	32,105921	16,585315	81,291827	9,95739	13,310488	153,33136	77,79332	54,188935	122,44602	51,274951	37,80415
mmu-miR-141-3p	MIMAT0000153	151,445434	12,57603	6,496541	78,665728	7,331291	9,800064	119,131719	43,59368	30,366297	97,795884	26,624814	19,630023
mmu-miR-7018-5p	MIMAT0027940	252,856282	113,986878	58,883479	115,030739	43,696303	58,4108	308,59854	233,060501	162,344278	339,049193	267,878124	197,501988
mmu-miR-6963-5p	MIMAT0027826	213,160414	74,29101	38,37734	91,149818	19,815381	26,488106	206,88559	131,34755	91,49351	215,608917	144,437848	106,491571
mmu-miR-465a-5p	MIMAT0002106	139,116147			76,285802	4,951365	6,618711	104,363037	28,824997	20,078792	71,929471		
mmu-miR-6919-5p	MIMAT0027738	167,890354	29,02095	14,991677	71,253634			110,202502	34,664463	24,146422	100,079295	28,908225	21,313543
mmu-miR-6904-3p	MIMAT0027709	166,372103	27,5027	14,207378	83,601086	12,266649	16,397377	148,444725	72,906685	50,785024	128,040253	56,869184	41,928683
mmu-miR-6935-5p	MIMAT0027770	1203,322597	1064,453193	549,876519	330,565269	259,230832	346,525436	1986,370704	1910,832664	1331,039576	1941,624968	1870,453899	1379,053873
mmu-miR-7235-5p	MIMAT0028438	447,039826	308,170422	159,19505	201,597899	130,263462	174,12899	641,711803	566,173763	394,382878	901,321861	830,150791	612,056071
mmu-miR-3067-3p	MIMAT0014841	207,62612	68,756716	35,518428	98,217309	26,882873	35,935537	237,547734	162,009695	112,852014	210,509586	139,338517	102,731921
mmu-miR-6942-3p	MIMAT0027785	156,885968	18,016564	9,307018	73,834679			99,011093	23,473054	16,350759	86,970142	15,799073	11,648388
mmu-miR-673-3p	MIMAT0004824	155,136221	16,266817	8,403132	76,031454			97,413723	21,875683	15,238069	84,543898	13,372829	9,859559
mmu-miR-7017-5p	MIMAT0027938	157,339088	18,469684	9,541092	76,584161	5,249724	7,017541	112,155739	36,6177	25,506999	103,470844	32,299774	23,814075
mmu-miR-3062-3p	MIMAT0014831	158,393961	19,524557	10,086019	73,956444			97,30139	21,763351	15,159821	91,32367	20,152601	14,85817
mmu-miR-467f	MIMAT0005846	144,910121			78,306701	6,972264	9,320137	101,01332	25,47528	17,745461	84,593933	13,422864	9,896449
mmu-miR-8102	MIMAT0031406	369,441756	230,572352	119,109345	136,054181	64,719744	86,513774	486,832468	411,294429	286,497699	509,334428	438,163359	323,050398
mmu-miR-6925-3p	MIMAT0027751	171,823284	32,95388	17,023355	79,758624	8,424188	11,260988	135,863195	60,325155	42,021036	127,82471	56,653641	41,769766
mmu-miR-1934-5p	MIMAT0009398	174,308077	35,438674	18,306953	80,501632	9,167196	12,2542	136,160306	60,622266	42,227997	136,685936	65,514867	48,302998
mmu-miR-7073-3p	MIMAT0028053	167,750452	28,881048	14,919407	77,9437	6,609263	8,834897	123,569329	48,03129	33,457429	121,137003	49,965934	36,839034
mmu-miR-708-5p	MIMAT0004828	149,455732			80,167049	8,832612	11,806948	101,655756	26,117717	18,192967	93,860419	22,68935	16,728472
mmu-miR-3572-3p	MIMAT0020636	163,352885	24,483481	12,647706	77,084683	5,750247	7,686612	125,717368	50,179329	34,953701	105,109192	33,938123	25,022001
mmu-miR-6899-5p	MIMAT0027698	344,917164	206,04776	106,440401	135,981834	64,647397	86,417064	516,76962	441,231581	307,351191	419,690636	348,519567	256,957554
mmu-miR-6937-3p	MIMAT0027775	181,817637	42,948233	22,18625	86,133325	14,798888	19,782335	169,189583	93,651544	65,235388	148,799999	77,62893	57,234491
mmu-miR-7040-5p	MIMAT0027984	373,61775	234,748346	121,266585	174,262842	102,928405	137,588998	587,921898	512,383859	356,914138	603,800993	532,629924	392,698991
mmu-miR-5116	MIMAT0020624	413,263082	274,393678	141,746618	167,907872	96,573435	129,094026	674,31272	598,774681	417,091885	565,999743	494,828673	364,828772
mmu-miR-3475-3p	MIMAT0015219	153,410908	14,541505	7,511868	74,428466			92,703748	17,165709	11,957215	84,099213	12,928144	9,531701
mmu-miR-7058-5p	MIMAT0028020	266,792313	127,922909	66,082571	114,070857	42,73642	57,127682	321,649144	246,111104	171,435012	313,911778	242,740709	178,9686
mmu-miR-6918-5p	MIMAT0027736	154,015182	15,145778	7,824024	75,537569			90,395887	14,857848	10,349615	87,208197	16,037128	11,823902
mmu-miR-363-3p	MIMAT0000708	147,583039			80,148281	8,813844	11,78186	98,401472	22,863433	15,926111	94,728078	23,557009	17,368182
mmu-miR-3081-5p	MIMAT0014870	188,002661	49,133257	25,381317	87,096301	15,761865	21,069589	167,872424	92,334384	64,317887	161,353893	90,182824	66,490263
mmu-miR-3475-5p	MIMAT0026642	223,035602	84,166198	43,478677	98,713775	27,379338	36,599185	236,727653	161,189614	112,280766	224,11369	152,942621	112,761995
mmu-miR-466i-5p	MIMAT0017325	202,812306	63,942902	33,031701	95,698613	24,364176	32,568683	199,340024	123,801985	86,237453	202,984963	131,813894	97,184144
mmu-miR-675-5p	MIMAT0003725	189,025296	50,155892	25,909592	96,807185	25,472748	34,05056	210,924445	135,386405	94,306878	170,395247	99,224177	73,1563
mmu-miR-3104-3p	MIMAT0014940	235,605093	96,735689	49,971839	106,099725	34,765288	46,472314	261,007843	185,469804	129,193756	258,697594	187,526525	138,26012
mmu-miR-3971	MIMAT0019356	208,447323	69,577919	35,942646	97,314181	25,979744	34,728285	216,571063	141,033023	98,240175	203,726764	132,555695	97,731061
mmu-miR-133b-3p	MIMAT0000769	151,735469	12,866066	6,646368	81,972405	10,637968	14,220248	119,239919	43,701879	30,441667	108,227032	37,055963	27,320732
mmu-miR-378d	MIMAT0025167	237,182328	98,312924	50,786609	112,00144	40,667003	54,361401	297,847632	222,309593	154,855457	252,687102	181,516033	133,828686
mmu-miR-361-3p	MIMAT0017075	201,12719	62,257786	32,161202	90,192579	18,858142	25,208521	182,511148	106,973108	74,514866	182,94666	111,775591	82,410243
mmu-miR-7052-3p	MIMAT0028009	313,200584	174,33118	90,056212	121,88615	50,551713	67,574734	398,303163	322,765124	224,830338	347,661365	276,490296	203,851596
mmu-miR-1906	MIMAT0007872	216,271412	77,402008	39,984423	99,964211	28,629774	38,270698	234,268752	158,730713	110,567955	208,935824	137,764755	101,571612
mmu-miR-3091-5p	MIMAT0014903	155,057493	16,188089	8,362463	75,97558			93,424522	17,886482	12,459289	84,903915	13,732846	10,124994
mmu-miR-877-5p	MIMAT0004861	185,182951	46,313547	23,924708	85,269883	13,935446	18,628134	144,28195	68,743911	47,885337	161,658181	90,487112	66,71461
mmu-miR-3544-3p	MIMAT0022354	150,929556	12,060152	6,230048	75,605592			89,898083	14,360043	10,002857	80,204871	9,033802	6,660468
mmu-miR-615-3p	MIMAT0003783	168,53227	29,662866	15,323279	82,473645	11,139209	14,890278	136,833122	61,295082	42,696664	122,22816	51,057091	37,643525
mmu-miR-7020-3p	MIMAT0027945	167,173855	28,304451	14,621548	76,490814	5,156377	6,89276	123,507893	47,969853	33,414633	103,068916	31,897846	23,51774
mmu-miR-6367	MIMAT0025111	151,333458	12,464054	6,438696	78,22116	6,886723	9,205791	109,158678	33,620639	23,419319	95,538424	24,367354	17,965636
mmu-miR-7665-3p	MIMAT0029837	164,248802	25,379398	13,11052	84,576561	13,242124	17,701339	142,948617	67,410577	46,956569	117,718883	46,547814	34,318912
mmu-miR-3070-5p	MIMAT0014846	239,373048	100,503644	51,918294	102,828021	31,493585	42,098882	263,479449	187,94141	130,915417	229,941743	158,770674	117,05892
mmu-miR-205-3p	MIMAT0017003	156,821692	17,952288	9,273815	72,5682			90,841295	15,303256	10,659876	89,763506	18,592437	13,707888
mmu-miR-664-3p	MIMAT0012774	168,406853	29,53745	15,258491	79,30582	7,971383	10,655704	132,876674	57,338635	39,940699	109,069761	37,898692	27,942062
mmu-miR-6974-5p	MIMAT0027850	316,851347	177,981943	91,942127	108,317849	36,983413	49,43738	298,27838	222,740341	155,155506	363,01094	291,839871	215,168577
mmu-miR-6953-5p	MIMAT0027806	517,721855	378,852451	195,708058	174,579682	103,245245	138,012532	675,372251	599,834211	417,829928	689,827615	618,656546	456,124958
mmu-miR-5106	MIMAT0020613	152,723289	13,853885	7,156657	67,579612			88,493546	12,955507	9,024491	84,185516	13,014446	9,59533
mmu-miR-6981-5p	MIMAT0027864	742,973603	604,104199	312,068878	293,056774	221,722337	296,386155	1034,920446	959,382406	668,282459	1309,385937	1238,214867	912,914779
mmu-miR-7085-3p	MIMAT0028077	183,30651	44,437106	22,955374	81,230278	9,895841	13,228213	148,563871	73,025832	50,868019	129,174116	58,003047	42,764661
mmu-miR-296-5p	MIMAT0000374	680,22098	541,351577	279,652052	317,033829	245,699392	328,437356	1208,536156	1132,998116	789,218941	1129,575621	1058,404552	780,343689
mmu-miR-3473g	MIMAT0031427	380,619583	241,750179	124,8836	136,946491	65,612054	87,706564	408,010089	332,472049	231,591946	495,107498	423,936429	312,561124
mmu-miR-3072-3p	MIMAT0014853	348,460902	209,591498	108,271029	133,761866	62,427429	83,449533	450,959347	375,421308	261,509355	381,891916	310,720847	229,0892
mmu-miR-3070-3p	MIMAT0014847	185,971278	47,101874	24,331943	94,132281	22,797844	30,474897	177,521946	101,983907	71,03951	164,824478	93,653409	69,049067
mmu-miR-7045-3p	MIMAT0027995	532,98753	394,118126	203,594018	178,527108	107,192671	143,289233	703,745192	628,207153	437,593829	670,798908	599,627839	442,095415
mmu-miR-18a-3p	MIMAT0004626	153,621346	14,751942	7,620576	82,936631	11,602195	15,509172	123,539208	48,001168	33,436447	104,795207	33,624138	24,790506
mmu-miR-7070-5p	MIMAT0028046	296,892672	158,023268	81,631851	106,283838	34,949401	46,718426	276,102725	200,564686	139,708484	319,826315	248,655246	183,329287
mmu-miR-6982-3p	MIMAT0027867	221,701647	82,832243	42,78958	82,994636	11,660199	15,586709	163,886629	88,34859	61,54148	186,956363	115,785293	85,366529
mmu-miR-292b-3p	MIMAT0029865	227,31155	88,442146	45,687551	105,655158	34,320721	45,878041	236,415401	160,877362	112,063259	231,704421	160,533352	118,358512
mmu-miR-7687-5p	MIMAT0029902	943,074116	804,204712	415,437043	544,678529	473,344092	632,740198	2128,662159	2053,12412	1430,156344	1706,286872	1635,115803	1205,543094
mmu-miR-7685-3p	MIMAT0029897	253,693803	114,824399	59,316127	109,234439	37,900003	50,662627	256,293945	180,755906	125,910169	275,038481	203,867412	150,307978
mmu-miR-6965-5p	MIMAT0027830	244,807935	105,938531	54,725855	95,676383	24,341946	32,538967	205,316779	129,778739	90,400714	245,330981	174,159912	128,405143
mmu-miR-7002-3p	MIMAT0027907	171,275152	32,405748	16,7402	77,639153	6,304717	8,427796	112,365215	36,827175	25,652915	121,756785	50,585716	37,295989
mmu-miR-293-3p	MIMAT0000371	152,007653	13,138249	6,786973	74,161906			83,245621	7,707582	5,368914	86,895904	15,724834	11,593653
mmu-miR-6954-3p	MIMAT0027809	172,869376	33,999972	17,563747	82,40431	11,069874	14,797595	134,812815	59,274775	41,289367	124,789902	53,618833	39,532254
mmu-miR-2861	MIMAT0013803	5140,771024	5001,90162	2583,888392	1606,484684	1535,150247	2052,103927	8185,714886	8110,176846	5649,352008	8052,036453	7980,865384	5884,156419
mmu-miR-6910-3p	MIMAT0027721	153,691259	14,821855	7,656692	70,847422			82,26546	6,727421	4,686158	90,623886	19,452817	14,342232
mmu-miR-3059-5p	MIMAT0014811	177,076032	38,206628	19,736826	78,393492	7,059055	9,436155	120,172849	44,634809	31,091523	126,92151	55,750441	41,103853
mmu-miR-6337	MIMAT0025080	154,930816	16,061412	8,297024	71,934663			87,704006	12,165967	8,474517	87,495498	16,324428	12,035724
mmu-miR-8109	MIMAT0031415	155,868398	16,998994	8,781361	73,64446			87,978584	12,440544	8,665781	88,798258	17,627188	12,996226
mmu-miR-5119	MIMAT0020627	181,997289	43,127885	22,279055	93,76848	22,434043	29,988588	171,512634	95,974595	66,853569	154,5387	83,36763	61,465537
mmu-miR-7031-3p	MIMAT0027967	166,992927	28,123523	14,528084	73,445103			99,110979	23,572939	16,420336	97,043698	25,872628	19,075449
mmu-miR-3100-3p	MIMAT0014920	165,875653	27,006249	13,950921	82,053971	10,719535	14,329281	125,407343	49,869303	34,737744	117,496118	46,325049	34,154671
mmu-miR-18b-3p	MIMAT0017270	168,952506	30,083102	15,540365	81,440166	10,105729	13,508779	135,457932	59,919893	41,738741	110,469322	39,298252	28,973934
mmu-miR-5135	MIMAT0020646	266,715468	127,846064	66,042874	104,776923	33,442486	44,704065	264,196028	188,657988	131,414568	258,259449	187,088379	137,937083
mmu-miR-451b	MIMAT0025178	274,483415	135,614011	70,05565	148,916066	77,581629	103,70683	393,137192	317,599152	221,231848	342,59339	271,422321	200,115065
mmu-miR-7046-3p	MIMAT0027997	244,974784	106,10538	54,812046	98,949418	27,614981	36,914179	250,72565	175,187611	122,03143	207,295785	136,124715	100,362439
mmu-miR-6346	MIMAT0025089	172,118344	33,24894	17,175778	76,225135	4,890698	6,537615	111,787167	36,249128	25,250261	114,188504	43,017435	31,716024
mmu-miR-6973a-3p	MIMAT0027849	154,020836	15,151433	7,826945	72,859203			89,642828	14,104788	9,825053	83,308217	12,137148	8,948513
mmu-miR-7082-3p	MIMAT0028071	340,154856	201,285452	103,980282	128,173978	56,839541	75,979954	357,697018	282,158979	196,545084	389,636874	318,465805	234,799426
mmu-miR-7686-5p	MIMAT0029898	237,289296	98,419892	50,841867	101,079338	29,744901	39,761339	236,979453	161,441414	112,456164	211,122935	139,951866	103,184133
mmu-miR-7050-3p	MIMAT0028005	186,560236	47,690833	24,636188	81,92485	10,590414	14,156679	141,943758	66,405718	46,256609	133,476087	62,305017	45,936431
mmu-miR-6916-5p	MIMAT0027732	179,411248	40,541844	20,943155	83,504645	12,170208	16,268461	134,792976	59,254937	41,275548	134,94909	63,778021	47,022451
mmu-miR-6956-5p	MIMAT0027812	246,253858	107,384454	55,472791	102,501675	31,167238	41,66264	210,819036	135,280996	94,233453	255,867104	184,696035	136,173248
mmu-miR-7082-5p	MIMAT0028070	257,93093	119,061526	61,504947	107,451027	36,11659	48,278659	217,402152	141,864113	98,819092	290,07763	218,906561	161,396087

mmu-miR-7040-3p	MIMAT0027985	159,610028	20,740624	10,714217	76,129144	4,794708	6,4093	106,275918	30,737879	21,411259	97,029316	25,858247	19,064846
mmu-miR-7087-5p	MIMAT0028080	199,216224	60,34682	31,174033	93,297257	21,96282	29,358683	167,598363	92,060323	64,126983	177,71966	106,548591	78,556466
mmu-miR-877-3p	MIMAT0004862	190,223106	51,353702	26,528357	78,708939	7,374502	9,857826	143,563403	68,025364	47,384814	123,047306	51,876237	38,247468
mmu-miR-7651-3p	MIMAT0029805	164,216772	25,347368	13,093974	78,117363	6,782927	9,067041	110,109492	34,571453	24,081633	109,16504	37,99397	28,012309
mmu-miR-1893	MIMAT0007879	309,382822	170,513418	88,084028	114,11753	42,783094	57,190073	310,517733	234,979693	163,681141	311,754163	240,583093	177,377826
mmu-miR-8105	MIMAT0031409	313,651855	174,782451	90,28933	121,321067	49,986631	66,819362	335,757309	260,219269	181,262416	325,491516	254,320447	187,506144
mmu-miR-7038-5p	MIMAT0027980	189,552675	50,683271	26,182025	101,592891	30,258454	40,447828	192,022717	116,484677	81,140394	173,040134	101,869065	75,10633
mmu-miR-204-3p	MIMAT0017002	313,957304	175,0879	90,447119	130,950116	59,61568	79,690943	350,864428	275,326389	191,785668	352,03012	280,859051	207,072605
mmu-miR-6980-5p	MIMAT0027862	2061,140083	1922,270679	993,008914	759,076419	687,741982	919,335436	2305,148589	2229,610549	1553,0925	4036,674176	3965,503107	2923,69805
mmu-miR-6909-3p	MIMAT0027719	194,516889	55,647485	28,746445	85,252959	13,918522	18,605511	163,689404	88,151365	61,404098	138,186412	67,015343	49,409273
mmu-miR-7651-5p	MIMAT0029804	193,217492	54,348088	28,075201	86,505895	15,171458	20,280365	152,992393	77,454353	53,952819	150,955805	79,784736	58,82393
mmu-miR-7028-3p	MIMAT0027961	288,674134	149,80473	77,386309	123,355079	52,020642	69,538316	319,536539	243,9985	169,963423	305,386986	234,215917	172,683415
mmu-miR-378a-3p	MIMAT0003151	325,02973	186,160326	96,166926	128,504136	57,169699	76,421291	336,510836	260,972797	181,787305	368,867262	297,696193	219,486344
mmu-miR-6939-3p	MIMAT0027779	168,939953	30,070549	15,533881	76,058109			101,608267	26,070228	18,159887	95,436719	24,265649	17,890651
mmu-miR-7022-5p	MIMAT0027948	208,934322	70,064918	36,19422	102,92055	31,586113	42,222568	215,065928	139,527889	97,191735	185,983773	114,812704	84,649455
mmu-miR-7072-3p	MIMAT0028051	166,70567	27,836266	14,379692	78,499501	7,165065	9,577862	112,340985	36,802946	25,636037	111,739698	40,568628	29,91056
mmu-miR-702-3p	MIMAT0003492	200,700677	61,831274	31,940874	86,754568	15,420131	20,612778	178,124753	102,586714	71,459411	139,476137	68,305068	50,360166
mmu-miR-186-3p	MIMAT0004540	152,382046	13,512642	6,980377	76,47389	5,139453	6,870137	105,502552	29,964512	20,872551	86,346175	15,175106	11,188348
mmu-miR-7081-3p	MIMAT0028069	181,507536	42,638132	22,026058	78,159612	6,825175	9,123516	122,895591	47,357551	32,988119	124,194317	53,023248	39,093139
mmu-miR-485-3p	MIMAT0003129	154,690613	15,821209	8,172939	69,043829			89,531011	13,992972	9,747164	83,586717	12,415648	9,153846
mmu-miR-1952	MIMAT0009423	159,591198	20,721794	10,70449	78,902701	7,568265	10,116838	108,022374	32,484334	22,627797	105,646681	34,475611	25,418282
mmu-miR-1224-3p	MIMAT0017231	210,519782	71,650378	37,013239	83,43361	12,099173	16,173505	158,094443	82,556404	57,506784	159,319994	88,148924	64,990704
mmu-miR-760-3p	MIMAT0003898	204,833631	65,964227	34,07588	97,856952	26,522515	35,453831	188,355778	112,817739	78,586094	181,508458	110,337389	81,349882
mmu-miR-25-5p	MIMAT0017049	199,223098	60,353694	31,177584	89,063458	17,729021	23,699175	166,171489	90,63345	63,133058	155,503892	84,332823	62,177157
mmu-miR-323-5p	MIMAT0004638	232,74744	93,878037	48,49563	95,370054	24,035618	32,129484	216,326042	140,788003	98,0695	187,756329	116,58526	85,956331
mmu-miR-7055-3p	MIMAT0028015	178,955615	40,086212	20,707784	83,730206	12,39577	16,569979	149,99559	74,45755	51,865319	115,805079	44,63401	32,907897
mmu-miR-7667-3p	MIMAT0029841	452,307632	313,438228	161,916299	178,064654	106,730217	142,67105	556,355138	480,817099	334,925501	555,910114	484,739045	357,38986
mmu-miR-574-5p	MIMAT0004893	165,059715	26,190311	13,529423	79,516957	8,182521	10,937941	116,474867	40,936827	28,515599	107,778339	36,60727	26,989918
mmu-miR-6931-3p	MIMAT0027763	181,62515	42,755746	22,086815	79,855946	8,52151	11,391083	127,217916	51,679876	35,998945	125,078781	53,907712	39,74524
mmu-miR-1964-5p	MIMAT0017349	334,755987	195,886583	101,191328	112,135417	40,80098	54,540494	303,658232	228,120193	158,90298	333,447789	262,276719	193,372168
mmu-miR-3064-5p	MIMAT0014834	159,545122	20,675718	10,680688	74,548019			88,493288	12,955249	9,024311	91,323613	20,152544	14,858128
mmu-miR-3113-3p	MIMAT0014960	151,429125	12,559721	6,488116	76,265703	4,931266	6,591843	106,280567	30,742528	21,414497	81,685942	10,514872	7,752437
mmu-miR-7042-5p	MIMAT0027988	24276,33465	24137,46525	12468,96101	11363,6008	11292,26637	15094,877	47923	47847,46196	33329,37868	38140	38068,82893	28067,50062
mmu-miR-7036b-5p	MIMAT0029808	167,817024	28,94762	14,953796	82,680602	11,346165	15,166926	122,622194	47,084154	32,797677	117,55258	46,381511	34,1963
mmu-miR-5134-5p	MIMAT0020645	208,781295	69,911891	36,115169	95,922925	24,588488	32,868531	191,689642	116,151602	80,908382	169,51709	98,346021	72,50885
mmu-miR-1894-5p	MIMAT0007877	210,386174	71,51677	36,94422	95,576848	24,242411	32,405914	195,576731	120,038691	83,616034	166,554096	95,383027	70,324285
mmu-miR-3620-3p	MIMAT0029879	201,980415	63,111011	32,601962	88,301682	16,967245	22,680875	167,676896	92,138856	64,181687	149,695188	78,524118	57,894498
mmu-miR-7009-3p	MIMAT0027923	188,681872	49,812469	25,732185	86,471883	15,137446	20,234901	144,834316	69,296276	48,270101	141,845067	70,673998	52,106738
mmu-miR-7013-3p	MIMAT0027931	166,059093	27,189689	14,045683	73,657203			94,500116	18,962076	13,208521	94,766776	23,595707	17,396713
mmu-miR-3089-5p	MIMAT0014899	168,788145	29,918741	15,455459	79,729407	8,39497	11,221931	118,042436	42,504396	29,607529	109,810666	38,639597	28,488318
mmu-miR-7080-3p	MIMAT0028067	180,355737	41,486333	21,43106	82,346306	11,011869	14,720058	131,090861	55,552822	38,696745	125,190252	54,019182	39,827425
mmu-miR-6896-5p	MIMAT0027692	223,397831	84,528427	43,665797	126,493768	55,159331	73,733943	285,287455	209,749415	146,106343	217,912466	146,741397	108,189938
mmu-miR-700-3p	MIMAT0003490	152,440029	13,570625	7,01033	75,217267			86,467348	10,929309	7,613091	81,416355	10,245286	7,553675
mmu-miR-7008-3p	MIMAT0027921	144,051847			77,39003	6,055593	8,094782	92,692224	17,154184	11,949188	78,651986	7,480916	5,515553
mmu-miR-6995-3p	MIMAT0027893	203,165712	64,296308	33,214265	85,769443	14,435006	19,295917	152,418158	76,880119	53,552822	150,879988	79,708919	58,768031
mmu-miR-6419	MIMAT0025175	155,001385	16,131981	8,333478	73,056778			89,981536	14,443497	10,060989	81,697223	10,526154	7,760754
mmu-miR-674-5p	MIMAT0003740	172,310913	33,441509	17,275255	78,036166	6,701729	8,958501	113,411978	37,873939	26,382065	111,387805	40,216736	29,651116
mmu-miR-712-3p	MIMAT0003743	159,673043	20,803639	10,746769	81,290064	9,955627	13,308132	121,728167	46,190128	32,17492	97,161601	25,990532	19,162377
mmu-let-7f-1-3p	MIMAT0004623	165,636361	26,766958	13,827307	84,149962	12,815525	17,131085	123,092816	47,554776	33,125501	115,788154	44,617085	32,895419
mmu-miR-6395	MIMAT0025146	158,117747	19,248343	9,943332	80,858159	9,523722	12,730785	111,142504	35,604465	24,801204	103,093119	31,92205	23,535585
mmu-miR-1188-3p	MIMAT0017328	214,633607	75,764203	39,138364	92,421153	21,086716	28,187556	192,596828	117,058788	81,540306	154,801741	83,630672	61,659473
mmu-miR-7067-3p	MIMAT0028039	188,960979	50,091576	25,876367	81,120744	9,786308	13,081795	132,882566	57,344526	39,944803	129,155676	57,984606	42,751065
mmu-miR-6994-5p	MIMAT0027890	223,123724	84,25432	43,524198	91,66159	20,327153	27,172213	183,32787	107,78983	75,083775	172,515511	101,344442	74,719535
mmu-miR-668-3p	MIMAT0003732	194,010811	55,141407	28,485015	88,453115	17,118678	22,883302	154,517638	78,979598	55,015268	143,308431	72,137361	53,185651
mmu-miR-6915-5p	MIMAT0027730	187,636701	48,767297	25,192269	83,94575	12,611313	16,858105	137,852654	62,314615	43,406846	132,282373	61,111304	45,056326
mmu-miR-6897-5p	MIMAT0027694	180,260451	41,391047	21,381837	92,404147	21,069711	28,164824	146,50931	70,971271	49,436862	145,431971	74,260902	54,751301
mmu-miR-7659-5p	MIMAT0029824	155,030869	16,161465	8,348709	73,605223			90,185895	14,647856	10,20334	81,053118	9,882049	7,285867
mmu-miR-6923-5p	MIMAT0027746	408,328328	269,458924	139,197417	154,182626	82,84819	110,746877	399,721661	324,183621	225,818428	474,57544	403,40437	297,423187
mmu-miR-7044-5p	MIMAT0027992	4119,570834	3980,70143	2056,355562	1454,728758	1383,394321	1849,245	5801,517682	5725,979643	3988,578203	5717,003022	5645,831953	4162,575952
mmu-miR-6967-3p	MIMAT0027837	196,151302	57,281898	29,590752	88,481452	17,147015	22,921181	166,339171	90,801132	63,249861	133,989429	62,818359	46,314909
mmu-miR-6981-3p	MIMAT0027865	169,972199	31,102795	16,067119	79,173605	7,839169	10,478967	119,984406	44,446367	30,960258	104,119684	32,948614	24,292453
mmu-miR-7117-3p	MIMAT0028132	300,026264	161,15686	83,250606	117,585497	46,25106	61,825858	293,828629	218,29059	152,055918	274,228982	203,057913	149,711148
mmu-miR-6347	MIMAT0025090	162,902899	24,033495	12,415252	74,720801			92,296986	16,758946	11,673874	90,339072	19,168003	14,132243
mmu-miR-365-3p	MIMAT0000711	198,074191	59,204787	30,58408	97,988654	26,654217	35,629883	172,146031	96,607991	67,294778	166,190922	95,019853	70,056523
mmu-miR-3473e	MIMAT0025587	3270,671048	3131,801644	1617,829883	1260,915068	1189,580631	1590,165581	4771,427077	4695,889037	3271,042132	4656,559768	4585,388698	3380,729162
mmu-miR-34a-5p	MIMAT0000542	176,89134	38,021936	19,641418	77,518351	6,183914	8,266314	112,379162	36,841123	25,662631	114,586429	43,41536	32,009407
mmu-miR-6915-3p	MIMAT0027731	160,585924	21,71652	11,218346	75,453791			92,801149	17,26311	12,025063	86,161319	14,99025	11,052056
mmu-miR-6907-5p	MIMAT0027714	843,631586	704,762182	364,066901	311,666279	240,331842	321,262311	1058,444844	982,906804	684,668982	1054,20089	983,02982	724,771181
mmu-miR-7048-3p	MIMAT0028001	175,06474	36,195336	18,69783	88,162993	16,828556	22,495483	145,854623	70,316583	48,980822	119,55828	48,387211	35,675068
mmu-miR-695	MIMAT0003481	171,343847	32,474443	16,775687	87,272895	15,938459	21,30565	135,033557	59,495518	41,443131	121,10383	49,932761	36,814576
mmu-miR-6923-3p	MIMAT0027747	200,708231	61,838827	31,944776	88,359686	17,02525	22,758412	151,394703	75,856663	52,839907	151,899393	80,728324	59,519621
mmu-miR-343	MIMAT0004868	150,812928	11,943524	6,1698	68,796855			85,238746	9,700706	6,757276	79,191103	8,020034	5,913035
mmu-miR-762	MIMAT0003892	2600,598167	2461,728763	1271,682824	824,554676	753,220239	1006,863148	3132,01092	3056,472881	2129,064696	3493,260675	3422,089605	2523,04851
mmu-miR-1898	MIMAT0007875	170,221531	31,352127	16,19592	75,358681			98,806267	23,268227	16,208081	93,989547	22,818478	16,823676
mmu-miR-6948-3p	MIMAT0027797	155,881598	17,012194	8,78818	70,565987			93,455111	17,917071	12,480596	78,485682	7,314613	5,39294
mmu-miR-7647-5p	MIMAT0029796	165,637638	26,768234	13,827967	74,08999			93,352076	17,814037	12,408825	92,480264	21,309195	15,710907
mmu-miR-1306-5p	MIMAT0019136	178,976941	40,107537	20,7188	88,983142	17,648705	23,591813	143,660546	68,122507	47,452482	128,919136	57,748067	42,576669
mmu-miR-6370	MIMAT0025114	227,9914	89,121996	46,038749	121,422733	50,088296	66,955263	261,564552	186,026512	129,581545	206,59364	135,422571	99,84476
mmu-miR-7658-5p	MIMAT0029822	553,80044	414,931036	214,345577	278,679205	207,344768	277,167016	755,081041	679,543001	473,353133	782,063974	710,892904	524,129257
mmu-miR-6975-5p	MIMAT0027852	211,613534	72,74413	37,578251	94,424616	23,090179	30,865674	167,716797	92,178757	64,209481	172,227246	101,056177	74,507002
mmu-miR-6921-5p	MIMAT0027742	228,516922	89,647518	46,310223	91,939481	20,605044	27,543683	181,643639	106,1056	73,910581	173,860182	102,689113	75,710938
mmu-miR-7083-3p	MIMAT0028073	174,560519	35,691115	18,437359	77,651815	6,317379	8,444722	119,216143	43,678104	30,425105	103,553939	32,38287	23,87534
mmu-miR-3059-3p	MIMAT0014812	155,421588	16,552184	8,550547	79,590636	8,256199	11,03643	104,256337	28,718298	20,004468	97,687689	26,51662	19,550253
mmu-miR-7054-5p	MIMAT0028012	153,154404	14,285	7,379362	82,266502	10,932065	14,61338	106,882132	31,344093	21,833533	101,771856	30,600787	22,56144
mmu-miR-3474	MIMAT0015646	210,775599	71,906195	37,145389	91,628459	20,294022	27,127927	155,034249	79,496209	55,375127	171,97464	100,803571	74,32076
mmu-miR-6960-3p	MIMAT0027821	179,302754	40,43335	20,887109	73,623991			111,427894	35,889854	25	94,190596	23,019527	16,971906
mmu-miR-3473c	MIMAT0020614	376,439011	237,569607	122,723995	133,976609	62,642172	83,73659	359,149254	283,611214	197,556676	363,527502	292,356433	215,549429
mmu-miR-6983-5p	MIMAT0027868	194,687146	55,817743	28,834397	84,887233	13,552796	18,116628	141,950633	66,412594	46,261398	135,667433	64,496363	47,552073
mmu-miR-7063-3p	MIMAT0028031	201,334104	62,4647	32,26809	85,625447	14,29101	19,103432	169,751567	94,213528	65,626853	120,654348	49,483278	36,48318
mmu-miR-467a-3p	MIMAT0002108	167,071912	28,202508	14,568886	70,735307			80,793128	5,255088	3,660567	105,479644	34,308575	25,295129
mmu-miR-6945-5p	MIMAT0027790	309,874804	171,0054	88,338176	121,541486	50,207049	67,114006	295,526808	219,988768	153,238827	281,028165	209,857096	154,72407
mmu-miR-5620-3p	MIMAT0022368	514,329943	375,460539	193,955859	158,480023	87,145586	116,491399	506,475755	430,937715	300,180735	496,621629	425,45056	313,677467
mmu-miR-346-3p	MIMAT0017039	307,324798	168,455394	87,020891	132,679049	61,344613	82,002085	307,221851	231,683811	161,38531	305,438725	234,267656	172,721561
mmu-miR-5046	MIMAT0020540	245,894805	107,025401	55,287311	100,256546	28,922109	38,661475	232,606267	157,068227	109,409908	174,555055	103,383985	76,223255
mmu-miR-324-5p	MIMAT0000555	155,822314	16,95291	8,757555	73,860453			87,481037	11,942998	8,319202	83,340538	12,169469	8,972343
mmu-miR-7240-5p	MIMAT0028448	286,374307	147,504903	76,198261	116,545379	45,210942	60,435487	256,73501	181,196971	126,217405	265,230391	194,059322	143,07664

mmu-let-7d-3p	MIMAT0000384	154,000755	15,131351	7,816572	71,459978			84,173176	8,635137	6,015026	83,856248	12,685178	9,352566
mmu-miR-409-3p	MIMAT0001090	156,504063	17,634659	9,109733	79,218148	7,883711	10,538509	102,948476	27,410437	19,093444	97,56844	26,39737	19,462333
mmu-miR-7080-5p	MIMAT0028066	193,18455	54,315146	28,058184	84,35065	13,016213	17,399353	126,794205	51,256165	35,703798	143,628735	72,457666	53,421806
mmu-miR-7231-3p	MIMAT0028431	160,203606	21,334202	11,020848	80,119495	8,785058	11,743379	104,142552	28,604513	19,925208	104,308606	33,137536	24,431742
mmu-miR-6366	MIMAT0025110	1546,16268	1407,293276	726,981264	662,659175	591,324738	790,450198	2143,358696	2067,820656	1440,393594	2107,633576	2036,462507	1501,449198
mmu-miR-6974-3p	MIMAT0027851	166,897866	28,028462	14,478977	71,597868			94,095837	18,557798	12,926911	91,652149	20,481079	15,100352
mmu-miR-7062-3p	MIMAT0028029	193,623865	54,754461	28,285126	84,491101	13,156665	17,587101	152,80224	77,2642	53,820363	118,571929	47,400859	34,947848
mmu-miR-452-5p	MIMAT0001637	153,283601	14,414197	7,446104	69,720514			88,604637	13,066597	9,101874	78,36062	7,18955	5,300733
mmu-miR-351-3p	MIMAT0017042	168,471137	29,601733	15,291699	78,500464	7,166027	9,579149	113,341759	37,80372	26,333152	100,671723	29,500654	21,750331
mmu-miR-7039-3p	MIMAT0027983	172,298998	33,429594	17,2691	76,983899	5,649462	7,551888	114,509121	38,971082	27,146308	99,355314	28,184245	20,779765
mmu-miR-8103	MIMAT0031407	156,950906	18,081502	9,340564	75,573262			88,02044	12,4824	8,694937	83,76345	12,59238	9,284148
mmu-miR-7081-5p	MIMAT0028068	792,808104	653,9387	337,812445	257,039151	185,704714	248,239789	815,776252	740,238213	515,631941	901,747823	830,576753	612,370125
mmu-miR-709	MIMAT0003499	971,407846	832,538442	430,073716	375,818496	304,484059	407,017446	1290,679222	1215,141182	846,437804	1099,285445	1028,114376	758,011257
mmu-miR-542-5p	MIMAT0003171	155,935841	17,066437	8,816201	75,03801			90,909865	15,371826	10,70764	79,518855	8,347786	6,154681
mmu-miR-6934-5p	MIMAT0027768	922,372278	783,502874	404,742863	343,527919	272,193482	363,85319	1021,830089	946,29205	659,164034	1169,677984	1098,506915	809,91048
mmu-miR-7049-3p	MIMAT0028003	157,372345	18,502941	9,558272	77,316351	5,981914	7,996292	102,158936	26,620897	18,543469	91,502946	20,331876	14,990347
mmu-miR-5113	MIMAT0020621	199,964727	61,095323	31,560696	84,484627	13,15019	17,578446	126,452089	50,91405	35,465489	150,180994	79,009925	58,252675
mmu-miR-7022-3p	MIMAT0027949	277,363031	138,493627	71,543205	125,974559	54,640123	73,039893	287,187631	211,649592	147,42996	244,420616	173,249546	127,733946
mmu-miR-3547-3p	MIMAT0027833	189,580568	50,711164	26,196434	84,463646	13,129209	17,550401	140,58542	65,047381	45,310424	122,620329	51,44926	37,932665
mmu-miR-7211-5p	MIMAT0028390	167,443519	28,574115	14,760851	80,840272	9,505835	12,706875	108,919081	33,381041	23,252422	110,490305	39,319236	28,989405
mmu-miR-7012-5p	MIMAT0027928	179,21838	40,348976	20,843523	84,095339	12,760902	17,058068	128,068328	52,530288	36,591322	119,181282	48,010213	35,397114
mmu-miR-3085-3p	MIMAT0014879	267,67571	128,806306	66,538917	113,188647	41,85421	55,948393	261,41185	185,87381	129,475177	210,71864	139,54757	102,886052
mmu-miR-365-2-5p	MIMAT0017179	213,575203	74,705799	38,591612	93,04549	21,711053	29,022135	166,088307	90,550268	63,075115	159,38052	88,209451	65,035329
mmu-miR-7653-5p	MIMAT0029812	2275,413646	2136,544242	1103,69861	744,021221	672,686784	899,210481	2400,052945	2324,514905	1619,200567	2999,849002	2928,677932	2159,264469
mmu-miR-6975-3p	MIMAT0027853	166,282316	27,412912	14,160995	73,254003			91,258082	15,720043	10,9502	92,535943	21,364874	15,751958
mmu-miR-5621-5p	MIMAT0022369	5400,689224	5261,81982	2718,15725	1292,97164	1221,637203	1633,017033	6348,513565	6272,975525	4369,602235	5269,227863	5198,056793	3832,438937
mmu-miR-30c-2-3p	MIMAT0005438	162,095145	23,225741	11,997981	80,812735	9,478298	12,670065	111,445716	35,907677	25,012415	100,034903	28,863834	21,280814
mmu-miR-935	MIMAT0035718	171,019269	32,149865	16,608016	75,956812			96,394142	20,856103	14,527854	93,65627	22,485201	16,577957
mmu-miR-7683-3p	MIMAT0029887	259,067656	120,198252	62,092158	130,302055	58,967618	78,82465	267,822033	192,283993	133,940355	246,726532	175,555463	129,43406
mmu-miR-6962-5p	MIMAT0027824	188,340655	49,471251	25,555919	84,23161	12,897173	17,240227	131,401145	55,863105	38,912881	126,825499	55,65443	41,033065
mmu-miR-6944-5p	MIMAT0027788	2919,964806	2781,095402	1436,661628	1284,594277	1213,25984	1621,818637	4103,061505	4027,523466	2805,474925	3997,441965	3926,270896	2894,772808
mmu-miR-598-5p	MIMAT0017283	204,672793	65,803389	33,992794	95,646633	24,312197	32,499199	169,232214	93,694174	65,265084	150,552236	79,381166	58,526385
mmu-miR-3100-5p	MIMAT0014919	287,21245	148,343046	76,63123	123,66565	52,331213	69,953471	291,550828	216,012788	150,469256	237,039559	165,868489	122,292018
mmu-miR-6897-3p	MIMAT0027695	305,869771	167,000367	86,269252	108,611065	37,276629	49,829335	276,266938	200,728899	139,82287	224,693084	153,522014	113,189172
mmu-miR-345-5p	MIMAT0000595	179,65515	40,785746	21,06915	88,105788	16,771351	22,419015	136,076656	60,538616	42,169728	123,619653	52,448583	38,669449
mmu-miR-1896	MIMAT0007873	154,888521	16,019117	8,275175	77,600798	6,266361	8,376525	96,447977	20,909938	14,565354	93,391475	22,220406	16,382728
mmu-miR-669n	MIMAT0009427	138,389599			77,498252	6,163815	8,239446	88,272288	12,734248	8,870368	79,906289	8,73522	6,440329
mmu-miR-324-3p	MIMAT0000556	167,720727	28,851323	14,904051	81,472046	10,137609	13,551395	114,839048	39,301008	27,376127	105,654066	34,482996	25,423727
mmu-miR-7088-5p	MIMAT0028082	217,27705	78,407646	40,503917	91,111813	19,777376	26,437302	148,462079	72,92404	50,797113	170,428125	99,257056	73,180541
mmu-miR-874-5p	MIMAT0017268	219,908917	81,039513	41,86349	95,689845	24,355408	32,556962	171,15683	95,61879	66,605724	167,406409	96,235339	70,95268
mmu-miR-6985-3p	MIMAT0027873	162,394963	23,52556	12,152862	78,533513	7,199076	9,623327	94,388041	18,850001	13,130453	107,760688	36,589618	26,976904
mmu-miR-1960	MIMAT0009433	157,248802	18,379398	9,494452	74,500464			85,523151	9,985112	6,955386	85,32677	14,155701	10,436758
mmu-miR-467a-5p	MIMAT0003409	155,694385	16,824981	8,691469	73,12267			89,350624	13,812585	9,621511	79,56319	8,392121	6,187368
mmu-miR-149-5p	MIMAT0000159	159,558297	20,688893	10,687494	78,65704	7,322603	9,788451	102,599485	27,061446	18,850345	96,042908	24,871839	18,337584
mmu-miR-6976-3p	MIMAT0027855	198,624487	59,755083	30,868353	87,897232	16,562795	22,140229	134,95404	59,416001	41,387742	145,405231	74,234162	54,731586
mmu-miR-6949-3p	MIMAT0027799	201,050353	62,180949	32,12151	83,175736	11,8413	15,828794	133,437626	57,899586	40,331444	134,069248	62,898179	46,373758
mmu-miR-6978-5p	MIMAT0027858	183,537012	44,667608	23,074447	77,786243	6,451806	8,624417	109,737514	34,199474	23,822522	116,512924	45,341855	33,429779
mmu-miR-7211-3p	MIMAT0028391	211,886339	73,016935	37,719177	95,614835	24,280398	32,456692	149,113298	73,575258	51,250736	173,430838	102,259768	75,394389
mmu-miR-6968-3p	MIMAT0027839	192,70071	53,831307	27,808241	83,980642	12,646205	16,904748	140,899885	65,361846	45,529473	118,550889	47,379819	34,932336
mmu-miR-483-3p	MIMAT0003120	202,115267	63,245863	32,671624	96,777598	25,443161	34,011011	171,424077	95,886037	66,791882	143,309163	72,138094	53,186191
mmu-miR-7060-5p	MIMAT0028024	153,71601	14,846606	7,669478	74,152338			81,823927	6,285888	4,378597	83,932121	12,761052	9,408506
mmu-miR-1895	MIMAT0007867	526,029891	387,160487	199,999833	180,302959	108,968522	145,663093	562,987761	487,449721	339,545624	450,39746	379,226391	279,597174
mmu-miR-3076-3p	MIMAT0014861	145,462042			76,129144	4,794708	6,4093	87,09655	11,55851	8,051377	75,806889	4,63582	3,417911
mmu-miR-185-3p	MIMAT0016996	160,658964	21,78956	11,256077	80,623398	9,288961	12,416969	112,305279	36,767239	25,611165	93,707884	22,536815	16,61601
mmu-miR-423-5p	MIMAT0004825	180,84839	41,978986	21,685555	90,877583	19,543146	26,124197	128,091315	52,553276	36,607334	136,987061	65,815992	48,525012
mmu-miR-764-5p	MIMAT0003894	151,843317	12,973913	6,70208	72,827241			81,402233	5,864193	4,084855	81,786807	10,615738	7,826803
mmu-miR-7053-3p	MIMAT0028011	188,179825	49,310421	25,472837	89,029897	17,69546	23,654312	132,782226	57,244186	39,874908	135,108115	63,937045	47,139697
mmu-miR-7011-3p	MIMAT0027927	170,04654	31,177136	16,105523	80,058827	8,724391	11,662283	107,150364	31,612324	22,020377	107,909947	36,738878	27,08695
mmu-let-7b-3p	MIMAT0004621	180,396522	41,527118	21,452129	83,379867	12,045431	16,101665	119,57816	44,04012	30,677277	119,453299	48,28223	35,597667
mmu-miR-483-5p	MIMAT0004782	178,134661	39,265258	20,283694	89,775036	18,440599	24,650373	129,909232	54,371192	37,873651	126,797181	55,626111	41,012187
mmu-miR-2183	MIMAT0011287	179,254424	40,38502	20,862142	87,495445	16,161008	21,603142	131,165471	55,627431	38,748716	119,718094	48,547025	35,792896
mmu-miR-6950-3p	MIMAT0027801	156,907948	18,038544	9,318373	72,777555			91,269349	15,731309	10,958048	78,454207	7,283138	5,369734
mmu-miR-297a-5p	MIMAT0000375	150,117399			81,409617	10,07518	13,467943	91,66215	16,124111	11,231664	87,71505	16,54398	12,197596
mmu-miR-1932	MIMAT0009395	155,949646	17,080242	8,823332	71,312089			88,099293	12,561253	8,749863	80,052392	8,881322	6,548048
mmu-miR-7054-3p	MIMAT0028013	179,875378	41,005975	21,182916	85,895101	14,560664	19,463891	135,151782	59,613742	41,525483	109,773547	38,602478	28,460951
mmu-miR-7662-5p	MIMAT0029830	173,306801	34,437397	17,789712	76,284471	4,950034	6,616931	118,178482	42,640443	29,702296	87,603636	16,432566	12,115452
mmu-miR-3093-3p	MIMAT0014908	239,968524	101,09912	52,225906	108,707425	37,372988	49,958143	195,405126	119,867086	83,496498	195,308418	124,137349	91,524358
mmu-miR-146b-3p	MIMAT0004826	155,436031	16,566627	8,558008	73,779687			88,378729	12,84069	8,944512	78,902224	7,731155	5,700049
mmu-miR-92b-3p	MIMAT0004899	180,975664	42,10626	21,751303	82,390398	11,055962	14,778998	128,644407	53,106368	36,992604	105,738739	34,56767	25,486156
mmu-miR-6368	MIMAT0025112	226,021797	87,152393	45,021289	90,303813	18,969376	25,357213	148,317657	72,779617	50,696512	165,49605	94,32498	69,544205
mmu-miR-194-2-3p	MIMAT0017073	172,069748	33,200344	17,150674	81,025266	9,690829	12,954165	107,53046	31,992421	22,285143	110,497527	39,326458	28,994729
mmu-miR-8110	MIMAT0031416	6620,103098	6481,233694	3348,083545	2314,692681	2243,358244	2998,7972	7280,384661	7204,846621	5018,720984	7888,8794	7817,708331	5763,863497
mmu-miR-3064-3p	MIMAT0014835	161,146848	22,27743	11,508102	72,461228			84,758099	9,22006	6,42247	88,975779	17,80471	13,12711
mmu-miR-6352	MIMAT0025095	238,34984	99,480436	51,389724	112,249745	40,915308	54,693321	211,713259	136,175219	94,856347	185,253366	114,082297	84,110939
mmu-miR-7074-3p	MIMAT0028055	164,437889	25,568485	13,208199	73,299345			93,828122	18,290082	12,740427	84,086291	12,915222	9,522174
mmu-miR-6386	MIMAT0025133	191,252251	52,382847	27,059994	87,422116	16,087679	21,505119	123,912429	48,374389	33,696423	136,238088	65,067019	47,972807
mmu-miR-7687-3p	MIMAT0029903	228,233453	89,364049	46,163789	109,778091	38,443654	51,38935	201,33021	125,79217	87,623767	174,644576	103,473507	76,289258
mmu-miR-26b-3p	MIMAT0004630	156,04091	17,171506	8,870477	75,83601			86,906397	11,368357	7,918921	80,627726	9,456657	6,972232
mmu-miR-760-5p	MIMAT0017245	189,087656	50,218253	25,941806	85,519438	14,185001	18,961725	134,416803	58,878763	41,013515	117,493575	46,322506	34,152796
mmu-miR-135a-2-3p	MIMAT0017064	224,355761	85,486357	44,160646	98,224665	26,890228	35,94537	165,296331	89,758292	62,523444	168,181276	97,010207	71,523977
mmu-miR-23a-5p	MIMAT0017019	200,095193	61,225789	31,628092	88,145987	16,81155	22,47275	129,848311	54,310272	37,831215	142,506942	71,335873	52,594727
mmu-miR-3473b	MIMAT0020367	3596,411338	3457,541934	1786,101196	1494,253766	1422,919329	1902,079844	4644,848356	4569,310316	3182,870472	4086,842397	4015,671327	2960,686226
mmu-miR-2137	MIMAT0011213	8478,120221	8339,250818	4307,900279	2380,909295	2309,574859	3087,311907	9161,445363	9085,907324	6329,022135	8138,25657	8067,085501	5947,725046
mmu-miR-671-5p	MIMAT0003731	585,591925	446,722521	230,768461	227,965061	156,630624	209,375154	616,222137	540,684097	376,627397	547,769417	476,598348	351,387862
mmu-miR-7001-3p	MIMAT0027905	166,398703	27,529299	14,221119	75,636141			92,294563	16,756523	11,672187	87,159797	15,988727	11,788217
mmu-miR-7666-3p	MIMAT0029839	245,759969	106,890565	55,217657	124,440538	53,106102	70,989299	227,575936	152,037896	105,905902	209,788869	138,6178	102,200548
mmu-miR-5126	MIMAT0020637	10823,76469	10684,89529	5519,616137	5978,574089	5907,239653	7896,471183	17044,87022	16969,33218	11820,42422	13955,43804	13884,26697	10236,63408
mmu-miR-30f	MIMAT0025179	149,8697			77,601249	6,266812	8,377127	88,13939	12,60135	8,777794	77,941654	6,770585	4,991837
mmu-miR-7023-3p	MIMAT0027951	181,175447	42,306043	21,854507	89,033872	17,699435	23,659626	132,384049	56,846009	39,597548	118,409691	47,238621	34,828233
mmu-miR-7648-3p	MIMAT0029799	482,984977	344,115573	177,763639	196,700814	125,366377	167,582838	459,309629	383,771589	267,325958	472,728595	401,557526	296,06154
mmu-miR-6392-5p	MIMAT0025141	159,015787	20,146383	10,407243	75,352125			85,179794	9,641754	6,716212	85,080654	13,909585	10,2553
mmu-miR-8101	MIMAT0031405	1586,796081	1447,926677	747,971715	564,54437	493,209933	659,295754	1671,452364	1595,914325	1111,675117	1673,211807	1602,040737	1181,15741
mmu-miR-7044-3p	MIMAT0027993	230,758111	91,888708	47,46798	100,192785	28,858348	38,576243	180,573568	105,035529	73,165196	161,812289	90,64122	66,828231
mmu-miR-669c-3p	MIMAT0017253	157,573379	18,703975	9,662122	82,70142	11,366983	15,194754	102,800647	27,262607	18,990469	100,035749	28,86468	21,281438
mmu-miR-6934-3p	MIMAT0027769	190,743669	51,874265	26,79727	94,05639	22,721953	30,37345	142,161548	66,623508	46,408316	133,667376	62,496307	46,077465
mmu-miR-7086-5p	MIMAT0028078	213,203364	74,33396	38,399527	96,687201	25,352764	33,890173	162,25867	86,72063	60,407483	147,649944	76,478875	56,386575

mmu-miR-7033-5p	MIMAT0027970	414,020093	275,150689	142,137676	142,450312	71,115876	95,063768	339,555583	264,017543	183,908202	337,455356	266,284286	196,326879
mmu-miR-129-1-3p	MIMAT0016994	166,110848	27,241444	14,072418	79,636941	8,302504	11,098328	106,056886	30,518846	21,258686	97,02356	25,85249	19,060602
mmu-miR-874-3p	MIMAT0004853	215,107439	76,238035	39,383137	92,685951	21,351514	28,541523	162,326194	86,788155	60,454518	136,108791	64,937722	47,877479
mmu-miR-3082-5p	MIMAT0014872	175,496243	36,62684	18,920737	73,441128			101,434498	25,896458	18,038843	87,610858	16,439789	12,120777
mmu-miR-3113-5p	MIMAT0014959	163,057816	24,188412	12,495279	74,682446			91,771544	16,233505	11,307865	82,805367	11,634298	8,57777
mmu-miR-7005-3p	MIMAT0027915	182,295864	43,42646	22,433293	73,99568			106,784473	31,246433	21,765506	89,864428	18,693359	13,782296
mmu-miR-7035-5p	MIMAT0027974	332,174291	193,304887	99,857672	131,258044	59,923607	80,102563	243,259013	167,720974	116,830352	297,764604	226,593535	167,063563
mmu-miR-210-3p	MIMAT0000658	166,314959	27,445555	14,177858	74,330345			94,044683	18,506644	12,891278	83,904648	12,733579	9,388251
mmu-miR-6977-3p	MIMAT0027857	148,186094			77,02836	5,693923	7,611321	86,862376	11,324337	7,888258	76,689838	5,518768	4,068894
mmu-miR-698-3p	MIMAT0003488	150,436297			76,276234	4,941797	6,605921	84,38291	8,844871	6,161122	76,889984	5,718915	4,216459
mmu-miR-6955-5p	MIMAT0027810	5478,924123	5340,054719	2758,571929	1465,441011	1394,106574	1863,564548	5205,987653	5130,449613	3573,746464	5072,099271	5000,928202	3687,099376
mmu-miR-3077-5p	MIMAT0014862	1779,944592	1641,075188	847,748607	645,995458	574,661022	768,175064	1706,033113	1630,495073	1135,763226	1954,531218	1883,360149	1388,569432
mmu-miR-693-5p	MIMAT0003472	150,802249	11,932845	6,164284	68,633641			82,727768	7,189729	5,00819	77,413029	6,241959	4,602091
mmu-miR-5099	MIMAT0020606	155,400237	16,530834	8,539518	79,9546	8,620163	11,522957	97,38215	21,84411	15,216076	92,707884	21,536815	15,878727
mmu-miR-6958-5p	MIMAT0027816	252,804527	113,935123	58,856744	103,733793	32,399356	43,309667	173,05013	97,512091	67,924551	193,426209	122,25514	90,136637
mmu-miR-6925-5p	MIMAT0027750	168,558894	29,689491	15,337033	86,113676	14,779239	19,756069	112,802825	37,264786	25,957744	109,592749	38,42168	28,327652
mmu-miR-504-3p	MIMAT0017277	280,030958	141,161554	72,921406	107,77596	36,441523	48,713012	188,088015	112,549975	78,399576	218,979482	147,808413	108,976631
mmu-miR-8096	MIMAT0031398	166,858713	27,989309	14,458751	80,463728	9,129291	12,203531	104,288169	28,750129	20,026641	99,603861	28,432792	20,963014
mmu-miR-7056-5p	MIMAT0028016	212,183904	73,3145	37,872893	107,371079	36,036642	48,17179	165,193506	89,655467	62,451818	165,468577	94,297507	69,52395
mmu-miR-129-2-3p	MIMAT0000544	157,681857	18,812453	9,71816	78,15785	6,823413	9,121161	95,715483	20,177443	14,055116	91,279272	20,108203	14,825436
mmu-miR-466h-3p	MIMAT0017274	188,460789	49,591385	25,617978	81,026516	9,692079	12,955835	113,703198	38,165158	26,584921	115,305618	44,134549	32,539653
mmu-miR-431-3p	MIMAT0004753	151,307472	12,438068	6,425272	82,631635	11,297198	15,101469	103,246313	27,708273	19,30091	89,707038	18,535969	13,666255
mmu-miR-7084-3p	MIMAT0028075	196,089539	57,220135	29,558846	81,328051	9,993614	13,35891	116,841742	41,303703	28,771155	120,893192	49,722123	36,659276
mmu-miR-6911-5p	MIMAT0027722	165,955043	27,085639	13,991932	72,082552			95,750979	20,21294	14,079842	80,903802	9,732733	7,175779
mmu-miR-667-5p	MIMAT0017239	529,308699	390,439295	201,693603	178,065616	106,73118	142,672337	440,478526	364,940486	254,20867	435,582091	364,411022	268,674054
mmu-miR-8114	MIMAT0031420	187,421248	48,551844	25,08097	80,767393	9,432956	12,609454	116,51109	40,97305	28,54083	109,870404	38,699335	28,532362
mmu-miR-7017-3p	MIMAT0027939	175,308425	36,439021	18,823714	83,566193	12,231756	16,350735	106,6631	31,125061	21,68096	113,831644	42,660574	31,452917
mmu-miR-6952-5p	MIMAT0027804	186,467323	47,597919	24,588191	81,141644	9,807207	13,109731	114,696838	39,158799	27,277067	111,391024	40,219955	29,653489
mmu-miR-24-2-5p	MIMAT0005440	157,484611	18,615207	9,616266	74,020123			86,862634	11,324595	7,888437	80,1387	8,967631	6,611682
mmu-miR-7023-5p	MIMAT0027950	297,979808	159,110404	82,193445	115,500631	44,166194	59,038925	211,918099	136,38006	94,999034	231,091911	159,920842	117,906919
mmu-miR-6953-3p	MIMAT0027807	198,683116	59,813712	30,89864	77,555375	6,220938	8,315806	122,039951	46,501912	32,392101	107,394971	36,223901	26,707267
mmu-miR-150-3p	MIMAT0004535	165,105169	26,235765	13,552903	77,972487	6,63805	8,873378	101,844667	26,306628	18,324557	92,108233	20,937164	15,436615
mmu-miR-6405	MIMAT0025157	206,035926	67,166522	34,696963	91,621391	20,286954	27,118478	132,096236	56,558197	39,397065	143,946835	72,775766	53,656335
mmu-miR-1967	MIMAT0009440	197,835546	58,966142	30,460801	86,26554	14,931103	19,959072	132,887941	57,349901	39,948547	119,588064	48,416995	35,697028
mmu-miR-491-5p	MIMAT0003486	285,696978	146,827574	75,848366	127,742973	56,408536	75,403811	264,418997	188,880957	131,569883	200,043799	128,87273	95,015673
mmu-miR-363-5p	MIMAT0017076	481,898787	343,029383	177,202534	174,031524	102,697087	137,279784	406,692782	331,154742	230,674342	396,402941	325,231871	239,787932
mmu-miR-292a-3p	MIMAT0000370	162,450442	23,581038	12,181521	74,280127			95,593126	20,055086	13,969885	76,851287	5,680217	4,187928
mmu-miR-466f-3p	MIMAT0004882	159,400254	20,53085	10,605851	76,611698	5,277261	7,054351	93,283554	17,745515	12,361094	89,85636	18,685291	13,776348
mmu-miR-7665-5p	MIMAT0029836	520,057742	381,188338	196,914733	189,243829	117,909392	157,614753	379,837387	304,299347	211,967527	493,269629	422,09856	311,206095
mmu-miR-3103-3p	MIMAT0014938	143,5793			79,397836	8,063399	10,778706	87,052529	11,51449	8,020714	81,850667	10,679597	7,873886
mmu-miR-1941-5p	MIMAT0009405	165,863116	26,993712	13,944444	77,170224	5,835788	7,800958	96,156758	20,618718	14,362498	95,09932	23,928251	17,641893
mmu-miR-3102-5p.2-5p	MIMAT0014934	577,808774	438,93937	226,747831	251,605196	180,270759	240,975979	557,898735	482,360696	336,000733	548,25382	477,082751	351,745005
mmu-miR-1907	MIMAT0007876	193,811078	54,941674	28,381837	89,12935	17,794913	23,787256	126,450589	50,912549	35,464444	127,002175	55,831106	41,163326
mmu-miR-328-3p	MIMAT0000565	184,342553	45,473149	23,490575	84,642453	13,308016	17,78942	116,064697	40,526658	28,229885	115,02542	43,854351	32,333068
mmu-miR-8115	MIMAT0031421	156,118377	17,248973	8,910495	71,493108			82,564539	7,0265	4,894489	82,237981	11,066912	8,159446
mmu-miR-5107-5p	MIMAT0020615	330,331189	191,461985	98,905664	140,228213	68,893776	92,093389	257,233221	181,695182	126,564446	277,96673	206,795661	152,466926
mmu-miR-6980-3p	MIMAT0027863	180,957473	42,088069	21,741906	85,765099	14,430662	19,290111	113,878935	38,340896	26,707336	116,066661	44,895592	33,100757
mmu-miR-6991-5p	MIMAT0027884	740,451175	601,581771	310,765839	333,646222	262,311785	350,643883	753,794484	678,256445	472,456949	735,856134	664,685065	490,061003
mmu-miR-7047-3p	MIMAT0027999	194,062525	55,193122	28,511729	86,615879	15,281442	20,427386	130,861743	55,323703	38,537147	114,989147	43,818077	32,306324
mmu-miR-7007-5p	MIMAT0027918	215,160438	76,291034	39,410515	98,330306	26,995869	36,086584	144,284435	68,746395	47,887067	154,424805	83,253736	61,381565
mmu-miR-6970-3p	MIMAT0027843	166,40498	27,535576	14,224361	77,268715	5,934278	7,932615	95,586766	20,048727	13,965456	95,586035	24,414965	18,000739
mmu-miR-6916-3p	MIMAT0027733	155,852486	16,983082	8,773141	76,979924	5,645487	7,546575	93,973677	18,435637	12,841817	85,544687	14,373618	10,597424
mmu-miR-129b-3p	MIMAT0029863	152,79094	13,921536	7,191604	77,809723	6,475286	8,655804	93,545427	18,007387	12,543508	84,879599	13,70853	10,107066
mmu-miR-486a-5p, mmu	MIMAT0003130, MIMA	233,574307	94,704903	48,922773	95,755286	24,420849	32,644 44	177,466191	101,928152	71,000672	132,425206	61,254137	45,161634
mmu-miR-8093	MIMAT0031394	172,597209	33,727805	17,42315	84,374643	13,040206	17,431426	106,903939	31,3659	21,848723	108,793685	37,622616	27,738515
mmu-miR-181a-2-3p	MIMAT0005443	150,028995			76,250459	4,916022	6,571467	88,954612	13,416573	9,345658	73,367785		
mmu-miR-125b-5p	MIMAT0000136	342,292204	203,4228	105,084396	133,830482	62,496046	83,541256	281,401336	205,863297	143,399367	239,937058	168,765989	124,428295
mmu-miR-1247-5p	MIMAT0014800	166,500024	27,63062	14,273459	81,718507	10,38407	13,880851	108,928121	33,390082	23,258719	93,633646	22,462576	16,561276
mmu-miR-8108	MIMAT0031413	204,70819	65,838786	34,01108	95,010516	23,676079	31,648873	146,78663	71,248591	49,630036	129,547049	58,37598	43,039618
mmu-miR-7004-3p	MIMAT0027913	188,820473	49,951069	25,803783	81,220197	9,885761	13,214738	108,468766	32,930726	22,938743	114,740487	43,569418	32,122991
mmu-miR-8094	MIMAT0031395	178,751471	39,882068	20,602327	82,814948	11,480511	15,346512	116,338562	40,800523	28,420652	101,025138	29,854069	22,010898
mmu-miR-5128	MIMAT0020639	1971,193656	1832,324252	946,54428	503,66152	432,327083	577,910928	1460,236176	1384,698137	964,547056	1629,159353	1557,988284	1148,678285
mmu-miR-92a-3p	MIMAT0000539	307,360575	168,491171	87,039373	123,069382	51,734945	69,156412	251,4026	175,86456	122,502977	198,180255	127,009186	93,641714
mmu-miR-7014-3p	MIMAT0027933	154,964081	16,094677	8,314208	74,058109			85,45634	9,9183	6,908847	77,36942	6,198351	4,569939
mmu-miR-3099-5p	MIMAT0014815	167,106429	28,237025	14,586717	76,157562	4,823126	6,447287	94,62738	19,08934	13,29717	92,438297	21,267228	15,679966
mmu-miR-3473a	MIMAT0015645	3551,138399	3412,268995	1762,714047	1098,459054	1027,124617	1373,003368	3156,258525	3080,720486	2145,954989	3016,543324	2945,372255	2171,572909
mmu-miR-6359	MIMAT0025102	159,688092	20,818688	10,754543	76,419779	5,085342	6,797804	93,236791	17,698752	12,32852	87,220386	16,049317	11,832889
mmu-miR-7240-3p	MIMAT0028449	143,170406			76,756575	5,422138	7,248014	83,326123	7,788083	5,424989	77,300036	6,128967	4,518783
mmu-miR-3473d	MIMAT0020632	168,481775	29,612371	15,297195	77,520482	6,186045	8,269163	103,128605	27,590565	19,218917	88,853936	17,682867	13,037277
mmu-miR-7039-5p	MIMAT0027982	157,875952	19,006548	9,818425	82,277402	10,942965	14,627951	105,114596	29,576556	20,60231	88,538254	17,367185	12,80453
mmu-miR-7213-3p	MIMAT0028395	161,965317	23,095913	11,930915	75,781899			85,04617	9,50813	6,623133	84,300206	13,129136	9,679889
mmu-miR-7056-3p	MIMAT0028017	177,721075	38,851671	20,070043	77,962037	6,6276	8,859409	104,349347	28,811308	20,069257	97,428764	26,257694	19,359352
mmu-miR-6941-3p	MIMAT0027783	151,87775	13,008346	6,719919	76,614342	5,279905	7,057885	86,467348	10,929309	7,613091	86,218745	15,047676	11,094396
mmu-miR-187-5p	MIMAT0016997	222,55362	83,684216	43,229693	105,686157	34,35172	45,919479	168,889778	93,351739	65,026552	147,147771	75,976702	56,016331
mmu-miR-5627-3p	MIMAT0022386	195,020222	56,150818	29,006458	83,678308	12,343871	16,500604	122,790784	47,252745	32,915113	110,303018	39,131949	28,851321
mmu-miR-7019-3p	MIMAT0027943	161,875669	23,006265	11,884604	74,447685			83,392209	7,854169	5,471023	85,617347	14,446278	10,650995
mmu-miR-194-5p	MIMAT0000224	165,438494	26,56909	13,725093	78,218148	6,883711	9,201764	105,645216	30,107176	20,971927	84,90865	13,737581	10,128485
mmu-miR-1249-5p	MIMAT0014804	687,660238	548,790834	283,495033	257,141616	185,807179	248,376759	527,160343	451,622304	314,589117	618,142802	546,971732	403,272963
mmu-miR-6959-5p	MIMAT0027818	178,100517	39,231113	20,266056	79,002155	7,667718	10,249781	103,937936	28,399896	19,782677	100,197931	29,026861	21,401012
mmu-miR-3060-3p	MIMAT0014827	158,55576	19,686356	10,169602	76,521363	5,186926	6,933596	87,58583	12,047791	8,392198	90,924955	19,753886	14,564204
mmu-miR-128-1-5p	MIMAT0016982	291,841224	152,97182	79,022368	118,12003	46,785593	62,540393	218,721796	143,183757	99,738324	193,460171	122,289102	90,161677
mmu-miR-200c-5p	MIMAT0004663	151,722908	12,853504	6,639879	75,810236			82,387817	6,849778	4,771389	76,716459	5,545389	4,088521
mmu-miR-1946b	MIMAT0009443	270,230102	131,360698	67,858468	110,242758	38,908321	52,010491	196,746109	121,20807	84,430594	172,891607	101,720538	74,996824
mmu-miR-6919-3p	MIMAT0027739	188,946561	50,077157	25,868918	80,826279	9,491842	12,688169	109,083036	33,544996	23,366629	108,953556	37,782486	27,856385
mmu-miR-7083-5p	MIMAT0028072	547,949604	409,0802	211,323145	219,506745	148,172308	198,068544	449,947403	374,409363	260,804459	453,240866	382,069797	281,693568
mmu-miR-7089-5p	MIMAT0028084	203,467413	64,598009	33,370118	90,113144	18,778707	25,102337	127,498336	51,960297	36,194279	126,643856	55,472786	40,899143
mmu-miR-3961	MIMAT0019338	143,834561			78,065465	6,731028	8,997666	86,727252	11,189213	7,794133	76,672913	5,501844	4,056416
mmu-miR-7088-3p	MIMAT0028083	144,825764			78,550519	7,216082	9,646059	80,55818			88,379405	17,208336	12,687413
mmu-miR-7016-3p	MIMAT0027937	204,157545	65,288141	33,726627	90,215178	18,880742	25,238731	136,480208	60,942168	42,450833	118,689775	47,518706	35,034735
mmu-miR-8119	MIMAT0031425	1151,992309	1013,122905	523,360256	394,645919	323,311482	432,184904	914,922102	839,384062	584,69453	970,017066	898,845997	662,703878
mmu-miR-1899	MIMAT0007869	156,834254	17,96485	9,280304	74,629216			87,210125	11,672085	8,130491	76,517164	5,346095	3,941585
mmu-miR-721	MIMAT0003515	178,682171	39,812767	20,566528	81,781387	10,44695	13,964905	111,258048	35,720008	24,881689	98,341434	27,170365	20,032248
mmu-miR-134-5p	MIMAT0000146	158,773104	19,9037	10,281878	73,207248			89,005298	13,467259	9,380965	76,425212	5,254143	3,87379
mmu-miR-1947-5p	MIMAT0009413	153,097035	14,227631	7,349727	70,194749			83,13768	7,59964	5,293725	76,807735	5,636665	4,155818
mmu-miR-6940-3p	MIMAT0027781	161,073537	22,204133	11,470238	71,788087			90,422811	14,884772	10,36837	77,028076	5,857006	4,318271
mmu-miR-5123	MIMAT0020633	159,635126	20,765722	10,727182	71,447316			86,782539	11,2445	7,832645	79,065251	7,894182	5,820246

mmu-miR-6984-5p	MIMAT0027870	341,954492	203,085088	104,909941	117,117018	45,782581	61,199622	221,762988	146,224949	101,856744	219,023937	147,852867	109,009406
mmu-miR-6930-3p	MIMAT0027761	163,141842	24,272438	12,538686	75,810154			84,917921	9,379882	6,533798	83,882931	12,711862	9,37224
mmu-miR-3057-3p	MIMAT0014823	157,841426	18,972022	9,80059	79,640035	8,305598	11,102464	90,733353	15,195314	10,584686	92,602903	21,431834	15,801327
mmu-miR-7212-3p	MIMAT0028393	162,912948	24,043544	12,420443	76,52712	5,192683	6,941291	92,39269	16,854651	11,74054	88,307421	17,136352	12,634341
mmu-miR-7647-3p	MIMAT0029797	230,539532	91,670128	47,355066	97,035921	25,701485	34,356323	146,281692	70,743653	49,278308	143,720061	72,548992	53,489139
mmu-miR-6998-5p	MIMAT0027898	295,06982	156,200416	80,6902	107,418265	36,083828	48,234866	179,338152	103,800113	72,304635	192,881504	121,710435	89,735035
mmu-miR-320-3p	MIMAT0000666	337,29033	198,420926	102,500522	140,426669	69,092232	92,358674	256,845069	181,307029	126,294068	231,025803	159,854734	117,858179
mmu-miR-7214-3p	MIMAT0028397	165,599066	26,729662	13,808041	83,014735	11,680298	15,613577	96,427412	20,889373	14,551029	101,404504	30,233435	22,290598
mmu-miR-376b-3p	MIMAT0001092	151,208	12,338596	6,373887	69,799867			81,577971	6,039931	4,207269	76,287058	5,115989	3,771931
mmu-miR-1927	MIMAT0009390	197,386465	58,517061	30,228814	86,655115	15,320678	20,479835	120,007456	44,469416	30,976314	115,16752	43,996451	32,437836
mmu-miR-466b-3p, mmu	MIMAT0004876, MIMA	158,846807	19,977403	10,319951	78,703714	7,369277	9,850842	93,786218	18,248178	12,711237	88,133113	16,962044	12,505827
mmu-miR-5118	MIMAT0020626	185,867203	46,997799	24,27818	75,697239			96,007944	20,469905	14,258838	92,997609	21,826539	16,092337
mmu-miR-669b-5p	MIMAT0003476	159,549484	20,68008	10,682941	69,222204			87,816081	12,278042	8,552585	77,655988	6,484919	4,781221
mmu-miR-7119-5p	MIMAT0028135	449,270701	310,401297	160,347477	139,605126	68,27069	91,260481	288,212377	212,674338	148,143773	294,431221	223,260152	164,605916
mmu-miR-6977-5p	MIMAT0027856	205,848033	66,978629	34,599901	86,047784	14,713347	19,667988	125,321356	49,783317	34,677848	115,565502	44,394433	32,731261
mmu-miR-7674-3p	MIMAT0029857	182,355712	43,486308	22,46421	81,741619	10,407182	13,911745	114,802825	39,264786	27,350895	95,192964	24,021894	17,710934
mmu-miR-3075-5p	MIMAT0014858	242,545767	103,676364	53,557261	104,909137	33,574701	44,880802	163,44298	87,90494	61,232444	153,312602	82,141533	60,561556
mmu-miR-24-3p	MIMAT0000219	627,187078	488,317674	252,255735	263,438213	192,103776	256,793701	574,769038	499,230999	347,752176	453,120589	381,949519	281,60489
mmu-miR-106a-5p	MIMAT0000385	599,242483	460,373079	237,820082	209,564174	138,229738	184,776866	457,847321	382,309281	266,30735	418,088499	346,91743	255,776325
mmu-miR-7087-3p	MIMAT0028081	181,573669	42,704265	22,060221	86,344893	15,010456	20,065148	109,842775	34,304735	23,895844	109,291737	38,120668	28,105721
mmu-miR-375-5p	MIMAT0017078	174,71945	35,850046	18,51946	83,482414	12,147977	16,238744	105,942389	30,404349	21,17893	100,62011	29,44904	21,712277
mmu-miR-7072-5p	MIMAT0028050	326,183976	187,314572	96,763188	120,745535	49,411098	66,050023	192,880717	117,342678	81,738056	232,076628	160,905558	118,632934
mmu-miR-6898-5p	MIMAT0027696	283,913053	145,043649	74,926824	122,314961	50,980525	68,147945	199,193179	123,65514	86,135164	192,919294	121,748224	89,762897
mmu-miR-344g-5p	MIMAT0014929	158,829835	19,960431	10,311183	75,562012			87,158245	11,620205	8,094352	77,384767	6,213697	4,581253
mmu-miR-7047-5p	MIMAT0027998	426,324256	287,454852	148,493776	149,943603	78,609166	105,080385	286,476428	210,938388	146,934553	293,039728	221,868659	163,579992
mmu-miR-7224-3p	MIMAT0028417	216,815157	77,945753	40,265312	99,394866	28,060429	37,50963	146,541916	71,003876	49,459574	133,210672	62,039602	45,740745
mmu-miR-3098-5p	MIMAT0014917	181,581231	42,711827	22,064127	84,272977	12,93854	17,295525	114,941356	39,403317	27,447392	99,268893	28,097824	20,716048
mmu-miR-378b	MIMAT0019348	179,714176	40,844772	21,099642	81,310082	9,975645	13,334891	113,582083	38,044043	26,500555	92,373705	21,202636	15,632343
mmu-miR-216a-3p	MIMAT0017054	148,669934			77,495239	6,160802	8,23542	80,786979	5,248939	3,656283	79,766613	8,595544	6,337348
mmu-miR-665-3p	MIMAT0003733	217,744953	78,875549	40,745627	112,512412	41,177975	55,044439	160,927033	85,388994	59,479897	148,133327	76,962257	56,742964
mmu-miR-6950-5p	MIMAT0027800	208,92992	70,060516	36,191946	86,628541	15,294104	20,444312	123,029153	47,491113	33,081155	119,400777	48,229708	35,558944
mmu-miR-1897-5p	MIMAT0007864	213,756545	74,887141	38,68529	104,043483	32,709046	43,723644	149,253329	73,71529	51,348279	136,941755	65,770686	48,491609
mmu-miR-298-5p	MIMAT0000376	175,975689	37,106285	19,16841	83,659171	12,324734	16,475023	94,868429	19,33039	13,46508	111,420921	40,249852	29,675532
mmu-miR-182-5p	MIMAT0000211	152,131188	13,261784	6,850788	71,693777			81,920602	6,382563	4,445938	76,385556	5,214487	3,844552
mmu-miR-7005-5p	MIMAT0027914	13321,35414	13182,48474	6809,823919	3316,505249	3245,170812	4337,964821	8314,238095	8238,700056	5738,878151	10551,46773	10480,29666	7726,944625
mmu-miR-3074-1-3p	MIMAT0014857	151,257873	12,388469	6,39965	73,135332			81,748591	6,210552	4,326119	75,777048	4,605979	3,39591
mmu-miR-532-3p	MIMAT0004781	196,055627	57,186223	29,541328	98,650977	27,31654	36,51524	134,733494	59,195455	41,234114	123,330717	52,159648	38,456422
mmu-miR-679-3p	MIMAT0017248	156,180779	17,311375	8,942731	76,990455	5,656018	7,560652	93,910543	18,372504	12,797839	80,805367	9,634298	7,103204
mmu-miR-574-3p	MIMAT0004894	190,061904	51,1925	26,445083	86,608892	15,274455	20,418046	121,420774	45,882734	31,960797	104,450706	33,279636	24,53651
mmu-miR-378c	MIMAT0025138	198,506591	59,637187	30,80745	85,262446	13,928009	18,618192	124,727909	49,189869	34,264467	105,503108	34,332039	25,312429
mmu-miR-7036a-3p	MIMAT0027977	196,23818	57,368776	29,635632	91,384048	20,049611	26,801211	130,171362	54,633323	38,056244	111,805079	40,63401	29,958765
mmu-miR-300-3p	MIMAT0000378	150,902948	12,033544	6,216303	74,770999			81,347462	5,809422	4,046702	75,835151	4,664082	3,438748
mmu-miR-7226-5p	MIMAT0028420	167,861209	28,991805	14,976622	74,893133			82,942877	7,404838	5,15803	88,497978	17,326909	12,774836
mmu-miR-6961-5p	MIMAT0027822	269,698263	130,828859	67,58373	94,999267	23,66483	31,633835	167,466177	91,928138	64,034906	145,283382	74,112312	54,641748
mmu-miR-3102-3p.2-3p	MIMAT0014935	221,794171	82,924767	42,837377	90,77893	19,444493	25,992322	149,066067	73,528027	51,217836	113,310353	42,139284	31,068578
mmu-miR-7079-5p	MIMAT0028064	193,411587	54,542183	28,175467	92,775467	21,44103	28,661183	122,142002	46,603962	32,463187	119,252363	48,081294	35,449521
mmu-miR-669l-5p	MIMAT0009418	141,111736			78,331144	6,996707	9,352811	85,614919	10,07688	7,019309	76,777894	5,606825	4,133817
mmu-miR-6378	MIMAT0025124	6470,738701	6331,869297	3270,924704	2032,805452	1961,471016	2621,985946	4586,588273	4511,050233	3142,287915	5337,638134	5266,467065	3882,876668
mmu-miR-484	MIMAT0003127	205,452953	66,583549	34,395811	93,155843	21,821406	29,169648	143,045292	67,507252	47,023911	110,093958	38,922889	28,697185
mmu-miR-449b	MIMAT0005447	161,771844	22,90244	11,83097	72,98523			88,869658	13,331618	9,286481	77,655199	6,48413	4,780639
mmu-miR-7658-3p	MIMAT0029823	193,747408	54,878004	28,348946	89,622353	18,287917	24,446275	118,982438	43,444398	30,262312	115,09238	43,921311	32,382436
mmu-miR-6989-5p	MIMAT0027880	931,242102	792,372698	409,324847	321,917821	250,583384	334,966005	662,907066	587,369026	409,146983	713,005187	641,834118	473,213388
mmu-miR-667-3p	MIMAT0003734	205,674078	66,804674	34,510039	84,752437	13,418	17,93644	131,894042	56,356003	39,256221	101,602277	30,431207	22,436412
mmu-miR-9769-5p	MIMAT0036461	171,737036	32,867632	16,978801	77,900489	6,566052	8,777135	97,845442	22,307402	15,538794	91,137228	19,966159	14,72071
mmu-miR-467c-5p	MIMAT0004885	157,57022	18,700816	9,66049	71,617886			81,644056	6,106017	4,253303	80,77874	9,60767	7,083572
mmu-miR-7094b-2-5p	MIMAT0028096	155,085759	16,216355	8,377064	73,640116			80,003846			84,495498	13,324428	9,823875
mmu-miR-6418-3p	MIMAT0025174	159,667155	20,797752	10,743728	73,735594			93,614207	18,076167	12,591419	74,919881		
mmu-miR-1903	MIMAT0007868	160,571464	21,70206	11,210877	68,952182			89,106832	13,568793	9,451691	76,149136	4,978066	3,670244
mmu-miR-21a-3p	MIMAT0004628	162,892891	24,023487	12,410082	72,886658			87,610528	12,072489	8,409402	79,466334	8,295264	6,115957
mmu-miR-5132-3p	MIMAT0022988	172,05154	33,182136	17,141268	79,581949	8,247512	11,024818	106,190768	30,652728	21,351945	86,869164	15,698094	11,573938
mmu-miR-6952-3p	MIMAT0027805	173,343183	34,473779	17,808507	79,389517	8,05508	10,767586	107,405877	31,867837	22,198361	86,237248	15,066179	11,108038
mmu-miR-6944-3p	MIMAT0027789	181,838333	42,968929	22,196941	82,735513	11,401076	15,240328	112,893351	37,355311	26,020802	95,042006	23,870937	17,599636
mmu-miR-320-5p	MIMAT0017057	171,928635	33,059231	17,077778	73,820254			96,114644	20,576604	14,333162	78,681037	7,509968	5,536972
mmu-miR-669f-5p	MIMAT0017327	173,815763	34,946359	18,052633	78,9437	7,609263	10,171642	104,966102	29,428063	20,498873	87,753804	16,582734	12,226168
mmu-miR-7222-3p	MIMAT0028413	235,954719	97,085315	50,152449	102,595022	31,260585	41,787421	143,408957	67,870917	47,277231	151,628165	80,457096	59,31965
mmu-miR-7a-2-3p	MIMAT0017070	151,489652	12,620248	6,519383	76,381362	5,046925	6,74645	89,266138	13,728099	9,56266	78,940978	7,769909	5,728622
mmu-miR-7648-5p	MIMAT0029798	174,810739	35,941335	18,566618	81,149962	9,815525	13,120851	103,854482	28,316442	19,724545	93,95333	22,782261	16,796974
mmu-miR-486a-3p	MIMAT0017206	1257,281513	1118,412109	577,750681	365,897888	294,563451	393,756126	867,872442	792,334402	551,92088	840,89467	769,723601	567,50413
mmu-miR-207	MIMAT0000240	185,859045	46,989641	24,273965	86,312663	14,978227	20,022065	117,63004	42,092	29,320264	100,40208	29,231011	21,551527
mmu-miR-130b-3p	MIMAT0000387	204,47709	65,607686	33,891698	98,534518	27,200081	36,359564	149,115524	73,577485	51,252287	110,557998	39,386929	29,039313
mmu-miR-6903-3p	MIMAT0027707	151,21557	12,346166	6,377797	72,048541			85,98814	10,450101	7,279286	74,123185		
mmu-miR-5622-3p	MIMAT0022372	3281,637319	3142,767915	1623,494853	969,900721	898,566285	1201,153701	2342,643275	2267,105235	1579,210387	2276,414878	2205,243809	1625,888784
mmu-miR-3095-3p	MIMAT0014912	166,175099	27,305695	14,105609	85,699657	14,365221	19,202632	104,859058	29,311019	20,417343	94,258345	23,087276	17,021856
mmu-miR-20b-5p	MIMAT0003187	639,361962	500,492558	258,545051	231,987272	160,652835	214,751823	521,15353	445,61549	310,404916	368,934391	297,763322	219,535837
mmu-miR-7025-5p	MIMAT0027954	192,569631	53,700227	27,740528	82,664315	11,329878	15,145154	108,149638	32,611598	22,716447	105,344146	34,173077	25,195229
mmu-miR-193b-5p	MIMAT0017271	238,400924	99,53152	51,416113	113,709556	42,375119	56,644715	144,417271	68,879231	47,979598	169,831018	98,659949	72,740303
mmu-miR-7684-5p	MIMAT0029890	275,95185	137,082446	70,814216	125,415214	54,080777	72,292191	196,323508	120,785468	84,156978	173,165992	101,994923	75,199123
mmu-miR-7003-3p	MIMAT0027911	288,304999	149,435595	77,19562	112,290313	40,955876	54,747549	180,656038	105,117998	73,222642	171,096602	99,925533	73,673397
mmu-miR-6381	MIMAT0025127	153,803484	14,934081	7,714665	77,921838	6,587401	8,805674	86,795048	11,257009	7,841359	85,370379	14,199309	10,46891
mmu-miR-211-5p	MIMAT0000668	161,499668	22,630264	11,690369	76,349113	5,014676	6,703341	88,58604	13,048001	9,08892	86,476882	15,305813	11,284715
mmu-miR-669p-3p	MIMAT0014890	166,452316	27,582912	14,248814	78,293158	6,958721	9,302033	86,397387	10,859348	7,564358	96,204357	25,033288	18,456618
mmu-miR-214-5p	MIMAT0004664	154,667405	15,798001	8,16095	74,203867			78,012886			83,397739	12,22667	9,014516
mmu-miR-6391	MIMAT0025140	3729,92303	3591,053626	1855,070828	1491,981818	1420,647381	1899,042829	3029,220305	2953,682265	2057,463253	2904,231975	2833,060905	2088,76766
mmu-miR-6918-3p	MIMAT0027737	151,900428	13,031024	6,731582	71,098308			86,185685	10,647646	7,416891	71,729325		
mmu-miR-155-5p	MIMAT0000165	215,665903	76,796499	39,671628	104,715456	33,381019	44,6219	150,568988	75,030949	52,264735	125,880389	54,709319	40,336251
mmu-miR-6962-3p	MIMAT0027825	165,690007	26,820603	13,85502	83,9462	12,611763	16,858707	99,569622	24,031582	16,739816	94,050976	22,879907	16,868967
mmu-miR-152-3p	MIMAT0000162	172,011749	33,142345	17,120713	79,59373	8,259293	11,040566	102,692495	27,154456	18,915134	87,209888	16,038819	11,825149
mmu-miR-15b-3p	MIMAT0004521	165,994179	27,124775	14,012149	76,074152			86,106574	10,568535	7,361784	81,877118	10,706049	7,893388
mmu-miR-188-5p	MIMAT0000217	423,427726	284,558322	146,997482	194,334432	122,999995	164,419589	325,754811	250,216771	174,294919	293,092977	221,921907	163,619251
mmu-miR-210-5p	MIMAT0017052	207,888521	69,019117	35,653979	82,712401	11,377964	15,209433	112,533609	36,99557	25,770214	110,439425	39,268355	28,951891
mmu-miR-5121	MIMAT0020629	196,981278	58,111874	30,019502	95,861295	24,526859	32,786148	134,745019	59,206979	41,242142	106,740431	35,569361	26,224686
mmu-miR-7014-5p	MIMAT0027932	215,271387	76,401983	39,467829	98,367329	27,032893	36,136075	129,258997	53,720958	37,420713	130,535831	59,364761	43,76863
mmu-miR-2136	MIMAT0011212	441,099093	302,229689	156,126179	191,743307	120,40887	160,955917	295,15922	219,62118	152,982775	325,005647	253,834578	187,147921
mmu-miR-8112	MIMAT0031418	199,717916	60,848512	31,433198	89,388104	18,053668	24,133144	116,976595	41,438555	28,86509	112,701677	41,530607	30,619811
mmu-miR-669f-3p	MIMAT0005839	157,772499	18,903095	9,764984	82,439203	11,104766	14,844237	92,021634	16,483594	11,482071	91,313291	20,142222	14,850518
mmu-miR-1966-5p	MIMAT0009439	201,9553	63,085896	32,588989	88,404229	17,069792	22,817954	118,324085	42,786046	29,803719	111,111729	39,940659	29,447569

mmu-miR-7667-5p	MIMAT0029840	203,928692	65,059288	33,608406	90,977036	19,642599	26,25714	126,237448	50,699409	35,315975	109,701733	38,530664	28,408004
mmu-miR-668-5p	MIMAT0017237	162,02708	23,157676	11,96282	80,047947	8,71351	11,647738	90,769834	15,231795	10,610098	90,810384	19,639315	14,479733
mmu-miR-6943-3p	MIMAT0027787	181,182337	42,312933	21,858066	80,585042	9,250606	12,365698	100,020453	24,482414	17,053854	97,34798	26,17691	19,299791
mmu-miR-7b-3p	MIMAT0017071	170,992911	32,123507	16,5944	80,631716	9,297279	12,428089	101,29085	25,75281	17,938781	88,591622	17,420553	12,843877
mmu-miR-466h-5p	MIMAT0004884	150,788444	11,91904	6,157152	76,120457	4,78602	6,397687	80,441924			89,189637	18,018568	13,284784
mmu-miR-18b-5p	MIMAT0004858	188,245974	49,37657	25,507008	81,956731	10,622294	14,199295	107,717919	32,17988	22,415722	97,603805	26,432736	19,488407
mmu-miR-466f	MIMAT0005844	181,638583	42,769179	22,093754	78,590267	7,25583	9,699193	101,818259	26,28022	18,306162	91,82082	20,64975	15,22471
mmu-miR-6914-3p	MIMAT0027729	183,971925	45,102521	23,299115	80,301395	8,966958	11,986534	108,668007	33,129968	23,07753	90,333316	19,162247	14,127999
mmu-miR-1905	MIMAT0007866	198,331608	59,462204	30,717057	80,994717	9,66028	12,913328	116,848814	41,310774	28,776081	94,5021	23,331031	17,201572
mmu-miR-671-3p	MIMAT0004821	155,736713	16,867309	8,713335	79,03926	7,704823	10,299381	89,888526	14,350487	9,9962	84,740098	13,569029	10,004215
mmu-miR-7094-3p	MIMAT0028095	156,200488	17,331084	8,952912	66,854941			82,495501	6,957462	4,846399	77,365418	6,194349	4,566988
mmu-miR-7671-3p	MIMAT0029849	1034,317387	895,447983	462,571602	284,451737	213,1173	284,883417	571,919965	496,381925	345,76758	662,332839	591,161769	435,853526
mmu-miR-3103-5p	MIMAT0014937	260,053859	121,184455	62,601612	111,080875	39,746439	53,13084	155,26429	79,72625	55,535367	158,761465	87,590396	64,57891
mmu-miR-532-5p	MIMAT0002889	143,60691			79,701501	8,367064	11,184629	82,700844	7,162804	4,989435	80,120986	8,949917	6,598622
mmu-miR-1968-5p	MIMAT0009441	147,957216			76,553694	5,219257	6,976814	85,907381	10,369341	7,223031	75,000546		
mmu-miR-1187	MIMAT0005837	158,525016	19,655612	10,15372	79,201942	7,867505	10,516845	84,927946	9,389906	6,54078	91,231718	20,060648	14,790375
mmu-miR-9768-5p	MIMAT0036459	207,320016	68,450612	35,3603	88,007216	16,672779	22,287249	110,441841	34,903802	24,31314	118,846314	47,675244	35,150147
mmu-miR-3620-5p	MIMAT0029878	347,422163	208,552759	107,734436	135,74361	64,409173	86,09862	208,796713	133,258673	92,824752	215,681577	144,510508	106,545142
mmu-miR-7028-5p	MIMAT0027960	189,581844	50,712441	26,197094	92,096138	20,761701	27,753094	111,579856	36,041817	25,105853	111,914802	40,743732	30,039662
mmu-miR-1946a	MIMAT0009412	242,868205	103,998801	53,723827	103,869983	32,535546	43,491718	155,300512	79,762473	55,560599	130,258903	59,087833	43,564455
mmu-miR-5130	MIMAT0020641	535,250941	396,381537	204,763254	183,450685	112,116248	149,870799	321,76085	246,222811	171,512824	328,201559	257,03049	189,504212
mmu-miR-466f-5p	MIMAT0004881	164,964065	26,094661	13,480012	86,192661	14,858224	19,861652	101,994723	26,456684	18,429083	92,163066	20,991997	15,477043
mmu-miR-7069-3p	MIMAT0028045	211,249804	72,3804	37,390354	90,328256	18,993819	25,389887	135,072461	59,534421	41,47023	101,482801	30,311732	22,348325
mmu-miR-1971	MIMAT0009446	171,381747	32,512343	16,795266	78,035716	6,701279	8,957899	87,641118	12,103078	8,43071	95,237361	24,066292	17,743668
mmu-miR-6906-5p	MIMAT0027712	181,031814	42,16241	21,780309	91,969599	20,635162	27,583943	112,186587	36,648547	25,528487	104,549874	33,378805	24,609625
mmu-miR-17-3p	MIMAT0000650	183,435683	44,566279	23,022102	92,334362	20,999925	28,071539	114,613398	39,075358	27,218945	104,630601	33,459532	24,669144
mmu-miR-30a-3p	MIMAT0000129	152,553281	13,683877	7,068834	73,803248			80,925768	5,387728	3,75296	75,797186	4,626117	3,410758
mmu-miR-17-5p	MIMAT0000649	861,245245	722,375841	373,165786	319,49989	248,165453	331,733849	655,336679	579,798639	403,873635	489,488386	418,317316	308,418248
mmu-miR-7009-5p	MIMAT0027922	2545,391352	2406,521948	1243,16402	745,87327	674,538833	901,686197	1626,080369	1550,542329	1080,070088	1539,416293	1468,245224	1082,512252
mmu-miR-3110-5p	MIMAT0014951	173,44642	34,577016	17,861837	81,124288	9,789852	13,086532	100,428397	24,890357	17,338018	89,955641	18,784572	13,849546
mmu-miR-3078-3p	MIMAT0014865	138,427525			79,236916	7,902479	10,563597	80,921586	5,383547	3,750048	80,489804	9,318735	6,870545
mmu-miR-7012-3p	MIMAT0027929	173,515679	34,646275	17,897614	80,290064	8,955627	11,971387	100,423747	24,885708	17,334779	88,373648	17,202579	12,683169
mmu-miR-7118-3p	MIMAT0028134	171,685977	32,816573	16,952425	77,075034	5,740597	7,673712	94,077953	18,539914	12,914453	87,219478	16,048409	11,832219
mmu-miR-139-3p	MIMAT0004662	262,90642	124,037016	64,075192	109,618421	38,283984	51,175912	159,160739	83,622699	58,249539	149,210835	78,039765	57,537393
mmu-miR-669a-3p, mmu	MIMAT0017243, MIMA	198,205536	59,336132	30,651931	78,55015	7,215713	9,645566	102,212833	26,674793	18,581013	100,824202	29,653133	21,862751
mmu-miR-691	MIMAT0003470	170,454214	31,58481	16,31612	81,422197	10,087761	13,48476	92,656259	17,11822	11,924136	95,555348	24,384279	17,978115
mmu-miR-30e-3p	MIMAT0000249	147,83175			79,492945	8,158508	10,905843	82,805121	7,267081	5,062072	79,046692	7,875622	5,806563
mmu-miR-683	MIMAT0003461	184,428154	45,55875	23,534794	78,278283	6,943846	9,28215	103,087475	27,549436	19,190267	89,480997	18,309928	13,499599
mmu-miR-7115-5p	MIMAT0028127	818,15613	679,286726	350,906759	329,258366	257,923929	344,77844	504,564508	429,026469	298,849407	605,684969	534,5139	394,088015
mmu-miR-466n-5p	MIMAT0014893	163,63637	24,766966	12,794149	72,532344			87,994242	12,456202	8,676687	76,684983	5,513914	4,065315
mmu-miR-6769b-3p	MIMAT0028041	172,611603	33,742199	17,430586	78,999942	7,665505	10,246823	94,987638	19,449598	13,548117	89,734455	18,563385	13,686469
mmu-miR-6926-3p	MIMAT0027753	164,775608	25,906204	13,382658	79,756493	8,422057	11,258139	91,276937	15,738897	10,963333	89,134804	17,963735	13,244357
mmu-miR-7024-5p	MIMAT0027952	304,464683	165,595279	85,54341	121,591971	50,257534	67,181492	165,257686	89,719646	62,496524	189,117692	117,946623	86,960041
mmu-miR-6906-3p	MIMAT0027713	160,232493	21,363089	11,03577	73,147113			82,733144	7,195104	5,011934	78,982889	7,811819	5,759522
mmu-miR-362-3p	MIMAT0004684	188,407168	49,537764	25,590278	77,243391	5,908954	7,898763	96,427929	20,889889	14,551389	95,654629	24,48356	18,051313
mmu-miR-6966-5p	MIMAT0027834	321,035998	182,166594	94,10384	112,315637	40,9812	54,781401	166,312973	90,774933	63,231612	181,821541	110,650472	81,580712
mmu-miR-202-3p	MIMAT0000235	156,288634	17,41923	8,998447	71,39843			88,035371	12,497332	8,705338	72,167582		
mmu-miR-18a-5p	MIMAT0000528	208,10079	69,231386	35,763633	101,05932	29,724884	39,73458	145,328713	69,790674	48,614487	104,260149	33,08908	24,396016
mmu-miR-7066-3p	MIMAT0028037	171,477414	32,60801	16,844685	79,870821	8,536384	11,410966	98,590899	23,05286	16,058062	86,4511	15,280031	11,265707
mmu-miR-1956	MIMAT0009428	166,052186	27,182782	14,042115	85,085852	13,751415	18,382131	97,876805	22,338766	15,560641	92,485175	21,314106	15,714528
mmu-miR-7677-5p	MIMAT0029868	313,53521	174,665806	90,229074	118,941223	47,606787	63,638119	176,046723	100,508684	70,011905	176,77066	105,599591	77,856784
mmu-miR-6537-3p	MIMAT0025582	154,540728	15,671324	8,095512	73,860372			86,682457	11,144418	7,76293	75,240236		
mmu-miR-6942-5p	MIMAT0027784	182,236572	43,367168	22,402664	82,320532	10,986095	14,685604	97,361585	21,823545	15,201751	98,731467	27,560398	20,319813
mmu-miR-19a-3p	MIMAT0000651	322,66726	183,797856	94,946519	118,282281	46,947845	62,757281	190,640332	115,102293	80,177458	167,099822	95,928753	70,726639
mmu-miR-6995-5p	MIMAT0027892	630,700013	491,830609	254,070451	225,39801	154,063573	205,943662	361,046477	285,508438	198,878236	397,607033	326,435964	240,675689
mmu-miR-7051-5p	MIMAT0028006	165,645159	26,775755	13,831852	77,284471	5,950034	7,953676	90,388544	14,850504	10,3445	85,058875	13,887805	10,239243
mmu-miR-7004-5p	MIMAT0027912	185,712908	46,843504	24,198474	84,431766	13,097329	17,507785	103,295499	27,757459	19,335171	98,240687	27,069618	19,957969
mmu-miR-6959-3p	MIMAT0027819	182,685678	43,816274	22,634664	84,358437	13,024	17,409762	100,291108	24,753068	17,242386	98,733778	27,562709	20,321517
mmu-miR-1929-3p	MIMAT0022729	175,285176	36,415772	18,811704	75,179793			85,023121	9,485081	6,607077	86,059664	14,888595	10,977108
mmu-miR-5127	MIMAT0020638	155,376117	16,506713	8,527058	72,178993			81,387817	5,849778	4,074813	76,386401	5,215332	3,845176
mmu-miR-7216-5p	MIMAT0028400	609,303375	470,433971	243,01735	200,553888	129,219451	172,733414	304,62309	229,085051	159,575077	378,392167	307,221098	226,508894
mmu-miR-93-5p	MIMAT0000540	472,456007	333,586603	172,324571	182,894003	111,559566	149,126656	317,766838	242,228799	168,730692	246,859663	175,688594	129,532214
mmu-miR-6970-5p	MIMAT0027842	265,458996	126,589592	65,393805	100,975111	29,640674	39,622013	130,6564	55,11836	38,39411	151,227935	80,056866	59,024566
mmu-miR-125a-5p	MIMAT0000135	220,657686	81,788282	42,25029	102,368661	31,034224	41,484834	130,35614	54,818101	38,184956	124,315371	53,144301	39,18239
mmu-miR-466c-5p	MIMAT0004877	156,774986	17,905582	9,249687	76,728238	5,393802	7,210136	87,075985	11,537945	8,037052	80,8941	9,72303	7,168625
mmu-miR-100-5p	MIMAT0000655	169,449149	30,579745	15,796921	84,836647	13,50221	18,049007	101,923262	26,385223	18,379305	88,513325	17,342255	12,78615
mmu-miR-326-3p	MIMAT0000559	166,123385	27,253981	14,078895	77,516138	6,181702	8,263357	93,253175	17,715135	12,339933	82,334049	11,16298	8,230275
mmu-miR-1306-3p	MIMAT0009411	140,760866			76,712032	5,377595	7,188472	74,263282			80,112806	8,941737	6,59259
mmu-miR-743a-3p	MIMAT0004238	181,695355	42,825951	22,123081	81,477271	10,142834	13,558379	103,101423	27,563383	19,199982	89,464868	18,293799	13,487707
mmu-miR-7681-5p	MIMAT0029882	178,630441	39,761037	20,539805	81,033154	9,698717	12,964708	98,238243	22,700204	15,81241	91,180893	20,009824	14,752903
mmu-miR-3544-5p	MIMAT0022353	160,158152	21,288748	10,997367	70,889752			82,491836	6,953797	4,843846	78,206449	7,03538	5,187066
mmu-miR-99a-5p	MIMAT0000131	172,939662	34,070258	17,600055	88,234991	16,900554	22,591725	104,14917	28,61113	19,929818	93,861265	22,690196	16,729096
mmu-miR-7075-5p	MIMAT0028056	396,899983	258,030579	133,293749	175,948707	104,614271	139,84257	243,793753	168,255713	117,202839	249,341698	178,170629	131,362177
mmu-miR-7238-5p	MIMAT0028444	215,657089	76,787685	39,667075	91,724019	20,389582	27,255665	109,74092	34,202881	23,824895	121,416236	50,245167	37,044908
mmu-miR-3569-3p	MIMAT0029855	154,287333	15,417929	7,964612	76,765262	5,430825	7,259627	86,933321	11,395282	7,937676	79,122508	7,951439	5,862461
mmu-miR-344f-5p	MIMAT0014931	153,229989	14,360585	7,418408	77,664478	6,330041	8,461648	88,221392	12,683352	8,834915	78,570469	7,3994	5,455452
mmu-miR-8095	MIMAT0031396	159,557037	20,687633	10,686843	73,658003			83,87069	8,332651	5,804322	76,335514	5,164445	3,807658
mmu-miR-6964-5p	MIMAT0027828	161,782531	22,913127	11,836491	77,093883	5,759446	7,698909	82,418407	6,880367	4,792697	88,419681	17,248612	12,717108
mmu-miR-466i-3p	MIMAT0005834	163,533547	24,664143	12,741033	76,709901	5,375464	7,185624	87,514579	11,97654	8,342566	84,025032	12,853962	9,477008
mmu-miR-669a-5p, mmu	MIMAT0003477, MIMA	169,319976	30,450572	15,730193	74,485589			89,781359	14,243319	9,92155	76,778683	5,607614	4,134398
mmu-miR-7656-3p	MIMAT0029819	160,176998	21,307594	11,007103	70,880184			81,60856	6,07052	4,228577	78,749632	7,578563	5,587545
mmu-miR-7078-5p	MIMAT0028062	560,318391	421,448987	217,712628	209,449334	138,114897	184,624354	316,324531	240,786491	167,726015	329,33407	258,163001	190,339193
mmu-miR-6408	MIMAT0025161	166,176359	27,306955	14,10626	76,38923	5,054793	6,756968	88,063538	12,525498	8,724958	84,484217	13,313147	9,815557
mmu-miR-20a-5p	MIMAT0000529	817,916557	679,047153	350,783	261,475687	190,14125	254,170305	556,970603	481,432563	335,354218	344,265814	273,094744	201,348114
mmu-miR-3084-3p	MIMAT0014877	197,313118	58,443714	30,190924	83,558387	12,22395	16,3403	109,610962	34,072922	23,734369	94,938773	23,767704	17,523524
mmu-miR-6963-3p	MIMAT0027827	185,779074	46,90967	24,232654	79,559718	8,225281	10,995101	100,785247	25,247208	17,586591	89,382618	18,211549	13,427066
mmu-miR-6920-3p	MIMAT0027741	170,754544	31,885118	16,471305	80,058028	8,723591	11,661214	95,999162	20,461123	14,252721	85,39943	14,228361	10,490329
mmu-miR-3065-3p	MIMAT0014837	187,656153	48,786749	25,202318	78,968512	7,634075	10,204809	96,126169	20,588129	14,34119	93,683687	22,512618	16,59817
mmu-miR-99b-5p	MIMAT0000132	168,358515	29,489111	15,233521	86,36278	15,028343	20,089058	97,449946	21,911906	15,263301	92,260713	21,089643	15,549036
mmu-miR-6901-5p	MIMAT0027702	293,592853	154,723449	79,927227	113,477007	42,14257	56,333856	161,432894	85,894854	59,832267	151,157699	79,98663	58,972783
mmu-miR-7652-5p	MIMAT0029810	187,382078	48,512674	25,060736	85,24419	13,909753	18,593789	103,955094	28,417054	19,794629	95,871813	24,700744	18,211439
mmu-miR-7686-3p	MIMAT0029899	400,622742	261,753338	135,216856	133,365303	62,030866	82,919431	224,093579	148,555539	103,480177	187,349553	116,178483	85,656421
mmu-miR-6969-5p	MIMAT0027840	707,422521	568,553118	293,703857	279,879543	208,545106	278,771561	416,731847	341,193807	237,667311	417,333776	346,162707	255,219881
mmu-miR-8116	MIMAT0031422	210,491476	71,622072	36,998617	98,617416	27,282979	36,470377	111,793317	36,255277	25,254545	122,404955	51,233886	37,773873
mmu-miR-698-5p	MIMAT0022930	174,970681	36,101277	18,649241	97,858202	26,523765	35,455502	100,098274	24,560234	17,108062	110,672682	39,501612	29,123867
mmu-miR-744-5p	MIMAT0004187	328,665999	189,796595	98,045355	127,55796	56,223523	75,156495	193,324945	117,786905	82,047494	160,16509	88,994021	65,613779

mmu-miR-487b-5p	MIMAT0017216	171,241879	32,372475	16,723012	76,366037	5,0316	6,725965	88,679309	13,141269	9,153888	85,81755	14,646481	10,798601
mmu-miR-6979-3p	MIMAT0027861	162,699385	23,829981	12,31012	77,462478	6,128041	8,191626	82,974709	7,436669	5,180203	87,769995	16,598926	12,238106
mmu-miR-466m-5p, mmu	MIMAT0014882, MIMA	160,415295	21,545891	11,130202	79,737357	8,40292	11,232558	89,539067	14,001028	9,752775	83,610858	12,439789	9,171645
mmu-miR-19b-3p	MIMAT0000513	870,580858	731,711454	377,988388	304,505153	233,170716	311,689715	605,42887	529,890831	369,109069	359,65403	288,482961	212,693585
mmu-miR-761	MIMAT0003893	159,782797	20,913393	10,803466	74,205199			83,367721	7,829681	5,453965	76,13052	4,95945	3,656519
mmu-miR-467c-3p	MIMAT0017275	155,334039	16,464635	8,505321	69,677303			85,82052	10,282481	7,162526	75,619602		
mmu-miR-3569-5p	MIMAT0029854	200,327811	61,458407	31,748258	102,637352	31,302915	41,844006	122,278048	46,740009	32,557954	111,02542	39,854351	29,383936
mmu-miR-7015-5p	MIMAT0027934	233,460175	94,590771	48,863815	108,777291	37,442855	50,051537	138,603549	63,065509	43,929901	124,292752	53,121683	39,165714
mmu-miR-299a-3p	MIMAT0004577	154,888497	16,019093	8,275162	73,706889			85,506978	9,968938	6,94412	72,397626		
mmu-miR-1968-3p	MIMAT0017350	152,724144	13,85474	7,157098	73,698571			84,12864	8,5906	5,984003	74,492172		
mmu-miR-25-3p	MIMAT0000652	377,955163	239,085759	123,507211	126,369872	55,035435	73,568325	207,954614	132,416575	92,238167	169,42909	98,258021	72,443969
mmu-miR-7043-5p	MIMAT0027990	164,363556	25,494152	13,1698	80,215854	8,881417	11,872187	85,696147	10,158107	7,07589	89,930599	18,75953	13,831082
mmu-miR-196b-3p	MIMAT0017170	161,795085	22,925681	11,842976	75,363906			84,194209	8,65617	6,029677	76,300882	5,129813	3,782124
mmu-miR-7682-3p	MIMAT0029885	155,148127	16,278723	8,409283	77,338582	6,004145	8,026009	81,648964	6,110924	4,256721	83,773942	12,602872	9,291884
mmu-miR-7001-5p	MIMAT0027904	210,077333	71,207929	36,784678	85,462315	14,127878	18,885366	106,994933	31,456893	21,912107	103,500684	32,329615	23,836076
mmu-miR-212-3p	MIMAT0000659	209,232881	70,363477	36,34845	100,488664	29,154227	38,971758	120,835125	45,297085	31,552848	112,21671	41,04564	30,262253
mmu-miR-547-5p	MIMAT0017210	151,410917	12,541513	6,47871	76,391811	5,057375	6,760418	85,774015	10,235976	7,130132	76,229801	5,058731	3,729717
mmu-miR-6961-3p	MIMAT0027823	154,15253	15,283126	7,894976	69,627085			84,79458	9,256541	6,447881	74,930373		
mmu-miR-6349	MIMAT0025092	904,974935	766,105531	395,755722	277,833751	206,499314	276,036859	423,228016	347,689977	242,192384	482,44224	411,271171	303,223245
mmu-miR-540-3p	MIMAT0003167	171,745203	32,875799	16,98302	81,392611	10,058174	13,445211	90,974661	15,436621	10,752775	90,068634	18,897564	13,932853
mmu-miR-7118-5p	MIMAT0028133	3407,884343	3269,014939	1688,711693	980,13112	908,796683	1214,829132	1492,872267	1417,334228	987,280563	1906,912268	1835,741199	1353,460789
mmu-miR-1843b-3p	MIMAT0019346	233,384987	94,515583	48,824974	102,772661	31,438224	42,024878	131,832654	56,294614	39,21346	117,215188	46,044118	33,947546
mmu-miR-7679-5p	MIMAT0029872	149,251605			77,069809	5,735372	7,666728	84,388544	8,850504	6,165046	73,482356		
mmu-miR-6945-3p	MIMAT0027791	161,672421	22,803017	11,77961	76,510995	5,176558	6,919737	87,201085	11,663045	8,124194	80,542263	9,371194	6,909222
mmu-miR-7676-3p	MIMAT0029861	176,018647	37,149243	19,190601	81,887745	10,553308	14,107079	94,844248	19,306208	13,448235	89,205034	18,033965	13,296136
mmu-miR-3102-3p	MIMAT0014936	210,322504	71,4531	36,911329	94,664602	23,330165	31,186474	113,205651	37,667612	26,238342	109,627438	38,456369	28,353227
mmu-miR-1199-5p	MIMAT0005860	155,502811	16,633407	8,592505	78,01791	6,683474	8,934098	87,446057	11,908018	8,294836	78,952202	7,781133	5,736897
mmu-miR-7041-5p	MIMAT0027986	154,825473	15,956069	8,242606	78,314139	6,979702	9,330079	83,814629	8,276589	5,765271	82,429271	11,258202	8,300481
mmu-miR-6966-3p	MIMAT0027835	160,789438	21,920034	11,323478	71,705639			82,934821	7,396782	5,152418	76,470399	5,299329	3,907106
mmu-let-7b-5p	MIMAT0000522	677,730814	538,861411	278,36568	230,119588	158,785151	212,255206	381,726541	306,188501	213,283465	313,650816	242,479747	178,776197
mmu-miR-503-3p	MIMAT0004790	155,147505	16,278101	8,408961	74,964762			77,308755			80,264609	9,093539	6,704512
mmu-miR-1949	MIMAT0009416	164,567683	25,698279	13,275248	78,414923	7,080486	9,464802	87,758826	12,220786	8,512702	84,163686	12,992617	9,579236
mmu-miR-7681-3p	MIMAT0029883	184,81233	45,942926	23,733252	87,189117	15,85468	21,193659	98,721116	23,183077	16,148768	97,685378	26,514309	19,548549
mmu-miR-8091	MIMAT0031392	157,917359	19,047955	9,839816	77,466903	6,132466	8,197541	86,008705	10,470666	7,293611	80,388882	9,217813	6,796137
mmu-miR-3970	MIMAT0019355	160,170713	21,301309	11,003856	79,773949	8,439512	11,281473	90,190544	14,652505	10,206579	80,86347	9,692401	7,146042
mmu-miR-467b-3p	MIMAT0003478	162,47554	23,606136	12,194486	79,437072	8,102635	10,831154	88,435775	12,897735	8,984249	83,295295	12,124226	8,938985
mmu-miR-7680-3p	MIMAT0029875	168,655208	29,785804	15,386787	81,80831	10,473873	14,000894	90,457066	14,919026	10,392231	88,044437	16,873367	12,440447
mmu-miR-7015-3p	MIMAT0027935	157,490258	18,620854	9,619183	76,096014	4,761577	6,365013	86,383636	10,845597	7,554779	77,712513	6,541443	4,822895
mmu-miR-6927-3p	MIMAT0027755	203,5279	64,658496	33,401364	90,448321	19,113884	25,550383	107,129698	31,585658	22,001802	103,202835	32,031766	23,616477
mmu-miR-6960-5p	MIMAT0027820	159,565196	20,695792	10,691057	80,473296	9,138859	12,216321	89,996468	14,458428	10,07139	81,513155	10,342086	7,625044
mmu-miR-1981-3p	MIMAT0017351	155,729168	16,859764	8,709437	76,743913	5,409476	7,231088	84,106623	8,568583	5,968667	79,747208	8,576138	6,323041
mmu-miR-467d-3p	MIMAT0004887	157,281073	18,411669	9,511122	86,276439	14,942003	19,973642	94,684168	19,146128	13,336727	83,913561	12,742492	9,394823
mmu-miR-1951	MIMAT0009422	193,477761	54,608358	28,209651	86,436991	15,102554	20,188258	104,931318	29,393279	20,474643	93,934714	22,763645	16,783249
mmu-miR-666-5p	MIMAT0003737	167,050312	28,180909	14,557728	77,129144	5,794708	7,746045	87,886816	12,348776	8,601857	82,770672	11,599603	8,55219
mmu-miR-192-5p	MIMAT0000517	156,166335	17,296931	8,93527	79,928825	8,594389	11,488503	84,865783	9,327744	6,497479	83,562401	12,391332	9,135918
mmu-miR-7242-3p	MIMAT0028453	171,963774	33,09437	17,09593	83,981974	12,647537	16,906527	98,359162	22,821122	15,896639	84,86595	13,694881	10,097003
mmu-miR-8100	MIMAT0031403	256,797992	117,928589	60,919693	113,658539	42,324102	56,576518	125,600645	50,062605	34,872394	145,222798	74,051729	54,597081
mmu-miR-7668-3p	MIMAT0029843	224,858438	85,989034	44,420319	94,668945	23,334509	31,19228	111,74092	36,202881	25,218047	114,793798	43,622729	32,162297
mmu-miR-5131	MIMAT0020642	296,092447	157,223043	81,21847	114,687244	43,352807	57,951634	151,340658	75,802618	52,802261	142,513369	71,3423	52,599465
mmu-miR-6914-5p	MIMAT0027728	6297,220247	6158,350843	3181,288331	2451,217491	2379,883054	3181,296013	3652,138005	3576,599965	2491,372578	3212,077418	3140,906349	2315,736874
mmu-miR-7070-3p	MIMAT0028047	182,906994	44,03759	22,748992	78,035716	6,701279	8,957899	89,239018	13,700978	9,543768	90,714317	19,543247	14,408904
mmu-miR-6986-5p	MIMAT0027874	295,777918	156,908514	81,05599	114,859576	43,525139	58,181998	144,334089	68,796049	47,921655	147,922751	76,751682	56,58771
mmu-miR-7215-3p	MIMAT0028399	176,087939	37,218535	19,226396	78,117282	6,782845	9,066932	86,38927	10,85123	7,558703	89,690846	18,519777	13,654317
mmu-miR-6967-5p	MIMAT0027836	479,195058	340,325654	175,805838	183,733452	112,399015	150,248785	261,418209	185,880169	129,479607	226,266896	155,095827	114,349517
mmu-let-7c-5p	MIMAT0000523	894,31166	755,442256	390,247275	300,649149	229,314712	306,535221	489,146737	413,608697	288,109762	384,734294	313,563225	231,184837
mmu-miR-8107	MIMAT0031412	177,85028	38,980877	20,136788	87,503313	16,168876	21,61366	99,976839	24,438799	17,023474	90,279272	19,108203	14,088153
mmu-miR-490-3p	MIMAT0003780	152,424108	13,554704	7,002106	75,128776			83,009947	7,471908	5,204749	70,958523		
mmu-miR-681	MIMAT0003458	151,811668	12,942264	6,68573	70,044197			82,6514	7,11336	4,954994	73,485626		
mmu-miR-3068-3p	MIMAT0014843	202,102075	63,232671	32,66481	90,097101	18,762664	25,080892	109,94967	34,411631	23,970305	96,352045	25,180976	18,565505
mmu-miR-7217-3p	MIMAT0028403	172,880855	34,011451	17,569263	77,680602	6,346165	8,483203	89,534886	13,996847	9,749863	83,953104	12,782035	9,423977
mmu-miR-6344	MIMAT0025087	155,460757	16,591353	8,570781	70,594324			84,555905	9,017866	6,281626	73,245936		
mmu-miR-6364	MIMAT0025108	174,89197	36,022566	18,608581	80,187149	8,852712	11,833815	88,111223	12,573184	8,758174	89,537521	18,366452	13,541273
mmu-let-7k	MIMAT0025580	189,502213	50,632809	26,155958	83,028647	11,69421	15,632173	94,885119	19,34708	13,476705	94,332583	23,161514	17,076591
mmu-miR-5098	MIMAT0020605	154,527545	15,658141	8,088701	79,235953	7,901516	10,56231	86,66044	11,122401	7,747594	79,154773	7,983704	5,886249
mmu-miR-6912-5p	MIMAT0027724	371,07704	232,207636	119,954102	202,000505	130,666068	174,667171	226,76315	151,22511	105,339735	220,404324	149,233254	110,027142
mmu-miR-455-3p	MIMAT0003742	156,624471	17,755068	9,171934	71,043316			85,088026	9,549986	6,652288	72,222472		
mmu-miR-5120	MIMAT0020628	190,234648	51,365244	26,53432	79,656959	8,322522	11,125087	99,561036	24,022997	16,733835	85,446252	14,275183	10,52485
mmu-miR-6939-5p	MIMAT0027778	663,53487	524,665466	271,032322	219,035522	147,701085	197,438639	290,043677	214,505637	149,419412	326,456708	255,285639	188,217763
mmu-miR-145b	MIMAT0025105	190,413469	51,544066	26,626696	85,91645	14,582014	19,492429	97,223312	21,685272	15,105433	95,740205	24,569136	18,114406
mmu-miR-7034-3p	MIMAT0027973	154,194228	15,324824	7,916516	82,069727	10,73529	14,350342	89,554467	14,016427	9,763502	79,653621	8,482551	6,254041
mmu-miR-5621-3p	MIMAT0022370	175,871897	37,002493	19,114792	81,074071	9,739634	13,019404	92,546879	17,008839	11,847944	86,438973	15,267904	11,256766
mmu-miR-466j	MIMAT0005848	168,122738	29,253334	15,111723	83,90387	12,569433	16,802123	94,259583	18,721543	13,040972	84,3147	13,143631	9,690576
mmu-miR-338-5p	MIMAT0004647	152,300565	13,431161	6,938286	70,635772			80,515292			77,86347	6,692401	4,934193
mmu-miR-6912-3p	MIMAT0027725	162,099539	23,230135	12,000251	82,899526	11,565089	15,459572	89,422811	13,884772	9,671795	84,464079	13,293009	9,80071
mmu-miR-7067-5p	MIMAT0028038	711,297502	572,428098	295,7056	227,125694	155,791257	208,253134	307,105901	231,567862	161,304542	336,743333	265,572263	195,801917
mmu-miR-7000-5p	MIMAT0027902	182,476137	43,606733	22,526419	79,803167	8,46873	11,32053	91,6837	16,14566	11,246674	88,416524	17,245455	12,714781
mmu-miR-1982-3p	MIMAT0009460	172,388024	33,51862	17,315089	77,132157	5,79772	7,750071	87,637159	12,099155	8,427977	83,76018	12,589111	9,281737
mmu-miR-1945	MIMAT0009410	172,095742	33,226338	17,164102	78,608236	7,273799	9,723212	85,245363	9,707324	6,761886	87,746581	16,575512	12,220843
mmu-miR-106b-5p	MIMAT0000386	398,88932	260,019916	134,321403	195,037404	123,702967	165,359284	259,788749	184,25071	128,344565	182,843314	111,672245	82,334048
mmu-miR-184-5p	MIMAT0022690	175,076663	36,207259	18,703989	78,268265	6,933828	9,268757	94,180988	18,642948	12,986225	80,218576	9,047507	6,670573
mmu-miR-7672-3p	MIMAT0029851	157,696292	18,826888	9,725617	67,874959			85,348398	9,810358	6,833657	74,635794		
mmu-miR-1941-3p	MIMAT0009406	150,986958	12,117554	6,259701	72,322907			78,696453			77,127363	5,956294	4,391474
mmu-miR-679-5p	MIMAT0003455	154,627606	15,758202	8,140391	78,783149	7,448712	9,957026	83,818294	8,280254	5,767824	80,497082	9,326013	6,875911
mmu-miR-187-3p	MIMAT0000216	154,20237	15,332966	7,920722	72,876209			83,471271	7,933232	5,526096	75,283055		
mmu-miR-302c-5p	MIMAT0003375	157,722634	18,85323	9,739224	75,227348			85,267429	9,729389	6,777256	70,703486		
mmu-miR-543-5p	MIMAT0017207	155,732699	16,863296	8,711262	72,406317			77,449512			79,384823	8,213754	6,055861
mmu-miR-3058-3p	MIMAT0014814	381,025666	242,156262	125,093375	148,856362	77,521925	103,627021	184,758604	109,220565	76,08039	183,526962	112,355892	82,83809
mmu-miR-485-5p	MIMAT0003128	164,859957	25,990553	13,426231	74,928907			88,862844	13,324805	9,281735	75,635737		
mmu-miR-6913-3p	MIMAT0027727	165,313699	26,444295	13,660626	84,473215	13,138778	17,563191	93,221601	17,683562	12,317939	83,668115	12,497046	9,213859
mmu-miR-505-5p	MIMAT0017259	202,456767	63,587363	32,848037	81,760837	10,4264	13,937435	94,556177	19,018138	13,247572	96,937984	25,766915	18,997508
mmu-miR-3082-3p	MIMAT0014873	154,309329	15,439926	7,975975	73,063334			80,594145			78,611591	7,440521	5,48577
mmu-miR-7042-3p	MIMAT0027989	240,695088	101,825684	52,601235	75,194586			112,614172	37,076133	25,826333	84,988645	13,817576	10,187464
mmu-miR-467b-5p	MIMAT0005448	151,056233	12,186829	6,295487	77,249947	5,91551	7,907526	84,407866	8,869827	6,178506	75,941711	4,770641	3,517313
mmu-miR-16-5p	MIMAT0000527	2603,803834	2464,93443	1273,338811	1142,697693	1071,363256	1432,139133	1777,438997	1701,900958	1185,502829	963,301322	892,130253	657,752475
mmu-miR-6937-5p	MIMAT0027774	592,140564	453,27116	234,151365	196,920351	125,585915	167,876303	254,090509	178,552469	124,375309	273,706902	202,535833	149,326228
mmu-miR-6972-5p	MIMAT0027846	476,851434	337,98203	174,595166	156,020663	84,686226	113,203862	208,078376	132,540336	92,324376	211,362074	140,191005	103,360445

mmu-miR-6410	MIMAT0025163	173,211465	34,342061	17,740464	89,126769	17,792332	23,783805	90,93968	15,401641	10,728409	94,879825	23,708755	17,480062
mmu-miR-15a-5p	MIMAT0000526	607,161649	468,292245	241,910974	202,489164	131,154728	175,320384	283,798739	208,260699	145,06934	257,903553	186,732484	137,674687
mmu-miR-674-3p	MIMAT0003741	162,806369	23,936965	12,365386	82,771287	11,43685	15,288148	92,981278	17,443239	12,150536	79,732651	8,561581	6,312308
mmu-miR-5122	MIMAT0020630	385,624923	246,755519	127,469264	164,412738	93,078301	124,421923	201,468999	125,930959	87,720445	178,852171	107,681102	79,391446
mmu-miR-423-3p	MIMAT0003454	224,008348	85,138944	43,981179	97,320287	25,98585	34,736447	121,150268	45,612228	31,772369	98,730621	27,559552	20,319189
mmu-miR-5112	MIMAT0020620	387,740679	248,871275	128,562225	152,168202	80,833765	108,054106	186,987417	111,449377	77,632926	177,644576	106,473507	78,501107
mmu-miR-6397	MIMAT0025149	160,15942	21,290016	10,998022	70,287646			76,586543			80,997446	9,826376	7,24482
mmu-miR-7059-5p	MIMAT0028022	996,008628	857,139224	442,782018	305,097528	233,763091	312,481569	458,692874	383,154834	266,896342	378,999096	307,828027	226,956373
mmu-miR-101b-3p	MIMAT0000616	155,300565	16,431161	8,488029	79,333275	7,998838	10,692405	81,546913	6,008874	4,185635	82,444673	11,273604	8,311837
mmu-miR-7672-5p	MIMAT0029850	525,96307	387,093666	199,965314	263,984077	192,649641	257,523382	255,941289	180,40325	125,664518	304,198064	233,026995	171,806843
mmu-miR-222-3p	MIMAT0000670	151,810416	12,941012	6,685084	83,240297	11,90586	15,915095	86,820262	11,282223	7,858922	80,303306	9,132237	6,733043
mmu-miR-350-3p	MIMAT0000605	202,782588	63,913184	33,01635	90,054259	18,719822	25,023622	101,811384	26,273344	18,301373	97,005	25,833931	19,046918
mmu-miR-103-1-5p	MIMAT0017024	177,226547	38,357143	19,81458	75,994799			87,938696	12,400657	8,637996	76,714091	5,543022	4,086776
mmu-miR-654-3p	MIMAT0004898	153,482737	14,613333	7,548973	74,684208			82,466154	6,928115	4,825956	75,262072		
mmu-miR-3066-3p	MIMAT0014839	182,203963	43,334549	22,385814	74,32342			85,436033	9,897993	6,894701	81,196803	10,025734	7,391803
mmu-miR-3076-5p	MIMAT0014860	300,004681	161,135277	83,239456	137,660631	66,326194	88,661187	155,152215	79,614175	55,457299	144,55134	73,380271	54,102026
mmu-miR-344d-3-5p	MIMAT0014807	143,186707			78,732501	7,398064	9,889322	78,433855			79,693953	8,522884	6,283777
mmu-miR-411-3p	MIMAT0001093	149,856492			82,927863	11,593426	15,49745	81,748333	6,210294	4,32594	78,628515	7,457446	5,498248
mmu-miR-412-3p	MIMAT0001094	159,838001	20,968598	10,831983	78,544044	7,209607	9,637404	82,871158	7,333119	5,108072	81,752902	10,581832	7,801805
mmu-miR-449c-5p	MIMAT0003460	167,059077	28,189673	14,562255	75,7618			81,793338	6,255298	4,357289	77,673759	6,502689	4,794322
mmu-miR-365-1-5p	MIMAT0017077	482,105419	343,236015	177,309276	157,367027	86,03259	115,003607	189,922301	114,384262	79,677296	211,748656	140,577587	103,645466
mmu-miR-7071-5p	MIMAT0028048	165,980374	27,11097	14,005018	79,033154	7,698717	10,291219	86,875807	11,337768	7,897613	80,923997	9,752927	7,190668
mmu-miR-7225-5p	MIMAT0028418	192,055643	53,18624	27,475012	87,982773	16,648336	22,254575	102,810461	27,272422	18,997306	87,171867	16,000798	11,797116
mmu-miR-344i	MIMAT0022503	175,615384	36,74598	18,982283	86,59666	15,262224	20,401696	94,701067	19,163028	13,348499	86,064518	14,893449	10,980687
mmu-miR-1954	MIMAT0009425	158,086364	19,21696	9,92712	73,961668			84,332272	8,794233	6,125849	75,354863		
mmu-miR-143-3p	MIMAT0000247	731,824971	592,955567	306,309704	274,240312	202,905876	271,23335	353,77319	278,235151	193,811841	291,096134	219,925064	162,147012
mmu-miR-8092	MIMAT0031393	178,672147	39,802743	20,561349	81,858077	10,523641	14,067421	94,821456	19,283417	13,432359	81,87554	10,704471	7,892224
mmu-miR-6406	MIMAT0025159	1031,028894	892,15949	460,872829	344,885533	273,551096	365,667972	430,049488	354,511448	246,944055	421,75759	350,586521	258,481484
mmu-miR-7218-3p	MIMAT0028405	183,995157	45,125753	23,311116	90,952593	19,618156	26,224466	97,953383	22,415344	15,613984	90,890993	19,719924	14,539165
mmu-let-7f-5p	MIMAT0000525	1370,771734	1231,90233	636,377597	391,265031	319,930594	427,66552	532,067299	456,529259	318,007182	517,846248	446,675179	329,326019
mmu-miR-3073a-5p	MIMAT0014854	179,074126	40,204722	20,769004	84,842322	13,507885	18,056593	96,219179	20,681139	14,405979	83,532561	12,361491	9,113917
mmu-miR-5617-3p	MIMAT0022362	163,199244	24,32984	12,568338	84,17963	12,845193	17,170743	93,292336	17,754297	12,367212	78,792451	7,621382	5,619115
mmu-miR-34c-3p	MIMAT0004580	173,127755	34,258351	17,69722	78,947162	7,612726	10,176271	85,91203	10,373991	7,22627	83,979732	12,808663	9,443609
mmu-miR-7a-5p	MIMAT0000677	167,650167	28,780763	14,867601	86,642453	15,308016	20,462909	91,623505	16,085466	11,204744	84,577804	13,406735	9,884558
mmu-miR-3472	MIMAT0015643	218,744589	79,875185	41,26202	97,789748	26,455311	35,363996	106,413933	30,875893	21,507397	103,984918	32,813849	24,193093
mmu-miR-98-5p	MIMAT0000545	239,01149	100,142086	51,73152	114,270213	42,935776	57,394171	131,71614	56,1781	39,132299	106,157705	34,986636	25,795052
mmu-let-7d-5p	MIMAT0000383	1028,267596	889,398192	459,446394	355,307357	283,97292	379,599291	456,600026	381,061986	265,438515	384,752127	313,581058	231,197985
mmu-miR-1943-3p	MIMAT0017342	247,86818	108,998776	56,30672	106,356798	35,022362	46,815956	133,538692	58,000652	40,401844	99,132549	27,96148	20,615524
mmu-miR-199b-5p	MIMAT0000672	198,05163	59,182226	30,572426	87,85189	16,517453	22,079618	97,77692	22,23888	15,491063	92,405243	21,234174	15,655595
mmu-miR-125b-1-3p	MIMAT0004669	180,834577	41,965173	21,67842	86,228885	14,894448	19,910074	95,318745	19,780705	13,778758	85,752169	14,581099	10,750397
mmu-miR-6929-5p	MIMAT0027758	549,503383	410,633979	212,125798	190,411818	119,077381	159,176055	226,565653	151,027614	105,202164	224,514766	153,343697	113,057702
mmu-miR-7057-5p	MIMAT0028018	719,258905	580,389501	299,818311	274,00137	202,666934	270,913945	309,075522	233,537482	162,676532	304,239919	233,068849	171,837702
mmu-miR-330-3p	MIMAT0000569	186,809859	47,940455	24,765138	82,269965	10,935528	14,618009	93,217937	17,679897	12,315386	85,675506	14,504437	10,693875
mmu-miR-1943-5p	MIMAT0009408	405,628404	266,759001	137,802687	143,446256	72,111819	96,395091	182,324931	106,786892	74,385152	155,642722	84,471653	62,279514
mmu-let-7a-5p	MIMAT0000521	1155,868492	1016,999088	525,362619	387,794258	316,459821	423,025983	502,623522	427,085483	297,497363	418,235397	347,064328	255,884631
mmu-miR-138-5p	MIMAT0000150	185,071986	46,202582	23,867386	84,677427	13,34299	17,836171	97,125911	21,587871	15,037586	83,763393	12,592324	9,284107
mmu-miR-6388	MIMAT0025135	228,4288	89,559396	46,264701	102,385135	31,050698	41,506855	113,140808	37,602768	26,193174	105,049511	33,878441	24,977999
mmu-miR-221-3p	MIMAT0000669	205,583411	66,714007	34,463203	95,07817	23,743733	31,739309	109,463132	33,925093	23,631395	91,38984	20,218771	14,906956
mmu-miR-3094-5p	MIMAT0014909	155,887866	17,018462	8,791418	73,575556			82,876066	7,338026	5,111491	72,62688		
mmu-miR-1962	MIMAT0009435	173,195811	34,326407	17,732377	86,554781	15,220344	20,345714	87,078728	11,540688	8,038963	90,078336	18,907267	13,940007
mmu-let-7a-1-3p, mmu-le	MIMAT0004620, MIMA	151,29239	12,422986	6,417481	71,58078			80,819794	5,281755	3,679142	74,320964		
mmu-miR-15a-3p	MIMAT0004624	151,070685	12,201281	6,302952	71,275865			80,704719	5,166679	3,598983	73,902055		
mmu-miR-30d-5p	MIMAT0000515	425,439399	286,569995	148,036675	194,306096	122,971659	164,381711	217,65282	142,11478	98,993701	177,333198	106,162129	78,271533
mmu-miR-1198-5p	MIMAT0005859	317,143878	178,274474	92,093244	114,456457	43,12202	57,643131	136,094736	60,556696	42,182322	129,187997	58,016927	42,774895
mmu-miR-1231-5p	MIMAT0022357	531,769065	392,899661	202,964582	205,76935	134,434913	179,705154	234,060002	158,521963	110,422545	214,990883	143,819813	106,035904
mmu-miR-216c-3p	MIMAT0029889	154,333433	15,464029	7,988427	72,819004			81,986656	6,450617	4,493343	73,901265		
mmu-miR-6360	MIMAT0025103	205,803897	66,934493	34,577102	97,170267	25,83583	34,535909	93,97027	18,43223	12,839443	106,482125	35,311056	26,034241
mmu-miR-1970	MIMAT0009444	148,758321			86,289102	14,954665	19,990568	83,89545	8,357411	5,821569	78,522012	7,350943	5,419725
mmu-miR-7038-3p	MIMAT0027981	155,38329	16,513886	8,530763	84,770324	13,435887	17,96035	88,814839	13,276799	9,248296	78,819861	7,648792	5,639324
mmu-miR-7676-5p	MIMAT0029860	148,632258			78,604261	7,269824	9,717898	83,336921	7,798882	5,432511	73,785849		
mmu-miR-6907-3p	MIMAT0027715	159,377386	20,507982	10,594038	77,596823	6,262387	8,371212	82,249286	6,711247	4,674891	79,153195	7,982125	5,885085
mmu-miR-543-3p	MIMAT0003168	151,548273	12,678869	6,549666	73,772331			80,767656	5,229616	3,642824	67,671109		
mmu-miR-6955-3p	MIMAT0027811	201,433915	62,564511	32,319651	78,70062	7,366183	9,846706	89,292336	13,754297	9,580909	89,787766	18,616697	13,725774
mmu-miR-7029-3p	MIMAT0027963	156,601861	17,732457	9,160254	72,019754			75,951708			78,025652	6,854582	5,053767
mmu-miR-215-3p	MIMAT0017169	157,733571	18,864167	9,744874	75,210342			78,239262			78,450142	7,279072	5,366736
mmu-miR-3962	MIMAT0019340	176,953725	38,084321	19,673645	91,957899	20,623462	27,568304	96,530963	20,992923	14,62316	86,52946	15,358391	11,323481
mmu-miR-758-5p	MIMAT0017235	175,170688	36,301284	18,752561	77,124801	5,790364	7,740238	83,935744	8,397704	5,849637	82,953048	11,781979	8,686653
mmu-miR-26a-5p	MIMAT0000533	1137,503625	998,634221	515,875674	404,52606	333,191623	445,39213	500,951026	425,412986	296,332343	375,423035	304,251966	224,319804
mmu-let-7e-5p	MIMAT0000524	546,960267	408,090863	210,812072	207,335969	136,001532	181,799325	232,239921	156,701882	109,154721	211,337752	140,166683	103,342513
mmu-miR-3068-5p	MIMAT0014842	157,60939	18,739986	9,680724	77,449816	6,115379	8,1747	89,410803	13,872263	9,663081	74,806833		
mmu-miR-7074-5p	MIMAT0028054	339,848147	200,978743	103,821842	126,998552	55,664115	74,40871	141,521289	65,98325	45,962328	138,991965	67,820896	50,003194
mmu-miR-6392-3p	MIMAT0025142	1072,667111	933,797707	482,382349	381,592872	310,258435	414,736312	418,827341	343,289302	239,126981	400,615327	329,444257	242,893652
mmu-miR-148a-3p	MIMAT0000516	253,345147	114,475743	59,136018	110,471782	39,137345	52,316638	124,429814	48,891775	34,056821	105,527312	34,356242	25,330274
mmu-miR-30a-5p	MIMAT0000128	424,449665	285,580261	147,525397	185,691572	114,357135	152,866292	203,634196	128,096157	89,228668	166,580723	95,409654	70,343917
mmu-miR-140-3p	MIMAT0000152	251,29117	112,421766	58,074972	108,410315	37,075879	49,560983	117,987345	42,449305	29,569154	108,461987	37,290917	27,49396
mmu-miR-6385	MIMAT0025131	465,201609	326,332205	168,577085	209,586937	138,2525	184,808294	210,140538	134,602499	93,760828	197,496614	126,325545	93,137677
mmu-miR-6999-3p	MIMAT0027901	156,938304	18,0689	9,334054	73,351325			82,594145	7,056105	4,915131	74,299191		
mmu-miR-3085-5p	MIMAT0014878	509,55896	370,689556	191,491259	217,407292	146,072855	195,262111	238,799582	163,261543	113,724021	192,6184	121,447331	89,541053
mmu-miR-3618-5p	MIMAT0035716	159,422177	20,552773	10,617176	71,524989			72,822265			78,702021	7,530952	5,552443
mmu-miR-1843a-3p	MIMAT0014806	176,975722	38,106318	19,685008	82,38208	11,047643	14,767878	87,421359	11,88332	8,277632	84,316278	13,145209	9,691739
mmu-miR-27a-3p	MIMAT0000537	522,495539	383,626135	198,174053	179,429499	108,095062	144,495499	216,819001	141,280962	98,412883	179,125034	107,953964	79,592623
mmu-miR-32-5p	MIMAT0000654	172,679054	33,80965	17,46543	83,888995	12,554558	16,782239	90,097534	14,559495	10,14179	81,534139	10,36307	7,640515
mmu-miR-466m-3p	MIMAT0014883	171,130275	32,260871	16,66536	77,152256	5,817819	7,776939	84,773289	9,23525	6,43305	79,65441	8,483341	6,254623
mmu-miR-6409	MIMAT0025162	248,401893	109,532489	56,582426	95,859533	24,525096	32,783792	104,158936	28,620897	19,936621	106,583892	35,412823	26,109273
mmu-miR-6351	MIMAT0025094	7720,581605	7581,712201	3916,570064	2375,632348	2304,297911	3080,257975	2831,25677	2755,71873	1919,566667	2356,62199	2285,450921	1685,024125
mmu-miR-7035-3p	MIMAT0027975	155,77123	16,901826	8,731166	77,578568	6,244131	8,346809	80,916469	5,37843	3,746483	78,01516	6,84409	5,046032
mmu-miR-195a-5p	MIMAT0000225	516,948427	378,079023	195,308519	211,42526	140,090823	187,265663	222,331418	146,793378	102,252698	198,846076	127,675007	94,132613
mmu-miR-30e-5p	MIMAT0000248	480,186617	341,317213	176,318059	177,982657	106,64822	142,561441	209,989866	134,451827	93,655874	162,257769	91,0867	67,156676
mmu-miR-1839-3p	MIMAT0009457	276,322262	137,452858	71,005564	116,76961	45,435173	60,735226	114,679484	39,141444	27,264978	124,273347	53,102278	39,151407
mmu-miR-199a-5p	MIMAT0000229	210,008639	71,139235	36,749192	95,493151	24,158714	32,294033	92,354241	16,816202	11,713757	102,384422	31,213353	23,013075
mmu-miR-153-5p	MIMAT0016992	154,757425	15,888021	8,207453	75,476471			81,420891	5,882852	4,097851	66,37574		
mmu-miR-1933-5p	MIMAT0009396	152,007031	13,137627	6,786651	76,294121	4,959684	6,629831	85,133547	9,595507	6,683997	75,316109		
mmu-miR-101c	MIMAT0019349	165,745841	26,876438	13,883863	79,39048	8,056043	10,768873	84,41521	8,87717	6,183621	79,332358	8,161288	6,017179
mmu-miR-301a-3p	MIMAT0000379	211,033745	72,164341	37,278743	92,525913	21,191476	28,327592	102,830252	27,292212	19,011092	89,412402	18,241333	13,449025
mmu-miR-7219-5p	MIMAT0028406	321,999325	183,129921	94,601476	131,051332	59,716895	79,826242	138,359819	62,821779	43,760124	128,544569	57,373499	42,300506
mmu-miR-1931	MIMAT0009394	336,376819	197,507415	102,028619	135,335779	64,001342	85,553454	146,676462	71,138423	49,553296	128,861766	57,690697	42,534371

mmu-miR-106b-3p	MIMAT0004582	149,937101			76,902701	5,568265	7,443348	79,052516			76,115963	4,944893	3,645786
mmu-miR-7a-1-3p	MIMAT0004670	141,601927			78,561562	7,227125	9,660821	76,347462			77,571202	6,400132	4,718709
mmu-miR-7674-5p	MIMAT0029856	215,311849	76,442445	39,488731	90,746168	19,411731	25,948528	95,526314	19,988274	13,923346	95,609505	24,438436	18,018043
mmu-miR-467e-3p	MIMAT0005294	153,119645	14,250242	7,361407	66,377612			80,167801			76,005457	4,834388	3,564312
mmu-miR-7228-5p	MIMAT0028424	189,064432	50,195028	25,929809	89,175573	17,841137	23,849044	90,274256	14,736217	10,26489	89,741733	18,570664	13,691835
mmu-miR-361-5p	MIMAT0000704	202,997984	64,12858	33,12762	104,936593	33,602156	44,917503	108,389703	32,851663	22,88367	90,881409	19,71034	14,532099
mmu-miR-3098-3p	MIMAT0014918	175,244755	36,375351	18,790823	86,275108	14,940671	19,971863	91,446329	15,908289	11,081328	81,346119	10,17505	7,501891
mmu-miR-744-3p	MIMAT0004820	169,473649	30,604246	15,809578	77,926694	6,592257	8,812165	86,498712	10,960672	7,634938	76,819016	5,647946	4,164135
mmu-miR-1191b-5p	MIMAT0029866	180,27453	41,405126	21,38911	83,328338	11,993901	16,032783	85,657759	10,11972	7,049151	85,895002	14,723932	10,855705
mmu-miR-7239-5p	MIMAT0028446	154,630118	15,760714	8,141689	67,591311			77,545929			76,406596	5,235527	3,860065
mmu-miR-143-5p	MIMAT0017006	258,220916	119,351512	61,654748	105,251239	33,916803	45,338105	106,784989	31,24695	21,765865	110,309508	39,138438	28,856105
mmu-let-7j	MIMAT0025123	347,208169	208,338765	107,623892	136,405052	65,070615	86,982798	144,871264	69,333225	48,295839	129,89172	58,720651	43,293738
mmu-miR-763	MIMAT0003896	160,07192	21,202517	10,952822	78,354256	7,019819	9,383706	82,81902	7,28098	5,071754	77,253271	6,082202	4,484304
mmu-miR-410-3p	MIMAT0001091	153,618825	14,749421	7,619274	72,642841			78,157051			75,997389	4,82632	3,558364
mmu-miR-22-3p	MIMAT0000531	298,711139	159,841735	82,571237	126,807084	55,472647	74,152765	133,981677	58,443638	40,710417	115,103661	43,932592	32,390753
mmu-miR-6354	MIMAT0025097	590,640788	451,771384	233,376608	161,982164	90,647727	121,172867	184,489402	108,951363	75,89287	191,456049	120,28498	88,684072
mmu-miR-7027-3p	MIMAT0027959	157,773112	18,903708	9,765301	81,506939	10,172502	13,598038	83,339358	7,801318	5,434209	78,509153	7,338084	5,410244
mmu-miR-3470a	MIMAT0015640	211,641402	72,771998	37,592647	97,065958	25,731521	34,396474	99,674869	24,13683	16,813129	93,552135	22,381066	16,501179
mmu-miR-33-5p	MIMAT0000667	195,822371	56,952967	29,420833	87,737562	16,403125	21,926791	96,243151	20,705111	14,422677	83,837688	12,666619	9,338883
mmu-miR-6999-5p	MIMAT0027900	2221,757603	2082,888199	1075,980906	894,052333	822,717896	1099,763771	816,362552	740,824512	516,040344	735,281777	664,110708	489,637539
mmu-miR-3470b	MIMAT0015641	254,994824	116,12542	59,98821	101,768686	30,434249	40,68282	101,828542	26,290502	18,313325	109,34736	38,17629	28,14673
mmu-miR-700-5p	MIMAT0017256	157,30996	18,440556	9,526045	72,141888			76,108061			77,110382	5,939312	4,378954
mmu-miR-6948-5p	MIMAT0027796	157,049069	18,179665	9,391273	70,657122			76,319037			77,007036	5,835966	4,302759
mmu-miR-7000-3p	MIMAT0027903	254,810795	115,941391	59,893144	103,329343	31,994906	42,769021	101,287849	25,749809	17,936691	110,592017	39,420947	29,064395
mmu-miR-3105-5p	MIMAT0014941	190,415343	51,545939	26,627664	85,328338	13,993901	18,706273	90,302361	14,764321	10,284467	85,285649	14,114579	10,406439
mmu-miR-30c-5p	MIMAT0000514	811,547769	672,678365	347,493004	284,678098	213,343662	285,186005	335,86054	260,3225	181,334324	215,97221	144,801141	106,75942
mmu-miR-142a-5p	MIMAT0000154	1187,816385	1048,946981	541,866301	457,116245	385,781808	515,69178	508,116887	432,578848	301,323908	310,531454	239,360385	176,476344
mmu-miR-6987-5p	MIMAT0027876	615,150961	476,281557	246,038103	219,5141	148,179664	198,078377	198,226043	122,688004	85,46148	225,175852	154,004783	113,545109
mmu-miR-295-5p	MIMAT0004575	188,655522	49,786118	25,718573	88,281583	16,947146	22,654007	86,763733	11,225693	7,819545	89,933023	18,761954	13,83287
mmu-miR-770-5p	MIMAT0004822	167,587765	28,718361	14,835366	78,847709	7,513273	10,043327	84,370463	8,832424	6,152452	77,920727	6,749658	4,976408
mmu-miR-6957-5p	MIMAT0027814	194,101462	55,232058	28,531843	82,173442	10,839006	14,488983	83,963442	8,425403	5,868931	89,284916	18,113847	13,355031
mmu-miR-7002-5p	MIMAT0027906	681,859382	542,989978	280,49842	208,713845	137,379408	183,641193	207,820168	132,282129	92,144515	227,122485	155,951415	114,980328
mmu-miR-15b-5p	MIMAT0000124	356,864795	217,995391	112,612323	142,176233	70,841796	94,697395	153,463015	77,924975	54,280643	122,495379	51,32431	37,840541
mmu-miR-6350	MIMAT0025093	154,342869	15,473465	7,993301	70,513208			76,966381			75,985319	4,81425	3,549465
mmu-miR-6345	MIMAT0025088	368,622087	229,752683	118,685919	90,192579	18,858142	25,208521	117,991994	42,453955	29,572393	116,616214	45,445144	33,505933
mmu-miR-185-5p	MIMAT0000214	218,44889	79,579486	41,109267	94,000947	22,666511	30,299337	95,67926	20,141221	14,029885	94,363269	23,1922	17,099215
mmu-miR-28c	MIMAT0019339	159,046806	20,177402	10,423267	76,147031	4,812594	6,43321	86,066219	10,528179	7,333674	71,323331		
mmu-miR-6540-5p	MIMAT0025585	168,654569	29,785165	15,386457	82,955399	11,620963	15,53426	88,604637	13,066597	9,101874	76,935221	5,764152	4,249811
mmu-miR-344c-5p	MIMAT0014927	152,669295	13,799891	7,128765	76,885246	5,550809	7,420014	84,516018	8,977978	6,253841	73,54864		
mmu-miR-16-1-3p	MIMAT0004625	157,880968	19,011564	9,821017	69,536401			79,043734			76,882762	5,711693	4,211134
mmu-miR-3066-5p	MIMAT0014838	159,092632	20,223228	10,44694	70,3662			77,075307			77,224952	6,053883	4,463425
mmu-miR-301b-3p	MIMAT0004186	158,39372	19,524316	10,085895	85,020329	13,685892	18,294543	87,083119	11,545079	8,042021	76,701964	5,530895	4,077835
mmu-miR-7223-5p	MIMAT0028414	155,985043	17,115639	8,841617	71,332988			78,685186			76,275777	5,104708	3,763614
mmu-miR-466o-3p	MIMAT0014886	156,013308	17,143904	8,856219	69,545969			80,926962	5,388922	3,753792	73,246669		
mmu-miR-7235-3p	MIMAT0028439	156,779058	17,909654	9,251791	70,507983			72,620071			76,486415	5,315346	3,918914
mmu-miR-340-5p	MIMAT0004651	204,359783	65,490379	33,831099	94,197292	22,862855	30,561799	97,506733	21,968694	15,302858	87,311543	16,140474	11,900097
mmu-miR-26b-5p	MIMAT0000534	609,501614	470,63221	243,119757	210,339513	139,005076	185,814296	231,173061	155,635022	108,411572	169,542928	98,371859	72,5279
mmu-miR-450a-5p	MIMAT0001546	163,197079	24,327676	12,56722	79,270477	7,93604	10,60846	82,531466	6,993426	4,871451	77,81817	6,647101	4,900794
mmu-miR-500-3p	MIMAT0003507	462,445294	323,575891	167,153225	184,229917	112,89548	150,912433	178,06088	102,522841	71,414918	155,143205	83,972135	61,911228
mmu-miR-31-5p	MIMAT0000538	159,26915	20,399746	10,538125	75,912351			80,325655			77,132944	5,961875	4,395589
mmu-miR-107-3p	MIMAT0000647	383,315677	244,446273	126,276351	143,876911	72,542475	96,970767	147,347065	71,809026	50,020422	129,449347	58,278277	42,967583
mmu-miR-23a-3p	MIMAT0000532	974,5242	835,654796	431,683566	393,145641	321,811205	430,179416	373,974982	298,436942	207,883918	275,788363	204,617293	150,860853
mmu-miR-7034-5p	MIMAT0027972	325,270281	186,400877	96,291191	133,780634	62,446197	83,474621	120,951394	45,413355	31,633839	129,620555	58,449485	43,093812
mmu-miR-101a-3p	MIMAT0000133	195,228147	56,358743	29,113868	89,316926	17,982489	24,037996	93,991819	18,453779	12,854454	83,496231	12,325161	9,087132
mmu-miR-1964-3p	MIMAT0009437	155,849327	16,979923	8,771509	80,175736	8,8413	11,81856	87,72922	12,191181	8,49208	75,767402		
mmu-let-7a-2-3p	MIMAT0017015	156,098287	17,228883	8,900117	72,00533			72,052564			76,149136	4,978066	3,670244
mmu-miR-6996-5p	MIMAT0027894	167,539193	28,66979	14,810275	74,839985			72,601265			79,262974	8,091904	5,966024
mmu-miR-27b-3p	MIMAT0000126	430,617393	291,747989	150,711529	176,016812	104,682375	139,933608	177,474003	101,935963	71,006113	132,267027	61,095958	45,045011
mmu-miR-30b-5p	MIMAT0000130	703,356131	564,486727	291,603237	287,6262	216,291763	289,126864	253,671963	178,133924	124,08376	216,538744	145,367675	107,177116
mmu-miR-6928-5p	MIMAT0027756	163,714459	24,845055	12,834488	89,872789	18,538352	24,781044	87,88609	12,34805	8,601352	79,784383	8,613314	6,35045
mmu-miR-1247-3p	MIMAT0014801	5904,117567	5765,248163	2978,218873	2235,118472	2163,784036	2892,4268	1805,979549	1730,441509	1205,383483	1588,931226	1517,760157	1119,018771
mmu-miR-99b-3p	MIMAT0004525	165,633857	26,764454	13,826014	82,120744	10,786308	14,418539	91,320503	15,782463	10,99368	74,610745		
mmu-miR-344f-3p	MIMAT0014932	153,247824	14,37842	7,427622	78,328932	6,994495	9,349854	84,85111	9,31307	6,487258	71,463797		
mmu-miR-6541	MIMAT0025588	182,373936	43,504532	22,473624	81,999061	10,664624	14,255879	82,592644	7,054605	4,914066	83,751323	12,580254	9,275207
mmu-miR-142a-3p	MIMAT0000155	2583,113546	2444,244142	1262,650597	945,663891	874,329454	1168,755247	865,63068	790,09264	550,359324	563,210381	492,039312	362,772223
mmu-miR-678	MIMAT0003452	177,41187	38,542466	19,910314	81,265621	9,931184	13,275458	84,988656	9,450617	6,58307	79,117717	7,946647	5,858928
mmu-miR-9768-3p	MIMAT0036460	164,294256	25,424852	13,134001	79,72072	8,386283	11,210319	80,828576	5,290537	3,685259	78,513888	7,342819	5,413736
mmu-miR-29b-3p	MIMAT0000127	697,676023	558,806619	288,669	265,525004	194,190567	259,583208	239,21001	163,67197	114,009915	192,997653	121,826584	89,82067
mmu-miR-342-3p	MIMAT0000590	230,600035	91,730631	47,38632	95,495364	24,160927	32,29699	99,999372	24,461333	17,03917	87,808749	16,63768	12,266679
mmu-miR-374c-5p	MIMAT0014953	256,152901	117,283497	60,586451	120,084175	48,749738	65,165953	117,607507	42,069467	29,304568	93,150877	21,979808	16,205339
mmu-miR-140-5p	MIMAT0000151	222,760931	83,891527	43,336787	97,471208	26,136771	34,938189	90,459034	14,920994	10,393602	94,921003	23,749933	17,510422
mmu-miR-103-3p	MIMAT0000546	403,984074	265,11467	136,953257	141,635675	70,301239	93,974807	143,74229	68,20425	47,509423	118,380583	47,209513	34,806772
mmu-miR-673-5p	MIMAT0003739	161,427209	22,557805	11,652938	73,553325			74,682492			76,779472	5,608403	4,13498
mmu-miR-1195	MIMAT0005856	1286,650639	1147,781235	592,922219	459,705239	388,370802	519,152604	361,965522	286,427482	199,51842	331,024144	259,853075	191,585256
mmu-miR-6927-5p	MIMAT0027754	418,828735	279,959331	144,62173	142,796226	71,46179	95,526167	130,580548	55,042508	38,341273	133,024124	61,853054	45,603206
mmu-miR-339-5p	MIMAT0000584	165,112722	26,243318	13,556805	83,278202	11,943765	15,965764	81,676356	6,138316	4,275802	79,34206	8,170991	6,024333
mmu-miR-706	MIMAT0003496	628,425067	489,555664	252,895257	237,523793	166,189356	222,152738	207,907077	132,369037	92,205053	170,901254	99,730184	73,52937
mmu-miR-340-3p	MIMAT0000586	152,727933	13,858529	7,159056	76,267465	4,933028	6,594199	82,410351	6,872311	4,787085	75,750365		
mmu-miR-686	MIMAT0003464	161,028979	22,159575	11,44722	67,610611			81,223346	5,685307	3,960246	73,188566		
mmu-miR-5617-5p	MIMAT0022361	330,714935	191,845531	99,103797	139,605208	68,270771	91,26059	126,747958	51,209918	35,671584	111,929409	40,75834	30,050431
mmu-miR-6361	MIMAT0025104	152,51689	13,647486	7,050035	76,30582	4,971383	6,64547	77,19668			77,500239	6,32917	4,66639
mmu-miR-374b-5p	MIMAT0003727	294,873229	156,003825	80,588645	109,787741	38,453304	51,40225	119,651589	44,113549	30,728426	89,287227	18,116158	13,356735
mmu-miR-1902	MIMAT0007863	4419,04311	4280,173706	2211,057313	1762,433434	1691,098997	2260,567589	1142,950115	1067,412075	743,5333	1086,759024	1015,587955	748,77574
mmu-miR-21a-5p	MIMAT0000530	1267,133534	1128,26413	582,84005	414,860624	343,526187	459,206803	377,277984	301,739944	210,184709	255,514428	184,343359	135,913226
mmu-miR-29a-3p	MIMAT0000535	891,528623	752,659219	388,80961	345,670583	274,336147	366,717385	275,917221	200,379182	139,579266	217,334651	146,163582	107,763925
mmu-let-7g-5p	MIMAT0000121	930,253346	791,383942	408,814074	371,380935	300,046498	401,08556	330,680201	255,142162	177,725826	189,612588	118,441518	87,324919
mmu-miR-7007-3p	MIMAT0027919	209,447315	70,577911	36,459223	82,680521	11,346084	15,166817	85,968559	10,43052	7,265647	83,121832	11,950763	8,811094
mmu-miR-3154	MIMAT0035714	40601	40462,1306	20901,97639	11398,91723	11327,58279	15142,08604	8285,056421	8209,518381	5718,550906	7452,169879	7380,99881	5441,884988
mmu-miR-7649-5p	MIMAT0029800	177,126842	38,257438	19,763074	79,372593	8,038156	10,744963	81,227283	5,689243	3,962989	78,606799	7,43573	5,482237
mmu-miR-7679-3p	MIMAT0029873	174,864957	35,995553	18,594626	82,26599	10,931553	14,612695	83,239988	7,701949	5,36499	77,72058	6,549511	4,828843
mmu-miR-7093-5p	MIMAT0028092	205,967207	67,097803	34,661464	96,225628	24,891191	33,273167	93,441876	17,903836	12,471377	82,367898	11,196829	8,255232
mmu-let-7i-5p	MIMAT0000122	781,499415	642,630011	331,970589	242,723949	171,389512	229,104019	223,127847	147,589807	102,807471	163,03196	91,860891	67,727474
mmu-miR-191-5p	MIMAT0000221	599,500993	460,631589	237,953623	236,224184	164,889747	220,415492	184,114939	108,576899	75,632028	156,342499	85,17143	62,795448
mmu-miR-5133	MIMAT0020644	178,505008	39,635604	20,475008	78,100358	6,765921	9,044309	82,833484	7,295444	5,081829	76,279898	5,108829	3,766653
mmu-miR-10b-5p	MIMAT0000208	185,417259	46,547855	24,045747	83,842322	12,507885	16,719848	86,950479	11,41244	7,949628	76,848856	5,677787	4,186136
mmu-miR-7025-3p	MIMAT0027955	201,706332	62,836928	32,460376	102,808803	31,474366	42,073192	89,621795	14,083755	9,810402	87,805536	16,634467	12,26431

mmu-miR-219a-5p	MIMAT0000664	159,419673	20,550269	10,615883	76,600798	5,266361	7,039781	82,937244	7,399205	5,154106	70,683292		
mmu-miR-3552	MIMAT0035715	201,044052	62,174648	32,118255	85,317069	13,982632	18,69121	82,917195	7,379156	5,140141	84,205773	13,034704	9,610265
mmu-miR-29c-3p	MIMAT0000536	370,709612	231,840208	119,764295	149,330597	77,99616	104,260952	128,993914	53,455875	37,236063	107,127808	35,956739	26,510292
mmu-miR-28a-5p	MIMAT0000653	175,903247	37,033843	19,130987	87,913807	16,57937	22,162385	86,538809	11,00077	7,662869	76,511464	5,340395	3,937382
mmu-miR-5100	MIMAT0020607	4604,854846	4465,985442	2307,044164	1672,77266	1601,438223	2140,714026	1014,450288	938,912249	654,023445	875,709019	804,537949	593,172158
mmu-miR-7049-5p	MIMAT0028002	382,747794	243,87839	125,982994	141,492561	70,158124	93,783499	115,686827	40,148788	27,966669	115,989147	44,818077	33,043607
mmu-miR-3090-3p	MIMAT0014902	164,91046	26,041056	13,45232	71,748338			79,95145			76,227377	5,056307	3,727929
mmu-miR-146b-5p	MIMAT0003475	346,042364	207,172961	107,021659	149,553147	78,21871	104,558444	118,856932	43,318892	30,174887	108,289313	37,118244	27,36665
mmu-miR-7065-5p	MIMAT0028034	154,150672	15,281269	7,894016	78,165718	6,831281	9,131679	75,688851			77,309739	6,13867	4,525937
mmu-miR-362-5p	MIMAT0000706	155,652098	16,782694	8,669625	77,776593	6,442156	8,611518	80,627883			77,39046	6,219391	4,585451
mmu-miR-3069-3p	MIMAT0014845	216,244804	77,3754	39,970678	92,957818	21,623381	28,904939	92,029542	16,491502	11,48758	80,364622	9,193553	6,77825
mmu-miR-107-5p	MIMAT0017048	158,901705	20,032301	10,34831	76,927145	5,592708	7,476022	76,787495			77,569623	6,398554	4,717545
mmu-miR-7227-3p	MIMAT0028423	184,988889	46,119485	23,824459	80,037047	8,70261	11,633168	83,07211	7,534071	5,24805	76,342004	5,170934	3,812442
mmu-miR-150-5p	MIMAT0000160	531,373887	392,504483	202,760441	234,676169	163,341732	218,346192	167,237331	91,699291	63,875497	129,120861	57,949792	42,725397
mmu-miR-6902-3p	MIMAT0027705	160,813542	21,944138	11,335929	79,229847	7,895411	10,554148	83,335421	7,797381	5,431466	75,475923		
mmu-miR-23b-3p	MIMAT0000125	1059,055457	920,186053	475,350825	395,708636	324,374199	433,605485	270,418949	194,880909	135,749303	183,112894	111,941825	82,532805
mmu-miR-145a-5p	MIMAT0000157	597,589638	458,720234	236,966253	241,731099	170,396662	227,776832	180,149241	104,611202	72,86962	123,625183	52,454114	38,673527
mmu-miR-28a-3p	MIMAT0004661	153,973758	15,104354	7,802626	76,918026	5,58359	7,463834	77,495759			76,043365	4,872296	3,592261
mmu-miR-425-5p	MIMAT0004750	274,419123	135,549719	70,022438	110,87107	39,536633	52,850383	103,287701	27,749661	19,329739	83,673871	12,502802	9,218104
mmu-miR-223-3p	MIMAT0000665	483,94483	345,075426	178,259481	192,591587	121,25715	162,08985	137,146616	61,608577	42,915037	119,574472	48,403403	35,687006
mmu-miR-7077-3p	MIMAT0028061	154,525662	15,656259	8,087729	80,454078	9,119641	12,190631	78,740732			77,275044	6,103975	4,500357
mmu-miR-6924-5p	MIMAT0027748	1562,535705	1423,666301	735,439261	587,262915	515,928479	689,664649	289,110279	213,572239	148,769229	286,085642	214,914572	158,452862
mmu-miR-3110-3p	MIMAT0014952	164,843001	25,973597	13,417472	80,963287	9,62885	12,871314	80,445073			78,839999	7,66893	5,654172
mmu-miR-6990-3p	MIMAT0027883	176,728247	37,858843	19,557167	69,599999			81,454371	5,916332	4,121173	67,587224		
mmu-miR-503-5p	MIMAT0003188	160,40901	21,539606	11,126956	80,661384	9,326947	12,467747	78,931659			77,57848	6,407411	4,724075
mmu-miR-199a-3p, mmu	MIMAT0000230, MIMA	283,102512	144,233108	74,508114	110,113637	38,7792	51,83789	95,095579	19,55754	13,623307	86,489917	15,318848	11,294326
mmu-miR-7027-5p	MIMAT0027958	701,480287	562,610883	290,634211	254,511768	183,177331	244,861322	139,408489	63,870449	44,490602	151,794469	80,623399	59,442262
mmu-miR-130a-3p	MIMAT0000141	313,01968	174,150277	89,962761	133,006708	61,672271	82,44008	106,617111	31,079072	21,648926	87,131535	15,960465	11,76738
mmu-miR-6416-3p	MIMAT0025171	540,659569	401,790165	207,557249	232,605072	161,270635	215,577664	130,304002	54,765962	38,148638	130,667376	59,496307	43,865616
mmu-miR-703	MIMAT0003493	163,09138	24,221976	12,512618	77,960706	6,626269	8,85763	81,17881	5,64077	3,929223	68,610695		
mmu-miR-6538	MIMAT0025583	16777,29202	16638,42262	8595,096491	7532,49996	7461,165523	9973,673324	2632,866121	2557,328082	1781,372565	2285,3798	2214,208731	1632,498469
mmu-miR-6402	MIMAT0025154	323,713352	184,843948	95,48691	127,96366	56,629223	75,698812	100,957048	25,419009	17,706264	89,619828	18,448758	13,601956
mmu-miR-214-3p	MIMAT0000661	504,895321	366,025917	189,082111	207,152656	135,818219	181,554282	118,778079	43,240039	30,11996	120,440377	49,269308	36,325423
mmu-miR-5710	MIMAT0022505	153,751779	14,882375	7,687955	83,613666	12,279229	16,414194	81,700586	6,162546	4,29268	71,728535		
mmu-miR-151-5p	MIMAT0004536	167,142812	28,273408	14,605511	77,188111	5,853675	7,824868	79,960294			76,372634	5,201564	3,835025
mmu-miR-652-3p	MIMAT0003711	179,510654	40,64125	20,994506	85,770324	14,435887	19,297095	80,696453			80,419568	9,248499	6,818761
mmu-miR-6238	MIMAT0024859	3030,83553	2891,966126	1493,935361	1236,697534	1165,363097	1557,792921	464,057991	388,519951	270,633552	401,704066	330,532996	243,696361
mmu-miR-204-5p	MIMAT0000237	167,865006	28,995602	14,978583	77,626491	6,292054	8,41087	80,347204			76,227377	5,056307	3,727929
mmu-miR-3106-3p	MIMAT0014818	162,910427	24,041023	12,419141	81,00951	9,675074	12,933103	79,92086			76,020684	4,849615	3,575539
mmu-miR-146a-5p	MIMAT0000158	505,907882	367,038478	189,605181	204,496252	133,161815	178,003348	121,310396	45,772356	31,883911	94,724865	23,553796	17,365813
mmu-miR-126a-5p	MIMAT0000137	210,151658	71,282254	36,823073	106,701032	35,366595	47,276107	91,319003	15,780963	10,992635	71,889984		
mmu-miR-322-5p	MIMAT0000548	263,536688	124,667284	64,400776	111,791203	40,456767	54,080368	85,132563	9,594523	6,683312	82,865894	11,694825	8,622395
mmu-miR-1983	MIMAT0009455	255,085483	116,216079	60,035043	104,409578	33,075141	44,213019	84,50186	8,963821	6,24398	80,272676	9,101607	6,71046
mmu-miR-6412	MIMAT0025165	2384,086545	2245,217141	1159,836988	868,113101	796,778664	1065,089641	254,54749	179,00945	124,693631	227,056377	155,885307	114,931588
mmu-miR-7075-3p	MIMAT0028057	191,65049	52,781086	27,265717	88,668146	17,333709	23,170743	83,208415	7,670375	5,342997	73,113482		
mmu-miR-7680-5p	MIMAT0029874	178,21523	39,345826	20,325314	85,791304	14,456868	19,325141	73,694485			76,434802	5,263732	3,88086
mmu-miR-6516-3p	MIMAT0027344	317,582919	178,713515	92,320044	124,159185	52,824748	70,613201	89,231416	13,693376	9,538473	79,752056	8,580987	6,326616
mmu-miR-451a	MIMAT0001632	4343,529781	4204,660377	2172,048546	1461,878999	1390,544562	1858,803047	336,079314	260,541274	181,486717	298,197895	227,026826	167,38302
mmu-miR-7077-5p	MIMAT0028060	1486,190152	1347,320748	696,000582	614,837221	543,502784	726,524455	174,775749	99,237709	69,126576	140,142979	68,97191	50,851817
mmu-miR-3968	MIMAT0019352	1316,714632	1177,845228	608,452713	407,183077	335,84864	448,943883	128,286376	52,748336	36,743209	134,501981	63,330912	46,692805
mmu-miR-144-3p	MIMAT0000156	445,441862	306,572458	158,369571	174,602363	103,267926	138,04285	95,523103	19,985064	13,921109	83,551177	12,380107	9,127643
mmu-miR-10a-5p	MIMAT0000648	187,261686	48,392282	24,998544	92,032909	20,698472	27,668573	79,791628			76,691529	5,52046	4,070141
mmu-miR-3963	MIMAT0019341	2125,082406	1986,213003	1026,040316	813,162313	741,827877	991,634468	159,62868	84,090641	58,575496	145,35192	74,180851	54,692281
mmu-miR-497a-5p	MIMAT0003453	237,803921	98,934517	51,107713	108,707937	37,3735	49,958828	83,16341	7,62537	5,311648	71,261226		
mmu-miR-692	MIMAT0003471	220,324991	81,455587	42,078426	105,08067	33,746233	45,110097	75,730239			76,797243	5,626174	4,148082
mmu-miR-126a-3p	MIMAT0000138	1048,703404	909,834	470,003149	352,668864	281,334427	376,072299	112,150622	36,612583	25,503435	86,327616	15,156547	11,174664
mmu-miR-494-3p	MIMAT0003182	13121,70022	12982,83082	6706,686455	4835,039778	4763,705341	6367,857775	466,29	390,75196	272,188315	348,689571	277,518502	204,609675
mmu-miR-501-3p	MIMAT0003509	387,041345	248,171941	128,200961	151,949196	80,61476	107,761351	85,093659	9,55562	6,656212	72,273353		
mmu-miR-690	MIMAT0003469	8636,908749	8498,039345	4389,927449	2932,870067	2861,53563	3825,142532	168,69596	93,157921	64,891542	158,059207	86,888138	64,061148

Supplementary table S4

miRNA only expressed in thymus

1: Raw data

2: Background substracted data

3: Global normalization

		ThymusExo 1 (T1)			ThymusExo2 (T2)		
**miRNA**	**Mature sequence**	1	2	3	1	2	3
mmu-miR-465	MIMAT0004217, MIMAT0004872, MIMAT(	81,459278	5,921239	4,124591	80,15156	8,980491	6,621163
mmu-miR-466	MIMAT0002107, MIMAT0004880	88,945572	13,407532	9,339361	81,874018	10,702949	7,891102
mmu-let-7i-3p	MIMAT0004520	88,061828	12,523788	8,723766	81,267765	10,096696	7,444122
mmu-miR-119	MIMAT0017329	89,713305	14,175265	9,874145	92,361691	21,190622	15,623485
mmu-miR-125	MIMAT0004529	84,72751	9,189471	6,401162	83,502776	12,331707	9,091958
mmu-miR-133	MIMAT0017083	87,917663	12,379624	8,623345	83,087024	11,915955	8,785431
mmu-miR-133	MIMAT0025078	95,100487	19,562447	13,626725	94,039695	22,868626	16,860649
mmu-miR-138	MIMAT0004668	80,90128	5,36324	3,735903	80,397006	9,225937	6,802126
mmu-miR-148	MIMAT0000580	86,995742	11,457702	7,981157	82,131478	10,960409	8,080923
mmu-miR-151	MIMAT0000161	84,083119	8,545079	5,952294	75,970649	4,79958	3,538649
mmu-miR-152	MIMAT0016991	82,263492	6,725452	4,684787	81,2663	10,095231	7,443042
mmu-miR-155	MIMAT0016993	82,943136	7,405096	5,15821	76,496118	5,325049	3,926068
mmu-miR-181	MIMAT0000660	98,651624	23,113584	16,100361	89,159797	17,988727	13,262783
mmu-miR-181	MIMAT0017264	84,057437	8,519397	5,934405	80,179822	9,008753	6,642
mmu-miR-189	MIMAT0007865	135,706842	60,168802	41,912125	104,11077	32,939701	24,285882
mmu-miR-193	MIMAT0004859	83,902006	8,363966	5,826135	82,095937	10,924868	8,05472
mmu-miR-195	MIMAT0009430	85,158245	9,620205	6,701201	79,533293	8,362224	6,165326
mmu-miR-195	MIMAT0017000	84,134273	8,596233	5,987927	78,291179	7,12011	5,249536
mmu-miR-196	MIMAT0022952	86,578697	11,040657	7,690653	78,311374	7,140305	5,264425
mmu-miR-200	MIMAT0000657	142,072216	66,534177	46,34609	132,407436	61,236366	45,148532
mmu-miR-203	MIMAT0000236	121,734785	46,196745	32,179529	87,171923	16,000854	11,797158
mmu-miR-205	MIMAT0000238	173,872892	98,334852	68,497667	147,155951	75,984882	56,022362
mmu-miR-206	MIMAT0000239	82,58412	7,046081	4,908128	79,517164	8,346095	6,153434
mmu-miR-20b	MIMAT0004788	80,732417	5,194378	3,618277	78,830297	7,659227	5,647018
mmu-miR-212	MIMAT0017053	82,697179	7,159139	4,986882	77,294393	6,123323	4,514622
mmu-miR-290	MIMAT0004572	91,780843	16,242803	11,314342	79,634272	8,463203	6,239775
mmu-miR-292	MIMAT0029864	107,383812	31,845772	22,182991	98,121268	26,950199	19,869924
mmu-miR-305	MIMAT0014822	82,302151	6,764111	4,711716	82,359887	11,188818	8,249325
mmu-miR-306	MIMAT0014844	96,343701	20,805661	14,492718	84,894269	13,7232	10,117882
mmu-miR-308	MIMAT0014875	80,936518	5,398479	3,760449	75,951357	4,780288	3,524425
mmu-miR-309	MIMAT0014904	89,831013	14,292973	9,956138	84,017697	12,846627	9,4716
mmu-miR-309	MIMAT0014907	90,842279	15,30424	10,660561	95,834638	24,663568	18,18403
mmu-miR-310	MIMAT0014942	89,459292	13,921253	9,697206	79,709299	8,53823	6,295092
mmu-miR-310	MIMAT0014949	81,665606	6,127566	4,268314	78,463853	7,292784	5,376846
mmu-miR-329	MIMAT0000567	82,588253	7,050214	4,911007	87,233183	16,062114	11,842324
mmu-miR-342	MIMAT0004653	85,358938	9,820899	6,840999	84,09357	12,922501	9,52754
mmu-miR-345	MIMAT0004656	82,004314	6,466275	4,50425	83,239616	12,068547	8,897934
mmu-miR-34b	MIMAT0004581	92,457534	16,919494	11,785708	85,158162	13,987093	10,312446
mmu-miR-353	MIMAT0031410	82,951192	7,413152	5,163821	78,333203	7,162134	5,28052
mmu-miR-434	MIMAT0001422	102,237015	26,698975	18,597857	84,492284	13,321215	9,821506
mmu-miR-449	MIMAT0017180	101,874063	26,336023	18,345033	97,080817	25,909748	19,102817
mmu-miR-449	MIMAT0022715	81,784556	6,246516	4,351171	78,665691	7,494622	5,525657
mmu-miR-466	MIMAT0004931	88,913014	13,374975	9,316682	84,464022	13,292953	9,800668
mmu-miR-466	MIMAT0004883	82,13132	6,593281	4,592719	80,247684	9,076615	6,692034
mmu-miR-468	MIMAT0002109	86,01119	10,47315	7,295342	78,934488	7,763419	5,723837
mmu-miR-488	MIMAT0003449	85,347414	9,809374	6,832972	76,13949	4,96842	3,663132
mmu-miR-496	MIMAT0017244	87,913789	12,375749	8,620646	82,216152	11,045083	8,143352
mmu-miR-510	MIMAT0020610	92,048606	16,510567	11,500859	89,94515	18,77408	13,84181
mmu-miR-510	MIMAT0020611	88,370673	12,832634	8,938901	76,175763	5,004694	3,689876
mmu-miR-510	MIMAT0022985	81,290416	5,752377	4,006966	79,036143	7,865074	5,798785
mmu-miR-512	MIMAT0020635	84,182216	8,644177	6,021323	77,265398	6,094328	4,493245
mmu-miR-513	MIMAT0022989	139,921951	64,383911	44,848267	113,06175	41,890681	30,885287
mmu-miR-539	MIMAT0003169	81,10538	5,567341	3,878074	79,873905	8,702836	6,416453
mmu-miR-551	MIMAT0017236	106,505701	30,967661	21,57132	90,95085	19,77978	14,583296
mmu-miR-562	MIMAT0022371	97,987638	22,449598	15,637844	85,47209	14,301021	10,5439

mmu-miR-562	MIMAT0022379	95,922326	20,384287	14,199198	84,351819	13,18075	9,717943
mmu-miR-636	MIMAT0025107	84,160471	8,622432	6,006176	78,571202	7,400132	5,455992
mmu-miR-637	MIMAT0025122	86,266654	10,728615	7,473292	78,199171	7,028102	5,1817
mmu-miR-639	MIMAT0025143	81,670723	6,132683	4,271878	80,036143	8,865074	6,536068
mmu-miR-675	MIMAT0003726	83,808996	8,270956	5,761347	76,141801	4,970732	3,664836
mmu-miR-689	MIMAT0027693	83,050093	7,512054	5,232714	78,215363	7,044293	5,193638
mmu-miR-690	MIMAT0027701	85,082393	9,544353	6,648364	80,235501	9,064431	6,683051
mmu-miR-690	MIMAT0027708	96,735532	21,197492	14,765658	91,087193	19,916124	14,68382
mmu-miR-690	MIMAT0027717	89,327317	13,789277	9,605275	81,388093	10,217023	7,532838
mmu-miR-692	MIMAT0027743	88,385088	12,847049	8,948942	77,016738	5,845669	4,309912
mmu-miR-692	MIMAT0027745	83,582362	8,044322	5,603479	81,344484	10,173415	7,500686
mmu-miR-693	MIMAT0027773	81,685912	6,147873	4,282459	79,495441	8,324372	6,137418
mmu-miR-694	MIMAT0027780	84,661682	9,123643	6,355308	81,473668	10,302599	7,595931
mmu-miR-694	MIMAT0027795	83,968608	8,430568	5,872529	80,194316	9,023247	6,652687
mmu-miR-694	MIMAT0027798	93,94754	18,409501	12,823611	92,791825	21,620755	15,940616
mmu-miR-695	MIMAT0027813	81,659714	6,121675	4,26421	76,199114	5,028045	3,707092
mmu-miR-697	MIMAT0027859	85,610528	10,072489	7,016251	83,423633	12,252564	9,033607
mmu-miR-698	MIMAT0027875	86,17881	10,64077	7,412102	86,193584	15,022514	11,075844
mmu-miR-698	MIMAT0027877	83,831061	8,293021	5,776717	83,802091	12,631022	9,312638
mmu-miR-698	MIMAT0027879	81,477163	5,939123	4,137049	79,367165	8,196096	6,042842
mmu-miR-700	MIMAT0027917	104,92202	29,38398	20,468166	94,230872	23,059803	17,001601
mmu-miR-700	MIMAT0027916	108,238195	32,700156	22,778133	101,744327	30,573257	22,541143
mmu-miR-702	MIMAT0027947	87,286703	11,748664	8,183833	78,71488	7,543811	5,561924
mmu-miR-702	MIMAT0027956	90,014548	14,476509	10,083984	80,578593	9,407524	6,936007
mmu-miR-703	MIMAT0027969	84,440424	8,902384	6,201184	76,559921	5,388851	3,973109
mmu-miR-703	MIMAT0027978	87,891207	12,353168	8,604916	80,112806	8,941737	6,59259
mmu-miR-709	MIMAT0028088	85,748543	10,210504	7,112389	94,051653	22,880583	16,869465
mmu-miR-711	MIMAT0028131	133,750136	58,212096	40,549131	144,110764	72,939695	53,777198
mmu-miR-721	MIMAT0028404	92,749527	17,211488	11,989104	82,191949	11,020879	8,125507
mmu-miR-722	MIMAT0028408	96,7052	21,167161	14,74453	92,969347	21,798277	16,071499
mmu-miR-722	MIMAT0028425	102,34273	26,80469	18,671496	77,128998	5,957928	4,392679
mmu-miR-723	MIMAT0028433	96,33466	20,796621	14,48642	95,329364	24,158294	17,8115
mmu-miR-723	MIMAT0028440	87,151689	11,613649	8,089786	93,898441	22,727371	16,756505
mmu-miR-723	MIMAT0028445	83,641376	8,103336	5,644587	79,641488	8,470418	6,245096
mmu-miR-724	MIMAT0028451	83,757841	8,219802	5,725714	87,095148	15,924079	11,740553
mmu-miR-764	MIMAT0003895	90,621279	15,083239	10,506618	84,339692	13,168623	9,709002
mmu-miR-765	MIMAT0029811	90,765185	15,227145	10,606859	79,456687	8,285618	6,108845
mmu-miR-766	MIMAT0029829	83,368753	7,830713	5,454685	79,806156	8,635087	6,366503
mmu-miR-766	MIMAT0029838	96,361843	20,823803	14,505355	93,440721	22,269652	16,419036
mmu-miR-766	MIMAT0029842	81,75789	6,21985	4,332596	80,848913	9,677843	7,13531
mmu-miR-767	MIMAT0029852	101,025054	25,487015	17,753635	111,75899	40,587921	29,924784
mmu-miR-767	MIMAT0029869	87,758099	12,22006	8,512197	84,860307	13,689237	10,092842
mmu-miR-768	MIMAT0029908	86,971512	11,433472	7,964279	82,359041	11,187972	8,248702
mmu-miR-809	MIMAT0031399	101,20838	25,670341	17,881335	92,368913	21,197844	15,62881
mmu-miR-811	MIMAT0031417	84,431642	8,893602	6,195067	79,208873	8,037804	5,926136
mmu-miR-873	MIMAT0017279	89,292853	13,754813	9,581268	78,311374	7,140305	5,264425
mmu-miR-878	MIMAT0004933	104,434498	28,896458	20,12857	93,640135	22,469066	16,56606
mmu-miR-96-	MIMAT0000541	88,135515	12,597476	8,775095	76,944868	5,773798	4,256923
mmu-miR-976	MIMAT0036462	81,569189	6,031149	4,201152	81,59642	10,425351	7,686434

miRNA only expressed in spleen

1: Raw data

2: Background substracted data

3: Global normalization

miRNA	Mature sequence	SpleenExo1 1 (S1)			SpleenExo2 (S2)		
		1	2	3	1	2	3
mmu-miR-119	MIMAT0029867	164,695015	25,825611	13,341026	83,966218	12,631781	16,885466
mmu-miR-124	MIMAT0004527	153,397112	14,527708	7,504741	76,415005	5,080568	6,791422
mmu-miR-130	MIMAT0004583	158,738968	19,869564	10,264243	77,057147	5,72271	7,649802
mmu-miR-144	MIMAT0016988	181,940542	43,071138	22,249741	81,053603	9,719166	12,992043
mmu-miR-195	MIMAT0025076	156,65335	17,783946	9,186853	80,337619	9,003182	12,034956
mmu-miR-22-	MIMAT0004629	157,247558	18,378154	9,493809	77,236035	5,901598	7,88893
mmu-miR-344	MIMAT0017038	152,187288	13,317884	6,879769	76,794561	5,460124	7,298792

mmu-miR-381	MIMAT0000746	157,841782	18,972378	9,800774	76,75215	5,417713	7,242099
mmu-miR-466	MIMAT0014894	157,550761	18,681357	9,650438	83,950093	12,615656	16,863912
mmu-miR-637	MIMAT0025116	167,387385	28,517981	14,731853	79,379068	8,044631	10,753618
mmu-miR-682	MIMAT0003459	154,968476	16,099072	8,316478	79,435822	8,101385	10,829484
mmu-miR-690	MIMAT0027703	211,617306	72,747902	37,580199	105,640652	34,306215	45,85865
mmu-miR-698	MIMAT0027881	152,746771	13,877367	7,168787	78,251709	6,917272	9,246627
mmu-miR-699	MIMAT0027885	158,52063	19,651226	10,151454	82,34895	11,014513	14,723591
mmu-miR-711	MIMAT0028136	151,874683	13,005279	6,718283	77,164918	5,830481	7,793865
